# Conservation status of the forest beetles (Insecta, Coleoptera) from Azores, Portugal

**DOI:** 10.3897/BDJ.5.e14557

**Published:** 2017-10-19

**Authors:** Paulo Alexandre Vieira Borges, Lucas Lamelas-López, Isabel R. Amorim, Anja Danielczak, Rui Nunes, Artur R.M. Serrano, Mário Boieiro, Carla Rego, Axel Hochkirch, Virgílio Vieira

**Affiliations:** 1 CE3C – Centre for Ecology, Evolution and Environmental Changes / Azorean Biodiversity Group and Universidade dos Açores, Dep. de Ciências e Engenharia do Ambiente, Angra do Heroísmo, Açores, Portugal; 2 IUCN SSC Mid-Atlantic Islands Specialist Group, Angra do Heroísmo, Açores, Portugal; 3 Trier University, Department of Biogeography, D-54296 Trier, Germany; 4 Centro de Biologia Ambiental/Departamento de Biologia Animal/, Faculdade de Ciências, Universidade de Lisboa, Lisboa, Lisboa, Portugal; 5 cE3c – Centre for Ecology, Evolution and Environmental Changes / Azorean Biodiversity Group and Universidade dos Açores - Departamento de Biologia, Ponta Delgada, São Miguel, Açores, Portugal

**Keywords:** Beetles, forest species, islands, IUCN, red list, invasive species, climatic changes.

## Abstract

**Background:**

Island biodiversity is under considerable pressure due to the ongoing threats of invasive alien species, land use change or climate change. The few remnants of Azorean native forests harbour a unique set of endemic beetles, some of them possibly already extinct or under severe long term threat due to the small areas of the remaining habitats or climatic changes. In this contribution we present the IUCN Red List profiles of 54 forest adapted beetle species endemic to the Azorean archipelago, including species belonging to four speciose families: Zopheridae (12 species), Carabidae (11 species), Curculionidae (11 species) and Staphylinidae (10 species).

**New information:**

Most species have a restricted distribution (i.e. 66% occur in only one island) and a very small extent of occurrence (EOO) and area of occupancy (AOO). Also common to most of the species is the severe fragmentation of their populations, and a continuing decline in EOO, AOO, habitat quality, number of locations and subpopulations caused by the ongoing threat from pasture intensification, forestry, invasive species and future climatic changes. Therefore, we suggest as future measures of conservation: (1) a long-term monitoring plan for the species; (2) control of invasive species; (3) species-specific conservation action for the most highly threatened species.

## Introduction

The currently known diversity of Azorean endemic beetle taxa totals 76 taxa, including 68 endemic species and the remaining being subspecies (Borges et al. 2010, Borges et al. 2017). Nine of the endemics are cave or lavicolous adapted (most of them belonging to the genus *Trechus*) (see Borges et al. 2004, Borges et al. 2007, three are freshwater species, and the remaining are forest adapted species. Therefore, the remnants of native forests on the Azores harbour a unique set of endemic beetles, some of them possibly already extinct (Terzopoulou et al. 2015) or under severe long term threats due to small sized habitats (Triantis et al. 2010) or climatic changes (Ferreira et al. 2016). Only two Azorean beetle species were so far assessed for the IUCN Red List, the click beetle *Alestrus dolosus* (*Nardi and Mico 2010*), with a current status of Data Deficient (DD) and the longhorn beetle *Crotchiella brachyptera* (Nardi and Mico Balaguer 2010), with a current status of Endangered (EN), both requiring reassessements.

In this contribution we present the IUCN Red List profiles of 54 forest adapted beetle species endemic to the Azores. This group of species includes 12 ironclade beetles (Zopheridae), 11 ground beetles (Carabidae), 11 weevil and bark beetles (Curculionidae) and 10 rove beetles (Staphylinidae) representing the four most speciose beetle families in Azores (Borges et al. 2010, Borges et al. 2017).

## Materials and Methods

To perform the IUCN Red List profiles we went through the following steps: i) the original species descriptions were examined to learn about the habitats and ecology of the species; ii) the most recent literature was also consulted to obtain information about synonyms and critical information for the taxonomic notes; iii) for the calculation of AOO and EOO we consulted the Azorean Biodiversity Portal and downloaded CSV files with the distribution of each species; iv) images of the species were also obtained from the repository available in the Azorean Biodiversity Portal, the most important source of information for Azores biodiversity (see Borges et al. 2010).

Prior to the calculation of area of occupancy (AOO) and extent of occurrence (EOO), the 500 m x 500 m cells obtained from Azorean Biodiversity Portal were filtered to consider only the cells with high level of precision as defined by Borges et al. 2010: 1 – very precise locality, usually point UTM data; 2 – literature locality not exceeding 25 km^2^. The centroid for each cell was calculated to obtain the distribution points for each species. Despite not fully following the guidelines, the calculation of AOO and EOO was performed using the commonly used Geospatial Conservation Assessment Tool (GeoCAT). For EOO calculation Minimum Convex Polygones (MCP) were used without need of species distribution medelling, since in most cases the final polygons covered all the native habitat area. Final maps with species distributions were produced using the IUCN standards with ArcMap 10.

Critical information on species threats and conservation were mostly obtained from Lindroth 1960, Gaston et al. 2006, Machado 2009, Triantis et al. 2010, Meijer et al. 2011, Terzopoulou et al. 2015, Ferreira et al. 2016, Borges et al. 2017.

The raw data on species distributions in the islands, values of AOO, EOO, altitudinal range and number of localities is available in Suppl. material 1.

## Species Conservation Profiles

### Bembidion derelictus

#### Species information

Scientific name: Bembidion derelictus

Species authority: Alluaud, 1926

Synonyms: *Bembidion derelictus* Alluaud, 1926; *Ocydromus derelictus* (Alluaud, 1926); *Peryphus derelictus* Alluaud, 1926.

Common names: Ground beetle (English); Carocho (Portuguese).

Kingdom: Animalia

Phylum: Arthropoda

Class: Insecta

Order: Coleoptera

Family: Carabidae

Taxonomic notes: *Bembidion derelictus* was described from a single male collected on the 31.VII.1888 and deposited in the Museum of Paris (Alluaud 1926). The wings are rudimentary, narrow, parallel-sided, length about one half, width about one third of one elytron (Lindroth 1960).

Region for assessment: Global

#### Editor & Reviewers

##### Reviewers

Reviewers: Anja Danielczak

##### Editor

Editor: Axel Hochkirch

#### Reviewers

Reviewers: Anja Danielczak

#### Editor

Editor: Axel Hochkirch

#### Geographic range

Biogeographic realm: Palearctic

Countries: Portugal

Map of records (Google Earth): Suppl. material 2

Basis of EOO and AOO: Observed

Basis (narrative): This species has a very small extent of occurrence (EOO = 0-12 km²) and area of occupancy (AOO = 0-12 km²).

Min Elevation/Depth (m): 562

Max Elevation/Depth (m): 870

Range description: *Bembidion derelictus* is a single-island endemic species restricted to Flores (Azores, Portugal) (Borges et al. 2010), known from Caldeira Comprida in the Natural Forest Reserve of Morro Alto e Pico da Sé. The species is considered possibly extinct (Terzopoulou et al. 2015).

#### New occurrences

#### Extent of occurrence

EOO (km2): 0-12

Trend: Stable

Justification for trend: The species is considered extinct in the historical locality due to habitat destruction (last sample dates from 1888). It was not sampled recently despite some intensive field work (see Borges et al. 2005, Borges et al. 2016).

Causes ceased?: No

Causes understood?: Yes

Causes reversible?: Unknown

Extreme fluctuations?: Unknown

#### Area of occupancy

Trend: Stable

Justification for trend: The species is considered extinct in the historical locality due to habitat destruction. It was not sampled recently despite some intensive field work.

Causes ceased?: No

Causes understood?: Yes

Causes reversible?: Unknown

Extreme fluctuations?: Unknown

AOO (km2): 0-12

#### Locations

Number of locations: 0-1

Justification for number of locations: The species is potentially extinct due to destruction of the habitat in all its range.

Trend: Stable

Justification for trend: Possibly went extinct more than 10 years ago.

Extreme fluctuations?: Unknown

#### Population

Trend: Stable

Justification for trend: The species is only known from a single subpopulation. According to Terzopoulou et al. 2015 this species was extinct more than ten years ago.

Causes ceased?: No

Causes understood?: Yes

Causes reversible?: Unknown

Extreme fluctuations?: Unknown

#### Subpopulations

Number of subpopulations: 0-1

Trend: Stable

Justification for trend: The species is only known from a single subpopulation. According to Terzopoulou et al. 2015 this species was extinct more than ten years ago.

Extreme fluctuations?: Unknown

Severe fragmentation?: No

#### Habitat

System: Terrestrial

Habitat specialist: Yes

Habitat (narrative): The species occurred in the hyper-humid native forest of the Flores Island (Azores) dominated by *Juniperus brevifolia* woodland, with an altitudinal range between 562 and 870 m. This species is possibly extinct (Terzopoulou et al. 2015).

Trend in extent, area or quality?: Decline (inferred)

Justification for trend: Since the historical record, the native habitat in the island of Flores was greatly reduced to accomodate pastures and *Cryptomeria japonica* plantations (Triantis et al. 2010). In the last ten years invasive plant species are spreading (e.g. *Hedychium gardnerianum*; *Hydrangea macrophylla*) changing the structure of the forest and the cover of bryophytes and ferns in the soil which will impact the species habitat quality.

##### Habitat

Habitat importance: Major Importance

Habitats: 1.4. Forest - Temperate

#### Habitat

Habitat importance: Major Importance

Habitats: 1.4. Forest - Temperate

#### Ecology

Size: 0.6 cm

Generation length (yr): 1

Dependency of single sp?: No

Ecology and traits (narrative): This is a nocturnal predator species that lived in hyper-humid forest floor.

#### Threats

Justification for threats: In the past, the species has probably strongly declined due to changes in habitat size and lack of resources due to its large body size (Terzopoulou et al. 2015). Currently, *Cryptomeria japonica* wood & pulp plantations management and invasive plant species spreading (e.g. *Hedychium gardnerianum*; *Hydrangea macrophylla*) are changing the structure of the forest and the cover of bryophytes and ferns in the soil with impacts on native invertebrate species. Based on Ferreira et al. (2016) the habitat will further decline as a consequence of climate change (increasing number of droughts and habitat shifting & alteration).

##### Threats

Threat type: Ongoing

Threats: 2.2.1. Agriculture & aquaculture - Wood & pulp plantations - Small-holder plantations8.1.2. Invasive and other problematic species, genes & diseases - Invasive non-native/alien species/diseases - Named species

##### Threats

Threat type: Future

Threats: 8. Invasive and other problematic species, genes & diseases11.1. Climate change & severe weather - Habitat shifting & alteration11.2. Climate change & severe weather - Droughts

#### Threats

Threat type: Ongoing

Threats: 2.2.1. Agriculture & aquaculture - Wood & pulp plantations - Small-holder plantations8.1.2. Invasive and other problematic species, genes & diseases - Invasive non-native/alien species/diseases - Named species

#### Threats

Threat type: Future

Threats: 8. Invasive and other problematic species, genes & diseases11.1. Climate change & severe weather - Habitat shifting & alteration11.2. Climate change & severe weather - Droughts

#### Conservation

Justification for conservation actions: The species is protected by regional law (RAA 2012). Its habitat is located in a regionally protected area (Natural Park of Flores Island). Therefore, degraded habitats in the Natural Park of Flores Island should be restored and a strategy needs to be developed to address the future threat by invasive species and climate change in this area. Ultimately, more area of pristine *Juniperus brevifolia* woodland is required around the lower-altitude, sheltered parts of the cloud forest zone, with greater connectivity between them. Formal education and awareness is needed to allow future investments in restored habitats invaded by invasive plants.

##### Conservation actions

Conservation action type: In Place

Conservation actions: 1. Land/water protection1.1. Land/water protection - Site/area protection5. Law & policy5.1. Law & policy - Legislation5.2. Law & policy - Policies and regulations

##### Conservation actions

Conservation action type: Needed

Conservation actions: 1.1. Land/water protection - Site/area protection2.1. Land/water management - Site/area management2.2. Land/water management - Invasive/problematic species control2.3. Land/water management - Habitat & natural process restoration4. Education & awareness5.4.3. Law & policy - Compliance and enforcement - Sub-national level

#### Conservation actions

Conservation action type: In Place

Conservation actions: 1. Land/water protection1.1. Land/water protection - Site/area protection5. Law & policy5.1. Law & policy - Legislation5.2. Law & policy - Policies and regulations

#### Conservation actions

Conservation action type: Needed

Conservation actions: 1.1. Land/water protection - Site/area protection2.1. Land/water management - Site/area management2.2. Land/water management - Invasive/problematic species control2.3. Land/water management - Habitat & natural process restoration4. Education & awareness5.4.3. Law & policy - Compliance and enforcement - Sub-national level

#### Other

##### Use and trade

Use type: International

##### Ecosystem services

Ecosystem service type: Less important

Ecosystem services: 12. Biocontrol

##### Research needed

Research needed: 1.2. Research - Population size, distribution & trends1.3. Research - Life history & ecology2.2. Conservation Planning - Area-based Management Plan3.1. Monitoring - Population trends3.4. Monitoring - Habitat trends

Justification for research needed: Further research is needed into its ecology and life history in order to find extant specimens and obtain information on population size, distribution and trends. It is also necessary an area-based management plan and a monitoring plan for the invertebrate community in the habitat in order to contribute to perform a species potential recovery plan. Monitoring every ten years using the BALA protocol will inform about habitat quality (see e.g. Gaspar et al. 2011).

#### Use and trade

Use type: International

#### Ecosystem services

Ecosystem service type: Less important

Ecosystem services: 12. Biocontrol

#### Research needed

Research needed: 1.2. Research - Population size, distribution & trends1.3. Research - Life history & ecology2.2. Conservation Planning - Area-based Management Plan3.1. Monitoring - Population trends3.4. Monitoring - Habitat trends

Justification for research needed: Further research is needed into its ecology and life history in order to find extant specimens and obtain information on population size, distribution and trends. It is also necessary an area-based management plan and a monitoring plan for the invertebrate community in the habitat in order to contribute to perform a species potential recovery plan. Monitoring every ten years using the BALA protocol will inform about habitat quality (see e.g. Gaspar et al. 2011).

#### Viability analysis

Justification for probability: 

### Bradycellus chavesi

#### Species information

Scientific name: Bradycellus chavesi

Species authority: Alluaud, 1919

Common names: Ground beetle (English); Carocho (Portuguese).

Kingdom: Animalia

Phylum: Arthropoda

Class: Insecta

Order: Coleoptera

Family: Carabidae

Taxonomic notes: *Bradycellus chavesi* was described from a single female collected in São Miguel island and deposited in the Museum of Paris. The hind-wings are quite rudimentary (Lindroth 1960).

Region for assessment: Global

#### Editor & Reviewers

##### Reviewers

Reviewers: Anja Danielczak

##### Editor

Editor: Axel Hochkirch

#### Reviewers

Reviewers: Anja Danielczak

#### Editor

Editor: Axel Hochkirch

#### Geographic range

Biogeographic realm: Palearctic

Countries: Portugal

Map of records (Google Earth): Suppl. material 3

Basis of EOO and AOO: Observed

Basis (narrative): The species is considered extinct in the unique historical locality. It had a very small extent of occurrence (EEO = 0-4 km^2^) and area of occupancy (AOO = 0-4 km^2^).

Range description: *Bradycellus chavesi* is a single-island endemic species restricted to S. Miguel (Azores, Portugal) (Borges et al. 2010) that is considered extinct in the Azorean arquipelago (Terzopoulou et al. 2015). Last record is from 1919 with the original species description.

#### New occurrences

#### Extent of occurrence

EOO (km2): 0-4

Trend: Stable

Justification for trend: The species is considered extinct in the historical locality, possibly due to habitat destruction. Not sampled recently despite some intensive field work during the BALA expeditions (Borges et al. 2005; Borges et al. 2016).

Causes ceased?: No

Causes understood?: Yes

Causes reversible?: Unknown

Extreme fluctuations?: Unknown

#### Area of occupancy

Trend: Stable

Justification for trend: The species is considered extinct in the historical locality, possibly due to habitat destruction. Not sampled recently despite some intensive field work.

Causes ceased?: No

Causes understood?: Yes

Causes reversible?: Unknown

Extreme fluctuations?: Unknown

AOO (km2): 0-4

#### Locations

Number of locations: 0-1

Justification for number of locations: The species is potentially extinct due to destruction of the habitat in all its range.

Trend: Stable

Justification for trend: Possibly went extinct more than 10 years ago.

Extreme fluctuations?: Unknown

#### Population

Trend: Stable

Justification for trend: The species is only known from a single subpopulation. According to Terzopoulou et al. 2015 this species was extinct more than ten years ago.

Causes ceased?: No

Causes understood?: Yes

Causes reversible?: Unknown

Extreme fluctuations?: Unknown

#### Subpopulations

Number of subpopulations: 0-1

Trend: Stable

Justification for trend: The species is only known from a single subpopulation. According to Terzopoulou et al. 2015 this species is extinct more than 10 years ago.

Extreme fluctuations?: Unknown

Severe fragmentation?: No

#### Habitat

System: Terrestrial

Habitat specialist: Yes

Habitat (narrative): The species occurred in the native forest of São Miguel Island (Azores), but it is considered extinct. Current altitudinal range is unknown.

Trend in extent, area or quality?: Decline (inferred)

Justification for trend: Since the historical record, the native habitat in the island of São Miguel was greatly reduced to accomodate pastures and *Cryptomeria japonica* plantations (Triantis et al. 2010). In the last ten years invasive plant species are spreading (e.g. *Hedychium gardnerianum*; *Gunnera tinctoria*, *Pittosporum undulatum*) changing the structure of the forest and the cover of bryophytes and ferns in the soil with impacts on soil invertebrates.

##### Habitat

Habitat importance: Major Importance

Habitats: 1.4. Forest - Temperate

#### Habitat

Habitat importance: Major Importance

Habitats: 1.4. Forest - Temperate

#### Ecology

Size: 0.5 cm

Generation length (yr): 1

Dependency of single sp?: No

Ecology and traits (narrative): This is an univoltine species. The species was a predator with night activity that lived in hyper-humid forest floor.

#### Threats

Justification for threats: In the past, the species has probably strongly declined due to changes in habitat size. In the last ten years and currently, *Cryptomeria japonica* wood & pulp plantations management and invasive plant species spreading (e.g. *Hedychium gardnerianum*; *Gunnera tinctoria*, *Pittosporum undulatum*) are changing the structure of the forest and the cover of bryophytes and ferns in the soil with impacts on soil invertebrates. Based on Ferreira et al. 2016 the habitat will further decline as a consequence of climate change (increasing number of droughts and habitat shifting & alteration).

##### Threats

Threat type: Ongoing

Threats: 2.2. Agriculture & aquaculture - Wood & pulp plantations8.1.2. Invasive and other problematic species, genes & diseases - Invasive non-native/alien species/diseases - Named species

##### Threats

Threat type: Future

Threats: 8.1. Invasive and other problematic species, genes & diseases - Invasive non-native/alien species/diseases11.1. Climate change & severe weather - Habitat shifting & alteration11.2. Climate change & severe weather - Droughts

#### Threats

Threat type: Ongoing

Threats: 2.2. Agriculture & aquaculture - Wood & pulp plantations8.1.2. Invasive and other problematic species, genes & diseases - Invasive non-native/alien species/diseases - Named species

#### Threats

Threat type: Future

Threats: 8.1. Invasive and other problematic species, genes & diseases - Invasive non-native/alien species/diseases11.1. Climate change & severe weather - Habitat shifting & alteration11.2. Climate change & severe weather - Droughts

#### Conservation

Justification for conservation actions: The species is not protected by regional law. Its habitat is possibly in a regionally protected area (Natural Park of São Miguel Island). There is the need to keep the control of invasive plants and the restoration of native habitat in some areas in the Northeast part of S. Miguel. Formal education and awareness is needed to allow future investments in restored habitats invaded by invasive plants.

##### Conservation actions

Conservation action type: In Place

Conservation actions: 1.1. Land/water protection - Site/area protection

##### Conservation actions

Conservation action type: Needed

Conservation actions: 2.1. Land/water management - Site/area management2.2. Land/water management - Invasive/problematic species control2.3. Land/water management - Habitat & natural process restoration4. Education & awareness5.4.3. Law & policy - Compliance and enforcement - Sub-national level

#### Conservation actions

Conservation action type: In Place

Conservation actions: 1.1. Land/water protection - Site/area protection

#### Conservation actions

Conservation action type: Needed

Conservation actions: 2.1. Land/water management - Site/area management2.2. Land/water management - Invasive/problematic species control2.3. Land/water management - Habitat & natural process restoration4. Education & awareness5.4.3. Law & policy - Compliance and enforcement - Sub-national level

#### Other

##### Use and trade

Use type: International

##### Ecosystem services

Ecosystem service type: Less important

Ecosystem services: 12. Biocontrol

##### Research needed

Research needed: 1.2. Research - Population size, distribution & trends1.3. Research - Life history & ecology

Justification for research needed: Further research is needed into its ecology and life history in order to find extant specimens and obtain information on population size, distribution and trends.

#### Use and trade

Use type: International

#### Ecosystem services

Ecosystem service type: Less important

Ecosystem services: 12. Biocontrol

#### Research needed

Research needed: 1.2. Research - Population size, distribution & trends1.3. Research - Life history & ecology

Justification for research needed: Further research is needed into its ecology and life history in order to find extant specimens and obtain information on population size, distribution and trends.

#### Viability analysis

Justification for probability: 

### Calathus carvalhoi

#### Species information

Scientific name: Calathus carvalhoi

Species authority: Serrano & Borges, 1986

Common names: Ground beetle (English); Carocho (Portuguese).

Kingdom: Animalia

Phylum: Arthropoda

Class: Insecta

Order: Coleoptera

Family: Carabidae

Taxonomic notes: *Calathus carvalhoi* was described from five individuals. A female (Holotype) was collected in Terra Chã (Terceira island) from 15.X.1983 to 4.XI.1983 and deposited in the collection of A. Serrano. A male (Allotype) was collected in Terra Chã (Terceira island) on the 9.X.1983 and deposited in the collection of P. Borges. A male and two females (Paratypes) were collected in Terra Chã (Terceira island) on the 1.X.1984 and on the 9.X.1983, respectively, deposited in the collections of P. Borges and A. Serrano. According to morphology of aedeagus, *Calathus mollis* Marsh, is the closted species to *C. carvalhoi* Serrano & Borges, but according to the index, length:width of the pronotum, this species is more similar to *Calathus lundbladi* Colas (Serrano and Borges 1986).

**Figure 1.**
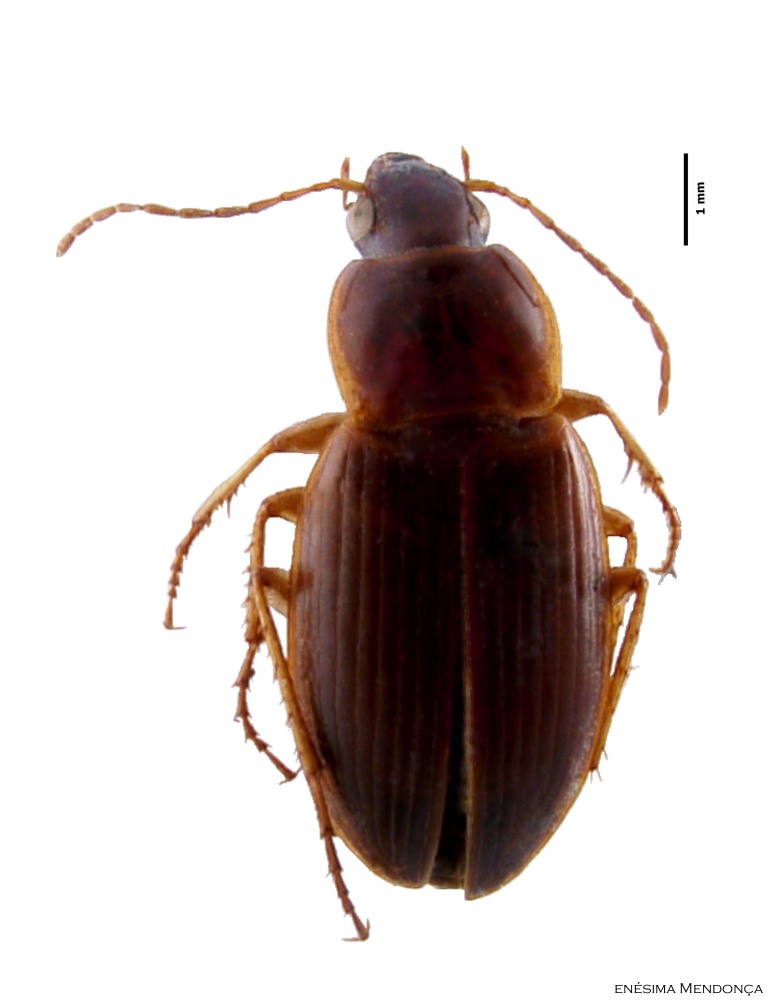
Female of *Calathus carvalhoi* from Terra Chã (Terceira, Azores) (Credit: Enésima Mendonça).

Figure(s) or Photo(s): Fig. 1

Region for assessment: Global

#### Editor & Reviewers

##### Reviewers

Reviewers: Anja Danielczak

##### Editor

Editor: Axel Hochkirch

#### Reviewers

Reviewers: Anja Danielczak

#### Editor

Editor: Axel Hochkirch

#### Geographic range

Biogeographic realm: Palearctic

Countries: Portugal

Map of records (Google Earth): Suppl. material 4

Basis of EOO and AOO: Observed

Basis (narrative): It has a very small extent of occurrence (EOO = 4-8 km²) and area of occupancy (AOO = 4-8 km²).

Min Elevation/Depth (m): 634

Max Elevation/Depth (m): 710

Range description: *Calathus carvalhoi* is a single-island endemic species restricted to Terceira (Azores, Portugal) (Borges et al. 2010), known from the Natural Reserve of Terra Brava. The species is possibly considered extinct in the original locality (Terra Chã). The extent of occurrence (EOO) is 4-8 km^2^ and the maximum area of occupancy (AOO) is also 4-8 km^2^.

#### New occurrences

#### Extent of occurrence

EOO (km2): 4-8

Trend: Stable

Justification for trend: The species is considered extinct in the historical locality (Terra Chã, Terceira island) due to habitat destruction. However, the species was found recently in Terra Brava pristine forest.

Causes ceased?: No

Causes understood?: Yes

Causes reversible?: Unknown

Extreme fluctuations?: Unknown

#### Area of occupancy

Trend: Stable

Justification for trend: The species is considered extinct in the historical locality (Terra Chã, Terceira island) due to habitat destruction. However, the species was found recently in Terra Brava pristine forest.

Causes ceased?: No

Causes understood?: Yes

Causes reversible?: Unknown

Extreme fluctuations?: Unknown

AOO (km2): 4-8

#### Locations

Number of locations: 1

Justification for number of locations: In the historical site (Terra Chã, Terceira island) the species is considered as possibly extinct. Only known with a stable subpopulation in Terra Brava pristine native forest fragment.

Trend: Stable

Justification for trend: Only one site left at Terra Brava (Terceira) that is in pristine native forest.

Extreme fluctuations?: No

#### Population

Trend: Decline (inferred)

Justification for trend: The species is very rare and only known from a single subpopulation in Terra Brava. Despite the fact that Terra Brava was considerered a native forest fragment with a high value of biotic integrity (Gaspar et al. 2011), a continuing decline in the number of mature individuals is inferred from the ongoing recent habitat degradation due to invasions of alien plants that are changing the habitat structure, namely decreasing the cover of bryophytes and ferns in the soil and promoting the spread of other plants with impacts in soil invertebrates.

Basis for decline: (c) a decline in area of occupancy, extent of occurrence and/or quality of habitat

Causes ceased?: No

Causes understood?: Yes

Causes reversible?: Unknown

Extreme fluctuations?: Unknown

#### Subpopulations

Number of subpopulations: 1

Trend: Decline (inferred)

Justification for trend: The species is very rare and only known from a single subpopulation at Terra Brava, since the other subpopulation in Terra Chã is considered as possible extinct. A continuing decline in the number of subpopulations is consequently inferred.

Extreme fluctuations?: Unknown

Severe fragmentation?: No

Justification for fragmentation: 

#### Habitat

System: Terrestrial

Habitat specialist: Yes

Habitat (narrative): The species occurs in a native forests of the Azores (Terceira Island) dominated by Ilex perado ssp. azorica, *Laurus azorica* and *Juniperus brevifolia*, with an altitudinal range between 634 and 710 m.

Trend in extent, area or quality?: Decline (observed)

Justification for trend: Ongoing invasion of exotic plants that are changing the habitat structure, namely decreasing the cover of bryophytes and ferns in the soil and promoting the spread of other plants.

##### Habitat

Habitat importance: Major Importance

Habitats: 1.4. Forest - Temperate

#### Habitat

Habitat importance: Major Importance

Habitats: 1.4. Forest - Temperate

#### Ecology

Size: 0.74 cm

Generation length (yr): 1

Dependency of single sp?: No

Ecology and traits (narrative): This is an univoltine species. It is a night activity predator that lives under barks of native trees and in the soil.

#### Threats

Justification for threats: In the past, the species has probably strongly declined due to deforestation. The species is considered extinct in Terra Chã (Terceira island) due to major historical land-use changes with clearing of original habitat and current *Eucalyptus* spp. wood & pulp plantations management. The most important ongoing threat to this species is the spread of invasive plants (e.g. *Hedychium gardnerianum*) that are changing the habitat structure, namely decreasing the cover of bryophytes and ferns in the soil and promoting the spread of other plants. Based on Ferreira et al. 2016 the habitat will decline as a consequence of climate change (increasing number of droughts, and habitat shift and alteration), which may drive this species to extinction, because it is depending on humid forests.

##### Threats

Threat type: Ongoing

Threats: 2.2.1. Agriculture & aquaculture - Wood & pulp plantations - Small-holder plantations8.1.2. Invasive and other problematic species, genes & diseases - Invasive non-native/alien species/diseases - Named species

##### Threats

Threat type: Future

Threats: 11.1. Climate change & severe weather - Habitat shifting & alteration11.2. Climate change & severe weather - Droughts

#### Threats

Threat type: Ongoing

Threats: 2.2.1. Agriculture & aquaculture - Wood & pulp plantations - Small-holder plantations8.1.2. Invasive and other problematic species, genes & diseases - Invasive non-native/alien species/diseases - Named species

#### Threats

Threat type: Future

Threats: 11.1. Climate change & severe weather - Habitat shifting & alteration11.2. Climate change & severe weather - Droughts

#### Conservation

Justification for conservation actions: The species is protected by regional law (RAA 2012). Its habitat is in a regionally protected area (Natural Park of Terceira). The Terceira Natural Park administration is currently starting control measures of the invasive plants. Further spread of invasive plants needs to be stopped in order to avoid any future declines of the species. Degraded habitats should be restored and a strategy needs to be developed to address the future threat by climate change. A habitat management plan is needed and anticipated to be developed during the coming years. Since this species is restricted to the relict native Azorean forests, it is suggested that some awareness measures should be put in practice.

##### Conservation actions

Conservation action type: In Place

Conservation actions: 1. Land/water protection1.1. Land/water protection - Site/area protection1.2. Land/water protection - Resource & habitat protection5.1. Law & policy - Legislation5.2. Law & policy - Policies and regulations

##### Conservation actions

Conservation action type: Needed

Conservation actions: 2.1. Land/water management - Site/area management2.2. Land/water management - Invasive/problematic species control2.3. Land/water management - Habitat & natural process restoration4. Education & awareness5.4.3. Law & policy - Compliance and enforcement - Sub-national level

#### Conservation actions

Conservation action type: In Place

Conservation actions: 1. Land/water protection1.1. Land/water protection - Site/area protection1.2. Land/water protection - Resource & habitat protection5.1. Law & policy - Legislation5.2. Law & policy - Policies and regulations

#### Conservation actions

Conservation action type: Needed

Conservation actions: 2.1. Land/water management - Site/area management2.2. Land/water management - Invasive/problematic species control2.3. Land/water management - Habitat & natural process restoration4. Education & awareness5.4.3. Law & policy - Compliance and enforcement - Sub-national level

#### Other

##### Use and trade

Use type: International

##### Ecosystem services

Ecosystem service type: Less important

Ecosystem services: 12. Biocontrol

##### Research needed

Research needed: 1.2. Research - Population size, distribution & trends1.3. Research - Life history & ecology2.2. Conservation Planning - Area-based Management Plan3.1. Monitoring - Population trends3.4. Monitoring - Habitat trends

Justification for research needed: Research is needed into its ecology and life history in order to learn about its current population size, distribution and trends. A general monitoring scheme for the invertebrate community in the habitat is in place, but the extant subpopulation of this particular species and its habitat in Terra Brava needs to be monitored in more detail. It is also necessary an area-based management plan for the species in Terra Brava. A monitoring every ten years using the BALA protocol will inform about habitat quality (Gaspar et al. 2011). Based on Borges et al. 2016and Borges et al. 2017b the species is very rare and there is the need to invest in direct nocturnal surveys to evaluate the rarity status of the species.

#### Use and trade

Use type: International

#### Ecosystem services

Ecosystem service type: Less important

Ecosystem services: 12. Biocontrol

#### Research needed

Research needed: 1.2. Research - Population size, distribution & trends1.3. Research - Life history & ecology2.2. Conservation Planning - Area-based Management Plan3.1. Monitoring - Population trends3.4. Monitoring - Habitat trends

Justification for research needed: Research is needed into its ecology and life history in order to learn about its current population size, distribution and trends. A general monitoring scheme for the invertebrate community in the habitat is in place, but the extant subpopulation of this particular species and its habitat in Terra Brava needs to be monitored in more detail. It is also necessary an area-based management plan for the species in Terra Brava. A monitoring every ten years using the BALA protocol will inform about habitat quality (Gaspar et al. 2011). Based on Borges et al. 2016and Borges et al. 2017b the species is very rare and there is the need to invest in direct nocturnal surveys to evaluate the rarity status of the species.

#### Viability analysis

Justification for probability: 

### Calathus extensicollis

#### Species information

Scientific name: Calathus extensicollis

Species authority: Putzeys, 1983

Synonyms: *Calathus mollis* Marsh

Common names: Ground beetle (English); Carocho (Portuguese).

Kingdom: Animalia

Phylum: Arthropoda

Class: Insecta

Order: Coleoptera

Family: Carabidae

Taxonomic notes: *Calathus extensicollis* was described from a single mature female collected in Pico island and deposited in the collection of Chaudoir. Two additional specimens are deposited in British Natural History Museum. The hind-wings are quite reduced (Lindroth 1960).

Region for assessment: Global

#### Editor & Reviewers

##### Reviewers

Reviewers: Anja Danielczak

##### Editor

Editor: Axel Hochkirch

#### Reviewers

Reviewers: Anja Danielczak

#### Editor

Editor: Axel Hochkirch

#### Geographic range

Biogeographic realm: Palearctic

Countries: Portugal

Map of records (Google Earth): Suppl. material 5

Basis of EOO and AOO: Observed

Basis (narrative): It has a very small extent of occurrence (EOO = 0-16 km²) and area of occupancy (EOO = 0-16 km²)

Min Elevation/Depth (m): 900

Max Elevation/Depth (m): 1000

Range description: *Calathus extensicollis* is a single-island endemic species restricted to Pico (Azores, Portugal) (Borges et al. 2010), known from high elevation native forest (900-1000 m). This large bodied species is considered extinct (Terzopoulou et al. 2015). No precise locality was indicated in the original description, but at the indicated elevation there are the current main remnants of native forest.

#### New occurrences

#### Extent of occurrence

EOO (km2): 0-16

Trend: Stable

Justification for trend: The species is considered extinct in the historical locality due to habitat destruction. Not sampled recently despite some intensive field work during the BALA expeditions (Borges et al. 2005; Borges et al. 2016).

Causes ceased?: No

Causes understood?: Yes

Causes reversible?: Unknown

Extreme fluctuations?: Unknown

#### Area of occupancy

Trend: Stable

Justification for trend: The species is considered extinct in the historical locality due to habitat destruction. Not sampled recently despite some intensive field work during the BALA expeditions (Borges et al. 2005; Borges et al. 2016).

Causes ceased?: No

Causes understood?: Yes

Causes reversible?: Unknown

Extreme fluctuations?: Unknown

AOO (km2): 0-16

#### Locations

Number of locations: 0-1

Justification for number of locations: The species is only known from a single subpopulation. According to Terzopoulou et al. 2015 this species is extinct more than 10 years ago. The historical site at 900 m of altitude is the main current area with fragments of native forest.

Trend: Stable

Justification for trend: Possibly went extinct more than 10 years ago.

Extreme fluctuations?: No

#### Population

Trend: Stable

Justification for trend: The species is only known from a single subpopulation. According to Terzopoulou et al. 2015 this species was extinct more than ten years ago.

Causes ceased?: No

Causes understood?: Yes

Causes reversible?: Unknown

Extreme fluctuations?: Unknown

#### Subpopulations

Number of subpopulations: 0-1

Trend: Stable

Justification for trend: The species is only known from a single subpopulation. According to Terzopoulou et al. 2015 this species was extinct more than 10 years ago.

Extreme fluctuations?: Unknown

Severe fragmentation?: No

#### Habitat

System: Terrestrial

Habitat specialist: Yes

Habitat (narrative): The species occurred in the native forest of the Pico Island (Azores), with an altitudinal range between 900 and 1000 m. This species is considered extinct (Terzopoulou et al. 2015) more than 10 years ago.

Trend in extent, area or quality?: Decline (inferred)

Justification for trend: Since the historical record, the native habitat in the island of Pico was greatly reduced to accomodate pastures. Recently invasive plant species are promoting changes in habitat structure.

##### Habitat

Habitat importance: Major Importance

Habitats: 1.4. Forest - Temperate

#### Habitat

Habitat importance: Major Importance

Habitats: 1.4. Forest - Temperate

#### Ecology

Size: 0.90 cm

Generation length (yr): 1

Dependency of single sp?: No

Ecology and traits (narrative): This is an univoltine species. It was a night activity predator that lived in the high elevation native forest.

#### Threats

Justification for threats: In the past, the species has probably strongly declined due to changes in habitat size and quality and the lack of resources due to its large body size (Terzopoulou et al. 2015). Currently, *Cryptomeria japonica* wood & pulp plantations management and invasive plant species spreading (e.g. *Hedychium gardnerianum*; *Pittosporum*) are changing the structure of the forest and the cover of bryophytes and ferns in the soil with impacts on native invertebrate species. Based on Ferreira et al. 2016 the habitat will further decline as a consequence of climate change (increasing number of droughts and habitat shifting & alteration).

##### Threats

Threat type: Ongoing

Threats: 2.2. Agriculture & aquaculture - Wood & pulp plantations8.1.2. Invasive and other problematic species, genes & diseases - Invasive non-native/alien species/diseases - Named species

##### Threats

Threat type: Future

Threats: 11.1. Climate change & severe weather - Habitat shifting & alteration11.2. Climate change & severe weather - Droughts

#### Threats

Threat type: Ongoing

Threats: 2.2. Agriculture & aquaculture - Wood & pulp plantations8.1.2. Invasive and other problematic species, genes & diseases - Invasive non-native/alien species/diseases - Named species

#### Threats

Threat type: Future

Threats: 11.1. Climate change & severe weather - Habitat shifting & alteration11.2. Climate change & severe weather - Droughts

#### Conservation

Justification for conservation actions: The species is not protected by regional law. Its habitat is in a regionally protected area (Natural Park of Pico). It is also necessary a monitoring plan for the invertebrate community in the habitat in order to contribute to perform a species potential recovery plan. Therefore, degraded habitats in the Natural Park of Pico Island should be restored and a strategy needs to be developed to address the future threat by invasive species and climate change in this area. Formal education and awareness is needed to allow future investments in restored habitats invaded by invasive plants.

##### Conservation actions

Conservation action type: In Place

Conservation actions: 1.1. Land/water protection - Site/area protection1.2. Land/water protection - Resource & habitat protection

##### Conservation actions

Conservation action type: Needed

Conservation actions: 2.1. Land/water management - Site/area management2.2. Land/water management - Invasive/problematic species control2.3. Land/water management - Habitat & natural process restoration4. Education & awareness5.4.3. Law & policy - Compliance and enforcement - Sub-national level

#### Conservation actions

Conservation action type: In Place

Conservation actions: 1.1. Land/water protection - Site/area protection1.2. Land/water protection - Resource & habitat protection

#### Conservation actions

Conservation action type: Needed

Conservation actions: 2.1. Land/water management - Site/area management2.2. Land/water management - Invasive/problematic species control2.3. Land/water management - Habitat & natural process restoration4. Education & awareness5.4.3. Law & policy - Compliance and enforcement - Sub-national level

#### Other

##### Use and trade

Use type: International

##### Ecosystem services

Ecosystem service type: Less important

Ecosystem services: 12. Biocontrol

##### Research needed

Research needed: 1.2. Research - Population size, distribution & trends1.3. Research - Life history & ecology3.1. Monitoring - Population trends3.4. Monitoring - Habitat trends

Justification for research needed: Further research is needed into its ecology and life history in order to find extant specimens and obtain information on population size, distribution and trends. It is also necessary a monitoring plan for the invertebrate community in the habitat in order to contribute to perform a species potential recovery plan. Monitoring every ten years using the BALA protocol will inform about habitat quality (see e.g. Gaspar et al. 2011).

#### Use and trade

Use type: International

#### Ecosystem services

Ecosystem service type: Less important

Ecosystem services: 12. Biocontrol

#### Research needed

Research needed: 1.2. Research - Population size, distribution & trends1.3. Research - Life history & ecology3.1. Monitoring - Population trends3.4. Monitoring - Habitat trends

Justification for research needed: Further research is needed into its ecology and life history in order to find extant specimens and obtain information on population size, distribution and trends. It is also necessary a monitoring plan for the invertebrate community in the habitat in order to contribute to perform a species potential recovery plan. Monitoring every ten years using the BALA protocol will inform about habitat quality (see e.g. Gaspar et al. 2011).

#### Viability analysis

Justification for probability: 

### Calathus lundbladi

#### Species information

Scientific name: Calathus lundbladi

Species authority: Colas, 1938

Synonyms: *Calathus melanocephalus* Uyttenboogaart, 1947; *Calathus mollis* Drouet, 1859

Common names: Ground beetle (English); Carocho (Portuguese).

Kingdom: Animalia

Phylum: Arthropoda

Class: Insecta

Order: Coleoptera

Family: Carabida

Taxonomic notes: This is the most similar Azorean endemic *Calathus* species to the mainland relative *Calathus mollis* (Lindroth 1960). The hind-wings are quite reduced (Lindroth 1960).

**Figure 2.**
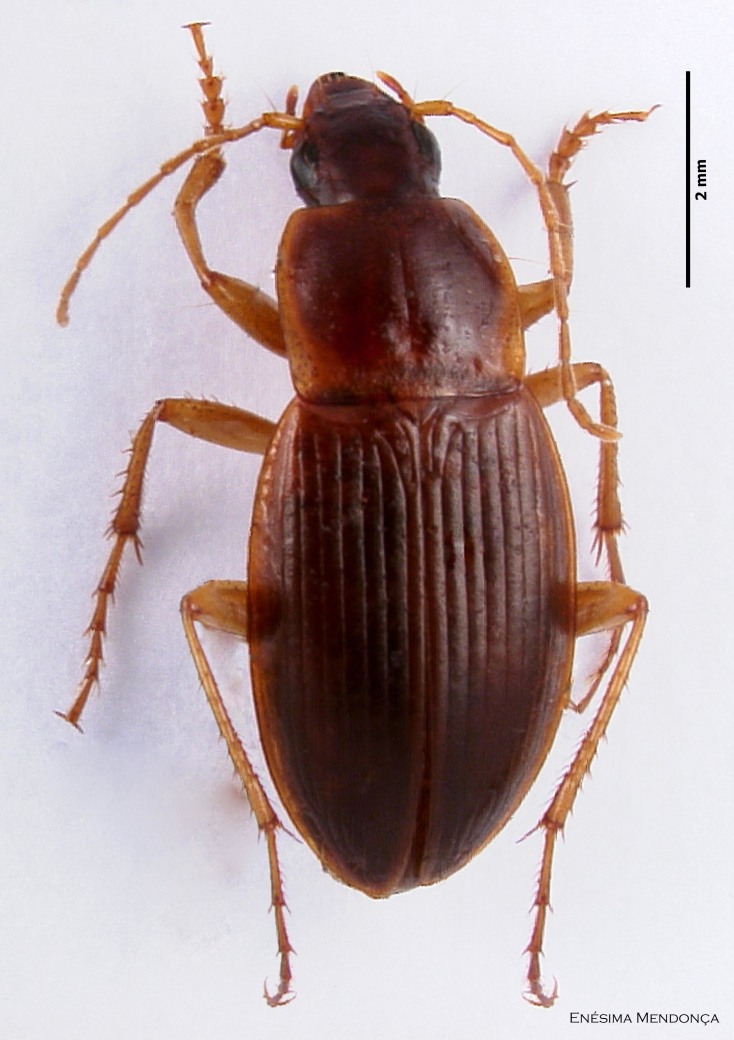
Male of *Calathus lundbladi* from Tronqueira (S. Miguel, Azores) (Credit: Enésima Mendonça).

Figure(s) or Photo(s): Fig. 2

Region for assessment: Global

#### Editor & Reviewers

##### Reviewers

Reviewers: Anja Danielczak

##### Editor

Editor: Axel Hochkirch

#### Reviewers

Reviewers: Anja Danielczak

#### Editor

Editor: Axel Hochkirch

#### Geographic range

Biogeographic realm: Palearctic

Countries: Portugal

Map of records (Google Earth): Suppl. material 6

Basis of EOO and AOO: Observed

Basis (narrative): The extent of occurrence (EOO) is 42.5 km^2^ and the maximum area of occupancy (AOO) is 36.0 km^2^.

Min Elevation/Depth (m): 543

Max Elevation/Depth (m): 100

Range description: *Calathus lundbladi* is an endemic species from S. Miguel (Azores, Portugal) (Borges et al. 2010), known from Natural Forest Reserve of Pico da Vara (Tronqueira). The extent of occurrence and the area of occupancy (AOO) are probably smaller since the species is possibly considered extinct in Furnas.

#### New occurrences

#### Extent of occurrence

EOO (km2): 42.5

Trend: Decline (observed)

Justification for trend: The species occurs in a small fragment of native forest (Tronqueira). This is the only locality known after intensive survey in the Island (Borges et al. 2005, Borges et al. 2016). The EOO of native forest is only around 4 km^2^.

Causes ceased?: No

Causes understood?: Yes

Causes reversible?: Yes

Extreme fluctuations?: Unknown

#### Area of occupancy

Trend: Decline (inferred)

Justification for trend: The species occurs in a small fragment of native forest (Tronqueira). This is the only localityn known after intensive survey in the Island (Borges et al. 2005, Borges et al. 2016). The AOO of native forest is only around 4 km^2^.

Causes ceased?: No

Causes understood?: Yes

Causes reversible?: Yes

Extreme fluctuations?: Unknown

AOO (km2): 36

#### Locations

Number of locations: 1

Justification for number of locations: The complete forest is threatened by invasive plants (*Hedychium gardnerianum* and *Clethra arborea*) that are changing the habitat structure, namely decreasing the cover of bryophytes and ferns in the soil and promoting the spread of other plants.

Trend: Decline (inferred)

Justification for trend: Only one location left, since the species seems extinct in all other sites of the island due to major historical land-use changes. The current subpopulation is threatened by invasive plants that are changing habitat structure.

Extreme fluctuations?: No

#### Population

Trend: Decline (observed)

Justification for trend: A continuing decline in the number of mature individuals is inferred from monitoring schemes (sampled in 1989 with a large population and decreasing numbers in 2000 and 2010) (Borges et al. 2005, Borges et al. 2016), and from the ongoing habitat degradation due to invasions of alien plants (*Hedychium gardnerianum* and *Clethra arborea*).

Basis for decline: (c) a decline in area of occupancy, extent of occurrence and/or quality of habitat

Causes ceased?: No

Causes understood?: Yes

Causes reversible?: Yes

Extreme fluctuations?: Unknown

#### Subpopulations

Number of subpopulations: 1

Trend: Stable

Justification for trend: The species is very rare and only known from a single subpopulation.

Extreme fluctuations?: Unknown

Severe fragmentation?: No

Justification for fragmentation: 

#### Habitat

System: Terrestrial

Habitat specialist: Yes

Habitat (narrative): The species occurs in the hyper-humid native forests of the Azores, surrounded by plantations of exotic trees, with an altitudinal range between 543 and 1000 m.

Trend in extent, area or quality?: Decline (inferred)

Justification for trend: Ongoing invasion of exotic plants (*Hedychium gardnerianum* and *Clethra arborea*) that are changing habitat structure.

##### Habitat

Habitat importance: Major Importance

Habitats: 1.4. Forest - Temperate

#### Habitat

Habitat importance: Major Importance

Habitats: 1.4. Forest - Temperate

#### Ecology

Size: 0.9 cm

Generation length (yr): 1

Dependency of single sp?: No

Ecology and traits (narrative): This is an univoltine species. It is a nocturnal predator that lives under barks of native trees and in the soil.

#### Threats

Justification for threats: In the past, the species has probably strongly declined due to deforestation. The species is considered as possibly extinct in Furnas due to major historical land use changes with clearing of original habitat. The most important ongoing threat to this species is the spread of invasive plants (*Hedychium gardnerianum* and *Clethra arborea*) that are changing the habitat structure, namely decreasing the cover of bryophytes and ferns in the soil and promoting the spread of other plants. Based on Ferreira et al. 2016 the habitat will decline as a consequence of climate change (increasing number of droughts and habitat shifting & alteration), which may drive this species to extinction, because it is dependent on humid forests.

##### Threats

Threat type: Ongoing

Threats: 2.2.1. Agriculture & aquaculture - Wood & pulp plantations - Small-holder plantations8.1. Invasive and other problematic species, genes & diseases - Invasive non-native/alien species/diseases

##### Threats

Threat type: Future

Threats: 11.1. Climate change & severe weather - Habitat shifting & alteration11.2. Climate change & severe weather - Droughts

#### Threats

Threat type: Ongoing

Threats: 2.2.1. Agriculture & aquaculture - Wood & pulp plantations - Small-holder plantations8.1. Invasive and other problematic species, genes & diseases - Invasive non-native/alien species/diseases

#### Threats

Threat type: Future

Threats: 11.1. Climate change & severe weather - Habitat shifting & alteration11.2. Climate change & severe weather - Droughts

#### Conservation

Justification for conservation actions: The species is protected by regional law (RAA 2012). Its habitat is in a regionally protected area (S. Miguel Natural Park). The São Miguel Natural Park administration is currently starting control measures of the invasive plants. LIFE PRIOLO project started with a restoration of degraded habitats increasing the area of pristine forest. A general monitoring scheme for the invertebrate community in the habitat is in place, but the subpopulation of this particular species and its habitat needs to be monitored in more detail. A habitat management plan is needed and anticipated to be developed during the coming years.

##### Conservation actions

Conservation action type: In Place

Conservation actions: 1. Land/water protection1.1. Land/water protection - Site/area protection1.2. Land/water protection - Resource & habitat protection5.1. Law & policy - Legislation5.2. Law & policy - Policies and regulations

##### Conservation actions

Conservation action type: Needed

Conservation actions: 2.1. Land/water management - Site/area management2.2. Land/water management - Invasive/problematic species control2.3. Land/water management - Habitat & natural process restoration4. Education & awareness5.4.3. Law & policy - Compliance and enforcement - Sub-national level

#### Conservation actions

Conservation action type: In Place

Conservation actions: 1. Land/water protection1.1. Land/water protection - Site/area protection1.2. Land/water protection - Resource & habitat protection5.1. Law & policy - Legislation5.2. Law & policy - Policies and regulations

#### Conservation actions

Conservation action type: Needed

Conservation actions: 2.1. Land/water management - Site/area management2.2. Land/water management - Invasive/problematic species control2.3. Land/water management - Habitat & natural process restoration4. Education & awareness5.4.3. Law & policy - Compliance and enforcement - Sub-national level

#### Other

##### Use and trade

Use type: International

##### Ecosystem services

Ecosystem service type: Less important

Ecosystem services: 12. Biocontrol

##### Research needed

Research needed: 1.2. Research - Population size, distribution & trends1.3. Research - Life history & ecology2. Conservation Planning2.2. Conservation Planning - Area-based Management Plan3.1. Monitoring - Population trends3.4. Monitoring - Habitat trends

Justification for research needed: Further research is needed into its ecology and life history in order to obtain information on population size, distribution and trends. A general monitoring scheme for the invertebrate community in the habitat is in place, but the subpopulation of this particular species and its habitat needs to be monitored in more detail in order to contribute to perform an area-based management plan and a species potential recovery plan due to recent rarity. Monitoring every ten years using the BALA protocol will inform about habitat quality (see e.g. Gaspar et al. 2011).

#### Use and trade

Use type: International

#### Ecosystem services

Ecosystem service type: Less important

Ecosystem services: 12. Biocontrol

#### Research needed

Research needed: 1.2. Research - Population size, distribution & trends1.3. Research - Life history & ecology2. Conservation Planning2.2. Conservation Planning - Area-based Management Plan3.1. Monitoring - Population trends3.4. Monitoring - Habitat trends

Justification for research needed: Further research is needed into its ecology and life history in order to obtain information on population size, distribution and trends. A general monitoring scheme for the invertebrate community in the habitat is in place, but the subpopulation of this particular species and its habitat needs to be monitored in more detail in order to contribute to perform an area-based management plan and a species potential recovery plan due to recent rarity. Monitoring every ten years using the BALA protocol will inform about habitat quality (see e.g. Gaspar et al. 2011).

#### Viability analysis

Justification for probability: 

### Calathus vicenteorum

#### Species information

Scientific name: Calathus vicenteorum

Species authority: Schatzmayr, 1939

Common names: Ground beetle (English); Carocho (Portuguese).

Kingdom: Animalia

Phylum: Arthropoda

Class: Insecta

Order: Coleoptera

Family: Carabida

Taxonomic notes: The most similar species is *C. lundbladi* that is endemic to S. Miguel; the eyes are flatter than in *C. mollis* and the hind wings are reduced (Lindroth 1960).

Region for assessment: Global

#### Editor & Reviewers

##### Reviewers

Reviewers: Anja Danielczak

##### Editor

Editor: Axel Hochkirch

#### Reviewers

Reviewers: Anja Danielczak

#### Editor

Editor: Axel Hochkirch

#### Geographic range

Biogeographic realm: Palearctic

Countries: Portugal

Map of records (Google Earth): Suppl. material 7

Basis of EOO and AOO: Observed

Basis (narrative): It has a very small extent of occurrence (EOO = 0-4 km²) and area of occupancy (AOO = 0-4 km²).

Min Elevation/Depth (m): 450

Max Elevation/Depth (m): 550

Range description: *Calathus vicenteorum* is an endemic species from Santa Maria (Azores, Portugal) (Borges et al. 2010), known from high elevation native forest in Pico Alto (550 m asl). This large bodied species is considered extinct (Terzopoulou et al. 2015). The area of its remaining native habitat is 0.09 km².

#### New occurrences

#### Extent of occurrence

EOO (km2): 0-4

Trend: Stable

Justification for trend: The area of its remaining native habitat is now 0.09 km². Not sampled recently despite some intensive field work during the BALA expeditions (Borges et al. 2005; Borges et al. 2016). This large bodied species is considered extinct.

Causes ceased?: No

Causes understood?: Yes

Causes reversible?: Unknown

Extreme fluctuations?: Unknown

#### Area of occupancy

Trend: Stable

Justification for trend: The area of its remaining native habitat is now 0.09 km². Not sampled recently despite some intensive field work during the BALA expeditions (Borges et al. 2005; Borges et al. 2016). This large bodied species is considered extinct.

Causes ceased?: No

Causes understood?: Yes

Causes reversible?: Unknown

Extreme fluctuations?: Unknown

AOO (km2): 0-4

#### Locations

Number of locations: 0-1

Justification for number of locations: A single fragment of native forest at Pico Alto currently with 0.09 km^2^ included in a Natural Reserve that has a very low Index of Biotic Integrity (Gaspar et al. 2011). The species is considered extinct due to destruction of the habitat in all its range.

Trend: Stable

Justification for trend: Possibly went extinct more than 10 years ago.

Extreme fluctuations?: No

#### Population

Trend: Stable

Justification for trend: The species is only known from a single subpopulation. According to Terzopoulou et al. 2015 this species was extinct more than ten years ago.

Causes ceased?: No

Causes understood?: Yes

Causes reversible?: Unknown

Extreme fluctuations?: Unknown

#### Subpopulations

Number of subpopulations: 0-1

Trend: Stable

Justification for trend: The species is only known from a single subpopulation. According to Terzopoulou et al. 2015 this species is extinct more than 10 years ago.

Extreme fluctuations?: Unknown

Severe fragmentation?: No

#### Habitat

System: Terrestrial

Habitat specialist: Yes

Habitat (narrative): The species occurred in the native forest of the Santa Maria Island (Azores), with an altitudinal range between 500 and 550 m. It is considered extinct (Terzopoulou et al. 2015).

Trend in extent, area or quality?: Decline (inferred)

Justification for trend: Since the historical record, the native habitat in the island of Santa Maria was greatly reduced to accomodate *Cryptomeria japonica* plantations (Triantis et al. 2010). Currently invasive plants (*Hedychium gardnerianum*; *Pittosporum undulatum*) are changing the structure of the forest and the cover of bryophytes and ferns in the soil which will impact the species habitat quality.

##### Habitat

Habitat importance: Major Importance

Habitats: 1.4. Forest - Temperate

#### Habitat

Habitat importance: Major Importance

Habitats: 1.4. Forest - Temperate

#### Ecology

Size: 0.87 cm

Generation length (yr): 1

Dependency of single sp?: No

Ecology and traits (narrative): This is a predator species with nocturnal activity. The last specimens found in 1957 were captured associated with *Calluna vulgaris* (Lindroth 1960).

#### Threats

Justification for threats: In the past, the species has probably strongly declined due to changes in habitat size and quality and its large body size (Terzopoulou et al. 2015). Based on Ferreira et al. 2016 the habitat will further decline as a consequence of climate change (increasing number of droughts and habitat shifting & alteration). The most important ongoing threat to this species is *Cryptomeria japonica* wood & pulp plantations management and the spread of invasive plants (*Hedychium gardnerianum* and *Pittosporum undulatum*) that are changing the habitat structure in the main native forest, namely decreasing the cover of bryophytes and ferns in the soil and promoting the spread of other plants.

##### Threats

Threat type: Ongoing

Threats: 2.2.1. Agriculture & aquaculture - Wood & pulp plantations - Small-holder plantations8.1.2. Invasive and other problematic species, genes & diseases - Invasive non-native/alien species/diseases - Named species

##### Threats

Threat type: Future

Threats: 11.1. Climate change & severe weather - Habitat shifting & alteration11.2. Climate change & severe weather - Droughts

#### Threats

Threat type: Ongoing

Threats: 2.2.1. Agriculture & aquaculture - Wood & pulp plantations - Small-holder plantations8.1.2. Invasive and other problematic species, genes & diseases - Invasive non-native/alien species/diseases - Named species

#### Threats

Threat type: Future

Threats: 11.1. Climate change & severe weather - Habitat shifting & alteration11.2. Climate change & severe weather - Droughts

#### Conservation

Justification for conservation actions: The species is not protected by regional law. Its habitat is in a regionally protected area (Natural Park of Santa Maria). It is also necessary a monitoring plan for the invertebrate community in the habitat in order to contribute to perform a species potential recovery plan. Therefore, degraded habitats in the Natural Park of Santa Maria Island should be restored and a strategy needs to be developed to address the future threat by invasive species and climate change in this area. Formal education and awareness is needed to allow future investments in restored habitats invaded by invasive plants.

##### Conservation actions

Conservation action type: In Place

Conservation actions: 1. Land/water protection1.1. Land/water protection - Site/area protection

##### Conservation actions

Conservation action type: Needed

Conservation actions: 2.1. Land/water management - Site/area management2.2. Land/water management - Invasive/problematic species control2.3. Land/water management - Habitat & natural process restoration4. Education & awareness5.4.3. Law & policy - Compliance and enforcement - Sub-national level

#### Conservation actions

Conservation action type: In Place

Conservation actions: 1. Land/water protection1.1. Land/water protection - Site/area protection

#### Conservation actions

Conservation action type: Needed

Conservation actions: 2.1. Land/water management - Site/area management2.2. Land/water management - Invasive/problematic species control2.3. Land/water management - Habitat & natural process restoration4. Education & awareness5.4.3. Law & policy - Compliance and enforcement - Sub-national level

#### Other

##### Use and trade

Use type: International

##### Ecosystem services

Ecosystem service type: Less important

Ecosystem services: 12. Biocontrol

##### Research needed

Research needed: 1.2. Research - Population size, distribution & trends1.3. Research - Life history & ecology2.2. Conservation Planning - Area-based Management Plan3.1. Monitoring - Population trends3.4. Monitoring - Habitat trends

Justification for research needed: Further research is needed into its ecology and life history in order to find extant specimens and obtain information on population size, distribution and trends. It is also necessary an area-based management plan and a monitoring plan for the invertebrate community in the habitat in order to contribute to perform a species potential recovery plan. Monitoring every ten years using the BALA protocol will inform about habitat quality (see e.g. Gaspar et al. 2011).

#### Use and trade

Use type: International

#### Ecosystem services

Ecosystem service type: Less important

Ecosystem services: 12. Biocontrol

#### Research needed

Research needed: 1.2. Research - Population size, distribution & trends1.3. Research - Life history & ecology2.2. Conservation Planning - Area-based Management Plan3.1. Monitoring - Population trends3.4. Monitoring - Habitat trends

Justification for research needed: Further research is needed into its ecology and life history in order to find extant specimens and obtain information on population size, distribution and trends. It is also necessary an area-based management plan and a monitoring plan for the invertebrate community in the habitat in order to contribute to perform a species potential recovery plan. Monitoring every ten years using the BALA protocol will inform about habitat quality (see e.g. Gaspar et al. 2011).

#### Viability analysis

Justification for probability: 

### Cedrorum azoricus

#### Species information

Scientific name: Cedrorum azoricus

Species authority: Borges & Serrano, 1993

Common names: Ground beetle (English); Carocho-da-penumbra (Portuguese)

Kingdom: Animalia

Phylum: Arthropoda

Class: Insecta

Order: Coleoptera

Family: Carabidae

Taxonomic notes: *Cedrorum azoricus* was described from individuals collected in Terceira (Terra Brava and Morro Assombrado), Santa Maria (Pico Alto) and Pico (Caveiro) islands, between 1990 and 1992. This species is recognized by the form of pronotum, the absence of crossed epipleura, the shape of the aedeagus and the shape of the terminal stylomere. *C. azoricus* has two subspecies, recognized by the form of pronotum (Borges and Serrano 1993).

**Figure 3.**
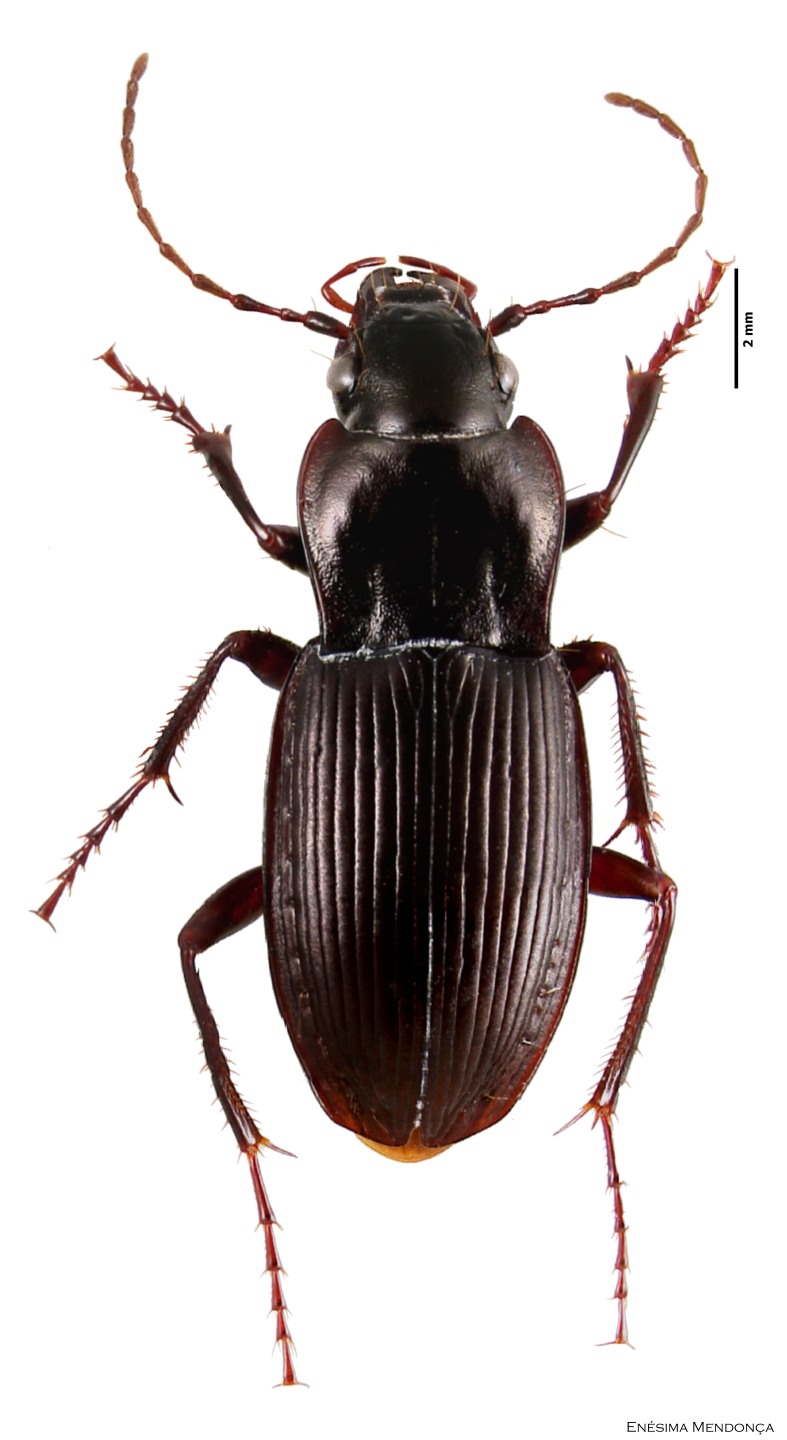
Male of *Cedrorum azoricus azoricus* from Caldeira de St. Bárbara (Terceira, Azores) (Credit: Enésima Mendonça).

**Figure 4.**
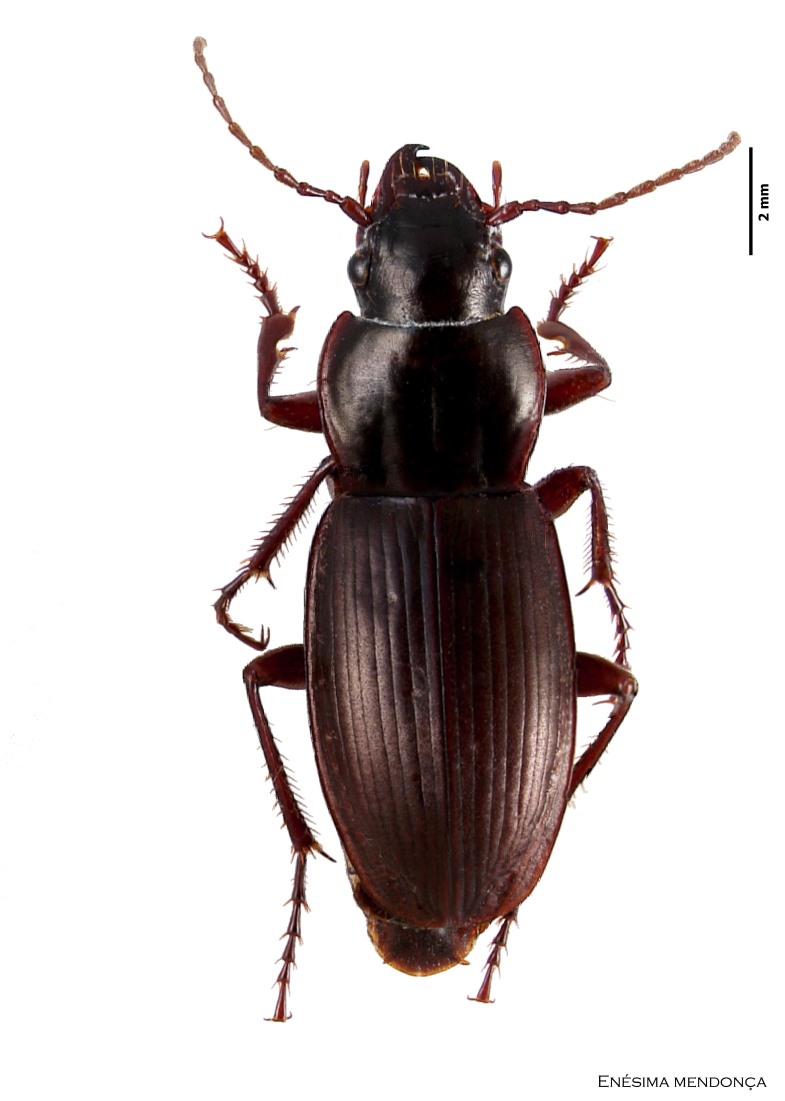
Male of *Cedrorum azoricus caveirensis* from Cabeço do Caveiro (Pico, Azores) (Credit: Enésima Mendonça).

Figure(s) or Photo(s): Fig. 3Fig. 4

Region for assessment: Global

#### Editor & Reviewers

##### Reviewers

Reviewers: Anja Danielczak

##### Editor

Editor: Axel Hochkirch

#### Reviewers

Reviewers: Anja Danielczak

#### Editor

Editor: Axel Hochkirch

#### Geographic range

Biogeographic realm: Palearctic

Countries: Portugal

Map of records (Google Earth): Suppl. material 8

Basis of EOO and AOO: Observed

Basis (narrative): The extent of occurrence (EOO) is ca 12,300 km^2^ and the maximum area of occupancy (AOO) is 40 km^2^.

Min Elevation/Depth (m): 500

Max Elevation/Depth (m): 1200

Range description: *Cedrorum azoricus* is an endemic species with two subspecies, *C. a. azoricus* occurring in Terceira and Santa Maria islands, and *C. a. caveirensis* restricted to Pico island (Azores, Portugal) (Borges and Serrano 1993; Borges et al. 2010). The species is known from the Natural Forest Reserves of Biscoito da Ferraria, Serra de Santa Bárbara, Terra Brava (Terceira), Caveiro and Mistério da Prainha (Pico) and Pico Alto (Santa Maria).

#### New occurrences

#### Extent of occurrence

EOO (km2): 12,300

Trend: Decline (inferred)

Justification for trend: The extent of occurrence includes large areas of unsuitable habitats and the size of its remaining native habitat is now only 40 km^2^. The species continues in decline due to native forest degradation (mainly *Juniperus brevifolia*-*Laurus azorica* and Ilex perado subsp. azorica forests) due to the ongoing spread of invasive species in Santa Maria (*Hedychium gardnerianum* and *Pittosporum undulatum*), Terceira and Pico (*Hedychium gardnerianum*).

Causes ceased?: No

Causes understood?: Yes

Causes reversible?: Unknown

Extreme fluctuations?: Unknown

#### Area of occupancy

Trend: Decline (inferred)

Justification for trend: The species occurs in native forests of several islands (Terceira, Pico and Santa Maria). The AOO with native forest is around 40 km². The species continues in decline due to native forest degradation (mainly *Juniperus brevifolia*, *Laurus azorica* and Ilex perado subsp. azorica forests) due to the ongoing spread of invasive species in Santa Maria (*Hedychium gardnerianum* and *Pittosporum undulatum*), Terceira and Pico (*Hedychium gardnerianum*).

Causes ceased?: No

Causes understood?: Yes

Causes reversible?: Unknown

Extreme fluctuations?: Unknown

AOO (km2): 40

#### Locations

Number of locations: 6

Justification for number of locations: The species inhabits in six native isolated forest patches in three islands (Terceira, Pico, Santa Maria).

Trend: Decline (observed)

Justification for trend: Six locations known since in the last ten years a spread of invasive plants is changing the habitat structure, namely decreasing the cover of bryophytes and ferns in the soil and promoting the spread of other plants. Pico Alto (Santa Maria) site has a very low Index of Biotic Integrity (Gaspar et al. 2011) with a size of 0.09 km² and invasive plants that can drive the local subpopulation to extinction very fast. In recent times at least one location was lost in S. Maria island due to major changes in habitat with the removal of vegetation.

Extreme fluctuations?: Unknown

#### Population

Trend: Decline (inferred)

Justification for trend: The species is particularly restricted and the subpopulation of Santa Maria is very low in number of individuals. A continuing decline in the number of mature individuals is inferred from monitoring schemes (never sampled in Santa Maria after its description, in spite of several sampling efforts in the last ten years) (Borges et al. 2005, Borges et al. 2016), and from the ongoing habitat degradation due to invasions of alien plants (Gaspar et al. 2011).

Basis for decline: (c) a decline in area of occupancy, extent of occurrence and/or quality of habitat

Causes ceased?: No

Causes understood?: Yes

Causes reversible?: Unknown

Extreme fluctuations?: Unknown

#### Subpopulations

Number of subpopulations: 4

Trend: Decline (inferred)

Justification for trend: The species is known from four subpopulations. The subpopulation of Santa Maria is very low in number of individuals. A continuing decline in the number of subpopulations is inferred from monitoring schemes (never sampled in Sta. Maria after its description, in spite of several sampling efforts in the last ten years) (Borges et al. 2005, Borges et al. 2016), and from the ongoing habitat degradation due to invasions of alien plants.

Extreme fluctuations?: Unknown

Severe fragmentation?: Yes

Justification for fragmentation: Major land-use changes at all elevations in Santa Maria, Terceira and Pico islands promoted the creation of small patches of native and exotic forest. The species occurs in four natural forest fragments that are isolated in a sea of pastures and *Cryptomeria japonica* plantations. At least two of the locations will be under severe threat in the next 10 years due to the aggressive spread of the invasive plant *Hedychium gardnerianum*.

#### Habitat

System: Terrestrial

Habitat specialist: Yes

Habitat (narrative): *Cedrorum azoricus* has two subspecies, *C. a. azoricus* present in Terceira and Santa Maria islands, occurs in native forests of high altitude (altitudinal range between 500 and 1000 m) ("cloud-zone forests"; dominated by *Juniperus brevifolia*, Ilex perado subsp. azorica and *Laurus azorica*), and *C. a. caveirensis*, restricted to Pico island, occurs also in native forests (dominated by *Juniperus brevifolia*) (altitudinal range between 800 and 1200 m) (Borges and Serrano 1993; Borges et al. 2010).

Trend in extent, area or quality?: Decline (inferred)

Justification for trend: In the past, the species has probably strongly declined due to changes in habitat size and quality and its large body size (Terzopoulou et al. 2015). Currently invasive plant species are decreasing the quality of the habitat .

##### Habitat

Habitat importance: Major Importance

Habitats: 1.4. Forest - Temperate

#### Habitat

Habitat importance: Major Importance

Habitats: 1.4. Forest - Temperate

#### Ecology

Size: 1.34-1.6 cm

Generation length (yr): 1

Dependency of single sp?: No

Ecology and traits (narrative): This is an univoltine species. It is a nocturnal predator that lives in the soil. In both Terceira and Pico islands it occurs mostly in sites with deep crevices in hyper-humid forest. The peak of activity in October, being an autumn breader.

#### Threats

Justification for threats: In the past, the species has probably strongly declined due to changes in habitat size and quality and its large body size (Terzopoulou et al. 2015). Ongoing invasion of an invasive plant species (*Hedychium gardnerianum*) in Terceira and Pico and in addition *Pittosporum undulatum* in Santa Maria, are major threats since these plant species are changing the habitat structure in the main native forest, namely decreasing the cover of bryophytes and ferns in the soil and promoting the spread of other plants. Based on Ferreira et al. (2016) the habitat will further decline as a consequence of climate change (increasing number of droughts and habitat shifting & alteration).

##### Threats

Threat type: Ongoing

Threats: 2.2.1. Agriculture & aquaculture - Wood & pulp plantations - Small-holder plantations8.1.2. Invasive and other problematic species, genes & diseases - Invasive non-native/alien species/diseases - Named species

##### Threats

Threat type: Future

Threats: 11.1. Climate change & severe weather - Habitat shifting & alteration11.2. Climate change & severe weather - Droughts

#### Threats

Threat type: Ongoing

Threats: 2.2.1. Agriculture & aquaculture - Wood & pulp plantations - Small-holder plantations8.1.2. Invasive and other problematic species, genes & diseases - Invasive non-native/alien species/diseases - Named species

#### Threats

Threat type: Future

Threats: 11.1. Climate change & severe weather - Habitat shifting & alteration11.2. Climate change & severe weather - Droughts

#### Conservation

Justification for conservation actions: The species is protected by regional law (RAA 2012). Its habitat is in regionally protected areas (Natural Parks of Terceira, Pico and Santa Maria). The Terceira Natural Park administration is currently starting control measures of the invasive plants. Further spread of invasive plants needs to be stopped in order to avoid any future declines of the species. Degraded habitats should be restored and a strategy needs to be developed to address the future threat by climate change. It is necessary a monitoring plan for the invertebrate community in the habitat in order to contribute to the conservation of this species. A habitat management plan is needed and anticipated to be developed during the coming years.

##### Conservation actions

Conservation action type: In Place

Conservation actions: 1. Land/water protection1.1. Land/water protection - Site/area protection1.2. Land/water protection - Resource & habitat protection2. Land/water management4. Education & awareness5.1. Law & policy - Legislation5.2. Law & policy - Policies and regulations

##### Conservation actions

Conservation action type: Needed

Conservation actions: 2.1. Land/water management - Site/area management2.2. Land/water management - Invasive/problematic species control2.3. Land/water management - Habitat & natural process restoration4. Education & awareness5.4.3. Law & policy - Compliance and enforcement - Sub-national level

#### Conservation actions

Conservation action type: In Place

Conservation actions: 1. Land/water protection1.1. Land/water protection - Site/area protection1.2. Land/water protection - Resource & habitat protection2. Land/water management4. Education & awareness5.1. Law & policy - Legislation5.2. Law & policy - Policies and regulations

#### Conservation actions

Conservation action type: Needed

Conservation actions: 2.1. Land/water management - Site/area management2.2. Land/water management - Invasive/problematic species control2.3. Land/water management - Habitat & natural process restoration4. Education & awareness5.4.3. Law & policy - Compliance and enforcement - Sub-national level

#### Other

Justification for use and trade: This species is not utilized.

##### Use and trade

Use type: International

##### Ecosystem services

Ecosystem service type: Less important

Ecosystem services: 12. Biocontrol

##### Research needed

Research needed: 1.2. Research - Population size, distribution & trends1.3. Research - Life history & ecology2.2. Conservation Planning - Area-based Management Plan3.1. Monitoring - Population trends3.4. Monitoring - Habitat trends

Justification for research needed: Further research is needed into its ecology and life history in order to find extant specimens and obtain information on population size, distribution and trends. It is also necessary an area-based management plan and a monitoring plan for the invertebrate community in the habitat in order to contribute to perform a species potential recovery plan in the island of Santa Maria. Monitoring every ten years using the BALA protocol will inform about habitat quality (see e.g. Gaspar et al. 2011).

#### Use and trade

Use type: International

#### Ecosystem services

Ecosystem service type: Less important

Ecosystem services: 12. Biocontrol

#### Research needed

Research needed: 1.2. Research - Population size, distribution & trends1.3. Research - Life history & ecology2.2. Conservation Planning - Area-based Management Plan3.1. Monitoring - Population trends3.4. Monitoring - Habitat trends

Justification for research needed: Further research is needed into its ecology and life history in order to find extant specimens and obtain information on population size, distribution and trends. It is also necessary an area-based management plan and a monitoring plan for the invertebrate community in the habitat in order to contribute to perform a species potential recovery plan in the island of Santa Maria. Monitoring every ten years using the BALA protocol will inform about habitat quality (see e.g. Gaspar et al. 2011).

#### Viability analysis

Justification for probability: 

### Olisthopus inclavatus

#### Species information

Scientific name: Olisthopus inclavatus

Species authority: Israelson, 1983

Common names: Ground beetle (English); Carocho (Portuguese)

Kingdom: Animalia

Phylum: Arthropoda

Class: Insecta

Order: Coleoptera

Family: Carabidae

Taxonomic notes: *Olisthopus inclavatus* was described from a single male collected on the 10.VII.1982 in Santa Maria (Airport) island. This species is similar to *O. elongatus* Wollaston but differs by the absence of a conspicuous nail-shaped sclerite in the internal sac of the penis (Israelson 1983).

**Figure 5.**
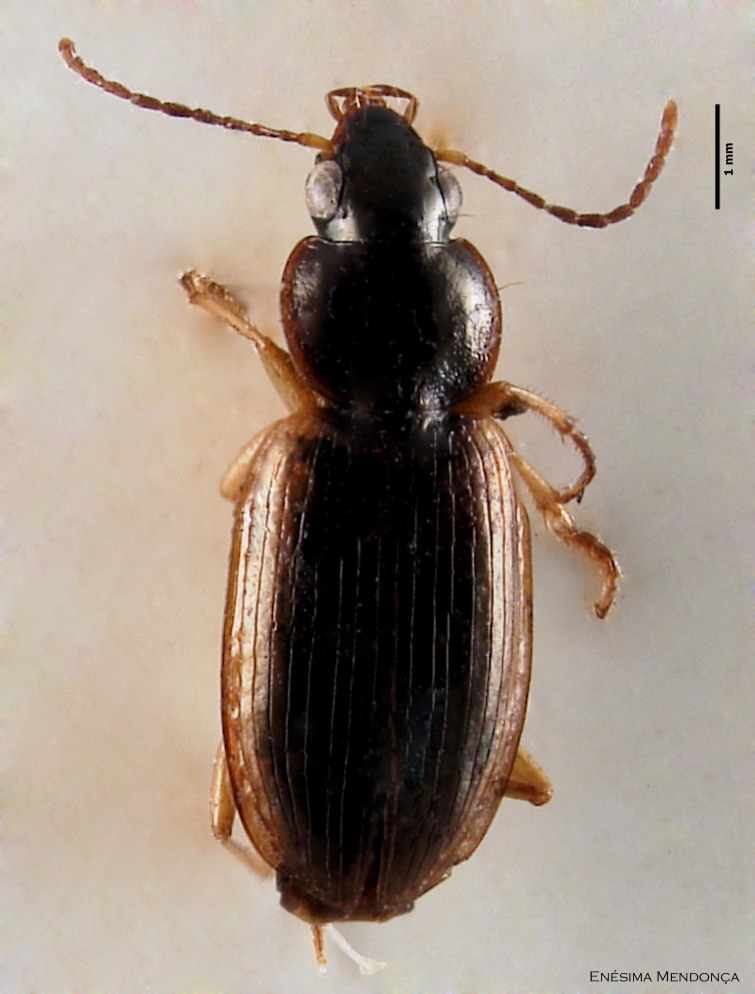
Female of *Olisthopus inclavatus* from Mata dos Anjos (Santa Maria, Azores) (Credit: Enésima Mendonça).

Figure(s) or Photo(s): Fig. 5

Region for assessment: Global

#### Editor & Reviewers

##### Reviewers

Reviewers: Anja Danielczak

##### Editor

Editor: Axel Hochkirch

#### Reviewers

Reviewers: Anja Danielczak

#### Editor

Editor: Axel Hochkirch

#### Geographic range

Biogeographic realm: Palearctic

Countries: Portugal

Map of records (Google Earth): Suppl. material 9

Basis of EOO and AOO: Observed

Basis (narrative): *Olisthopus inclavatus* is a single-island endemic species from Santa Maria (Azores, Portugal) (Borges et al. 2010), known from few patches of highly modified lowland forests.

Min Elevation/Depth (m): 150

Max Elevation/Depth (m): 300

Range description: The extent of occurrence (EOO) is 35 km^2^ and the maximum area of occupancy (AOO) is 32 km^2^.

#### New occurrences

#### Extent of occurrence

EOO (km2): 35

Trend: Decline (inferred)

Justification for trend: The species occurs in several small fragments of exotic forest in a small area at low altitude. The species continues in decline due to native forest destruction and habitat fragmentation.

Causes ceased?: No

Causes understood?: Yes

Causes reversible?: Unknown

Extreme fluctuations?: Unknown

#### Area of occupancy

Trend: Decline (inferred)

Justification for trend: The species occurs in several exotic forest patchs in Santa Maria island and it is relatively widespread by the island, but highly fragmented. The species continues in decline due to native forest destruction, invasive plant spread and habitat fragmentation.

Causes ceased?: No

Causes understood?: Yes

Causes reversible?: Unknown

Extreme fluctuations?: Unknown

AOO (km2): 32

#### Locations

Number of locations: 4

Justification for number of locations: The species occurs in at least four isolated exotic forest patches in Santa Maria island that are under major disturbance.

Trend: Decline (observed)

Justification for trend: Four locations known but the area decreased in the last ten years due to exotic forest removal.

Extreme fluctuations?: No

#### Population

Trend: Decline (inferred)

Justification for trend: The species is particularly restricted but the abundance is relatively high in some of the known sites. A continuing decline in the number of mature individuals is inferred from the ongoing habitat degradation due to invasions of alien plants and the destruction of exotic plantations for the implementation of pastures.

Basis for decline: (c) a decline in area of occupancy, extent of occurrence and/or quality of habitat

Causes ceased?: No

Causes understood?: Yes

Causes reversible?: Unknown

Extreme fluctuations?: Unknown

#### Subpopulations

Number of subpopulations: 5

Trend: Decline (inferred)

Justification for trend: The species is particularly restricted but the abundance is relatively high in some of the known subpopulations. A continuing decline in the number of subpopulations is inferred from the ongoing habitat degradation due to invasions of alien plants and the destruction of exotic plantations for the implementation of pastures.

Extreme fluctuations?: Unknown

Severe fragmentation?: Yes

Justification for fragmentation: Major land-use changes at all elevations in S. Maria island promoted the creation of small patches of native and exotic forest. The species occurs in five small patches of exotic forest that are isolated in a sea of pastures and *Cryptomeria japonica* plantations. More than 50% of the known subpopulations are under severe threat due to exotic forest management and eventual forest removal.

#### Habitat

System: Terrestrial

Habitat specialist: Yes

Habitat (narrative): The species occurs in exotic forests (dominated by *Cryptomeria japonica*, *Acacia* spp.), semi-natural forests and in semi-natural pastures in Santa Maria island, with an altitudinal range between 150 and 300 m, being relatively widespread (Meijer et al. 2011).

Trend in extent, area or quality?: Decline (inferred)

Justification for trend: In the past, the species has probably strongly declined due to changes in habitat size and quality and its large body size (Terzopoulou et al. 2015).

##### Habitat

Habitat importance: Major Importance

Habitats: 1.4. Forest - Temperate14.2. Artificial/Terrestrial - Pastureland16. Introduced vegetation

#### Habitat

Habitat importance: Major Importance

Habitats: 1.4. Forest - Temperate14.2. Artificial/Terrestrial - Pastureland16. Introduced vegetation

#### Ecology

Size: 0.55 cm

Generation length (yr): 1

Dependency of single sp?: No

Ecology and traits (narrative): This is an univoltine species. It is a nocturnal predator that lives in native trees and in the soil associated with plant litter.

#### Threats

Justification for threats: In the past, the species has probably strongly declined due to changes in habitat size and quality. Currently the modified habitats where it occurs are being highly disturbed, namely pactches of *Cryptomeria japonica* and *Acacia* spp.. Agriculture and wood & pulp productions are also a major threat. Based on Ferreira et al. (2016) the habitat will further decline as a consequence of climate change (increasing number of droughts and habitat shifting & alteration).

##### Threats

Threat type: Ongoing

Threats: 2.2.1. Agriculture & aquaculture - Wood & pulp plantations - Small-holder plantations8.1.2. Invasive and other problematic species, genes & diseases - Invasive non-native/alien species/diseases - Named species

##### Threats

Threat type: Future

Threats: 11.1. Climate change & severe weather - Habitat shifting & alteration11.2. Climate change & severe weather - Droughts

#### Threats

Threat type: Ongoing

Threats: 2.2.1. Agriculture & aquaculture - Wood & pulp plantations - Small-holder plantations8.1.2. Invasive and other problematic species, genes & diseases - Invasive non-native/alien species/diseases - Named species

#### Threats

Threat type: Future

Threats: 11.1. Climate change & severe weather - Habitat shifting & alteration11.2. Climate change & severe weather - Droughts

#### Conservation

Justification for conservation actions: The species is not protected by regional law. Degraded habitats should be restored and a strategy needs to be developed to address the ongoing impact of invasive plants and future threat by climate change. It is necessary a monitoring plan for the invertebrate community in the habitat in order to contribute to the conservation of this species. A habitat management plan is needed and anticipated to be developed during the coming years. Formal education and awareness is needed to allow future investments in restored habitats invaded by alien plants.

##### Conservation actions

Conservation action type: Needed

Conservation actions: 2.1. Land/water management - Site/area management2.2. Land/water management - Invasive/problematic species control2.3. Land/water management - Habitat & natural process restoration4. Education & awareness5.4.3. Law & policy - Compliance and enforcement - Sub-national level

#### Conservation actions

Conservation action type: Needed

Conservation actions: 2.1. Land/water management - Site/area management2.2. Land/water management - Invasive/problematic species control2.3. Land/water management - Habitat & natural process restoration4. Education & awareness5.4.3. Law & policy - Compliance and enforcement - Sub-national level

#### Other

Justification for use and trade: This species is not utilized.

##### Use and trade

Use type: International

##### Ecosystem services

Ecosystem service type: Less important

Ecosystem services: 12. Biocontrol

##### Research needed

Research needed: 1.2. Research - Population size, distribution & trends1.3. Research - Life history & ecology3.1. Monitoring - Population trends3.4. Monitoring - Habitat trends

Justification for research needed: Further research is needed into its ecology and life history in order to find extant specimens and obtain information on population size, distribution and trends. It is also necessary a monitoring plan for the invertebrate community in the habitat in order to contribute to perform a species potential recovery plan in some of the isolated exotic *Acacia* spp. patches.

#### Use and trade

Use type: International

#### Ecosystem services

Ecosystem service type: Less important

Ecosystem services: 12. Biocontrol

#### Research needed

Research needed: 1.2. Research - Population size, distribution & trends1.3. Research - Life history & ecology3.1. Monitoring - Population trends3.4. Monitoring - Habitat trends

Justification for research needed: Further research is needed into its ecology and life history in order to find extant specimens and obtain information on population size, distribution and trends. It is also necessary a monitoring plan for the invertebrate community in the habitat in order to contribute to perform a species potential recovery plan in some of the isolated exotic *Acacia* spp. patches.

#### Viability analysis

Justification for probability: 

### Pseudanchomenus aptinoides

#### Species information

Scientific name: Pseudanchomenus aptinoides

Species authority: Tarnier, 1860

Synonyms: *Anchomenus aptinoides* Tarnier, 1860; *Azoranchus aptinoides* Tarnier, 1860

Common names: Ground beetle (English); Laurocho (Portuguese)

Kingdom: Animalia

Phylum: Arthropoda

Class: Insecta

Order: Coleoptera

Family: Carabidae

Taxonomic notes: *Pseudanchomenus aptinoides* was described from an individual collected in S. Miguel, on 1867, and deposited in the Natural History Museum of Paris. The specimens found more recently in Pico Island have no difference in the shape of aedeagus (PAV Borges, pers. observation).

**Figure 6.**
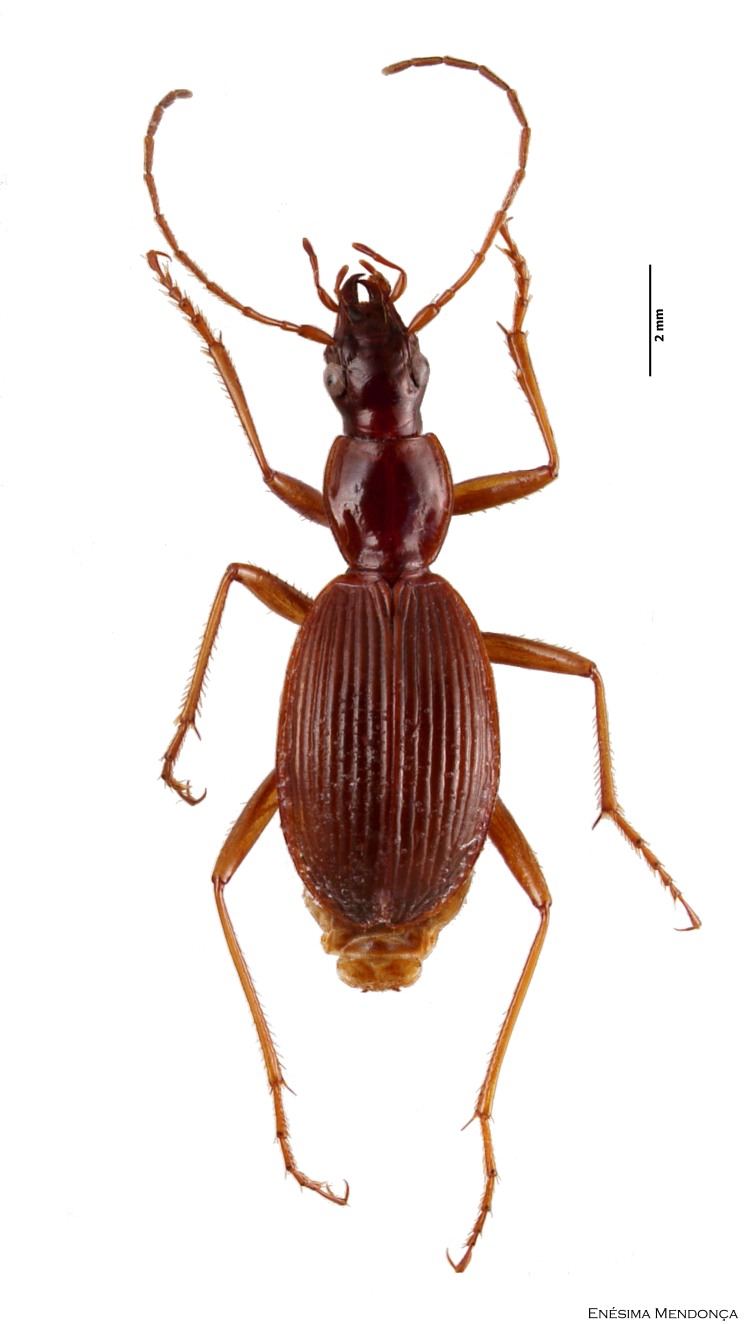
Female of *Pseudanchomenus aptinoides* from Cabeço do Caveiro (Pico, Azores) (Credit: Enésima Mendonça).

Figure(s) or Photo(s): Fig. 6

Region for assessment: Global

#### Editor & Reviewers

##### Reviewers

Reviewers: Anja Danielczak

##### Editor

Editor: Axel Hochkirch

#### Reviewers

Reviewers: Anja Danielczak

#### Editor

Editor: Axel Hochkirch

#### Geographic range

Biogeographic realm: Palearctic

Countries: Portugal

Map of records (Google Earth): Suppl. material 10

Basis of EOO and AOO: Observed

Basis (narrative): *Pseudanchomenus aptinoides* is an endemic species present in Pico and S. Miguel islands (Azores, Portugal) (Borges et al. 2010), known from Natural Forest Reserves of Caveiro, Lagoa do Caiado and Mistério da Prainha in Pico island and in Furnas in S. Miguel island. After some surveys in Furnas the species was not found in the last 20 years despite some intensive search (Borges et al. 2005, Borges et al. 2016), and is potentially considered extinct in S. Miguel.

Min Elevation/Depth (m): 800

Max Elevation/Depth (m): 1200

Range description: The extent of occurrence (EOO) is therefore only 16 km^2^ and the maximum area of occupancy (AOO) is 16 km^2^.

#### New occurrences

#### Extent of occurrence

EOO (km2): 16

Trend: Decline (inferred)

Justification for trend: The EOO was calculated based on Pico distribution. The species continues in decline due to ongoing native forest degradation and habitat fragmentation. The species is considered extinct in Furnas locality at S. Miguel due to major land-use changes and habitat destruction.

Causes ceased?: No

Causes understood?: Yes

Causes reversible?: Unknown

Extreme fluctuations?: Unknown

#### Area of occupancy

Trend: Decline (inferred)

Justification for trend: The species occurs in native forest patches included in the Natural Reserves of Caveiro, Lagoa do Caiado and Mistério da Prainha in Pico island and it is considered extinct in S. Miguel (Furnas), due to habitat destruction. The species continues in decline due to native forest degradation and habitat fragmentation. Despite an AOO of 16 km^2^, the current areal area of available native forest is only 9.52 km^2^.

Causes ceased?: No

Causes understood?: Yes

Causes reversible?: Unknown

Extreme fluctuations?: Unknown

AOO (km2): 16

#### Locations

Number of locations: 3

Justification for number of locations: The species occurs in several native forest patches in Pico, and in a location in a S. Miguel island (Furnas), but possibly extinct there.

Trend: Decline (observed)

Justification for trend: Three locations known but the original area was larger. In one of the locations now is possibly extinct. In the last ten years a spread of invasive plants (namely *Hedychium gardnerianum*) is changing the habitat structure, namely decreasing the cover of bryophytes and ferns in the soil and promoting the spread of other plants.

Extreme fluctuations?: Unknown

#### Population

Trend: Decline (inferred)

Justification for trend: The species is relatively abundant in Pico Island native forest in particular in the pristine fragment of Caveiro. In spite of several sampling efforts in the last 20 years no individuals were sampled in S. Miguel (Borges et al. 2005, Borges et al. 2016), and consequently the subpopulation of this island is possibly considered extinct, which is inferred from major land-use changes in this locality in the last 100 years, that were maintained in the last ten years with increaing pasture development and urbanization.

Basis for decline: (c) a decline in area of occupancy, extent of occurrence and/or quality of habitat

Causes ceased?: No

Causes understood?: Yes

Causes reversible?: Unknown

Extreme fluctuations?: Unknown

#### Subpopulations

Number of subpopulations: 3

Trend: Decline (inferred)

Justification for trend: The species is relatively abundant in Pico Island native forest in particular in the pristine fragment of Caveiro. In spite of several sampling efforts in the last 20 years no individuals were sampled in S. Miguel (Borges et al. 2005, Borges et al. 2016), and consequently the subpopulation of this island is considered extinct, which is inferred from major land-use changes in this locality in the last 100 years, that were maintained in the last ten years with increasing pasture development and urbanization.

Extreme fluctuations?: Unknown

Severe fragmentation?: Yes

Justification for fragmentation: Major land-use changes at middle and high elevations in Pico and S. Miguel promoted the creation of small patches of native forest. The species is confirmed to occur in three natural forest fragments in Pico that are isolated in a sea of pastures and *Cryptomeria japonica* plantations. In at least half of the locations in the last ten years a spread of invasive plants is changing the habitat structure, namely decreasing the cover of bryophytes and ferns in the soil and promoting the spread of other plants. At least two of the locations will be under severe threat in the next 10 years, with only Caveiro area still having some resistance to the spread of the invasive plant *Hedychium gardnerianum*.

#### Habitat

System: Terrestrial

Habitat specialist: Yes

Habitat (narrative): The species occurs in native forests dominated by montane *Juniperus brevifolia* woodland in Caveiro but also lavic formations dominated by *Erica azorica* in Mistério da Prainha, in the island of Pico, with an altitudinal range between 800 and 1200 m

Trend in extent, area or quality?: Decline (observed)

Justification for trend: Due to ongoing invasion of exotic plants that are changing the structure of the forest and the cover of bryophytes and ferns in the soil with impacts on native invertebrate species.

##### Habitat

Habitat importance: Major Importance

Habitats: 1.4. Forest - Temperate

#### Habitat

Habitat importance: Major Importance

Habitats: 1.4. Forest - Temperate

#### Ecology

Size: 1.2 cm

Generation length (yr): 1

Dependency of single sp?: No

Ecology and traits (narrative): This is an univoltine species. It is a nocturnal predator that lives in native trees and in the soil, particularly in ravines. Based on seasonal data from SLAM traps obtained in several islands between 2012 and 2016 (see Borges et al. 2017b), the adults are active all year, being most abundant in summer and autumn.

#### Threats

Justification for threats: In the past, the species has probably strongly declined due to changes in habitat size and quality and its large body size (Terzopoulou et al. 2015). Currently, *Cryptomeria japonica* wood & pulp plantations management and invasive plant species spreading (e.g. *Hedychium gardnerianum*;) are changing the structure of the forest and the cover of bryophytes and ferns in the soil with impacts on the species. Based on Ferreira et al. (2016) the habitat will further decline as a consequence of climate change (increasing number of droughts and habitat shifting & alteration). The species is considered extinct in S. Miguel island due to major historical land-use changes with clearing of original habitat.

##### Threats

Threat type: Ongoing

Threats: 2.2.1. Agriculture & aquaculture - Wood & pulp plantations - Small-holder plantations8.1.2. Invasive and other problematic species, genes & diseases - Invasive non-native/alien species/diseases - Named species

##### Threats

Threat type: Future

Threats: 11.1. Climate change & severe weather - Habitat shifting & alteration11.2. Climate change & severe weather - Droughts

#### Threats

Threat type: Ongoing

Threats: 2.2.1. Agriculture & aquaculture - Wood & pulp plantations - Small-holder plantations8.1.2. Invasive and other problematic species, genes & diseases - Invasive non-native/alien species/diseases - Named species

#### Threats

Threat type: Future

Threats: 11.1. Climate change & severe weather - Habitat shifting & alteration11.2. Climate change & severe weather - Droughts

#### Conservation

Justification for conservation actions: The species is not protected by regional law. Its habitat is in a regionally protected area (Natural Park of Pico). Degraded habitats should be restored and of critical importance is the continued expansion and linking of habitat fragments as well as removal of invasive non-native species where this is possible. A strategy needs to be developed to address the future threat by climate change. It is necessary a monitoring plan for the invertebrate community in the habitat in order to contribute to the conservation of this species. A habitat management plan is needed and anticipated to be developed during the coming years. Formal education and awareness is needed to allow future investments in restored habitats invaded by invasive plants.

##### Conservation actions

Conservation action type: In Place

Conservation actions: 1. Land/water protection1.1. Land/water protection - Site/area protection1.2. Land/water protection - Resource & habitat protection2. Land/water management2.2. Land/water management - Invasive/problematic species control4. Education & awareness

##### Conservation actions

Conservation action type: Needed

Conservation actions: 2.1. Land/water management - Site/area management2.2. Land/water management - Invasive/problematic species control2.3. Land/water management - Habitat & natural process restoration4.1. Education & awareness - Formal education4.3. Education & awareness - Awareness & communications5.4.3. Law & policy - Compliance and enforcement - Sub-national level

#### Conservation actions

Conservation action type: In Place

Conservation actions: 1. Land/water protection1.1. Land/water protection - Site/area protection1.2. Land/water protection - Resource & habitat protection2. Land/water management2.2. Land/water management - Invasive/problematic species control4. Education & awareness

#### Conservation actions

Conservation action type: Needed

Conservation actions: 2.1. Land/water management - Site/area management2.2. Land/water management - Invasive/problematic species control2.3. Land/water management - Habitat & natural process restoration4.1. Education & awareness - Formal education4.3. Education & awareness - Awareness & communications5.4.3. Law & policy - Compliance and enforcement - Sub-national level

#### Other

Justification for use and trade: This species is not utilized.

##### Use and trade

Use type: International

##### Ecosystem services

Ecosystem service type: Less important

Ecosystem services: 12. Biocontrol

##### Research needed

Research needed: 1.2. Research - Population size, distribution & trends1.3. Research - Life history & ecology2.2. Conservation Planning - Area-based Management Plan3.1. Monitoring - Population trends3.4. Monitoring - Habitat trends

Justification for research needed: Further research is needed into its ecology and life history in order to find extant specimens in S.Miguel and obtain information on population size, distribution and trends in both S. Miguel and Pico islands. It is also necessary an area-based management plan and a monitoring plan for the invertebrate community in the habitat in order to contribute to perform a species potential recovery plan. Monitoring every ten years using the BALA protocol will inform about habitat quality (see e.g. Gaspar et al. 2011).

#### Use and trade

Use type: International

#### Ecosystem services

Ecosystem service type: Less important

Ecosystem services: 12. Biocontrol

#### Research needed

Research needed: 1.2. Research - Population size, distribution & trends1.3. Research - Life history & ecology2.2. Conservation Planning - Area-based Management Plan3.1. Monitoring - Population trends3.4. Monitoring - Habitat trends

Justification for research needed: Further research is needed into its ecology and life history in order to find extant specimens in S.Miguel and obtain information on population size, distribution and trends in both S. Miguel and Pico islands. It is also necessary an area-based management plan and a monitoring plan for the invertebrate community in the habitat in order to contribute to perform a species potential recovery plan. Monitoring every ten years using the BALA protocol will inform about habitat quality (see e.g. Gaspar et al. 2011).

#### Viability analysis

Justification for probability: 

### Trechus terrabravensis

#### Species information

Scientific name: Trechus terrabravensis

Species authority: Borges, Serrano & Amorim, 2004

Common names: Ground beetle (English); Carocho-da-terra-brava (Portuguese)

Kingdom: Animalia

Phylum: Arthropoda

Class: Insecta

Order: Coleoptera

Family: Carabidae

Taxonomic notes: *Trechus terrabravensis* was described from three individuals (one male and two females), collected from 18.VII.2001 to 02.VIII.2001, in Terceira island (Terra Brava). The aedeagus of this species clearly resembles those of the cavernicolous troglobitic species *Trechus jorgensis* (S. Jorge) and *T. pereirai* (Pico) (Borges et al. 2004).

**Figure 7.**
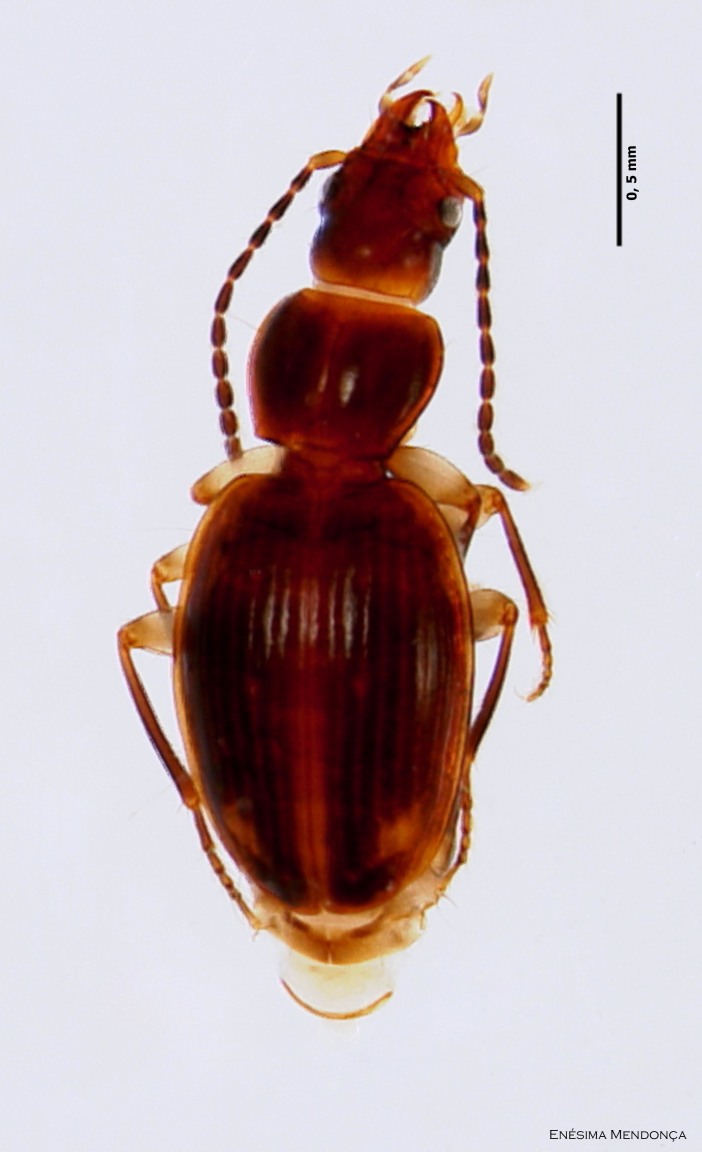
Male of *Trechus terrabravensis* from Terrra Brava (Terceira, Azores) (Credit: Enésima Mendonça).

Figure(s) or Photo(s): Fig. 7

Region for assessment: Global

#### Editor & Reviewers

##### Reviewers

Reviewers: Anja Danielczak

##### Editor

Editor: Axel Hochkirch

#### Reviewers

Reviewers: Anja Danielczak

#### Editor

Editor: Axel Hochkirch

#### Geographic range

Biogeographic realm: Palearctic

Countries: Portugal

Map of records (Google Earth): Suppl. material 11

Basis of EOO and AOO: Observed

Basis (narrative): *Trechus terrabravensis* is a single-island endemic species restricted to Terceira (Azores, Portugal) (Borges et al. 2010), known from the Natural Forest Reserves of Biscoito da Ferraria e Pico Alto, Pico do Galhardo, Terra Brava and Serra de Sta Bárbara.

Min Elevation/Depth (m): 500

Max Elevation/Depth (m): 1000

Range description: The extent of occurrence (EOO) is 32 km^2^ and the maximum area of occupancy (AOO) is 32 km^2^.

#### New occurrences

#### Extent of occurrence

EOO (km2): 32

Trend: Decline (inferred)

Justification for trend: This species occurs in native forests included in several protected areas of Terceira island. The extent of occurrence of this species continues to decline due to habitat degradation in the native forest (mainly *Juniperus brevifolia*-*Laurus azorica* and Ilex perado subsp. azorica forests) mostly caused by invasive plants, and habitat fragmentation.

Causes ceased?: No

Causes understood?: Yes

Causes reversible?: Unknown

Extreme fluctuations?: Unknown

#### Area of occupancy

Trend: Decline (inferred)

Justification for trend: This species occurs in native forests included in several protected areas of Terceira island. The AOO including only native forest is around 21 km². The area of occupancy of this species continues to decline due to habitat degradation in the native forest (mainly *Juniperus brevifolia*-*Laurus azorica* and *Ilex perado subsp. azorica* forests) mostly caused by invasive plants, and habitat fragmentation.

Causes ceased?: No

Causes understood?: Yes

Causes reversible?: Unknown

Extreme fluctuations?: Unknown

AOO (km2): 32

#### Locations

Number of locations: 4

Justification for number of locations: The species occurs in four native forest patches included in Natural Park of the Terceira island, and two of them (Biscoito da Ferraria e Pico Alto, Pico do Galhardo) were highly impacted by invasive plants in the last ten years having a low Index of Biotic Integrity (Gaspar et al. 2011).

Trend: Decline (inferred)

Justification for trend: Four locations known but the last ten years we observed a spread of invasive plants changing the habitat structure, namely decreasing the cover of bryophytes and ferns in the soil and promoting the spread of other plants.

Extreme fluctuations?: Unknown

#### Population

Trend: Decline (observed)

Justification for trend: The species is particularly restricted but abundant in some of the localities (Gaston et al. 2006). A continuing decline in the number of mature individuals is inferred from monitoring schemes (Borges et al. 2005, Borges et al. 2016) and from the ongoing habitat degradation due to invasions of alien plants (Gaspar et al. 2011).

Basis for decline: (c) a decline in area of occupancy, extent of occurrence and/or quality of habitat

Causes ceased?: Yes

Causes understood?: Yes

Causes reversible?: Unknown

Extreme fluctuations?: No

#### Subpopulations

Number of subpopulations: 4

Trend: Decline (inferred)

Justification for trend: The species is particularly restricted but abundant in some of the subpopulations in Terra Brava and Caldeira St. Barbara (Gaston et al. 2006). A continuing decline in the number of subpopulations is inferred from monitoring schemes (Borges et al. 2005, Borges et al. 2016) and from the ongoing habitat degradation due to invasions of alien plants in Biscoito da Ferraria e Pico Alto and Pico do Galhardo.

Extreme fluctuations?: Unknown

Severe fragmentation?: Yes

Justification for fragmentation: Major land-use changes at middle elevations promoted the creation of small patches of native forest. The species occurs in four natural forest fragments that are isolated in a sea of pastures and *Cryptomeria japonica* plantations, and is very rare in two of the subpopulations (Biscoito da Ferraria e Pico Alto and Pico do Galhardo).

#### Habitat

System: Terrestrial

Habitat specialist: Yes

Habitat (narrative): This species occurs deep inside very humid laurel forests (native forests dominated by *Laurus azorica*, Ilex perado subsp. azorica and *Juniperus azorica*) on Terceira, with an altitudinal range between 500 and 1000 m. Several specimens were collected in leaf litter, suggesting that this is a litter species. In both Terra Brava and Caldeira da Serra de Santa Bárbara, the terrain is basaltic with a system of cracks and deep holes and the forest floor is covered by a dense carpet of mosses and ferns with little light reaching the ground (Borges et al. 2004)

Trend in extent, area or quality?: Decline (inferred)

Justification for trend: In the past, the species has probably strongly declined due to changes in habitat size and quality. Currently invasive plant species are decreasing the quality of the habitat changing the habitat structure, namely decreasing the cover of bryophytes and ferns in the soil and promoting the spread of other plants.

##### Habitat

Habitat importance: Major Importance

Habitats: 1.4. Forest - Temperate

#### Habitat

Habitat importance: Major Importance

Habitats: 1.4. Forest - Temperate

#### Ecology

Size: 0.35 cm

Generation length (yr): 1

Dependency of single sp?: No

Ecology and traits (narrative): This is an univoltine species and it is a predator that lives in the soil litter in hyperhumid conditions.

#### Threats

Justification for threats: In the past, the species has probably strongly declined due to changes in habitat size and quality and lack of resources due to its large body size (Terzopoulou et al. 2015). Currently, *Cryptomeria japonica* wood & pulp plantations management and invasive plant species spreading (e.g. *Hedychium gardnerianum*) are changing the structure of the forest and the cover of bryophytes and ferns in the soil with impacts on the species. Based on Ferreira et al. (2016) the habitat will further decline as a consequence of climate change (increasing number of droughts and habitat shifting & alteration).

##### Threats

Threat type: Ongoing

Threats: 2.2.1. Agriculture & aquaculture - Wood & pulp plantations - Small-holder plantations8.1.2. Invasive and other problematic species, genes & diseases - Invasive non-native/alien species/diseases - Named species

##### Threats

Threat type: Future

Threats: 11.1. Climate change & severe weather - Habitat shifting & alteration11.2. Climate change & severe weather - Droughts

#### Threats

Threat type: Ongoing

Threats: 2.2.1. Agriculture & aquaculture - Wood & pulp plantations - Small-holder plantations8.1.2. Invasive and other problematic species, genes & diseases - Invasive non-native/alien species/diseases - Named species

#### Threats

Threat type: Future

Threats: 11.1. Climate change & severe weather - Habitat shifting & alteration11.2. Climate change & severe weather - Droughts

#### Conservation

Justification for conservation actions: The species is not protected by regional law. Its habitat is in a regionally protected area (Natural Park of Terceira). The Terceira Natural Park administration is currently starting control measures of the invasive plants. Degraded habitats should be restored and a strategy needs to be developed to address the future threat by climate change. It is necessary a monitoring plan for the invertebrate community in the habitat in order to contribute to the conservation of this species. A habitat management plan is needed and anticipated to be developed during the coming years.

##### Conservation actions

Conservation action type: In Place

Conservation actions: 1. Land/water protection1.1. Land/water protection - Site/area protection1.2. Land/water protection - Resource & habitat protection2. Land/water management2.2. Land/water management - Invasive/problematic species control

##### Conservation actions

Conservation action type: Needed

Conservation actions: 2.1. Land/water management - Site/area management2.2. Land/water management - Invasive/problematic species control2.3. Land/water management - Habitat & natural process restoration4. Education & awareness5.4.3. Law & policy - Compliance and enforcement - Sub-national level

#### Conservation actions

Conservation action type: In Place

Conservation actions: 1. Land/water protection1.1. Land/water protection - Site/area protection1.2. Land/water protection - Resource & habitat protection2. Land/water management2.2. Land/water management - Invasive/problematic species control

#### Conservation actions

Conservation action type: Needed

Conservation actions: 2.1. Land/water management - Site/area management2.2. Land/water management - Invasive/problematic species control2.3. Land/water management - Habitat & natural process restoration4. Education & awareness5.4.3. Law & policy - Compliance and enforcement - Sub-national level

#### Other

Justification for use and trade: The species is not utilized.

##### Use and trade

Use type: International

##### Ecosystem services

Ecosystem service type: Less important

Ecosystem services: 12. Biocontrol

##### Research needed

Research needed: 1.2. Research - Population size, distribution & trends1.3. Research - Life history & ecology2.2. Conservation Planning - Area-based Management Plan3.1. Monitoring - Population trends3.4. Monitoring - Habitat trends

Justification for research needed: Further research is needed into its ecology and life history in order to obtain information on population size, distribution and trends. It is also necessary an area-based management plan and a monitoring plan for the invertebrate community in the habitat in order to contribute to perform a species potential recovery plan as a consequence of invasive plant species spread in two native forest fragments (Biscoito da Ferraria e Pico Alto and Pico do Galhardo). Monitoring every ten years using the BALA protocol will inform about habitat quality (see e.g. Gaspar et al. 2011).

#### Use and trade

Use type: International

#### Ecosystem services

Ecosystem service type: Less important

Ecosystem services: 12. Biocontrol

#### Research needed

Research needed: 1.2. Research - Population size, distribution & trends1.3. Research - Life history & ecology2.2. Conservation Planning - Area-based Management Plan3.1. Monitoring - Population trends3.4. Monitoring - Habitat trends

Justification for research needed: Further research is needed into its ecology and life history in order to obtain information on population size, distribution and trends. It is also necessary an area-based management plan and a monitoring plan for the invertebrate community in the habitat in order to contribute to perform a species potential recovery plan as a consequence of invasive plant species spread in two native forest fragments (Biscoito da Ferraria e Pico Alto and Pico do Galhardo). Monitoring every ten years using the BALA protocol will inform about habitat quality (see e.g. Gaspar et al. 2011).

#### Viability analysis

Justification for probability: 

### Trechus torretassoi

#### Species information

Scientific name: Trechus torretassoi

Species authority: Jeannel, 1937

Common names: Ground beetle (English); Carocho (Portuguese)

Kingdom: Animalia

Phylum: Arthropoda

Class: Insecta

Order: Coleoptera

Family: Carabidae

Taxonomic notes: This species was described from individuals collected in S. Miguel island (Sete Cidades), on the 4.VI.1935. The hind-wings are reduced to almost inconspicuous rudiments (Lindroth 1960). Later was cited to Furnas (Gillerfors 1986a) and never found after that.

Region for assessment: Global

#### Editor & Reviewers

##### Reviewers

Reviewers: Anja Danielczak

##### Editor

Editor: Axel Hochkirch

#### Reviewers

Reviewers: Anja Danielczak

#### Editor

Editor: Axel Hochkirch

#### Geographic range

Biogeographic realm: Palearctic

Countries: Portugal

Map of records (Google Earth): Suppl. material 12

Basis of EOO and AOO: Observed

Basis (narrative): *Trechus torretassoi* is a single-island endemic species known from S. Miguel (Azores, Portugal) (Borges et al. 2010), and occurring historically only at Furnas and Sete Cidades volcanos.

Min Elevation/Depth (m): 700

Max Elevation/Depth (m): 845

Range description: The extent of occurrence (EOO) is 24 km^2^ and the maximum area of occupancy (AOO) is 12 km^2^. The species is considered possibly extinct (Terzopoulou et al. 2015) due to destruction of the habitat in all its range.

#### New occurrences

#### Extent of occurrence

EOO (km2): 0-24

Trend: Stable

Justification for trend: The species occured in two areas now dominated by exotic plantations and pastures in S. Miguel island (Sete Cidades and Furnas). The extent of occurrence of this species declined due to habitat degradation in the native forest, mostly caused by invasive plants, and suitable habitat has been extirpated from areas where the species once occurred (historical distribution). Now the species is considered extinct more than 10 years ago (Terzopoulou et al. 2015).

Causes ceased?: No

Causes understood?: Yes

Causes reversible?: Unknown

Extreme fluctuations?: Unknown

#### Area of occupancy

Trend: Stable

Justification for trend: The species occured in two areas now dominated by exotic plantations and pastures in S. Miguel island. The area of occupancy of this species declined in the last decades due to habitat degradation in the native forest, mostly caused by invasive plants, and suitable habitat has been extirpated from areas where the species once occurred (historical distribution). Now the species is considered extinct more than 10 years ago (Terzopoulou et al. 2015).

Causes ceased?: No

Causes understood?: Yes

Causes reversible?: Unknown

Extreme fluctuations?: Unknown

AOO (km2): 0-12

#### Locations

Number of locations: 0-2

Justification for number of locations: The species occured in two areas now dominated by exotic plantations and pastures in S. Miguel island (Furnas and Sete Cidades). Now the species is considered extinct more than 10 years ago (Terzopoulou et al. 2015).

Trend: Stable

Justification for trend: Possibly went extinct more than 10 years ago.

Extreme fluctuations?: Unknown

#### Population

Trend: Stable

Justification for trend: The species was historically particularly restricted and with very low number of sampled individuals. A continuing decline in the number of mature individuals is inferred from monitoring schemes, from massive land-use changes in the last 100 years and the ongoing habitat degradation due to invasions of alien plants in the last 10 years. Despite intensive collecting efforts in the past 20 years (Borges et al. 2005, Borges et al. 2016), no specimens have been collected since 1985 and according to Terzopoulou et al. (2015) this species is possibly extinct.

Causes ceased?: No

Causes understood?: Yes

Causes reversible?: Unknown

Extreme fluctuations?: Unknown

#### Subpopulations

Number of subpopulations: 0-2

Trend: Stable

Justification for trend: The species was particularly restricted and with very low in number of sampled individuals. A possible decline in the number of subpopulations is inferred from monitoring schemes (Borges et al. 2005, Borges et al. 2016), from massive land-use changes in the last 50 years and the ongoing habitat degradation due to invasions of alien plants. Despite intensive collecting efforts in the past 20 years, no specimens have been collected since 1985 and according to Terzopoulou et al. (2015) this species was extinct more than ten years ago.

Extreme fluctuations?: Unknown

Severe fragmentation?: No

#### Habitat

System: Terrestrial

Habitat specialist: Yes

Habitat (narrative): This species occurred originally in native forest in S. Miguel island (Azores), with an altitudinal range between 700 and 845 m. However, if still extant it should occur in exotic plantations of *Cryptomeria japonica*. It is a predator that lives in the soil associated with debris and litter.

Trend in extent, area or quality?: Decline (inferred)

Justification for trend: Massive land-use changes in the last 100 years have probably led to strong declined of this species due to changes in habitat size and quality.

##### Habitat

Habitat importance: Major Importance

Habitats: 1.4. Forest - Temperate14.3. Artificial/Terrestrial - Plantations16. Introduced vegetation

#### Habitat

Habitat importance: Major Importance

Habitats: 1.4. Forest - Temperate14.3. Artificial/Terrestrial - Plantations16. Introduced vegetation

#### Ecology

Size: 0.30-0.32 cm

Generation length (yr): 1

Dependency of single sp?: No

Ecology and traits (narrative): This is an univoltine species and it is a predator that lives in the soil associated with debris and litter. In the last record in 1985 it was sampled associated with debris in a lake shore near a *Cryptomeria japonica* plantation (Gillerfors 1986a).

#### Threats

Justification for threats: In the past, the species has probably strongly declined due to deforestation (Triantis et al. 2010). The species is potentially considered extinct due to major historical land-use changes with clearing of original habitat (Terzopoulou et al. 2015). The most important ongoing threat to this species is *Cryptomeria japonica* wood & pulp plantations management and the spread of invasive plants (*Hedychium gardnerianum*) that are changing the habitat structure, namely decreasing the cover of bryophytes and ferns in the soil and promoting the spread of other plants. Based on Ferreira et al. (2016) the habitat will further decline as a consequence of climate change (increasing number of droughts and habitat shifting & alteration).

##### Threats

Threat type: Ongoing

Threats: 2.2.1. Agriculture & aquaculture - Wood & pulp plantations - Small-holder plantations8.1.2. Invasive and other problematic species, genes & diseases - Invasive non-native/alien species/diseases - Named species

##### Threats

Threat type: Future

Threats: 11.1. Climate change & severe weather - Habitat shifting & alteration11.2. Climate change & severe weather - Droughts

#### Threats

Threat type: Ongoing

Threats: 2.2.1. Agriculture & aquaculture - Wood & pulp plantations - Small-holder plantations8.1.2. Invasive and other problematic species, genes & diseases - Invasive non-native/alien species/diseases - Named species

#### Threats

Threat type: Future

Threats: 11.1. Climate change & severe weather - Habitat shifting & alteration11.2. Climate change & severe weather - Droughts

#### Conservation

Justification for conservation actions: The species is not protected by regional law. It is also necessary a monitoring plan for the invertebrate community in the habitat in order to contribute to perform a species potential recovery plan in Furnas and Sete Cidades. Therefore, degraded habitats in Furnas and Sete Cidades should be restored and a strategy needs to be developed to address the ongoing threat by invasive species and future threat by climate change. Formal education and awareness is needed to allow future investments in restored habitats invaded by invasive plants.

##### Conservation actions

Conservation action type: Needed

Conservation actions: 2.1. Land/water management - Site/area management2.2. Land/water management - Invasive/problematic species control2.3. Land/water management - Habitat & natural process restoration4. Education & awareness5.4.3. Law & policy - Compliance and enforcement - Sub-national level

#### Conservation actions

Conservation action type: Needed

Conservation actions: 2.1. Land/water management - Site/area management2.2. Land/water management - Invasive/problematic species control2.3. Land/water management - Habitat & natural process restoration4. Education & awareness5.4.3. Law & policy - Compliance and enforcement - Sub-national level

#### Other

Justification for use and trade: The species is not utilized.

##### Use and trade

Use type: International

##### Ecosystem services

Ecosystem service type: Less important

Ecosystem services: 12. Biocontrol

##### Research needed

Research needed: 1.2. Research - Population size, distribution & trends1.3. Research - Life history & ecology3.1. Monitoring - Population trends3.4. Monitoring - Habitat trends

Justification for research needed: Further research is needed into its ecology and life history in order to find extant specimens in historical areas of Furnas and Sete Cidades. It is also necessary a monitoring plan for the invertebrate community in the habitat in order to contribute to perform a species potential recovery plan.

#### Use and trade

Use type: International

#### Ecosystem services

Ecosystem service type: Less important

Ecosystem services: 12. Biocontrol

#### Research needed

Research needed: 1.2. Research - Population size, distribution & trends1.3. Research - Life history & ecology3.1. Monitoring - Population trends3.4. Monitoring - Habitat trends

Justification for research needed: Further research is needed into its ecology and life history in order to find extant specimens in historical areas of Furnas and Sete Cidades. It is also necessary a monitoring plan for the invertebrate community in the habitat in order to contribute to perform a species potential recovery plan.

#### Viability analysis

Justification for probability: 

### Mniophilosoma obscurum

#### Species information

Scientific name: Mniophilosoma obscurum

Species authority: Gillerfors, 1986

Common names: Leaf beetle (English); Escaravelho-do-musgão (Portuguese)

Kingdom: Animalia

Phylum: Arthropoda

Class: Insecta

Order: Coleoptera

Family: Chrysomelidae

Taxonomic notes: *Mniophilosoma obscurum* was described from a single male, collected in Flores island (Caldeira Comprida), on the 15.VI.1985. It is deposited in Museu Municipal do Funchal (Madeira). This species is very similar to the Madeiran *M. laeve* Wollaston, that has a body-form suborbiculate-ovate, exceedingly convex, but easily separated from that species by rather strong microsculpture of upper surface; by being apparently glabrous impunctate and by structure of male genitalia (Gillerfors 1986b).

Region for assessment: Global

#### Editor & Reviewers

##### Reviewers

Reviewers: Anja Danielczak

##### Editor

Editor: Axel Hochkirch

#### Reviewers

Reviewers: Anja Danielczak

#### Editor

Editor: Axel Hochkirch

#### Geographic range

Biogeographic realm: Palearctic

Countries: Portugal

Map of records (Google Earth): Suppl. material 13

Basis of EOO and AOO: Observed

Basis (narrative): *Mniophilosoma obscurum* is a single-island endemic species restricted to Flores (Azores, Portugal) (Borges et al. 2010), known from Natural Forest Reserve of Morro Alto e Pico da Sé.

Min Elevation/Depth (m): 560

Max Elevation/Depth (m): 880

Range description: The extent of occurrence (EOO) is 13 km^2^ and the maximum area of occupancy (AOO) is 4 km^2^.

#### New occurrences

#### Extent of occurrence

EOO (km2): 13

Trend: Stable

Justification for trend: This species occurs in a small fragment of native forest in Flores island. The extent of occurrence is stable in the last 60 years and has a high value of biotic integrity (Gaspar et al. 2011).

Causes ceased?: No

Causes understood?: Yes

Causes reversible?: Unknown

Extreme fluctuations?: Unknown

#### Area of occupancy

Trend: Decline (inferred)

Justification for trend: This species occurs in a small fragment of native forest in Flores island. In spite for the fact that the whole nature reserve has some levels of biotic integrity (Gaspar et al. 2011), part of the area is being recently invaded by two invasive plants (*Hydrangea macrophylla* and *Hedychium gardnerianum)* with destruction of some areas.

Causes ceased?: No

Causes understood?: Yes

Causes reversible?: Unknown

Extreme fluctuations?: Unknown

AOO (km2): 4

#### Locations

Number of locations: 1

Justification for number of locations: The species occurs in a single native forest fragment in the Flores island that is starting to be impacted by invasive plants (*Hydrangea macrophylla* and *Hedychium gardnerianum)* that are disrupting the quality of forest ground.

Trend: Stable

Justification for trend: Between 1940 and 1950 major land-use changes occurred in the island, the reserve has a high Index of Biotic Integrity (Gaspar et al. 2011), but the historical site is already under strong impact of invasive plants that are decreasing the cover of *Sphagnum* spp., disrupting the quality of forest ground, habitat of the species.

Extreme fluctuations?: Unknown

#### Population

Trend: Decline (inferred)

Justification for trend: The species is rare and only known from a single subpopulation in Flores island. The surrounding area is protected and it is well preserved with a high Index of Biotic Integrity (Gaspar et al. 2011). However, part of the AAO is starting to be impacted by invasive plants (*Hydrangea macrophylla* and *Hedychium gardnerianum)* that are disrupting the quality of forest ground with potential decline un the number of individuals.

Causes ceased?: No

Causes understood?: Yes

Causes reversible?: Unknown

Extreme fluctuations?: Unknown

#### Subpopulations

Number of subpopulations: 1

Trend: Stable

Justification for trend: The species is rare and only known from a single subpopulation in Flores island. The surrounding area is protected and it is well preserved with a high Index of Biotic Integrity (Gaspar et al. 2011). However, part of the AAO is starting to be impacted by invasive plants (*Hydrangea macrophylla* and *Hedychium gardnerianum)* that are disrupting the quality of forest ground and the supopulation may be under threat.

Extreme fluctuations?: Unknown

Severe fragmentation?: No

#### Habitat

System: Terrestrial

Habitat specialist: Yes

Habitat (narrative): This species occurs in a small fragment of native forest in Flores island (Azores), dominated by *Juniperus brevifolia*, *Calluna vulgaris* shrubs and *Sphagnum* spp. moss (Gillerfors 1986a), with an altitudinal range between 560 and 880 m.

Trend in extent, area or quality?: Decline (observed)

Justification for trend: The Habitat is stable in the last 60 years, part of the AAO is starting to be impacted by invasive plants (*Hydrangea macrophylla* and *Hedychium gardnerianum)* that are disrupting the quality of forest ground.

##### Habitat

Habitat importance: Major Importance

Habitats: 1.4. Forest - Temperate

#### Habitat

Habitat importance: Major Importance

Habitats: 1.4. Forest - Temperate

#### Ecology

Size: 0.16 cm

Generation length (yr): 1

Dependency of single sp?: No

Ecology and traits (narrative): This is an univoltine species. Adults and larvae are herbivores and feed on all sorts of plant tissue in *Sphagnum* spp. bogs.

#### Threats

Justification for threats: In the past, the species has probably strongly declined due to changes in habitat size and quality (Triantis et al. 2010; Terzopoulou et al. 2015). Currently invasive plants *Hydrangea macrophylla* and *Hedychium gardnerianum* are changing some of the areas and decreasing the quality of the habitat, since are changing the habitat structure, namely decreasing the cover of bryophytes and ferns in the soil and promoting the spread of other plants. Based on Ferreira et al. (2016) the habitat will further decline as a consequence of climate change (increasing number of droughts and habitat shifting & alteration).

##### Threats

Threat type: Ongoing

Threats: 8.1.2. Invasive and other problematic species, genes & diseases - Invasive non-native/alien species/diseases - Named species

##### Threats

Threat type: Future

Threats: 11.1. Climate change & severe weather - Habitat shifting & alteration11.2. Climate change & severe weather - Droughts

#### Threats

Threat type: Ongoing

Threats: 8.1.2. Invasive and other problematic species, genes & diseases - Invasive non-native/alien species/diseases - Named species

#### Threats

Threat type: Future

Threats: 11.1. Climate change & severe weather - Habitat shifting & alteration11.2. Climate change & severe weather - Droughts

#### Conservation

Justification for conservation actions: The species is not protected by regional law. Its habitat is in a regionally protected area (Natural Park of Flores). Degraded habitats should be restored and a strategy needs to be developed to address the future threat by climate change. It is necessary a monitoring plan for the invertebrate community in the habitat in order to contribute to the conservation of this species. A habitat management plan is needed and anticipated to be developed during the coming years. Formal education and awareness is needed to allow future investments in restored habitats invaded by invasive plants.

##### Conservation actions

Conservation action type: In Place

Conservation actions: 1. Land/water protection1.1. Land/water protection - Site/area protection

##### Conservation actions

Conservation action type: Needed

Conservation actions: 2.1. Land/water management - Site/area management2.2. Land/water management - Invasive/problematic species control2.3. Land/water management - Habitat & natural process restoration4.1. Education & awareness - Formal education4.3. Education & awareness - Awareness & communications5.4.3. Law & policy - Compliance and enforcement - Sub-national level

#### Conservation actions

Conservation action type: In Place

Conservation actions: 1. Land/water protection1.1. Land/water protection - Site/area protection

#### Conservation actions

Conservation action type: Needed

Conservation actions: 2.1. Land/water management - Site/area management2.2. Land/water management - Invasive/problematic species control2.3. Land/water management - Habitat & natural process restoration4.1. Education & awareness - Formal education4.3. Education & awareness - Awareness & communications5.4.3. Law & policy - Compliance and enforcement - Sub-national level

#### Other

Justification for use and trade: This species is not utilized.

##### Use and trade

Use type: International

##### Ecosystem services

Ecosystem service type: Less important

Ecosystem services: 8. Habitat Maintenance

##### Research needed

Research needed: 1.2. Research - Population size, distribution & trends1.3. Research - Life history & ecology3.1. Monitoring - Population trends3.4. Monitoring - Habitat trends

Justification for research needed: Further research is needed into its ecology and life history in order to obtain information on population size, distribution and trends. Monitoring every ten years using the BALA protocol will inform about population trends and habitat quality (see e.g. Gaspar et al. 2011)

#### Use and trade

Use type: International

#### Ecosystem services

Ecosystem service type: Less important

Ecosystem services: 8. Habitat Maintenance

#### Research needed

Research needed: 1.2. Research - Population size, distribution & trends1.3. Research - Life history & ecology3.1. Monitoring - Population trends3.4. Monitoring - Habitat trends

Justification for research needed: Further research is needed into its ecology and life history in order to obtain information on population size, distribution and trends. Monitoring every ten years using the BALA protocol will inform about population trends and habitat quality (see e.g. Gaspar et al. 2011)

#### Viability analysis

Justification for probability: 

### Atlantocis gillerforsi

#### Species information

Scientific name: Atlantocis gillerforsi

Species authority: Israelson, 1985

Common names: Minute tree beetle, fungus beetle (English)

Kingdom: Animalia

Phylum: Arthropoda

Class: Insecta

Order: Coleoptera

Family: Ciidae

Taxonomic notes: *Atlantocis gillerforsi* was described from several individuals collected in Santa Maria (Coevas) and S. Miguel (Povoação) islands, between 30.VII.1983 and 10.VIII.1983. These specimens are deposited in G. Gillerfors and G. Israelson collections (Israelson 1985c).

**Figure 8.**
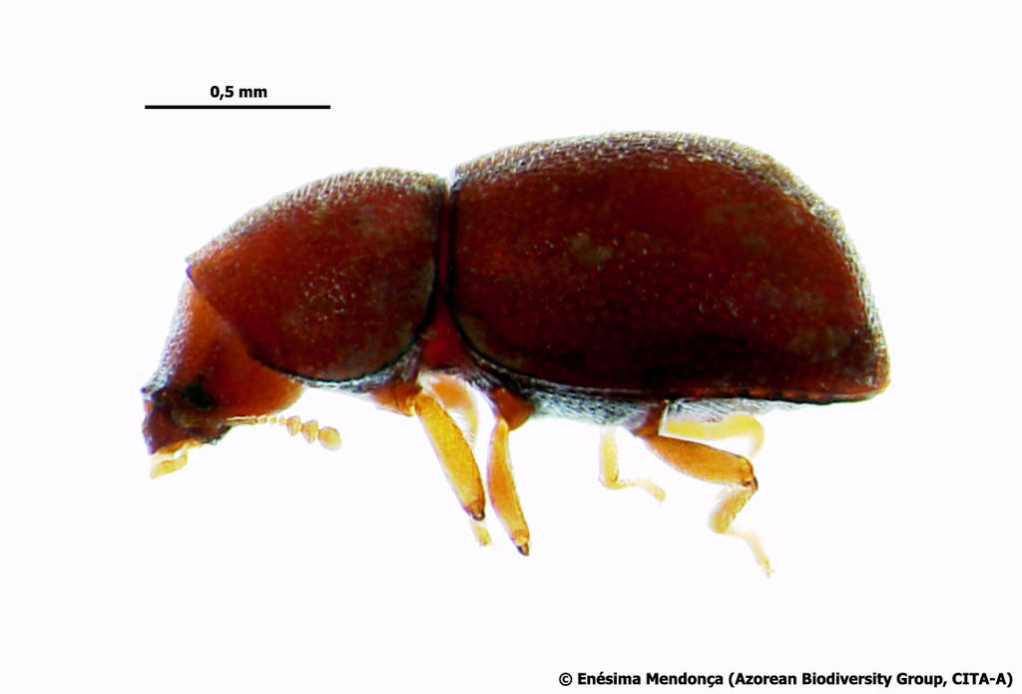
Female of *Atlantocis gillerforsi* from Caveiro (Pico, Azores) (Credit: Enésima Mendonça).

Figure(s) or Photo(s): Fig. 8

Region for assessment: Global

#### Editor & Reviewers

##### Reviewers

Reviewers: Anja Danielczak

##### Editor

Editor: Axel Hochkirch

#### Reviewers

Reviewers: Anja Danielczak

#### Editor

Editor: Axel Hochkirch

#### Geographic range

Biogeographic realm: Palearctic

Countries: Portugal

Map of records (Google Earth): Suppl. material 14

Basis of EOO and AOO: Observed

Basis (narrative): *Atlantocis gillerforsi* is an endemic species present in Flores, Terceira, Pico, S. Miguel and Santa Maria islands (Azores, Portugal) (Borges et al. 2010), known from Natural Forest Reserves of Morro Alto e Pico da Sé (Flores); Pico Galhardo and Terra Brava (Terceira); Atalhada and Pico da Vara (S. Miguel) and Pico Alto (Santa Maria).

Min Elevation/Depth (m): 350

Max Elevation/Depth (m): 1000

Range description: The extent of occurrence (EOO) is ca 34,000 km^2^ and the maximum area of occupancy (AOO) is 64 km^2^.

#### New occurrences

#### Extent of occurrence

EOO (km2): 34,000

Trend: Stable

Justification for trend: The species occurs in several islands and many natural areas, most of them currently well preserved.

Causes ceased?: No

Causes understood?: Yes

Causes reversible?: Unknown

Extreme fluctuations?: Unknown

#### Area of occupancy

Trend: Decline (inferred)

Justification for trend: The species occurs in areas of native and exotic forests of several islands (Flores, Terceira, Pico, S. Miguel and Santa Maria). The area with native forest is around one third of the AOO. The species continues in decline due to native forest degradation and habitat fragmentation.

Causes ceased?: No

Causes understood?: Yes

Causes reversible?: Unknown

Extreme fluctuations?: Unknown

AOO (km2): 64

#### Locations

Number of locations: 11

Justification for number of locations: The species occurs in eleven native and exotic forest patches in five islands (Flores, Terceira, Pico, S. Miguel and Santa Maria). All the locations are currently under severe invasion by plants like *Hedychium gardnerianum*, with major impacts on the structure of the forest floor.

Trend: Decline (inferred)

Justification for trend: Eleven locations known but the original area was larger with decreasing of habitat quality in the last 10 years due to land-use changes and invasive plants that are changing the habitat structure, namely decreasing the cover of bryophytes and ferns in the soil and promoting the spread of other plants. In fact, Pico Galhardo (Terceira); Atalhada and Pico da Vara (S. Miguel) and Pico Alto (Santa Maria) have a low Index of Biotic Integrity (Gaspar et al. 2011).

Extreme fluctuations?: Unknown

#### Population

Trend: Decline (inferred)

Justification for trend: The species is highly abundant. However, part of the AAO is starting to be impacted by invasive plants (*Hedychium gardnerianum*, *Pittosporum undulatum*) that are disrupting the quality of forest ground with potential decline in the number of individuals.

Causes ceased?: No

Causes understood?: Yes

Causes reversible?: Unknown

Extreme fluctuations?: Unknown

#### Subpopulations

Number of subpopulations: 7

Trend: Decline (inferred)

Justification for trend: The species is very abundant. However, some of the subpopulations are starting to be impacted by invasive plants (*Hedychium gardnerianum*, *Pittosporum undulatum*) that are disrupting the quality of forest ground with potential decline in the number of subpopulations.

Extreme fluctuations?: No

Severe fragmentation?: Yes

Justification for fragmentation: Major land-use changes at all elevations in the islands promoted the creation of small patches of native and exotic forest. The species occurs in seven natural forest fragments that are isolated in a sea of pastures and *Cryptomeria japonica* plantations. In the last ten years a spread of invasive plants is changing the habitat structure in more than 50% of the subpopulations, namely decreasing the cover of bryophytes and ferns in the soil and promoting the spread of other plants. The sites at Pico Galhardo (Terceira); Atalhada (S. Miguel) and Pico Alto (Santa Maria) have a low Index of Biotic Integrity (Gaspar et al. 2011).

#### Habitat

System: Terrestrial

Habitat specialist: Yes

Habitat (narrative): The species occurs in native (dominated by *Laurus azorica* and *Juniperus brevifolia*) and exotic (e.g. *Eucalyptus* spp.) forests of several islands (Flores, Terceira, Pico, S. Miguel and Santa Maria), with an altitudinal range between 350 and 1000 m. This species was an inhabitant of ancient azorean laurel forests and successfully adapted itself to changed conditions of life (habitat transformation) (Israelson 1985c).

Trend in extent, area or quality?: Decline (observed)

Justification for trend: The habitat was stable during last century. However, in the last ten years invasive plant species spreading (e.g. *Hedychium gardnerianum*; *Pittosporum undulatum*) are changing the structure of the forest and the cover of bryophytes and ferns in the soil decreasing the quality of the habitat with impacts on the species.

##### Habitat

Habitat importance: Major Importance

Habitats: 1.4. Forest - Temperate16. Introduced vegetation

#### Habitat

Habitat importance: Major Importance

Habitats: 1.4. Forest - Temperate16. Introduced vegetation

#### Ecology

Size: 0.14 - 0.17 cm

Generation length (yr): 1

Dependency of single sp?: No

Ecology and traits (narrative): This species feeds mostly on fungi. Based on seasonal data from SLAM traps obtained in several islands between 2012 and 2016, the adults are active all year (Borges et al. 2017b), being more abundant in summer.

#### Threats

Justification for threats: In the past, the species has probably strongly declined due to changes in habitat size and quality (Triantis et al. 2010, Terzopoulou et al. 2015). Currently invasive plants *Hydrangea macrophylla, Pittosporum undulatum* and *Hedychium gardnerianum* are changing some of the areas and decreasing the quality of the habitat, since are changing the habitat structure, namely decreasing the cover of bryophytes and ferns in the soil and promoting the spread of other plants. Based on Ferreira et al. (2016) the habitat will further decline as a consequence of climate change (increasing number of droughts and habitat shifting & alteration).

##### Threats

Threat type: Ongoing

Threats: 8.1.2. Invasive and other problematic species, genes & diseases - Invasive non-native/alien species/diseases - Named species

##### Threats

Threat type: Future

Threats: 11.1. Climate change & severe weather - Habitat shifting & alteration11.2. Climate change & severe weather - Droughts

#### Threats

Threat type: Ongoing

Threats: 8.1.2. Invasive and other problematic species, genes & diseases - Invasive non-native/alien species/diseases - Named species

#### Threats

Threat type: Future

Threats: 11.1. Climate change & severe weather - Habitat shifting & alteration11.2. Climate change & severe weather - Droughts

#### Conservation

Justification for conservation actions: The species is not protected by regional law. Its habitat is in regionally protected areas (Natural Parks of Flores, Terceira, S. Miguel and Santa Maria). Further spread of invasive plants needs to be stopped in order to avoid any future declines of the species. Degraded habitats should be restored and a strategy needs to be developed to address the future threat by climate change. A habitat management plan is needed and anticipated to be developed during the coming years. Formal education and awareness is needed to allow future investments in restored habitats invaded by invasive plants.

##### Conservation actions

Conservation action type: In Place

Conservation actions: 1. Land/water protection1.1. Land/water protection - Site/area protection1.2. Land/water protection - Resource & habitat protection2.2. Land/water management - Invasive/problematic species control

##### Conservation actions

Conservation action type: Needed

Conservation actions: 2.1. Land/water management - Site/area management2.2. Land/water management - Invasive/problematic species control2.3. Land/water management - Habitat & natural process restoration4. Education & awareness5.4.3. Law & policy - Compliance and enforcement - Sub-national level

#### Conservation actions

Conservation action type: In Place

Conservation actions: 1. Land/water protection1.1. Land/water protection - Site/area protection1.2. Land/water protection - Resource & habitat protection2.2. Land/water management - Invasive/problematic species control

#### Conservation actions

Conservation action type: Needed

Conservation actions: 2.1. Land/water management - Site/area management2.2. Land/water management - Invasive/problematic species control2.3. Land/water management - Habitat & natural process restoration4. Education & awareness5.4.3. Law & policy - Compliance and enforcement - Sub-national level

#### Other

Justification for use and trade: This species is not utilized.

##### Use and trade

Use type: International

##### Ecosystem services

Ecosystem service type: Less important

Ecosystem services: 8. Habitat Maintenance

##### Research needed

Research needed: 1.2. Research - Population size, distribution & trends1.3. Research - Life history & ecology2.2. Conservation Planning - Area-based Management Plan3.1. Monitoring - Population trends3.4. Monitoring - Habitat trends

Justification for research needed: Further research is needed into its ecology and life history in order to obtain information on population size, distribution and trends. It is necessary a monitoring plan for the invertebrate community in the habitat in order to contribute to the conservation of this species. Monitoring every ten years using the BALA protocol will inform about habitat quality (see e.g. Gaspar et al. 2011).

#### Use and trade

Use type: International

#### Ecosystem services

Ecosystem service type: Less important

Ecosystem services: 8. Habitat Maintenance

#### Research needed

Research needed: 1.2. Research - Population size, distribution & trends1.3. Research - Life history & ecology2.2. Conservation Planning - Area-based Management Plan3.1. Monitoring - Population trends3.4. Monitoring - Habitat trends

Justification for research needed: Further research is needed into its ecology and life history in order to obtain information on population size, distribution and trends. It is necessary a monitoring plan for the invertebrate community in the habitat in order to contribute to the conservation of this species. Monitoring every ten years using the BALA protocol will inform about habitat quality (see e.g. Gaspar et al. 2011).

#### Viability analysis

Justification for probability: 

### Calacalles azoricus

#### Species information

Scientific name: Calacalles azoricus

Species authority: Stüben, 2004

Common names: True weevil, Snout beetle, Weevil (English); Gorgulho (Portuguese)

Kingdom: Animalia

Phylum: Arthropoda

Class: Insecta

Order: Coleoptera

Family: Curculionidae

Taxonomic notes: Species described by Stüben 2004 based on specimens found in Caladeira do Faial associated with roots of *Tolpis azorica*.

**Figure 9.**
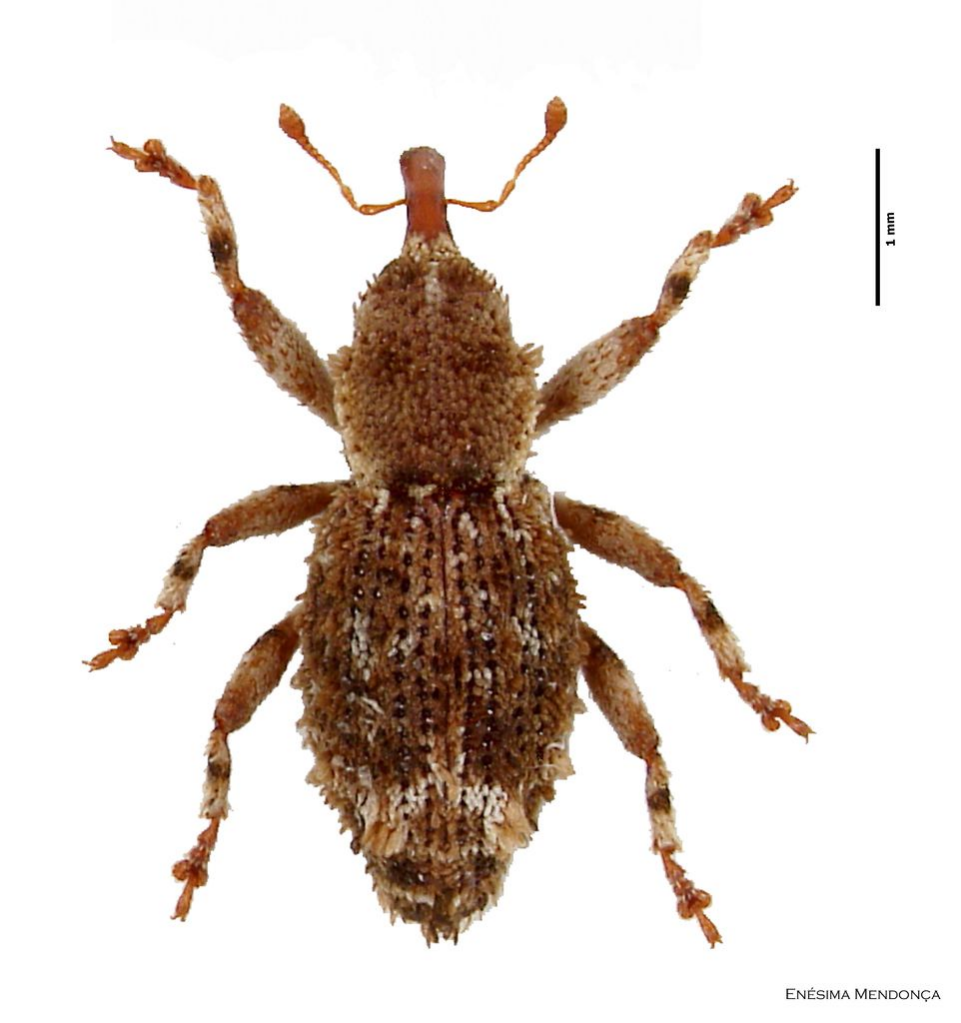
Female of *Calacalles azoricus* from Caldeira do Faial (Faial, Azores) (Credit: Enésima Mendonça).

Figure(s) or Photo(s): Fig. 9

Region for assessment: Global

#### Editor & Reviewers

##### Reviewers

Reviewers: Anja Danielczak

##### Editor

Editor: Axel Hochkirch

#### Reviewers

Reviewers: Anja Danielczak

#### Editor

Editor: Axel Hochkirch

#### Geographic range

Biogeographic realm: Palearctic

Countries: Portugal

Map of records (Google Earth): Suppl. material 15

Basis of EOO and AOO: Observed

Basis (narrative): *Calacalles azoricus* is a single-island endemic species from Faial (Azores, Portugal) (Borges et al. 2010), known from Natural Forest Reserve of Caldeira do Faial.

Min Elevation/Depth (m): 800

Max Elevation/Depth (m): 1000

Range description: The extent of occurrence (EOO) is 4 km^2^ and the maximum area of occupancy (AOO) is 4 km^2^.

#### New occurrences

#### Extent of occurrence

EOO (km2): 4

Trend: Decline (inferred)

Justification for trend: This species occurs in a small fragment of native forest in Faial island. The host plant is also very rare and decreasing its distribution due to invasive plants, that are changing the habitat structure, namely decreasing the cover of bryophytes and ferns in the soil and promoting the spread of other plants.

Causes ceased?: No

Causes understood?: Yes

Causes reversible?: Unknown

Extreme fluctuations?: Unknown

#### Area of occupancy

Trend: Decline (inferred)

Justification for trend: The species occurs in a small fragment of native forest of Faial island. The AOO with native forest is around 1.9 km². The species continues in decline due to native forest destruction and habitat fragmentation.

Causes ceased?: No

Causes understood?: Yes

Causes reversible?: Unknown

Extreme fluctuations?: Unknown

AOO (km2): 4

#### Locations

Number of locations: 1

Justification for number of locations: The species occurs only in Caldeira do Faial in the Faial island.

Trend: Decline (inferred)

Justification for trend: The species had a larger distribution in recent past, but major reductions in the distribution of the host plant (*Tolpis azorica*) due to major land-use changes in the last decades decreased the number of adequate patches of habitat. The single site is being heavily impacted by invasive plants in the last ten years.

Extreme fluctuations?: Unknown

#### Population

Trend: Decline (inferred)

Justification for trend: The species is very rare and only known from a single subpopulation in Faial island. A continuing decline in the number of mature individuals is inferred due to restricted distribution and host-plant rarity (*Tolpis azorica*).

Basis for decline: (c) a decline in area of occupancy, extent of occurrence and/or quality of habitat

Causes ceased?: No

Causes understood?: Yes

Causes reversible?: Unknown

Extreme fluctuations?: Unknown

#### Subpopulations

Number of subpopulations: 1

Trend: Decline (inferred)

Justification for trend: The species is very rare and only known from a single subpopulation in Faial island. A continuing decline in the number of supoputaions is inferred due to restricted distribution and host-plant rarity (*Tolpis azorica*), with potential extinction of the species.

Extreme fluctuations?: Unknown

Severe fragmentation?: No

#### Habitat

System: Terrestrial

Habitat specialist: Yes

Habitat (narrative): The species occurs in native forests of high altitude in the Faial island (Azores), with an altitudinal range between 800 and 1000 m.

Trend in extent, area or quality?: Decline (inferred)

Justification for trend: In the last 100 years, the species has probably strongly declined due to changes in habitat size and quality. Currently invasive plant species are also decreasing the quality of the remaining habitat

##### Habitat

Habitat importance: Major Importance

Habitats: 1.4. Forest - Temperate

#### Habitat

Habitat importance: Major Importance

Habitats: 1.4. Forest - Temperate

#### Ecology

Size: 0.35 -0.5 cm

Generation length (yr): 1

Dependency of single sp?: Yes

Dependent on species: *Tolpis azorica*

Ecology and traits (narrative): This is an univoltine species. Adults and larvae are herbivores and feed of plant tissues (*Tolpis azorica*).

#### Threats

Justification for threats: In the past, the species has probably strongly declined due to changes in habitat size and quality (Triantis et al. 2010, Terzopoulou et al. 2015). Currently invasive plants (*Hedychium gardnerianum* and *Rubus ulmifolius*) are changing some of the areas and decreasing the quality of the habitat, as they are changing the habitat structure, namely decreasing the cover of bryophytes and ferns in the soil and promoting the spread of other plants. Based on Ferreira et al. (2016) the habitat will further decline as a consequence of climate change (increasing number of droughts and habitat shifting & alteration).

##### Threats

Threat type: Ongoing

Threats: 8.1.2. Invasive and other problematic species, genes & diseases - Invasive non-native/alien species/diseases - Named species

##### Threats

Threat type: Future

Threats: 11.1. Climate change & severe weather - Habitat shifting & alteration11.2. Climate change & severe weather - Droughts

#### Threats

Threat type: Ongoing

Threats: 8.1.2. Invasive and other problematic species, genes & diseases - Invasive non-native/alien species/diseases - Named species

#### Threats

Threat type: Future

Threats: 11.1. Climate change & severe weather - Habitat shifting & alteration11.2. Climate change & severe weather - Droughts

#### Conservation

Justification for conservation actions: The species is protected by regional law (RAA 2012). Its habitat is in a regionally protected area (Natural Park of Faial). Degraded habitats should be restored in Caldeira do Faial and of critical importance is the removal of invasive non-native species where this is possible. A strategy needs to be developed to address the future threat by climate change. It is necessary a monitoring plan for the invertebrate community in the habitat in order to contribute to the conservation of this species. A habitat management plan is needed and anticipated to be developed during the coming years. Formal education and awareness is needed to allow future investments in restored habitats invaded by invasive plants.

##### Conservation actions

Conservation action type: In Place

Conservation actions: 1. Land/water protection1.1. Land/water protection - Site/area protection2.2. Land/water management - Invasive/problematic species control5.1. Law & policy - Legislation5.2. Law & policy - Policies and regulations

##### Conservation actions

Conservation action type: Needed

Conservation actions: 2.1. Land/water management - Site/area management2.2. Land/water management - Invasive/problematic species control2.3. Land/water management - Habitat & natural process restoration4. Education & awareness5.4.3. Law & policy - Compliance and enforcement - Sub-national level

#### Conservation actions

Conservation action type: In Place

Conservation actions: 1. Land/water protection1.1. Land/water protection - Site/area protection2.2. Land/water management - Invasive/problematic species control5.1. Law & policy - Legislation5.2. Law & policy - Policies and regulations

#### Conservation actions

Conservation action type: Needed

Conservation actions: 2.1. Land/water management - Site/area management2.2. Land/water management - Invasive/problematic species control2.3. Land/water management - Habitat & natural process restoration4. Education & awareness5.4.3. Law & policy - Compliance and enforcement - Sub-national level

#### Other

##### Use and trade

Use type: International

##### Ecosystem services

Ecosystem service type: Less important

Ecosystem services: 8. Habitat Maintenance

##### Research needed

Research needed: 1.2. Research - Population size, distribution & trends1.3. Research - Life history & ecology2.2. Conservation Planning - Area-based Management Plan3.1. Monitoring - Population trends3.4. Monitoring - Habitat trends

Justification for research needed: Further research is needed into its ecology and life history in order to find extant specimens and obtain information on population size, distribution and trends. It is also necessary an area-based management plan and a monitoring plan for the invertebrate community in the habitat in order to contribute to perform a species potential recovery plan. Monitoring every ten years using the BALA protocol will inform about habitat quality (see e.g. Gaspar et al. 2011).

#### Use and trade

Use type: International

#### Ecosystem services

Ecosystem service type: Less important

Ecosystem services: 8. Habitat Maintenance

#### Research needed

Research needed: 1.2. Research - Population size, distribution & trends1.3. Research - Life history & ecology2.2. Conservation Planning - Area-based Management Plan3.1. Monitoring - Population trends3.4. Monitoring - Habitat trends

Justification for research needed: Further research is needed into its ecology and life history in order to find extant specimens and obtain information on population size, distribution and trends. It is also necessary an area-based management plan and a monitoring plan for the invertebrate community in the habitat in order to contribute to perform a species potential recovery plan. Monitoring every ten years using the BALA protocol will inform about habitat quality (see e.g. Gaspar et al. 2011).

#### Viability analysis

Justification for probability: 

### Calacalles droueti

#### Species information

Scientific name: Calacalles droueti

Species authority: (Crotch, 1867)

Common names: True weevil, Snout beetle, Weevil (English); Gorgulho (Portuguese)

Kingdom: Animalia

Phylum: Arthropoda

Class: Insecta

Order: Coleoptera

Family: Curculionidae

Taxonomic notes: *Calacalles droueti* was originally described based on specimens collected by Godman in the island of Flores associated with stems of *Euphorbia stygiana*. More recently the Lectotypes were designated by Stüben 2004 based on specimens collected in Pico (Lagoa do Caiado) and Faial (Caldeira).

**Figure 10.**
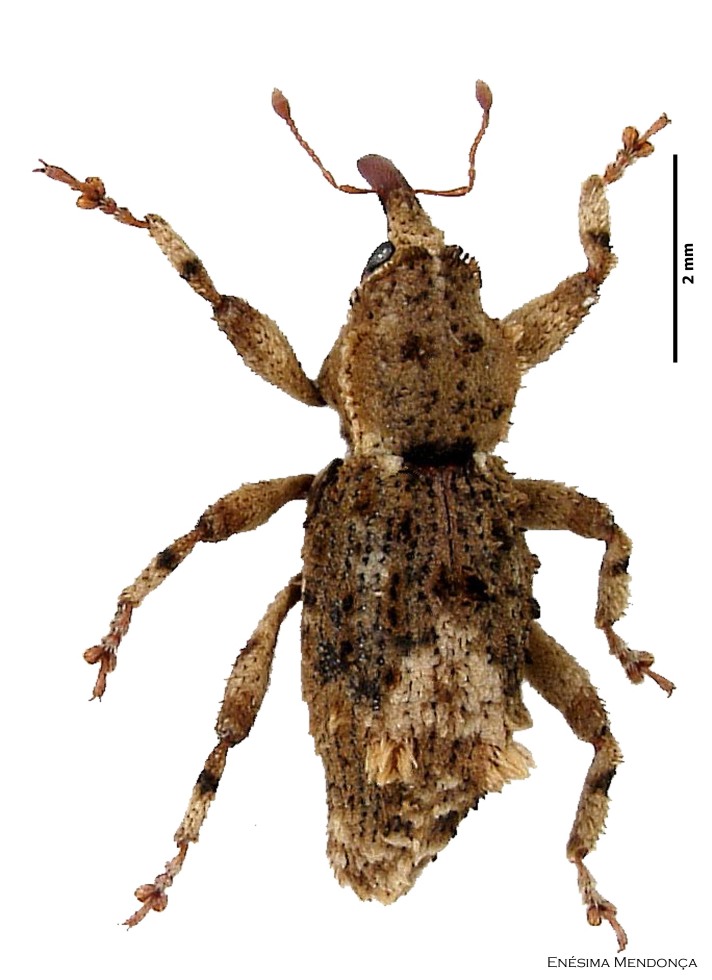
Female of *Calacalles droueti* from Lagoa do Caiado (Pico, Azores) (Credit: Enésima Mendonça).

Figure(s) or Photo(s): Fig. 10

Region for assessment: Global

#### Editor & Reviewers

##### Reviewers

Reviewers: Anja Danielczak

##### Editor

Editor: Axel Hochkirch

#### Reviewers

Reviewers: Anja Danielczak

#### Editor

Editor: Axel Hochkirch

#### Geographic range

Biogeographic realm: Palearctic

Countries: Portugal

Map of records (Google Earth): Suppl. material 16

Basis of EOO and AOO: Observed

Basis (narrative): *Calacalles droueti* is an endemic species present in Flores, Faial and Pico islands, but possibly extinct in Flores (Azores, Portugal) (Borges et al. 2010), known currently from Natural Forest Reserves of Caldeira do Faial (Faial) and Lagoa do Caiado (Pico).

Min Elevation/Depth (m): 600

Max Elevation/Depth (m): 1200

Range description: The extent of occurrence (EOO) is 706 km^2^ and the maximum area of occupancy (AOO) is 28 km^2^.

#### New occurrences

#### Extent of occurrence

EOO (km2): 706

Trend: Decline (observed)

Justification for trend: This species occurs in several areas of native forest of Faial and Pico islands. Possibly extinct in Flores. Most of the EOO includes habitats not occupied by this species that is restricted to few patches with the rare host plant (Euphorbia stygiana subsp. stygiana). The extent of occurrence of this species continues to decline due to habitat degradation in the native forest (mostly due to invasive plants) and to habitat fragmentation.

Causes ceased?: No

Causes understood?: Yes

Causes reversible?: Unknown

Extreme fluctuations?: Unknown

#### Area of occupancy

Trend: Decline (observed)

Justification for trend: The species occurs in several native forest patchs in Faial and Pico islands, but possibly extinct in Flores island. The area of occupancy of this species continues to decline due to habitat degradation in the native forest (mostly due to invasive plants) and to habitat fragmentation.

Causes ceased?: No

Causes understood?: Yes

Causes reversible?: Unknown

Extreme fluctuations?: Unknown

AOO (km2): 28

#### Locations

Number of locations: 7

Justification for number of locations: This species occurs in seven isolated native forest patches in the Faial and Pico islands. Possibly extinct in Flores.

Trend: Decline (inferred)

Justification for trend: Seven locations known but the original area was larger. Most of the sites have very few plants of Euphorbia stygiana subsp. stygiana, and the species can easily be extinct in some sites in near future.

Extreme fluctuations?: No

#### Population

Trend: Decline (inferred)

Justification for trend: The species is very rare and only known from a single subpopulation in Faial island and several subpopulations in Pico island. A continuing decline in the number of mature individuals is inferred due to host-plant rarity (Euphorbia stygiana subsp. stygiana). Most of the sites have very few plants of Euphorbia stygiana subsp. stygiana, and the species can easily be extinct in some sites in near future.

Basis for decline: (c) a decline in area of occupancy, extent of occurrence and/or quality of habitat

Causes ceased?: No

Causes understood?: Yes

Causes reversible?: Unknown

Extreme fluctuations?: Unknown

#### Subpopulations

Number of subpopulations: 7

Trend: Decline (inferred)

Justification for trend: The species is very rare, possibly extinct in Flores, known from a single subpopulation in Faial island and six subpopulations in Pico island. A continuing decline in the number of subpopulations is inferred due to host-plant rarity (Euphorbia stygiana subsp. stygiana). Decline is due to rarity and abundance decreasing of host plant.

Extreme fluctuations?: Unknown

Severe fragmentation?: Yes

Justification for fragmentation: Major land-use changes at middle and high elevations in Pico island promoted the creation of small patches of native forest. The species occurs in five natural forest fragments in Pico that are isolated in a sea of pastures and *Cryptomeria japonica* plantations. Most of the small fragments are not sustainable at long-term due to the spread of invasive plants. Possibly only the Lagoa do Caiado fragment is sustainable at long-term due to its larger size and larger populations of the host plant.

#### Habitat

System: Terrestrial

Habitat specialist: Yes

Habitat (narrative): The species occurs in native forests of high altitude in the Faial and Pico islands (Azores), with an altitudinal range between 600 and 1200 m.

Trend in extent, area or quality?: Decline (inferred)

Justification for trend: In the last 100 years, the species has probably strongly declined due to changes in habitat size and quality. Currently invasive plant species are also decreasing the quality of the remaining habitat. The host plant is very rare and has a declining trend (IUCN classification: Critically Endangered).

##### Habitat

Habitat importance: Major Importance

Habitats: 1.4. Forest - Temperate

#### Habitat

Habitat importance: Major Importance

Habitats: 1.4. Forest - Temperate

#### Ecology

Size: 0.4 - 0.6 cm

Generation length (yr): 1

Dependency of single sp?: Yes

Dependent on species: *Euphorbia stygiana stygiana*

Dependent on IUCN Status: Critically Endangered (CR)

Ecology and traits (narrative): This is an univoltine species. Adults and larvae are nocturnal herbivores and feed of plant tissues of Euphorbia stygiana subsp. stygiana.

#### Threats

Justification for threats: In the past, the species has probably strongly declined due to changes in habitat size and quality (Triantis et al. 2010, Terzopoulou et al. 2015). Currently, *Cryptomeria japonica* wood & pulp plantations management and invasive plants (*Hedychium gardnerianum*) are changing some of the areas and decreasing the quality of the habitat, since are changing the habitat structure, namely decreasing the cover of bryophytes and ferns in the soil and promoting the spread of other plants. Based on Ferreira et al. (2016) the habitat will further decline as a consequence of climate change (increasing number of droughts and habitat shifting & alteration). Other important threat is the extreme rarity of the host plant.

##### Threats

Threat type: Ongoing

Threats: 2.2.1. Agriculture & aquaculture - Wood & pulp plantations - Small-holder plantations8.1.2. Invasive and other problematic species, genes & diseases - Invasive non-native/alien species/diseases - Named species12. Other options - Other threat

##### Threats

Threat type: Future

Threats: 11.1. Climate change & severe weather - Habitat shifting & alteration11.2. Climate change & severe weather - Droughts

#### Threats

Threat type: Ongoing

Threats: 2.2.1. Agriculture & aquaculture - Wood & pulp plantations - Small-holder plantations8.1.2. Invasive and other problematic species, genes & diseases - Invasive non-native/alien species/diseases - Named species12. Other options - Other threat

#### Threats

Threat type: Future

Threats: 11.1. Climate change & severe weather - Habitat shifting & alteration11.2. Climate change & severe weather - Droughts

#### Conservation

Justification for conservation actions: The species is protected by regional law (RAA 2012). Its habitat is in regionally protected areas (Natural Parks of Faial and Pico). It is necessary a monitoring plan for the invertebrate community in the habitat in order to contribute to the conservation of this species. A habitat management plan is needed and anticipated to be developed during the coming years. The conservation of the host plant is critical. Since this species occurs in relict native Azorean forests and associated also with a very rare plant, it is suggested that some awareness measures should be put in practice.

##### Conservation actions

Conservation action type: In Place

Conservation actions: 1. Land/water protection1.1. Land/water protection - Site/area protection2. Land/water management2.2. Land/water management - Invasive/problematic species control4. Education & awareness5.1. Law & policy - Legislation5.2. Law & policy - Policies and regulations

##### Conservation actions

Conservation action type: Needed

Conservation actions: 2.1. Land/water management - Site/area management2.2. Land/water management - Invasive/problematic species control2.3. Land/water management - Habitat & natural process restoration4. Education & awareness5.4.3. Law & policy - Compliance and enforcement - Sub-national level

#### Conservation actions

Conservation action type: In Place

Conservation actions: 1. Land/water protection1.1. Land/water protection - Site/area protection2. Land/water management2.2. Land/water management - Invasive/problematic species control4. Education & awareness5.1. Law & policy - Legislation5.2. Law & policy - Policies and regulations

#### Conservation actions

Conservation action type: Needed

Conservation actions: 2.1. Land/water management - Site/area management2.2. Land/water management - Invasive/problematic species control2.3. Land/water management - Habitat & natural process restoration4. Education & awareness5.4.3. Law & policy - Compliance and enforcement - Sub-national level

#### Other

Justification for use and trade: This species is not utilized.

Justification for ecosystem services : There is insufficient information available to identify the ecosystem services for this species.

##### Use and trade

Use type: International

##### Ecosystem services

Ecosystem service type: Less important

##### Research needed

Research needed: 1.2. Research - Population size, distribution & trends1.3. Research - Life history & ecology2.2. Conservation Planning - Area-based Management Plan3.1. Monitoring - Population trends3.4. Monitoring - Habitat trends

Justification for research needed: Further research is needed into its ecology and life history in order to find extant specimens in other sites with the host plant, and obtain information on population size, distribution and trends. It is also necessary an area-based management plan and a monitoring plan for the invertebrate community in the habitat in order to contribute to perform a species potential recovery plan. Monitoring every ten years using the BALA protocol will inform about habitat quality (see e.g. Gaspar et al. 2011).

#### Use and trade

Use type: International

#### Ecosystem services

Ecosystem service type: Less important

#### Research needed

Research needed: 1.2. Research - Population size, distribution & trends1.3. Research - Life history & ecology2.2. Conservation Planning - Area-based Management Plan3.1. Monitoring - Population trends3.4. Monitoring - Habitat trends

Justification for research needed: Further research is needed into its ecology and life history in order to find extant specimens in other sites with the host plant, and obtain information on population size, distribution and trends. It is also necessary an area-based management plan and a monitoring plan for the invertebrate community in the habitat in order to contribute to perform a species potential recovery plan. Monitoring every ten years using the BALA protocol will inform about habitat quality (see e.g. Gaspar et al. 2011).

#### Viability analysis

Justification for probability: 

### Calacalles subcarinatus

#### Species information

Scientific name: Calacalles subcarinatus

Species authority: (Israelson, 1984)

Synonyms: *Acalles subcarinatus* Israelson, 1984; *Acalles wollastoni* Chevr., 1852

Common names: True weevil, Snout beetle, Weevil (English); Gorgulho (Portuguese)

Kingdom: Animalia

Phylum: Arthropoda

Class: Insecta

Order: Coleoptera

Family: Curculionidae

Taxonomic notes: This small species was described from a individuals collected in Santa Maria (Pico Alto) and S. Miguel (Furnas) islands, between 8.VII.1982 and 13.VII.1982. These specimens are deposited in the Finnish Museum of Natural History (Israelson 1984b).

**Figure 11.**
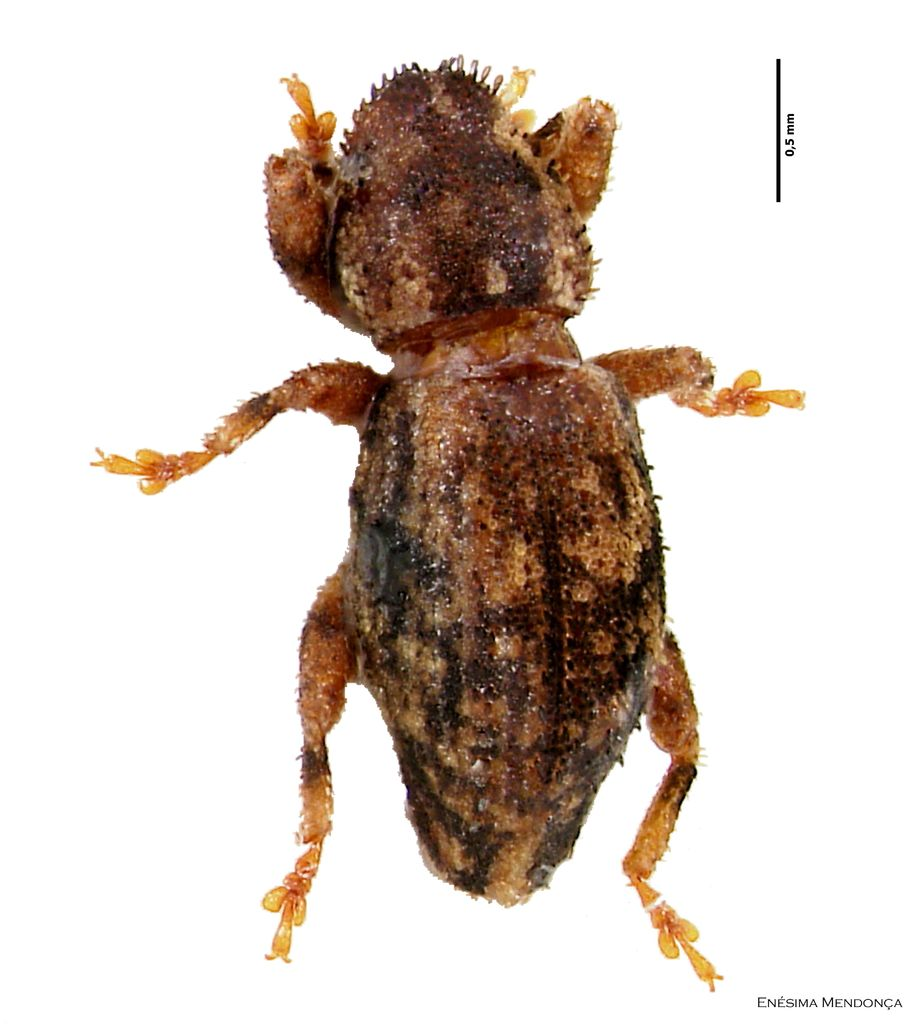
Female of *Calacalles subcarinatus* from Terra Brava (Terceira, Azores) (Credit: Enésima Mendonça).

**Figure 12.**
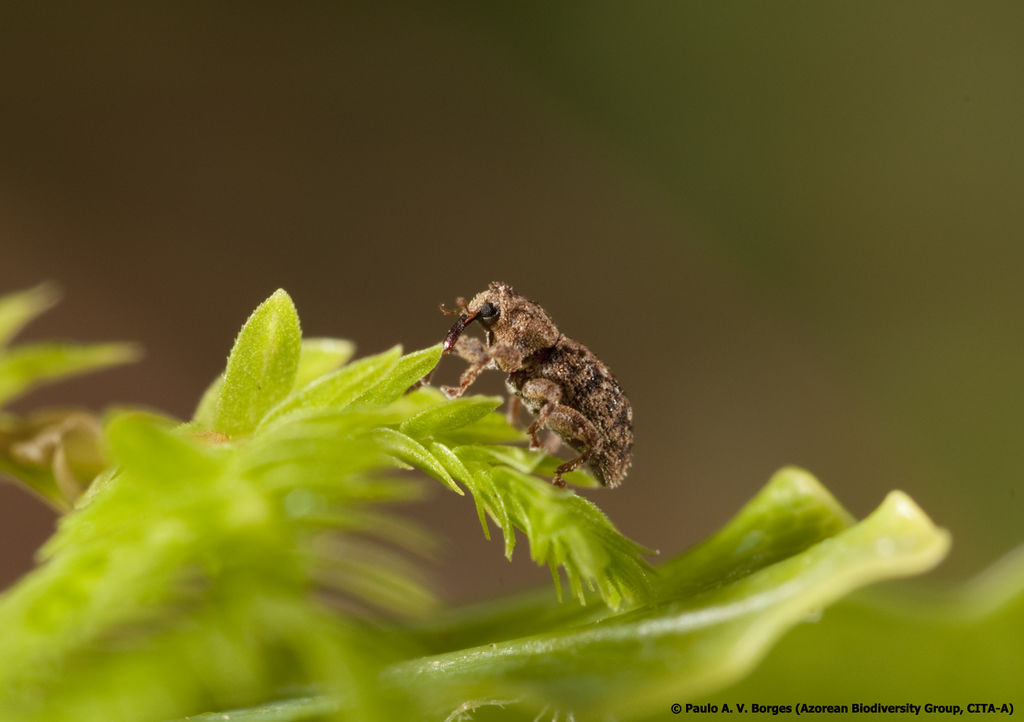
Female of *Calacalles subcarinatus* from Terra Brava (Terceira, Azores) (Credit: Paulo A.V. Borges).

Figure(s) or Photo(s): Figs 11, 12

Region for assessment: Global

#### Editor & Reviewers

##### Reviewers

Reviewers: Anja Danielczak

##### Editor

Editor: Axel Hochkirch

#### Reviewers

Reviewers: Anja Danielczak

#### Editor

Editor: Axel Hochkirch

#### Geographic range

Biogeographic realm: Palearctic

Countries: Portugal

Map of records (Google Earth): Suppl. material 17

Basis of EOO and AOO: Observed

Basis (narrative): *Calacalles subcarinatus* is a widespread endemic species present in all islands of the Azorean archipelago (Azores, Portugal) (Borges et al. 2010), known from Natural Forest Reserves of Caldeiras Funda e Rasa and Morro Alto e Pico da Sé (Flores); Caldeira do Faial (Faial); Mistério da Prainha (Pico); Pico Pinheiro and Topo (S. Jorge); Biscoito da Ferraria, Pico Galhardo, Serra Sta. Bárbara and Terra Brava (Terceira); Atalhada e Pico da Vara (S. Miguel) and Pico Alto (Santa Maria).

Min Elevation/Depth (m): 100

Max Elevation/Depth (m): 1200

Range description: The extent of occurrence (EOO) is ca 42,600 km^2^ and the maximum area of occupancy (AOO) is 220 km^2^.

#### New occurrences

#### Extent of occurrence

EOO (km2): 42,600

Trend: Increase

Justification for trend: The species is expanding its range to exotic trees and man made habitats.

Causes ceased?: No

Causes understood?: Yes

Causes reversible?: Unknown

Extreme fluctuations?: No

#### Area of occupancy

Trend: Increase

Justification for trend: The species is expanding its range to exotic trees and man made habitats. The species is expanding to non-native habitats.

Causes ceased?: No

Causes understood?: Yes

Causes reversible?: Unknown

Extreme fluctuations?: No

AOO (km2): 220

#### Locations

Number of locations: 0

Justification for number of locations: This is a widespread species, with no threats.

Trend: Increase

Justification for trend: Possibly is increasing distribution due to adaptation to exotic trees, namely low altitude orchards.

Extreme fluctuations?: No

#### Population

Trend: Increase

Justification for trend: *C. subcarinatus* is a widespread and highly abundant species. The species is expanding to exotic habitats and population is increasing. We assume no impact for the population as it occurs naturally in several native and exotic patches in all islands of the archipelago.

Basis for decline: (a) direct observation

Causes ceased?: No

Causes understood?: Yes

Causes reversible?: Unknown

Extreme fluctuations?: No

#### Subpopulations

Number of subpopulations: 9

Trend: Stable

Justification for trend: *C. subcarinatus* is a widespread and highly abundant species. The species presents a stable population and occurs in several islands. We assume no impact for the subpopulations as it occurs naturally in several patches in all islands of the arquipelago.

Extreme fluctuations?: Unknown

Severe fragmentation?: No

#### Habitat

System: Terrestrial

Habitat specialist: No

Habitat (narrative): The species occurs in several habitats and in all islands of the Azorean arquipelago, with an altitudinal range between 100 and 1200 m. *C. subcarinatus* inhabits the native forests dominated by native and endemic vegetation, prefering Ilex perado subsp. azorica but also occurring in *Juniperus brevifolia*, *Frangula azorica*, *Vaccinium cylindraceum* and *Erica azorica*; exotic forests (mainly plantations and forests of *Pittosporum* spp. and *Eucalyptus* spp.); in agricultural areas occurs in orchards associated with *Castanea sativa*.

Trend in extent, area or quality?: Increase

Justification for trend: The species is expanding to exotic trees

##### Habitat

Habitat importance: Suitable

Habitats: 1.4. Forest - Temperate

#### Habitat

Habitat importance: Suitable

Habitats: 1.4. Forest - Temperate

#### Ecology

Size: 0.17 - 0.26 cm

Generation length (yr): 1

Dependency of single sp?: No

Ecology and traits (narrative): Adults and larvae are herbivores and feed of plant tissues both during the day and night. Based on seasonal data from SLAM traps obtained in several islands between 2012 and 2016 (Borges et al. 2017b), the adults are active all year, being most abundant in spring and summer.

#### Threats

Justification for threats: In the past, the species has probably strongly declined due to changes in habitat size and quality (Triantis et al. 2010; Terzopoulou et al. 2015). However, the species seems to be adapting to other non-native trees and is expanding its range. No threats are known for this species, but possibly invasive species may create an impact in future.

##### Threats

Threat type: Future

Threats: 8.1. Invasive and other problematic species, genes & diseases - Invasive non-native/alien species/diseases

#### Threats

Threat type: Future

Threats: 8.1. Invasive and other problematic species, genes & diseases - Invasive non-native/alien species/diseases

#### Conservation

Justification for conservation actions: The species is not protected by regional law. Its habitat is in regionally protected areas (Natural Parks of Flores, Faial, Pico, S. Jorge, Terceira, Graciosa, S. Miguel and Santa Maria). No special measures of conservation are needed since the species also occurs in non-native plants.

##### Conservation actions

Conservation action type: In Place

#### Conservation actions

Conservation action type: In Place

#### Other

Justification for use and trade: The species is not utilized.

##### Use and trade

Use type: International

##### Ecosystem services

Ecosystem service type: Important

Ecosystem services: 8. Habitat Maintenance

##### Research needed

Research needed: 1.2. Research - Population size, distribution & trends1.3. Research - Life history & ecology3.1. Monitoring - Population trends3.4. Monitoring - Habitat trends

Justification for research needed: Further research is needed into its ecology and life history in order to obtain information on population size, distribution and trends. A monitoring every ten years using the BALA protocol will inform about habitat quality (see e.g. Gaspar et al. 2011)

#### Use and trade

Use type: International

#### Ecosystem services

Ecosystem service type: Important

Ecosystem services: 8. Habitat Maintenance

#### Research needed

Research needed: 1.2. Research - Population size, distribution & trends1.3. Research - Life history & ecology3.1. Monitoring - Population trends3.4. Monitoring - Habitat trends

Justification for research needed: Further research is needed into its ecology and life history in order to obtain information on population size, distribution and trends. A monitoring every ten years using the BALA protocol will inform about habitat quality (see e.g. Gaspar et al. 2011)

#### Viability analysis

Justification for probability: 

### Caulotrupis parvus

#### Species information

Scientific name: Caulotrupis parvus

Species authority: Israelson, 1985

Common names: True weevil, Snout beetle, Weevil (English); Gorgulho (Portuguese)

Kingdom: Animalia

Phylum: Arthropoda

Class: Insecta

Order: Coleoptera

Family: Curculionidae

Taxonomic notes: *Caulotrupis parvus* was described from two individuals collected in Santa Maria island (Pico Alto), between 11.VII.1982 and 8.VIII.1983, deposited in G. Israelson collection. The particularities of the aedeagal structures may indicate a more profund difference between *C. parvus* and the Madeiran forms (Israelson 1985a).

**Figure 13.**
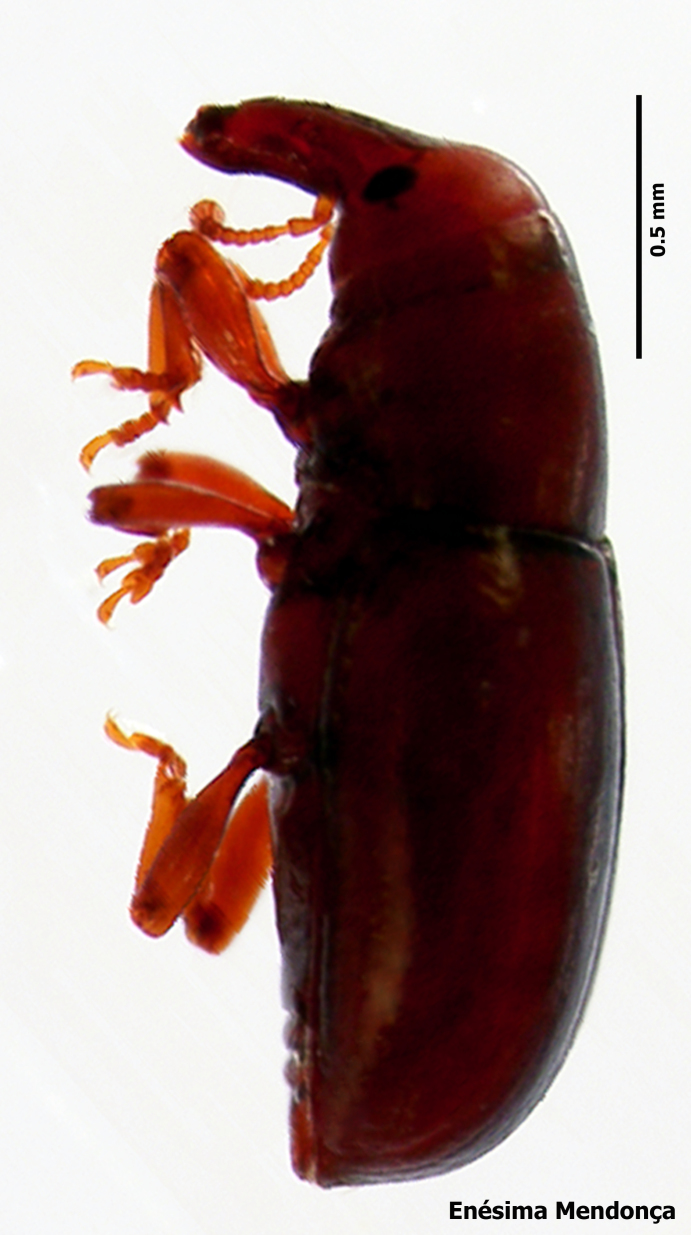
*Caulotrupis parvus* from Pico Alto (Santa Maria, Azores) (Credit: Enésima Mendonça).

Figure(s) or Photo(s): Fig. 13

Region for assessment: Global

#### Editor & Reviewers

##### Reviewers

Reviewers: Anja Danielczak

##### Editor

Editor: Axel Hochkirch

#### Reviewers

Reviewers: Anja Danielczak

#### Editor

Editor: Axel Hochkirch

#### Geographic range

Biogeographic realm: Palearctic

Countries: Portugal

Map of records (Google Earth): Suppl. material 18

Basis of EOO and AOO: Observed

Basis (narrative): The area of its remaining native habitat is 0.09 km², but the AOO is 8 km². Its extent of occurrence (EOO) is therefore also 8 km².

Min Elevation/Depth (m): 310

Max Elevation/Depth (m): 550

Range description: *Caulotrupis parvus* is a single-island endemic species from Santa Maria (Azores, Portugal) (Borges et al. 2010), where it is restricted to the highest elevations of the island (310 to 550 m asl) and to the unique native primary forest. However the species was also found recently by Meijer et al. 2011 in a *Cryptomeria japonica* plantation, prone to be cut soon.

#### New occurrences

#### Extent of occurrence

EOO (km2): 8

Trend: Decline (inferred)

Justification for trend: This species occurs in a native forest patch and in a small fragment of exotic forest in Santa Maria island. There is an inferred continuing decline in EOO due to the spread of invasive plants and observed loss of habitat quality. In addition one of the sites is an industrial *Cryptomeria japonica* plantation, prone to be cut soon, which will imply a reduction of EOO to half.

Causes ceased?: No

Causes understood?: Yes

Causes reversible?: Unknown

Extreme fluctuations?: Unknown

#### Area of occupancy

Trend: Decline (inferred)

Justification for trend: There is an inferred continuing decline in AOO due to the spread of invasive plants and observed loss of habitat quality. In addition one of the localities is an industrial *Cryptomeria japonica* plantation, prone to be cut soon.

Causes ceased?: No

Causes understood?: Yes

Causes reversible?: Unknown

Extreme fluctuations?: Unknown

AOO (km2): 8

#### Locations

Number of locations: 2

Justification for number of locations: The main native complete forest is threatened by invasive plants. The additional location is a pulp *Cryptomeria japonica* plantation that will be cut soon.

Trend: Decline (observed)

Justification for trend: Only one location of native forest left, that has a very low Index of Biotic Integrity (Gaspar et al. 2011) with a size of 0.09 km² and invasive plants that can drive this species to extinction very fast. The other location is a *Cryptomeria japonica* plantation that will be cut soon.

Extreme fluctuations?: Unknown

#### Population

Trend: Decline (observed)

Justification for trend: The species is very rare and only known from a single sustainable subpopulation (the species occurs in a single native forest patch included in a Natural Reserve of Santa Maria island that has a very low Index of Biotic Integrity, Gaspar et al. 2011)), since the second known location is a *Cryptomeria japonica* plantation threaten by deforestation. A continuing decline in the number of mature individuals is inferred from the ongoing habitat degradation due to invasions of alien plants, and deforestation of pulp plantation of *Cryptomeria japonica*.

Basis for decline: (c) a decline in area of occupancy, extent of occurrence and/or quality of habitat

Causes ceased?: No

Causes understood?: Yes

Causes reversible?: Unknown

Extreme fluctuations?: Unknown

#### Subpopulations

Number of subpopulations: 2

Trend: Decline (inferred)

Justification for trend: The species is very rare and only known from a single sustainable subpopulation, since the second known location is a *Cryptomeria japonica* plantation threatened by deforestation. A continuing decline in the number of subpopulations is inferred from the ongoing habitat degradation due to invasions of alien plants, and deforestation of production pulp plantation of *Cryptomeria japonica*.

Extreme fluctuations?: Unknown

Severe fragmentation?: Yes

Justification for fragmentation: Major land-use changes at all elevations in Santa Maria island promoted the creation of small patches of native and exotic forest. The species occurs in one natural forest fragment and in a small patch of exotic forest that are both isolated in a sea of pastures and *Cryptomeria japonica* plantations. The subpopulation of the exotic plantation is not sustainable in the next ten years and the single native forest patch is included the Natural Reserve of Santa Maria island that has a very low Index of Biotic Integrity (Gaspar et al. 2011).

#### Habitat

System: Terrestrial

Habitat specialist: Yes

Habitat (narrative): The species occurs in the native forests of the Azores, surrounded by plantations of exotic trees and pastures, but also found in a *Cryptomeria japonica* plantation. This species has an altitudinal range between 310 and 550 m.

Trend in extent, area or quality?: Decline (observed)

Justification for trend: Ongoing invasion of exotic plants that are changing the structure of the forest and the cover of bryophytes and ferns in the soil with impacts on native invertebrate species.

##### Habitat

Habitat importance: Major Importance

Habitats: 1.4. Forest - Temperate16. Introduced vegetation

#### Habitat

Habitat importance: Major Importance

Habitats: 1.4. Forest - Temperate16. Introduced vegetation

#### Ecology

Size: 0.23 cm

Generation length (yr): 1

Dependency of single sp?: No

Ecology and traits (narrative): This is an univoltine species. It feeds on dead wood.

#### Threats

Justification for threats: In the past, the species has probably strongly declined due to deforestation. The most important ongoing threats to this species are *Cryptomeria japonica* wood & pulp plantations management and the spread of invasive plants (*Hedychium gardnerianum* and *Pittosporum undulatum*) that are changing the habitat structure in the main native forest, namely decreasing the cover of bryophytes and ferns in the soil and promoting the spread of other plants. Based on Ferreira et al. 2016 the habitat will decline as a consequence of climate change (increasing number of droughts), which may drive this species to extinction, because it is depending on humid forests.

##### Threats

Threat type: Ongoing

Threats: 2.2.1. Agriculture & aquaculture - Wood & pulp plantations - Small-holder plantations2.3.2. Agriculture & aquaculture - Livestock farming & ranching - Small-holder grazing, ranching or farming8.1.2. Invasive and other problematic species, genes & diseases - Invasive non-native/alien species/diseases - Named species

##### Threats

Threat type: Future

Threats: 11.1. Climate change & severe weather - Habitat shifting & alteration11.2. Climate change & severe weather - Droughts

#### Threats

Threat type: Ongoing

Threats: 2.2.1. Agriculture & aquaculture - Wood & pulp plantations - Small-holder plantations2.3.2. Agriculture & aquaculture - Livestock farming & ranching - Small-holder grazing, ranching or farming8.1.2. Invasive and other problematic species, genes & diseases - Invasive non-native/alien species/diseases - Named species

#### Threats

Threat type: Future

Threats: 11.1. Climate change & severe weather - Habitat shifting & alteration11.2. Climate change & severe weather - Droughts

#### Conservation

Justification for conservation actions: The species is protected by regional law (RAA 2012). Its habitat is in a regionally protected area (Santa Maria Natural Park). The Santa Maria Natural Park administration is currently starting control measures of the invasive plants. Further spread of invasive plants needs to be stopped in order to avoid any future declines of the species. Degraded habitats should be restored and a strategy needs to be developed to address the future threat by climate change. A general monitoring scheme for the invertebrate community in the habitat is in place, but the population of this particular species and its habitat needs to be monitored in more detail. A habitat management plan is needed and anticipated to be developed during the coming years.

##### Conservation actions

Conservation action type: In Place

Conservation actions: 1.1. Land/water protection - Site/area protection1.2. Land/water protection - Resource & habitat protection2.2. Land/water management - Invasive/problematic species control5.1. Law & policy - Legislation5.2. Law & policy - Policies and regulations

##### Conservation actions

Conservation action type: Needed

Conservation actions: 2.1. Land/water management - Site/area management2.2. Land/water management - Invasive/problematic species control2.3. Land/water management - Habitat & natural process restoration4. Education & awareness5.4.3. Law & policy - Compliance and enforcement - Sub-national level

#### Conservation actions

Conservation action type: In Place

Conservation actions: 1.1. Land/water protection - Site/area protection1.2. Land/water protection - Resource & habitat protection2.2. Land/water management - Invasive/problematic species control5.1. Law & policy - Legislation5.2. Law & policy - Policies and regulations

#### Conservation actions

Conservation action type: Needed

Conservation actions: 2.1. Land/water management - Site/area management2.2. Land/water management - Invasive/problematic species control2.3. Land/water management - Habitat & natural process restoration4. Education & awareness5.4.3. Law & policy - Compliance and enforcement - Sub-national level

#### Other

##### Use and trade

Use type: International

##### Ecosystem services

Ecosystem service type: Less important

Ecosystem services: 8. Habitat Maintenance

##### Research needed

Research needed: 1.2. Research - Population size, distribution & trends1.3. Research - Life history & ecology2.2. Conservation Planning - Area-based Management Plan3.1. Monitoring - Population trends3.4. Monitoring - Habitat trends

Justification for research needed: Further research is needed into its ecology and life history in order to find extant specimens and obtain information on population size, distribution and trends. It is also necessary an area-based management plan and a monitoring plan for the invertebrate community in the Pico Alto native forest and surrounded areas of non-native habitat in order to contribute to perform a species potential recovery plan. Monitoring every ten years using the BALA protocol will inform about habitat quality (see e.g. Gaspar et al. 2011).

#### Use and trade

Use type: International

#### Ecosystem services

Ecosystem service type: Less important

Ecosystem services: 8. Habitat Maintenance

#### Research needed

Research needed: 1.2. Research - Population size, distribution & trends1.3. Research - Life history & ecology2.2. Conservation Planning - Area-based Management Plan3.1. Monitoring - Population trends3.4. Monitoring - Habitat trends

Justification for research needed: Further research is needed into its ecology and life history in order to find extant specimens and obtain information on population size, distribution and trends. It is also necessary an area-based management plan and a monitoring plan for the invertebrate community in the Pico Alto native forest and surrounded areas of non-native habitat in order to contribute to perform a species potential recovery plan. Monitoring every ten years using the BALA protocol will inform about habitat quality (see e.g. Gaspar et al. 2011).

#### Viability analysis

Justification for probability: 

### Donus multifidus

#### Species information

Scientific name: Donus multifidus

Species authority: (Israelson, 1984)

Synonyms: *Hypera multifida* Israelson, 1984

Common names: True weevil, Snout beetle, Weevil (English); Gorgulho (Portuguese)

Kingdom: Animalia

Phylum: Arthropoda

Class: Insecta

Order: Coleoptera

Family: Curculiniodae

Taxonomic notes: *Donus multifidus* was described from four individuals collected in Santa Maria island (Pico Alto), between 8.VII.1982 and 4.VIII.1983, deposited in G. Israelson collection (Israelson 1984b).

**Figure 14.**
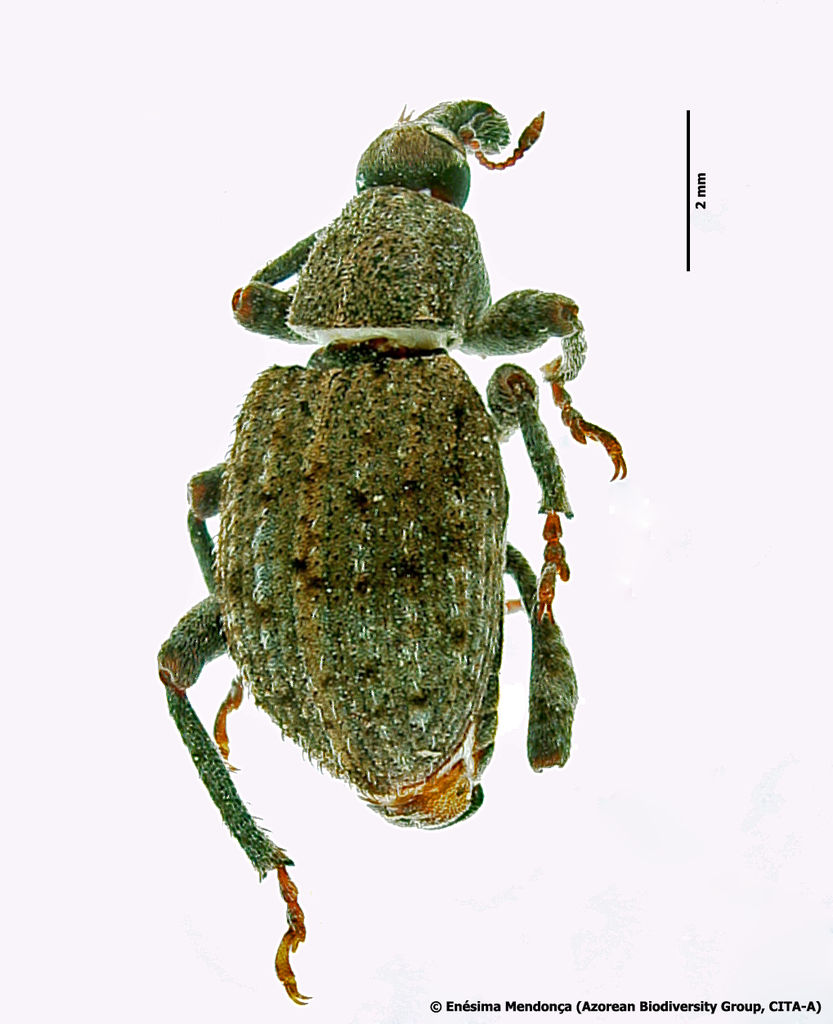
*Donus multifidus* from Pico Alto (Santa Maria, Azores) (Credit: Enésima Mendonça).

Figure(s) or Photo(s): Fig. 14

Region for assessment: Global

#### Editor & Reviewers

##### Reviewers

Reviewers: Anja Danielczak

##### Editor

Editor: Axel Hochkirch

#### Reviewers

Reviewers: Anja Danielczak

#### Editor

Editor: Axel Hochkirch

#### Geographic range

Biogeographic realm: Palearctic

Countries: Portugal

Map of records (Google Earth): Suppl. material 19

Basis of EOO and AOO: Observed

Basis (narrative): The area of its remaining native habitat is 0.09 km², but the AOO is 4 km². Its extent of occurrence (EOO) is therefore also 4 km².

Min Elevation/Depth (m): 450

Max Elevation/Depth (m): 550

Range description: *Donus multifidus* is a single-island endemic species from Santa Maria (Azores, Portugal) (Borges et al. 2010), where it is restricted to the highest elevations of the island (450 to 550 m asl) and to the unique native forest remnant included in a Natural Reserve of Santa Maria island that has a very low Index of Biotic Integrity (Gaspar et al. 2011).

#### New occurrences

#### Extent of occurrence

EOO (km2): 4

Trend: Decline (inferred)

Justification for trend: This species occurs in a single native forest patch of Santa Maria island. There is an inferred continuing decline in EOO due to the spread of invasive plants and observed loss of habitat quality.

Causes ceased?: No

Causes understood?: Yes

Causes reversible?: Unknown

Extreme fluctuations?: Unknown

#### Area of occupancy

Trend: Decline (inferred)

Justification for trend: This species occurs in a small fragment of native forest in Santa Maria island. The size of its remaining native habitat is now only around 0.09 km². The species continues in decline due to native forest destruction and habitat fragmentation.

Causes ceased?: No

Causes understood?: Yes

Causes reversible?: Unknown

Extreme fluctuations?: Unknown

AOO (km2): 4

#### Locations

Number of locations: 1

Justification for number of locations: The species occurs in a single native forest patch in the Santa Maria island.

Trend: Stable

Justification for trend: The species occurs in a single native forest patch included in a Natural Reserve of Santa Maria island that has a very low Index of Biotic Integrity (Gaspar et al. 2011).

Extreme fluctuations?: Unknown

#### Population

Trend: Decline (inferred)

Justification for trend: The species is very rare and only known from a single sustainable subpopulation. A continuing decline in the number of mature individuals is inferred from the ongoing habitat degradation due to invasions of alien plants (e.g. *Hedychium gardnerianum*; *Pittosporum undulatum*), that are changing the structure of the forest and the cover of bryophytes and ferns in the soil decreasing the quality of the habitat with impacts on the species.

Basis for decline: (c) a decline in area of occupancy, extent of occurrence and/or quality of habitat

Causes ceased?: No

Causes understood?: Yes

Causes reversible?: Unknown

Extreme fluctuations?: Unknown

#### Subpopulations

Number of subpopulations: 1

Trend: Stable

Justification for trend: The species is very rare and only known from a single subpopulation.

Extreme fluctuations?: Unknown

Severe fragmentation?: No

#### Habitat

System: Terrestrial

Habitat specialist: Yes

Habitat (narrative): The species occurs in native forests of high altitude in Santa Maria island (Azores), with an altitudinal range between 450 and 550 m.

Trend in extent, area or quality?: Decline (observed)

Justification for trend: Due to invasive plant species that are changing the structure of the forest and the cover of bryophytes and ferns in the soil with impacts on the species.

##### Habitat

Habitat importance: Major Importance

Habitats: 1.4. Forest - Temperate

#### Habitat

Habitat importance: Major Importance

Habitats: 1.4. Forest - Temperate

#### Ecology

Generation length (yr): 1

Dependency of single sp?: No

Ecology and traits (narrative): Adults and larvae are herbivores and feed of plant tissues. This is an univoltine species.

#### Threats

Justification for threats: In the past, the species has probably strongly declined due to deforestation. The most important ongoing threats to this species are *Cryptomeria japonica* wood & pulp plantations management and the spread of invasive plants (*Hedychium gardnerianum* and *Pittosporum undulatum*) that are changing the habitat structure in the main native forest, namely decreasing the cover of bryophytes and ferns in the soil and promoting the spread of other plants. Based on Ferreira et al. 2016 the habitat will decline as a consequence of climate change (increasing number of droughts), which may drive this species to extinction, because it is dependend on humid forests.

##### Threats

Threat type: Ongoing

Threats: 2.2.1. Agriculture & aquaculture - Wood & pulp plantations - Small-holder plantations8.1.2. Invasive and other problematic species, genes & diseases - Invasive non-native/alien species/diseases - Named species

##### Threats

Threat type: Future

Threats: 11.1. Climate change & severe weather - Habitat shifting & alteration11.2. Climate change & severe weather - Droughts

#### Threats

Threat type: Ongoing

Threats: 2.2.1. Agriculture & aquaculture - Wood & pulp plantations - Small-holder plantations8.1.2. Invasive and other problematic species, genes & diseases - Invasive non-native/alien species/diseases - Named species

#### Threats

Threat type: Future

Threats: 11.1. Climate change & severe weather - Habitat shifting & alteration11.2. Climate change & severe weather - Droughts

#### Conservation

Justification for conservation actions: The species is protected by regional law (RAA 2012). Its habitat is in a regionally protected area (Natural Park of Santa Maria). Further spread of invasive plants needs to be stopped in order to avoid any future decline of the species. Degraded habitats should be restored and a strategy needs to be developed to address the future threat by climate change. It is necessary a monitoring plan for the invertebrate community in the habitat in order to contribute to the conservation of this species. A habitat management plan is needed and anticipated to be developed during the coming years.

##### Conservation actions

Conservation action type: In Place

Conservation actions: 1. Land/water protection1.1. Land/water protection - Site/area protection2.1. Land/water management - Site/area management5. Law & policy5.1. Law & policy - Legislation5.2. Law & policy - Policies and regulations

##### Conservation actions

Conservation action type: Needed

Conservation actions: 2.1. Land/water management - Site/area management2.2. Land/water management - Invasive/problematic species control2.3. Land/water management - Habitat & natural process restoration4. Education & awareness5.4.3. Law & policy - Compliance and enforcement - Sub-national level

#### Conservation actions

Conservation action type: In Place

Conservation actions: 1. Land/water protection1.1. Land/water protection - Site/area protection2.1. Land/water management - Site/area management5. Law & policy5.1. Law & policy - Legislation5.2. Law & policy - Policies and regulations

#### Conservation actions

Conservation action type: Needed

Conservation actions: 2.1. Land/water management - Site/area management2.2. Land/water management - Invasive/problematic species control2.3. Land/water management - Habitat & natural process restoration4. Education & awareness5.4.3. Law & policy - Compliance and enforcement - Sub-national level

#### Other

##### Use and trade

Use type: International

##### Ecosystem services

Ecosystem service type: Less important

Ecosystem services: 8. Habitat Maintenance

##### Research needed

Research needed: 1.2. Research - Population size, distribution & trends1.3. Research - Life history & ecology2.2. Conservation Planning - Area-based Management Plan3.1. Monitoring - Population trends3.4. Monitoring - Habitat trends

Justification for research needed: Further research is needed into its ecology and life history in order to find extant specimens in the Pico Alto region (including non-native habitats) and obtain information on population size, distribution and trends. It is also necessary an area-based management plan and a monitoring plan for the invertebrate community in the habitat in order to contribute to perform a species potential recovery plan. Monitoring every ten years using the BALA protocol will inform about habitat quality (see e.g. Gaspar et al. 2011).

#### Use and trade

Use type: International

#### Ecosystem services

Ecosystem service type: Less important

Ecosystem services: 8. Habitat Maintenance

#### Research needed

Research needed: 1.2. Research - Population size, distribution & trends1.3. Research - Life history & ecology2.2. Conservation Planning - Area-based Management Plan3.1. Monitoring - Population trends3.4. Monitoring - Habitat trends

Justification for research needed: Further research is needed into its ecology and life history in order to find extant specimens in the Pico Alto region (including non-native habitats) and obtain information on population size, distribution and trends. It is also necessary an area-based management plan and a monitoring plan for the invertebrate community in the habitat in order to contribute to perform a species potential recovery plan. Monitoring every ten years using the BALA protocol will inform about habitat quality (see e.g. Gaspar et al. 2011).

#### Viability analysis

Justification for probability: 

### Drouetius azoricus

#### Species information

Scientific name: Drouetius azoricus

Species authority: (Drouet, 1859)

Synonyms: *Laparocerus azoricus* Drouet, 1859

Common names: True weevil, Snout beetle, Weevil (English); Gorgulho (Portuguese)

Kingdom: Animalia

Phylum: Arthropoda

Class: Insecta

Order: Coleoptera

Family: Curculionidae

Taxonomic notes: Machado 2009 describes in detail all the subspecies of this taxon originally described by Drouet in 1859. Differences between the subspecies are based on shape of rostrum, thorax and scales.

**Figure 15.**
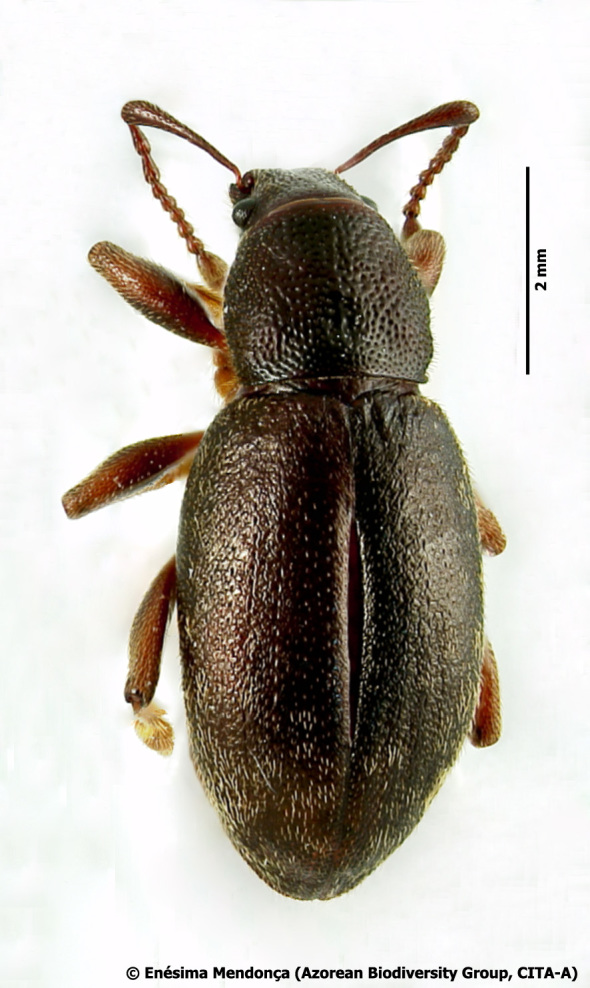
*Drouetius azoricus nitens* from Flores (Azores) (Credit: Enésima Mendonça).

Figure(s) or Photo(s): Fig. 15

Region for assessment: Global

#### Editor & Reviewers

##### Reviewers

Reviewers: Anja Danielczak

##### Editor

Editor: Axel Hochkirch

#### Reviewers

Reviewers: Anja Danielczak

#### Editor

Editor: Axel Hochkirch

#### Geographic range

Biogeographic realm: Palearctic

Countries: Portugal

Map of records (Google Earth): Suppl. material 20

Basis of EOO and AOO: Observed

Basis (narrative): The extent of occurrence (EOO) is 23,800 km^2^ and the maximum area of occupancy (AOO) is 76 km^2^.

Min Elevation/Depth (m): 0

Max Elevation/Depth (m): 500

Range description: *Drouetius azoricus* is an endemic species with four subspecies, *D. a. azoricus* occurring in Faial, S. Jorge and Graciosa islands; *D. a. nitens* occurring in Occidental group (Corvo and Flores islands); *D. a. parallelirostris* restricted to Terceira island and *D. a. separandus* restricted to S. Miguel island (Azores, Portugal) (Borges et al. 2010). *D. a. parallelirostris* is known from a small area within Terceira Natural Park.

#### New occurrences

#### Extent of occurrence

EOO (km2): 23,800

Trend: Decline (inferred)

Justification for trend: The Extent of Occurrence includes large areas of unsuitable habitats. The species continues in decline due to native forest destruction at lower altitudes and habitat continuing degradation and fragmentation.

Causes ceased?: No

Causes understood?: Yes

Causes reversible?: Unknown

Extreme fluctuations?: Unknown

#### Area of occupancy

Trend: Decline (inferred)

Justification for trend: The species continues in decline due to native forest destruction at lower altitudes and habitat continuing degradation and fragmentation.

Causes ceased?: No

Causes understood?: Yes

Causes reversible?: Unknown

Extreme fluctuations?: Unknown

AOO (km2): 76

#### Locations

Number of locations: 11

Justification for number of locations: This species occurs in eleven isolated locations at lower altitudes by the occidental group (Flores and Corvo islands), central group (Faial, S. Jorge, Graciosa and Terceira islands) and also in São Miguel. All these locations are under threat due to continuous change of the habitat as a consequence Human activities at lower elevations. The locations keeping native vegetation are also changing due to the spread of invasive plants (e.g. *Pittosporum undulatum*)

Trend: Decline (inferred)

Justification for trend: Eleven locations known but the original area was larger due to major land-use changes in low elevation habitats.

Extreme fluctuations?: Unknown

#### Population

Trend: Decline (inferred)

Justification for trend: The species is rare (very few specimens known) and known from subpopulations in low elevation areas in several islands (Corvo, Flores, Faial, S. Jorge, Graciosa, Terceira and S. Miguel islands). A continuing decline in the number of mature individuals is inferred from the ongoing habitat degradation due to human activities at lower elevations.

Basis for decline: (c) a decline in area of occupancy, extent of occurrence and/or quality of habitat

Causes ceased?: No

Causes understood?: Yes

Causes reversible?: Unknown

Extreme fluctuations?: Unknown

#### Subpopulations

Number of subpopulations: 7

Trend: Decline (inferred)

Justification for trend: The species is very rare (very few specimens known) and known from subpopulations in low elevation areas in seven islands (Corvo, Flores, Faial, S. Jorge, Graciosa, Terceira and S. Miguel islands). A continuing decline in the number of subpopulations is inferred from the ongoing habitat degradation due to human activities at lower elevations.

Extreme fluctuations?: Unknown

Severe fragmentation?: Yes

Justification for fragmentation: As a consequence of major past and ongoing land-use changes at low and middle elevations in all islands the seven subpoulations are restricted to small patches.

#### Habitat

System: Terrestrial

Habitat specialist: Yes

Habitat (narrative): This species has four subspecies (*D. a. azoricus* present in Faial, S. Jorge and Graciosa islands; *D. a. nitens* present in Occidental group (Corvo and Flores islands); *D. a. parallelirostris* restricted to Terceira island and *D. a. separandus* restricted to S. Miguel island), and occurs in modified native forests (dominated by *Erica azorica* and *Morella faya*), exotic forests and semi-natural pastures (Machado 2009). Some specimens can also be found inside caves at Terceira island, possibly falling there when migrating to the soil (adults stay underground during the day). This species has an altitudinal range between 0 and 500 m.

Trend in extent, area or quality?: Decline (observed)

Justification for trend: Due to major land-use changes at low elevations, with the destruction of many habitats for urbanization and implementation of agriculture activities.

##### Habitat

Habitat importance: Major Importance

Habitats: 1.4. Forest - Temperate4. Grassland7.1. Caves and Subterranean Habitats (non-aquatic) - Caves14.2. Artificial/Terrestrial - Pastureland

#### Habitat

Habitat importance: Major Importance

Habitats: 1.4. Forest - Temperate4. Grassland7.1. Caves and Subterranean Habitats (non-aquatic) - Caves14.2. Artificial/Terrestrial - Pastureland

#### Ecology

Size: 0.62-0.82 cm

Generation length (yr): 1

Dependency of single sp?: No

Ecology and traits (narrative): It is frequent to find specimens in caves, since the adults stay underground during the day. The fact that the species is polyphagous facilitates its survival in a highly human modified territory at lower elevations. Adults and larvae are herbivores and feed on plant tissues. This is an univoltine species.

#### Threats

Justification for threats: In the past, the species has probably strongly declined due to changes in habitat size and quality (Terzopoulou et al. 2015, Triantis et al. 2010). One of the most important ongoing threat to this species is the continuous change of habitat due to Human activities at lower elevations. The sites keeping native vegetation are also changing due to the spread of invasive plants (e.g. *Pittosporum undulatum*). Based on Ferreira et al. 2016 the habitat will further decline as a consequence of climate change (increasing number of droughts and Habitat shifting & alteration).

##### Threats

Threat type: Ongoing

Threats: 1.1. Residential & commercial development - Housing & urban areas2.2.1. Agriculture & aquaculture - Wood & pulp plantations - Small-holder plantations8.1.2. Invasive and other problematic species, genes & diseases - Invasive non-native/alien species/diseases - Named species9.1.1. Pollution - Domestic & urban waste water - Sewage

##### Threats

Threat type: Future

Threats: 11.1. Climate change & severe weather - Habitat shifting & alteration11.2. Climate change & severe weather - Droughts

#### Threats

Threat type: Ongoing

Threats: 1.1. Residential & commercial development - Housing & urban areas2.2.1. Agriculture & aquaculture - Wood & pulp plantations - Small-holder plantations8.1.2. Invasive and other problematic species, genes & diseases - Invasive non-native/alien species/diseases - Named species9.1.1. Pollution - Domestic & urban waste water - Sewage

#### Threats

Threat type: Future

Threats: 11.1. Climate change & severe weather - Habitat shifting & alteration11.2. Climate change & severe weather - Droughts

#### Conservation

Justification for conservation actions: The species is not protected by regional law. Its habitat is in a regionally protected area (Natural Park of Terceira). In the other six islands none of subpopulations are located within the range of protected areas. Further spread of invasive plants needs to be stopped in order to avoid any future declines of the species. Degraded habitats should be restored and a strategy needs to be developed to address the future threat by climate change. It is necessary a monitoring plan for the invertebrate community in the habitat in order to contribute to the conservation of this species. Formal education and awareness is needed to allow future investments in restored habitats at low elevations.

##### Conservation actions

Conservation action type: In Place

Conservation actions: 1. Land/water protection1.1. Land/water protection - Site/area protection

##### Conservation actions

Conservation action type: Needed

Conservation actions: 2.1. Land/water management - Site/area management2.2. Land/water management - Invasive/problematic species control2.3. Land/water management - Habitat & natural process restoration4. Education & awareness5.4.3. Law & policy - Compliance and enforcement - Sub-national level

#### Conservation actions

Conservation action type: In Place

Conservation actions: 1. Land/water protection1.1. Land/water protection - Site/area protection

#### Conservation actions

Conservation action type: Needed

Conservation actions: 2.1. Land/water management - Site/area management2.2. Land/water management - Invasive/problematic species control2.3. Land/water management - Habitat & natural process restoration4. Education & awareness5.4.3. Law & policy - Compliance and enforcement - Sub-national level

#### Other

##### Use and trade

Use type: International

##### Ecosystem services

Ecosystem service type: Less important

Ecosystem services: 7. Nutrient Cycling

##### Research needed

Research needed: 1.2. Research - Population size, distribution & trends1.3. Research - Life history & ecology2.2. Conservation Planning - Area-based Management Plan3.1. Monitoring - Population trends3.4. Monitoring - Habitat trends

Justification for research needed: Further research is needed into its ecology and life history in order to find extant specimens in all the historical localities and obtain information on population size, distribution and trends. It is also necessary a monitoring plan for the invertebrate community in the habitat in order to contribute to perform a species potential recovery plan in many of the historical localities. In addition, there is the need of special area-based management plans for most of the subpopulations.

#### Use and trade

Use type: International

#### Ecosystem services

Ecosystem service type: Less important

Ecosystem services: 7. Nutrient Cycling

#### Research needed

Research needed: 1.2. Research - Population size, distribution & trends1.3. Research - Life history & ecology2.2. Conservation Planning - Area-based Management Plan3.1. Monitoring - Population trends3.4. Monitoring - Habitat trends

Justification for research needed: Further research is needed into its ecology and life history in order to find extant specimens in all the historical localities and obtain information on population size, distribution and trends. It is also necessary a monitoring plan for the invertebrate community in the habitat in order to contribute to perform a species potential recovery plan in many of the historical localities. In addition, there is the need of special area-based management plans for most of the subpopulations.

#### Viability analysis

Justification for probability: 

### Drouetius borgesi

#### Species information

Scientific name: Drouetius borgesi

Species authority: Machado, 2009

Common names: True weevil, Snout beetle, Weevil (English); Gorgulho (Portuguese)

Kingdom: Animalia

Phylum: Arthropoda

Class: Insecta

Order: Coleoptera

Family: Curculionidae

Taxonomic notes: Machado 2009 describes in detail all the subspecies of this taxon. Differences between the subspecies are based on shape of rostrum, thorax and scales.

**Figure 16.**
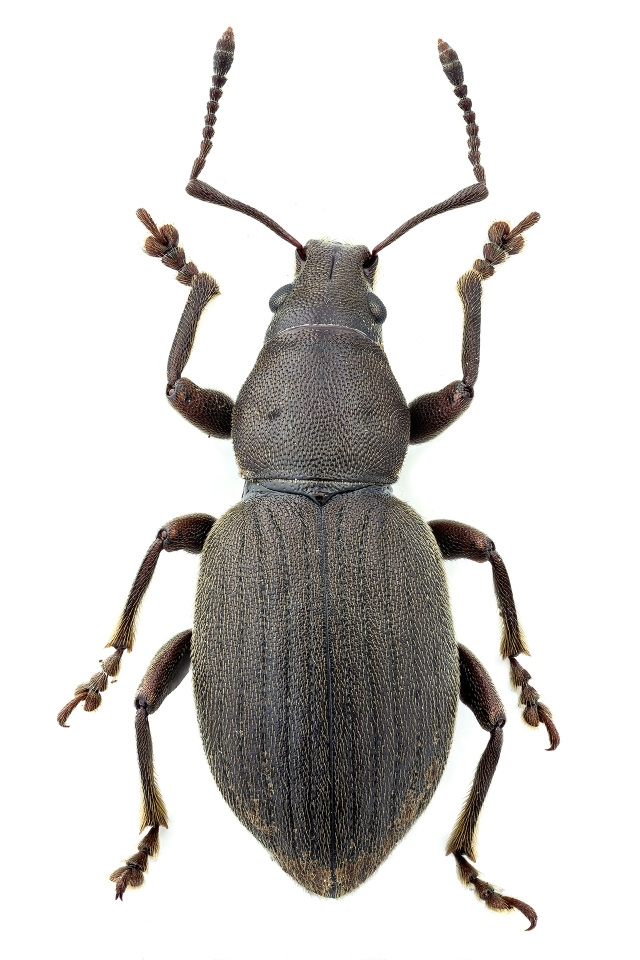
*Drouetius borgesi borgesi* from Terra Brava (Terceira, Azores) (Credit: Javier Torrent).

Figure(s) or Photo(s): Fig. 16

Region for assessment: Global

#### Editor & Reviewers

##### Reviewers

Reviewers: Anja Danielczak

##### Editor

Editor: Axel Hochkirch

#### Reviewers

Reviewers: Anja Danielczak

#### Editor

Editor: Axel Hochkirch

#### Geographic range

Biogeographic realm: Palearctic

Countries: Portugal

Map of records (Google Earth): Suppl. material 21

Basis of EOO and AOO: Observed

Basis (narrative): *Drouetius borgesi* is an endemic species with three subspecies: *D. b. borgesi* occurring in Terceira island; *D. b. centralis* occurring in Faial, Pico, S. Jorge and Graciosa islands and *D. b. sanctmichaelis* restricted to S. Miguel island (Azores, Portugal) (Borges et al. 2010). *D. borgesi* is known from the Natural Forest Reserves of Caveiro (Pico); Pico Pinheiro (S. Jorge); Pico Galhardo, Serra de Sta Bárbara and Terra Brava (Terceira); and Pico da Vara (S. Miguel).

Min Elevation/Depth (m): 300

Max Elevation/Depth (m): 1100

Range description: The extent of occurrence (EOO) is ca 11,600 km^2^ and the maximum area of occupancy (AOO) is 92 km^2^.

#### New occurrences

#### Extent of occurrence

EOO (km2): 11,600

Trend: Decline (observed)

Justification for trend: The Extent of Occurrence includes large areas of unsuitable habitats. This species continues to decline due to habitat degradation in the native forest (mostly due to invasive plants) and to habitat fragmentation.

Causes ceased?: No

Causes understood?: Yes

Causes reversible?: Unknown

Extreme fluctuations?: Unknown

#### Area of occupancy

Trend: Decline (inferred)

Justification for trend: The species occurs in the native and exotic forests of the islands of the central group (Faial, Pico, S. Jorge, Graciosa and Terceira) and S. Miguel island. Possibly the AOO value is slightly overestimated. The area of occupancy of this species continues to decline due to habitat degradation in the native forest (mostly due to invasive plants) and to habitat fragmentation.

Causes ceased?: No

Causes understood?: Yes

Causes reversible?: Unknown

Extreme fluctuations?: Unknown

AOO (km2): 92

#### Locations

Number of locations: 17

Justification for number of locations: This species occurs in 17 native and exotic forest patches distributed by the islands of the central group (Faial, Pico, S. Jorge, Graciosa and Terceira islands) and S. Miguel island.

Trend: Decline (observed)

Justification for trend: Seventeen locations known, but the original area was larger. Invasive plant species spreading (e.g. *Hedychium gardnerianum*) are changing the structure of the forest and the cover of bryophytes and ferns in the soil with impacts on the species.

Extreme fluctuations?: No

#### Population

Trend: Decline (inferred)

Justification for trend: *D. borgesi* is a widespread and still an abundant species in some pristine sites. The species currently has a decreasing population density due to the spread of the invasive plant *Hedychium gardnerianum* that is changing the structure of the forest and the cover of bryophytes and ferns in the soil with impacts on the species.

Causes ceased?: No

Causes understood?: Yes

Causes reversible?: Unknown

Extreme fluctuations?: Unknown

#### Subpopulations

Number of subpopulations: 17

Trend: Decline (inferred)

Justification for trend: *D. borgesi* is a widespread and still a highly abundant species. The species presents currently a stable population only in pristine sites. However, the spread of *Hedychium gardnerianum*) is changing the structure of the forest and the cover of bryophytes and ferns in the soil with impacts on the species and potential decline of the most disturbed subpopulations.

Extreme fluctuations?: Unknown

Severe fragmentation?: Yes

Justification for fragmentation: Major land-use changes at all elevations in all islands promoted the creation of small patches of native forest. The species occurs in 17 natural forest fragments that are isolated in a sea of pastures and *Cryptomeria japonica* plantations. In the last ten years many of those subpopulations were highly impacted by invasive plants that are changing the habitat structure, namely decreasing the cover of bryophytes and ferns in the soil and promoting the spread of other plants. The trend of invasive plant species spread will continue in future with negative impacts in most of the subpopulations.

#### Habitat

System: Terrestrial

Habitat specialist: Yes

Habitat (narrative): This species has three subspecies: *D. b. borgesi* present in Terceira island, inhabits native forests (dominated by *Juniperus brevifolia*, Ilex perado subsp. azorica, *Laurus azorica* and *Erica azorica*) and *Cryptomeria plantations*; *D. b. centralis* present in Faial, Pico, S. Jorge and Graciosa islands, inhabits in native and exotic forests, native plants on lavic formations (*Erica azorica*) and grasslands and natural pastures and *D. b. sanctmichaelis* restricted to S. Miguel island, inhabits native forests.

Trend in extent, area or quality?: Decline (inferred)

Justification for trend: The spread of *Hedychium gardnerianum* is changing the structure of the forest and the cover of bryophytes and ferns in the soil with impacts on the species.

##### Habitat

Habitat importance: Major Importance

Habitats: 1.4. Forest - Temperate14.3. Artificial/Terrestrial - Plantations16. Introduced vegetation

#### Habitat

Habitat importance: Major Importance

Habitats: 1.4. Forest - Temperate14.3. Artificial/Terrestrial - Plantations16. Introduced vegetation

#### Ecology

Size: 0.17 - 0.26 cm

Generation length (yr): 1

Dependency of single sp?: No

Ecology and traits (narrative): This species has an altitudinal range between 300 and 1100 m. Adults and larvae are herbivores and feed on plant tissues, mostly leafs and during the night. Based on seasonal data from SLAM traps obtained in several islands between 2012 and 2016 (Borges et al. 2017b), the adults are active all year, being most abundant in spring and summer.

#### Threats

Justification for threats: In the past, the species has probably strongly declined due to changes in habitat size and quality (Triantis et al. 2010; Terzopoulou et al. 2015). The most important ongoing threats to this species are *Cryptomeria japonica* wood & pulp plantations management and the spread of invasive plants (e.g. *Hedychium gardnerianum*) that are changing the habitat structure, namely decreasing the cover of bryophytes and ferns in the soil and promoting the spread of other plants. Based on Ferreira et al. (2016) the habitat will further decline as a consequence of climate change (increasing number of droughts and habitat shifting & alteration).

##### Threats

Threat type: Ongoing

Threats: 2.1.1. Agriculture & aquaculture - Annual & perennial non-timber crops - Shifting agriculture8.1.2. Invasive and other problematic species, genes & diseases - Invasive non-native/alien species/diseases - Named species

##### Threats

Threat type: Future

Threats: 11.1. Climate change & severe weather - Habitat shifting & alteration11.2. Climate change & severe weather - Droughts

#### Threats

Threat type: Ongoing

Threats: 2.1.1. Agriculture & aquaculture - Annual & perennial non-timber crops - Shifting agriculture8.1.2. Invasive and other problematic species, genes & diseases - Invasive non-native/alien species/diseases - Named species

#### Threats

Threat type: Future

Threats: 11.1. Climate change & severe weather - Habitat shifting & alteration11.2. Climate change & severe weather - Droughts

#### Conservation

**Figure 17.**
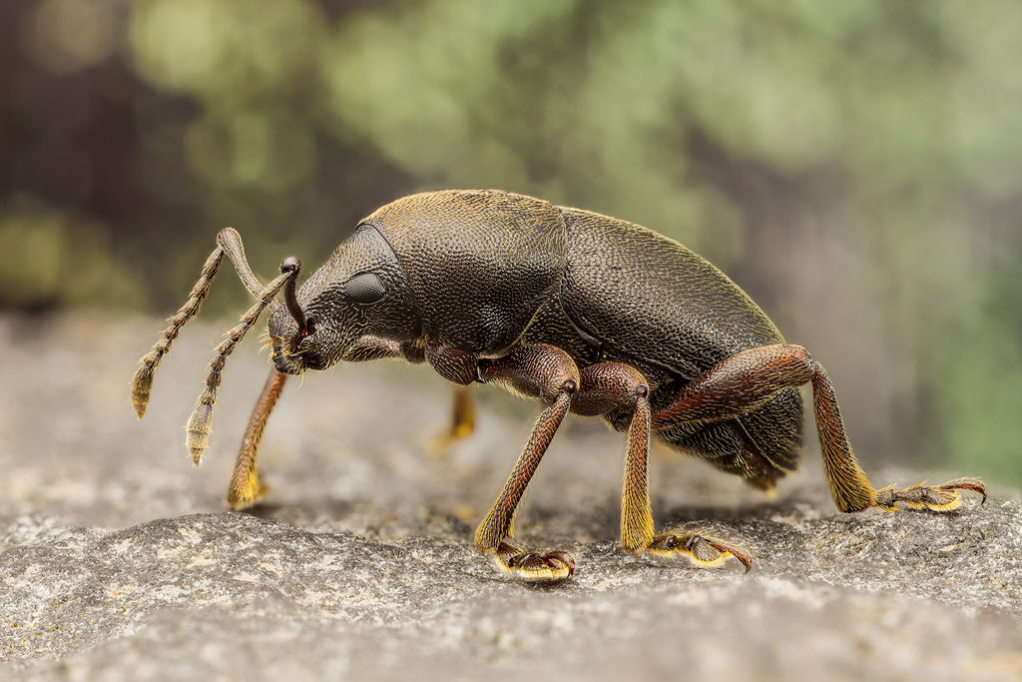
Extreme macro photo of *Drouetius borgesi borgesi* from Terra Brava (Terceira, Azores) (Credit: Javier Torrent).

Justification for conservation actions: The species is not protected by regional law. Its habitat is in regionally protected areas (Natural Parks of Pico S. Jorge, Terceira and S. Miguel). The Terceira Natural Park administration is currently starting control measures of the invasive plants. Degraded habitats should be restored and a strategy needs to be developed to address the future threat by climate change. It is necessary a monitoring plan for the invertebrate community in the habitat in order to contribute to the conservation of this species. A habitat management plan is needed and anticipated to be developed during the coming years. Since this species occurs in relict native Azorean forests, some awareness measures were put recently in practice using for instance images from extreme macro (see Fig. 17 and Arroz et al. 2016)

##### Conservation actions

Conservation action type: In Place

Conservation actions: 1. Land/water protection1.1. Land/water protection - Site/area protection2. Land/water management2.2. Land/water management - Invasive/problematic species control4. Education & awareness

##### Conservation actions

Conservation action type: Needed

Conservation actions: 2.1. Land/water management - Site/area management2.2. Land/water management - Invasive/problematic species control2.3. Land/water management - Habitat & natural process restoration4. Education & awareness5.4.3. Law & policy - Compliance and enforcement - Sub-national level

#### Conservation actions

Conservation action type: In Place

Conservation actions: 1. Land/water protection1.1. Land/water protection - Site/area protection2. Land/water management2.2. Land/water management - Invasive/problematic species control4. Education & awareness

#### Conservation actions

Conservation action type: Needed

Conservation actions: 2.1. Land/water management - Site/area management2.2. Land/water management - Invasive/problematic species control2.3. Land/water management - Habitat & natural process restoration4. Education & awareness5.4.3. Law & policy - Compliance and enforcement - Sub-national level

#### Other

Justification for use and trade: This species is not utilized.

##### Use and trade

Use type: International

##### Ecosystem services

Ecosystem service type: Important

Ecosystem services: 8. Habitat Maintenance

##### Research needed

Research needed: 1.2. Research - Population size, distribution & trends1.3. Research - Life history & ecology3.1. Monitoring - Population trends3.4. Monitoring - Habitat trends

Justification for research needed: Further research is needed into its ecology and life history in order to find extant specimens in more sites and obtain information on population size, distribution and trends. Monitoring every ten years using the BALA protocol will inform about habitat quality (see e.g. Gaspar et al. 2011).

#### Use and trade

Use type: International

#### Ecosystem services

Ecosystem service type: Important

Ecosystem services: 8. Habitat Maintenance

#### Research needed

Research needed: 1.2. Research - Population size, distribution & trends1.3. Research - Life history & ecology3.1. Monitoring - Population trends3.4. Monitoring - Habitat trends

Justification for research needed: Further research is needed into its ecology and life history in order to find extant specimens in more sites and obtain information on population size, distribution and trends. Monitoring every ten years using the BALA protocol will inform about habitat quality (see e.g. Gaspar et al. 2011).

#### Viability analysis

Justification for probability: 

### Drouetius oceanicus

#### Species information

Scientific name: Drouetius oceanicus

Species authority: Machado, 2009

Synonyms: *Laparocerus azoricus* Drouet, 1859

Common names: True weevil, Snout beetle, Weevil (English); Gorgulho (Portuguese)

Kingdom: Animalia

Phylum: Arthropoda

Class: Insecta

Order: Coleoptera

Family: Curculionidae

Taxonomic notes: Machado 2009 describes in detail all the subspecies of this new taxon. Differences between the subspecies are based on shape of rostrum, thorax and scales.

**Figure 18.**
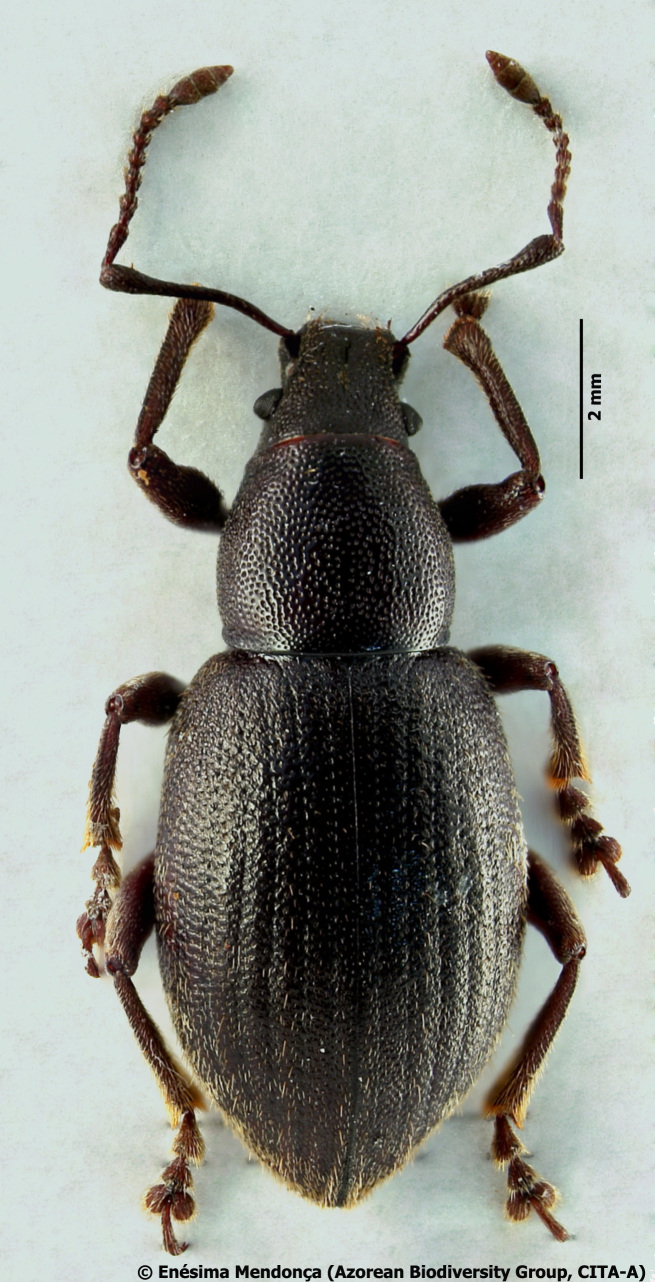
*Drouetius oceanicus tristis* from Pico Timão (Graciosa, Azores) (Credit: Enésima Mendonça).

Figure(s) or Photo(s): Fig. 18

Region for assessment: Global

#### Editor & Reviewers

##### Reviewers

Reviewers: Anja Danielczak

##### Editor

Editor: Axel Hochkirch

#### Reviewers

Reviewers: Anja Danielczak

#### Editor

Editor: Axel Hochkirch

#### Geographic range

Biogeographic realm: Palearctic

Countries: Portugal

Map of records (Google Earth): Suppl. material 22

Basis of EOO and AOO: Observed

Basis (narrative): The extent of occurrence (EOO) is ca 22,000 km^2^ and the maximum area of occupancy (AOO) is 40 km^2^.

Min Elevation/Depth (m): 0

Max Elevation/Depth (m): 200

Range description: *Drouetius oceanicus* is an endemic species with two subspecies: *D. o. oceanicus* restricted to Terceira island and *D. o. tristis* occurring in Corvo, Faial, S. Jorge, Graciosa and S. Miguel islands (Azores, Portugal) (Borges et al. 2010). The species occurs in coastal areas, but in Graciosa also at higher altitudes (Pico Timão).

#### New occurrences

#### Extent of occurrence

EOO (km2): 22,000

Trend: Decline (inferred)

Justification for trend: The Extent of Occurrence includes many unsuitable habitats not occupied by this species. The species continues in decline due to native forest destruction at lower elevations and habitat continuing destruction and fragmentation.

Causes ceased?: No

Causes understood?: Yes

Causes reversible?: Unknown

Extreme fluctuations?: Unknown

#### Area of occupancy

Trend: Decline (inferred)

Justification for trend: The species continues in decline due to native forest destruction at lower elevations and habitat continuing destruction and fragmentation.

Causes ceased?: No

Causes understood?: Yes

Causes reversible?: Unknown

Extreme fluctuations?: Unknown

AOO (km2): 40

#### Locations

Number of locations: 6

Justification for number of locations: This species occurs in six isolated locations in the islands of Corvo, Faial, S. Jorge, Graciosa, Terceira and S. Miguel islands under threat.

Trend: Decline (inferred)

Justification for trend: Six locations known but the original area was larger due to major changes in low elevation habitats. Possibly extinct in some of these six locations and in danger in the others due to continuing disturbance and land-use changes for urbanization ang agriculture activities.

Extreme fluctuations?: Unknown

#### Population

Trend: Decline (inferred)

Justification for trend: The species is extremely rare and there are six known subpopulations at low elevation areas in several islands (Corvo, Faial, S. Jorge, Graciosa, Terceira and São Miguel islands). A continuing decline in the number of mature individuals is inferred from the ongoing habitat degradation due to human activities. Recently in 2016 (Borges et al. 2017a) found two specimens in the historical site from Terceira in a highly degraded marsh associated with native and exotic plants (Paul do Belo Jardim).

Basis for decline: (c) a decline in area of occupancy, extent of occurrence and/or quality of habitat

Causes ceased?: No

Causes understood?: Yes

Causes reversible?: Unknown

Extreme fluctuations?: Unknown

#### Subpopulations

Number of subpopulations: 6

Trend: Decline (inferred)

Justification for trend: The species is extremely rare and there are six known subpopulations at low elevations in several islands (Corvo, Faial, S. Jorge, Graciosa, Terceira and São Miguel islands). A continuing decline in the number of subpopulations is inferred from the ongoing habitat degradation due to human activities. Recently in 2016 few specimens were found in the historical site from Terceira in a highly degraded marsh associated with native and exotic plants (Paul do Belo Jardim) (Borges et al. 2017a).

Extreme fluctuations?: Unknown

Severe fragmentation?: Yes

Justification for fragmentation: Major land-use changes at low and middle elevations in all islands promoted the creation of small patches of native and exotic forest. The species occurs in six islands in small patches of exotic habitats fragmented due to urban and agriculture development, that will keep occurring in the next ten years in all the subpopulations.

#### Habitat

System: Terrestrial

Habitat specialist: Yes

Habitat (narrative): This species has two subspecies (*D. o. oceanicus* restricted to Terceira island and *D. o. tristis* present in Corvo, Faial, S. Jorge, Graciosa and S. Miguel islands), and occurs in modified native forests (dominated by *Erica azorica* and *Morella faya*), exotic forests and marsh areas (in Terceira) (Machado 2009; Borges et al. 2017a). Some of the historical llocalities are completely destroyed and urbanized or with intensive pastures, which may imply local extinction. The subpopulation of Terceira is located in a small water course near a march area surrounded by an industrial area. This species has an altitudinal range between 0 and 200 m.

Trend in extent, area or quality?: Decline (observed)

Justification for trend: The habitat is being highly modified and is currently under extreme disturbance regimes due to urbanization and agriculture activities at low elevations.

##### Habitat

Habitat importance: Major Importance

Habitats: 1.4. Forest - Temperate3.4. Shrubland - Temperate5.16. Wetlands (inland) - Permanent Saline, Brackish or Alkaline Marshes/Pools14.2. Artificial/Terrestrial - Pastureland16. Introduced vegetation

#### Habitat

Habitat importance: Major Importance

Habitats: 1.4. Forest - Temperate3.4. Shrubland - Temperate5.16. Wetlands (inland) - Permanent Saline, Brackish or Alkaline Marshes/Pools14.2. Artificial/Terrestrial - Pastureland16. Introduced vegetation

#### Ecology

Size: 0.70-0.82 cm

Generation length (yr): 1

Dependency of single sp?: No

Ecology and traits (narrative): Adults and larvae are herbivores and feed on plant tissues. The fact that the species is polyphagous facilitates its survival in a highly human modified territory at lower elevations. This is an univoltine species.

#### Threats

Justification for threats: In the past, the species has probably strongly declined due to changes in habitat size and quality (Terzopoulou et al. 2015, Triantis et al. 2010). One of the most important ongoing threat to this species is the continuous change of habitat due to Human activities at lower elevations, mostly urbanization and pollution, but also agriculture activities. The sites with still native vegetation are also changing due to the spread of invasive plants (e.g. *Pittosporum undulatum*) and managment of pulp plantations. Based on Ferreira et al. 2016 the habitat will further decline as a consequence of climate change (increasing number of droughts and habitat shifting & alteration due to severe weather).

##### Threats

Threat type: Ongoing

Threats: 1.1. Residential & commercial development - Housing & urban areas2.2.1. Agriculture & aquaculture - Wood & pulp plantations - Small-holder plantations8.1.2. Invasive and other problematic species, genes & diseases - Invasive non-native/alien species/diseases - Named species

##### Threats

Threat type: Future

Threats: 11.1. Climate change & severe weather - Habitat shifting & alteration11.2. Climate change & severe weather - Droughts

#### Threats

Threat type: Ongoing

Threats: 1.1. Residential & commercial development - Housing & urban areas2.2.1. Agriculture & aquaculture - Wood & pulp plantations - Small-holder plantations8.1.2. Invasive and other problematic species, genes & diseases - Invasive non-native/alien species/diseases - Named species

#### Threats

Threat type: Future

Threats: 11.1. Climate change & severe weather - Habitat shifting & alteration11.2. Climate change & severe weather - Droughts

#### Conservation

Justification for conservation actions: The species is not protected by regional law. Further spread of invasive plants needs to be stopped in order to avoid any future declines of the species. Degraded habitats should be restored and a strategy needs to be developed to address the future threat by climate change. It is necessary a monitoring plan for the invertebrate community in the habitat in order to contribute to the conservation of this species. Formal education and awareness is needed to allow future investments in restored habitats at low elevations.

##### Conservation actions

Conservation action type: Needed

Conservation actions: 1.1. Land/water protection - Site/area protection2.1. Land/water management - Site/area management2.2. Land/water management - Invasive/problematic species control2.3. Land/water management - Habitat & natural process restoration4. Education & awareness5.4.3. Law & policy - Compliance and enforcement - Sub-national level

#### Conservation actions

Conservation action type: Needed

Conservation actions: 1.1. Land/water protection - Site/area protection2.1. Land/water management - Site/area management2.2. Land/water management - Invasive/problematic species control2.3. Land/water management - Habitat & natural process restoration4. Education & awareness5.4.3. Law & policy - Compliance and enforcement - Sub-national level

#### Other

##### Use and trade

Use type: International

##### Ecosystem services

Ecosystem service type: Less important

Ecosystem services: 7. Nutrient Cycling

##### Research needed

Research needed: 1.2. Research - Population size, distribution & trends1.3. Research - Life history & ecology2.2. Conservation Planning - Area-based Management Plan3.1. Monitoring - Population trends3.4. Monitoring - Habitat trends

Justification for research needed: Further research is needed into its ecology and life history in order to find extant specimens at lower elevations and obtain information on population size, distribution and trends. It is also necessary a monitoring plan for the invertebrate community in the habitat in order to contribute to perform a species potential recovery plan. In addition, there is the need of special area-based management plans for most of the subpopulations. Monitoring every ten years using the BALA protocol will inform about habitat quality (see e.g. Gaspar et al. 2011).

#### Use and trade

Use type: International

#### Ecosystem services

Ecosystem service type: Less important

Ecosystem services: 7. Nutrient Cycling

#### Research needed

Research needed: 1.2. Research - Population size, distribution & trends1.3. Research - Life history & ecology2.2. Conservation Planning - Area-based Management Plan3.1. Monitoring - Population trends3.4. Monitoring - Habitat trends

Justification for research needed: Further research is needed into its ecology and life history in order to find extant specimens at lower elevations and obtain information on population size, distribution and trends. It is also necessary a monitoring plan for the invertebrate community in the habitat in order to contribute to perform a species potential recovery plan. In addition, there is the need of special area-based management plans for most of the subpopulations. Monitoring every ten years using the BALA protocol will inform about habitat quality (see e.g. Gaspar et al. 2011).

#### Viability analysis

Justification for probability: 

### Neocnemis occidentalis

#### Species information

Scientific name: Neocnemis occidentalis

Species authority: Crotch, 1867

Common names: True weevil, Snout beetle, Weevil (English); Gorgulho (Portuguese)

Kingdom: Animalia

Phylum: Arthropoda

Class: Insecta

Order: Coleoptera

Family: Curculionidae

Region for assessment: Global

#### Editor & Reviewers

##### Reviewers

Reviewers: Anja Danielczak

##### Editor

Editor: Axel Hochkirch

#### Reviewers

Reviewers: Anja Danielczak

#### Editor

Editor: Axel Hochkirch

#### Geographic range

Biogeographic realm: Palearctic

Countries: Portugal

Map of records (Google Earth): Suppl. material 23

Basis of EOO and AOO: Observed

Basis (narrative): This species is considered extinct in Azores (Terzopoulou et al. 2015).

Min Elevation/Depth (m): 450

Max Elevation/Depth (m): 550

Range description: *Neocnemis occidentalis* is a single-island endemic species from Santa Maria (Azores, Portugal) (Borges et al. 2010).

#### New occurrences

#### Extent of occurrence

EOO (km2): 0-4

Trend: Stable

Justification for trend: Based on the area of a unique cell of the historical locality. The species is considered extinct in the historical locality possibly due to habitat destruction. Not sampled recently despite some intensive field work (Borges et al. 2005, Borges et al. 2016).

Causes ceased?: No

Causes understood?: Yes

Causes reversible?: Unknown

Extreme fluctuations?: Unknown

#### Area of occupancy

Trend: Stable

Justification for trend: Based on the area of a unique cell of the historical locality. The species is considered extinct in the historical locality possibly due to habitat destruction. Not sampled recently despite some intensive field work (Borges et al. 2005, Borges et al. 2016).

Causes ceased?: No

Causes understood?: Yes

Causes reversible?: Unknown

Extreme fluctuations?: Unknown

AOO (km2): 0-4

#### Locations

Number of locations: 0-1

Justification for number of locations: The species is potentially extinct due to destruction of the habitat in all its range (Terzopoulou et al. 2015).

Trend: Unknown

Justification for trend: Possibly went extinct more than 10 years ago.

Extreme fluctuations?: Unknown

#### Population

Trend: Unknown

Justification for trend: The species is only known from a single subpopulation. A continuing decline in the number of mature individuals is inferred from historical records. According to Terzopoulou et al. 2015 this species was extinct more than ten years ago.

Causes ceased?: No

Causes understood?: Yes

Causes reversible?: Unknown

Extreme fluctuations?: Unknown

#### Subpopulations

Number of subpopulations: 0-1

Trend: Unknown

Justification for trend: The species is only known from a single subpopulation. According to Terzopoulou et al. 2015 this species was extinct more than ten years ago.

Extreme fluctuations?: Unknown

Severe fragmentation?: No

#### Habitat

System: Terrestrial

Habitat specialist: Yes

Habitat (narrative): The species occured in the native forest of Santa Maria Island (Azores), with an altitudinal range between 450 and 550 m. It is considered extinct.

Trend in extent, area or quality?: Decline (observed)

Justification for trend: Since the historical record, the native habitat in the island of Santa Maria was greatly reduced to accomodate *Cryptomeria japonica* plantations (Triantis et al. 2010). In the last ten years invasive plant species are spreading (e.g. *Hedychium gardnerianum*; *Pittosporum undulatum*) changing the structure of the forest and the cover of bryophytes and ferns in the soil which will impact the species habitat quality.

##### Habitat

Habitat importance: Major Importance

Habitats: 1.4. Forest - Temperate

#### Habitat

Habitat importance: Major Importance

Habitats: 1.4. Forest - Temperate

#### Ecology

Generation length (yr): 1

Dependency of single sp?: No

Ecology and traits (narrative): It is a phytophagous species. This is an univoltine species.

#### Threats

Justification for threats: In the past, the species has probably strongly declined due to changes in habitat size and quality (Terzopoulou et al. 2015). The most important ongoing threat to this species is the spread of invasive plants (*Hedychium gardnerianum* and *Pittosporum undulatum*) that are changing the habitat structure in the main native forest, namely decreasing the cover of bryophytes and ferns in the soil and promoting the spread of other plants. Based on Ferreira et al. 2016 the habitat will further decline as a consequence of climate change (increasing number of droughts and habitat shifting & alteration).

##### Threats

Threat type: Ongoing

Threats: 2.2.1. Agriculture & aquaculture - Wood & pulp plantations - Small-holder plantations8.1.2. Invasive and other problematic species, genes & diseases - Invasive non-native/alien species/diseases - Named species

##### Threats

Threat type: Future

Threats: 11.1. Climate change & severe weather - Habitat shifting & alteration11.2. Climate change & severe weather - Droughts

#### Threats

Threat type: Ongoing

Threats: 2.2.1. Agriculture & aquaculture - Wood & pulp plantations - Small-holder plantations8.1.2. Invasive and other problematic species, genes & diseases - Invasive non-native/alien species/diseases - Named species

#### Threats

Threat type: Future

Threats: 11.1. Climate change & severe weather - Habitat shifting & alteration11.2. Climate change & severe weather - Droughts

#### Conservation

Justification for conservation actions: The species is not protected by regional law. Its habitat is in a regionally protected area (Natural Park of Santa Maria). Degraded habitats should be restored and of critical importance is the removal of invasive non-native species where this is possible. A strategy needs to be developed to address the future threat by climate change. It is necessary a monitoring plan for the invertebrate community in the habitat in order to contribute to the conservation of this species. A habitat management plan is needed and anticipated to be developed during the coming years. Formal education and awareness is needed to allow future investments in restored habitats invaded by invasive plants.

##### Conservation actions

Conservation action type: In Place

Conservation actions: 1. Land/water protection1.1. Land/water protection - Site/area protection2.2. Land/water management - Invasive/problematic species control

##### Conservation actions

Conservation action type: Needed

Conservation actions: 2.1. Land/water management - Site/area management2.2. Land/water management - Invasive/problematic species control2.3. Land/water management - Habitat & natural process restoration4. Education & awareness5.4.3. Law & policy - Compliance and enforcement - Sub-national level

#### Conservation actions

Conservation action type: In Place

Conservation actions: 1. Land/water protection1.1. Land/water protection - Site/area protection2.2. Land/water management - Invasive/problematic species control

#### Conservation actions

Conservation action type: Needed

Conservation actions: 2.1. Land/water management - Site/area management2.2. Land/water management - Invasive/problematic species control2.3. Land/water management - Habitat & natural process restoration4. Education & awareness5.4.3. Law & policy - Compliance and enforcement - Sub-national level

#### Other

##### Use and trade

Use type: International

##### Ecosystem services

Ecosystem service type: Less important

Ecosystem services: 8. Habitat Maintenance

##### Research needed

Research needed: 1.2. Research - Population size, distribution & trends1.3. Research - Life history & ecology2.2. Conservation Planning - Area-based Management Plan3.1. Monitoring - Population trends3.4. Monitoring - Habitat trends

Justification for research needed: Further research is needed into its ecology and life history in order to find extant specimens at Pico Alto (Santa Maria) and obtain information on population size, distribution and trends. It is also necessary an area-based management plan and a monitoring plan for the invertebrate community in the habitat in order to contribute to perform a species potential recovery plan. Monitoring every ten years using the BALA protocol will inform about habitat quality (see e.g. Gaspar et al. 2011).

#### Use and trade

Use type: International

#### Ecosystem services

Ecosystem service type: Less important

Ecosystem services: 8. Habitat Maintenance

#### Research needed

Research needed: 1.2. Research - Population size, distribution & trends1.3. Research - Life history & ecology2.2. Conservation Planning - Area-based Management Plan3.1. Monitoring - Population trends3.4. Monitoring - Habitat trends

Justification for research needed: Further research is needed into its ecology and life history in order to find extant specimens at Pico Alto (Santa Maria) and obtain information on population size, distribution and trends. It is also necessary an area-based management plan and a monitoring plan for the invertebrate community in the habitat in order to contribute to perform a species potential recovery plan. Monitoring every ten years using the BALA protocol will inform about habitat quality (see e.g. Gaspar et al. 2011).

#### Viability analysis

Justification for probability: 

### Phloeosinus gillerforsi

#### Species information

Scientific name: Phloeosinus gillerforsi

Species authority: Bright, 1987

Common names: Bark beetle (English); Caruncho-do-cedro-do-mato (Portuguese)

Kingdom: Animalia

Phylum: Arthropoda

Class: Insecta

Order: Coleoptera

Family: Curculionidae

Taxonomic notes: This species was described from individuals collected in Pico island, between 29.VII.1983 and 25.6.1985. These are deposited in G. Israelson collection and in the Canadian National Collection of Insects. Adults are easily recognized by the convex elytral declivity on which the first and third interstriae are, at most, very weakly elevated and all interstriae bear a row of minute to small granules and distinct punctures and by the distinct, median carina on the frons of both sexes (Bright 1987).

Region for assessment: Global

#### Editor & Reviewers

##### Reviewers

Reviewers: Anja Danielczak

##### Editor

Editor: Axel Hochkirch

#### Reviewers

Reviewers: Anja Danielczak

#### Editor

Editor: Axel Hochkirch

#### Geographic range

Biogeographic realm: Palearctic

Countries: Portugal

Map of records (Google Earth): Suppl. material 24

Basis of EOO and AOO: Observed

Basis (narrative): The extent of occurrence (EOO) is ca 10,100 km^2^ and the maximum area of occupancy (AOO) is 40 km^2^.

Min Elevation/Depth (m): 0

Max Elevation/Depth (m): 1200

Range description: *Phloeosinus gillerforsi* is an endemic species present in Pico, S. Jorge, Terceira and S. Miguel islands (Azores, Portugal) (Borges et al. 2010), known from Natural Forest Reserves of Mistério da Prainha (Pico); Pico Pinheiro (S. Jorge); Biscoito da Ferraria and Serra de Sta. Bárbara (Terceira); Atalhada and Pico da Vara (S. Miguel).

#### New occurrences

#### Extent of occurrence

EOO (km2): 10,100

Trend: Decline (inferred)

Justification for trend: The species is well adapted to *Juniperus brevifolia*, species that is protected, but is reducing its area due to invasive plants and forest fragmentation.

Causes ceased?: No

Causes understood?: Yes

Causes reversible?: Unknown

Extreme fluctuations?: Unknown

#### Area of occupancy

Trend: Decline (inferred)

Justification for trend: The species occurs in the native forests of the Pico, S. Jorge, Terceira and S. Miguel islands. The AOO value including native forest is around 30 km^2^. The species may decline due to invasive plants that are promoting host tree *Juniperus brevifolia* habitat fragmentation.

Causes ceased?: No

Causes understood?: Yes

Causes reversible?: Unknown

Extreme fluctuations?: Unknown

AOO (km2): 40

#### Locations

Number of locations: 8

Justification for number of locations: This species occurs in eigth isolated native forest patches in the Pico, S. Jorge, Terceira and S. Miguel islands.

Trend: Decline (inferred)

Justification for trend: Eight locations known but the original area was larger. In the last ten years invasive plant species are spreading (e.g. *Hedychium gardnerianum*; *Hydrangea macrophylla, Pittosporum undulatum, Clethra arborea*), changing the structure of the forest and the cover of bryophytes and ferns in the soil which will impact the species habitat quality.

Extreme fluctuations?: Unknown

#### Population

Trend: Decline (inferred)

Justification for trend: *P. gillerforsi* is a widespread and particularly abundant species in native forests (very common in the host tree *Juniperus brevifolia*). The species is currently abundant but a declining in the abundance of some subpopulation is inferred from the fragmentation and declining of the host species *Juniperus brevifolia*.

Causes ceased?: No

Causes understood?: Yes

Causes reversible?: Unknown

Extreme fluctuations?: Unknown

#### Subpopulations

Number of subpopulations: 8

Trend: Decline (inferred)

Justification for trend: *P. gillerforsi* is a widespread and particularly abundant species in native forests (very common in the host tree *Juniperus brevifolia*). A declining in the number of subpopulations is inferred from the fragmentation and declining in the abundance of the host species *Juniperus brevifolia*.

Extreme fluctuations?: Unknown

Severe fragmentation?: No

#### Habitat

System: Terrestrial

Habitat specialist: Yes

Habitat (narrative): The species occurs in native forests dominated by the main host Azorean endemic tree *Juniperus brevifolia* in Pico, S. Jorge, Terceira and S. Miguel islands (Azores), with an altitudinal range between 0 and 1200 m.

Trend in extent, area or quality?: Stable

Justification for trend: The habitat is more or less stable since the host plant is protected.

##### Habitat

Habitat importance: Major Importance

Habitats: 1.4. Forest - Temperate

#### Habitat

Habitat importance: Major Importance

Habitats: 1.4. Forest - Temperate

#### Ecology

Size: 0.32 cm

Generation length (yr): 0

Dependency of single sp?: Yes

Dependent on species: *Juniperus brevifolia*

Dependent on IUCN Status: Vulnerable (VU)

Ecology and traits (narrative): Adults and larvae are herbivores and feed on plant tissues of the host tree, the Azorean Cedar *Juniperus brevifolia*. Based on seasonal data from SLAM traps obtained in several islands between 2012 and 2016 (Borges et al. 2017b), the adults are active all year, being most abundant in spring and autumn.

#### Threats

Justification for threats: In the past, the species has probably strongly declined due to changes in habitat size and quality (Terzopoulou et al. 2015, Triantis et al. 2010). One of the most important ongoing threat to this species is the fragmentation of the host plant habitat due to invasive plants (e.g. *Hedychium gardnerianum*; *Hydrangea macrophylla, Pittosporum undulatum, Clethra arborea*) and *Cryptomeria japonica* pulp plantation management. Based on Ferreira et al. 2016 the habitat will further decline as a consequence of climate change (increasing number of droughts).

##### Threats

Threat type: Ongoing

Threats: 2.2.1. Agriculture & aquaculture - Wood & pulp plantations - Small-holder plantations8.1.2. Invasive and other problematic species, genes & diseases - Invasive non-native/alien species/diseases - Named species

##### Threats

Threat type: Future

Threats: 11.1. Climate change & severe weather - Habitat shifting & alteration11.2. Climate change & severe weather - Droughts

#### Threats

Threat type: Ongoing

Threats: 2.2.1. Agriculture & aquaculture - Wood & pulp plantations - Small-holder plantations8.1.2. Invasive and other problematic species, genes & diseases - Invasive non-native/alien species/diseases - Named species

#### Threats

Threat type: Future

Threats: 11.1. Climate change & severe weather - Habitat shifting & alteration11.2. Climate change & severe weather - Droughts

#### Conservation

Justification for conservation actions: The species is not protected by regional law. Its habitat is in regionally protected areas (Natural Parks of Pico, S. Jorge, Terceira, Pico and S. Miguel). Further spread of invasive plants needs to be stopped in order to avoid any future declines of the host plant species. Degraded habitats should be restored and a strategy needs to be developed to address the future threat by climate change. It is necessary a monitoring plan for the invertebrate community in the habitat in order to contribute to the conservation of this species. A habitat management plan is needed and anticipated to be developed during the coming years.

##### Conservation actions

Conservation action type: In Place

Conservation actions: 1. Land/water protection1.1. Land/water protection - Site/area protection2.2. Land/water management - Invasive/problematic species control

##### Conservation actions

Conservation action type: Needed

Conservation actions: 2.1. Land/water management - Site/area management2.2. Land/water management - Invasive/problematic species control2.3. Land/water management - Habitat & natural process restoration4. Education & awareness5.4.3. Law & policy - Compliance and enforcement - Sub-national level

#### Conservation actions

Conservation action type: In Place

Conservation actions: 1. Land/water protection1.1. Land/water protection - Site/area protection2.2. Land/water management - Invasive/problematic species control

#### Conservation actions

Conservation action type: Needed

Conservation actions: 2.1. Land/water management - Site/area management2.2. Land/water management - Invasive/problematic species control2.3. Land/water management - Habitat & natural process restoration4. Education & awareness5.4.3. Law & policy - Compliance and enforcement - Sub-national level

#### Other

##### Use and trade

Use type: International

##### Ecosystem services

Ecosystem service type: Less important

Ecosystem services: 7. Nutrient Cycling

##### Research needed

Research needed: 1.2. Research - Population size, distribution & trends1.3. Research - Life history & ecology3.1. Monitoring - Population trends3.4. Monitoring - Habitat trends

Justification for research needed: Further research is needed into its ecology and life history in order to find extant specimens in small fragments with the host plant *Juniperus brevifolia*. and obtain information on population size, distribution and trends. It is also necessary a monitoring plan for the invertebrate community in the habitat in order to contribute to perform a species potential recovery plan in some areas. Monitoring every ten years using the BALA protocol will inform about habitat quality (see e.g. Gaspar et al. 2011).

#### Use and trade

Use type: International

#### Ecosystem services

Ecosystem service type: Less important

Ecosystem services: 7. Nutrient Cycling

#### Research needed

Research needed: 1.2. Research - Population size, distribution & trends1.3. Research - Life history & ecology3.1. Monitoring - Population trends3.4. Monitoring - Habitat trends

Justification for research needed: Further research is needed into its ecology and life history in order to find extant specimens in small fragments with the host plant *Juniperus brevifolia*. and obtain information on population size, distribution and trends. It is also necessary a monitoring plan for the invertebrate community in the habitat in order to contribute to perform a species potential recovery plan in some areas. Monitoring every ten years using the BALA protocol will inform about habitat quality (see e.g. Gaspar et al. 2011).

#### Viability analysis

Justification for probability: 

### Pseudechinosoma nodosum

#### Species information

Scientific name: Pseudechinosoma nodosum

Species authority: Hustache, 1936

Common names: True weevil, Snout beetle, Weevil (English); Gorgulho-casca-de-noz (Portuguese).

Kingdom: Animalia

Phylum: Arthropoda

Class: Insecta

Order: Coleoptera

Family: Curculionidae

**Figure 19.**
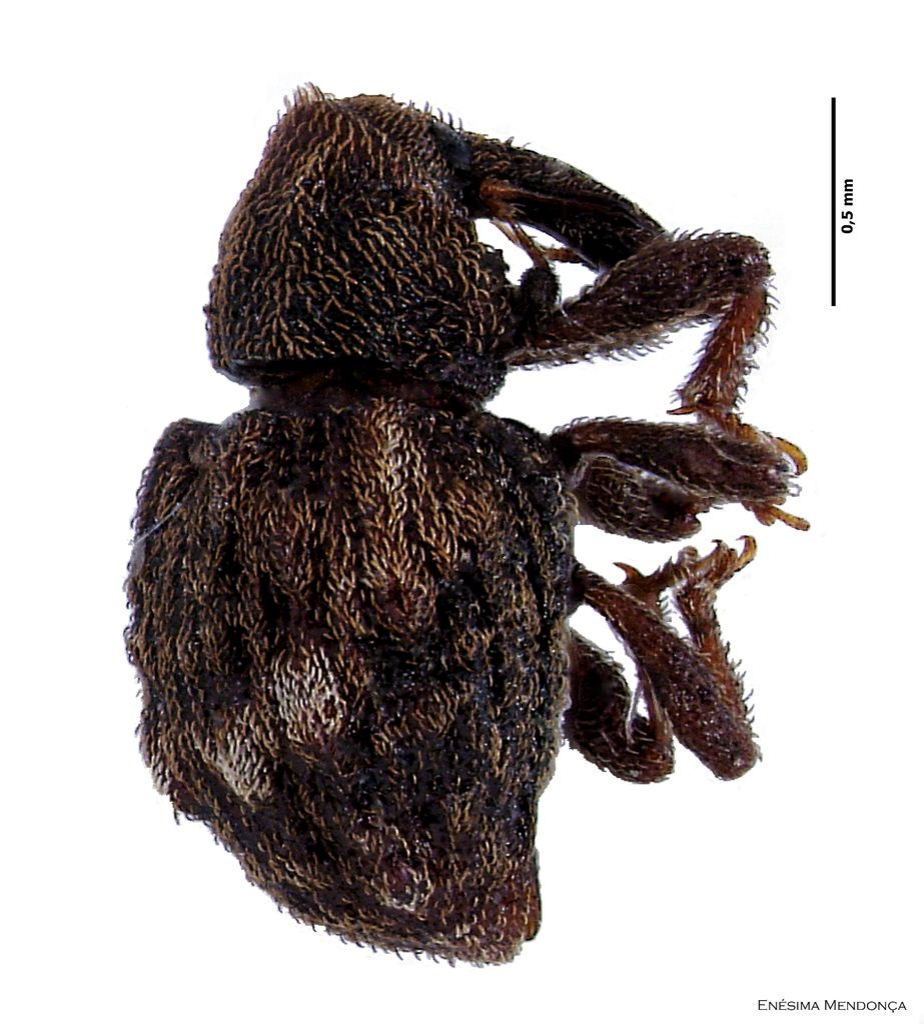
*Pseudechinosoma nodosum* from Pico Alto (Santa Maria, Azores) (Credit: Enésima Mendonça).

Figure(s) or Photo(s): Fig. 19

Region for assessment: Global

#### Editor & Reviewers

##### Reviewers

Reviewers: Anja Danielczak

##### Editor

Editor: Axel Hochkirch

#### Reviewers

Reviewers: Anja Danielczak

#### Editor

Editor: Axel Hochkirch

#### Geographic range

Biogeographic realm: Palearctic

Countries: Portugal

Map of records (Google Earth): Suppl. material 25

Basis of EOO and AOO: Observed

Basis (narrative): *Pseudechinosoma nodosum* is an endemic species present in Flores, Faial, Pico, S. Jorge, Terceira, S. Miguel and Santa Maria islands (Azores, Portugal) (Borges et al. 2010), known from Natural Forest Reserves of Caveiro and Mistério da Prainha (Pico); Pico Pinheiro (S. Jorge); Pico Galhardo and Terra Brava (Terceira); Graminhais and Pico da Vara (S. Miguel) and Pico Alto (Santa Maria).

Min Elevation/Depth (m): 400

Max Elevation/Depth (m): 1200

Range description: The extent of occurrence (EOO) is ca 33,700 km^2^ and the maximum area of occupancy (AOO) is 100 km^2^.

#### New occurrences

#### Extent of occurrence

EOO (km2): 33,700

Trend: Decline (observed)

Justification for trend: The EOO includes large areas of unsuitable habitats. The EOO of this species continues to decline due to habitat degradation in the native forest (mostly due to invasive plants) and to habitat fragmentation.

Causes ceased?: No

Causes understood?: Yes

Causes reversible?: Unknown

Extreme fluctuations?: Unknown

#### Area of occupancy

Trend: Decline (observed)

Justification for trend: The species occurs in the native and exotic forests of the Flores, Faial, Pico, S. Jorge, Terceira, S. Miguel and Santa Maria islands. Possibly the AOO value is slightly overestimated. The species continues in decline due to native forest destruction and habitat fragmentation.

Causes ceased?: No

Causes understood?: Yes

Causes reversible?: Unknown

Extreme fluctuations?: Unknown

AOO (km2): 100

#### Locations

Number of locations: 13

Justification for number of locations: This species occurs in 13 native and exotic forest fragmented patches in the Flores, Faial, Pico, S. Jorge,Terceira, S. Miguel and Santa Maria islands.

Trend: Decline (inferred)

Justification for trend: Thirteen locations known but the original area was larger. The natural forest reserves of Pico Pinheiro (S. Jorge); Pico Galhardo (Terceira) and Pico Alto (St. Maria) have a very low Index of Biotic Integrity (Gaspar et al. 2011) and in the last ten years invasive plant species spreading (e.g. *Hedychium gardnerianum*; *Pittosporum undulatum*) are changing the structure of the forest and the cover of bryophytes and ferns in the soil decreasing the quality of the habitat with impacts on the species.

Extreme fluctuations?: No

#### Population

Trend: Decline (inferred)

Justification for trend: *P. nodosum* is a widespread and particularly abundant species in native and few patches of exotic forests. The species currently has a decreasing population density due to the spread of the invasive plant *Hedychium gardnerianum* that is changing the structure of the forest and the cover of bryophytes and ferns in the soil with impacts on the species

Causes ceased?: No

Causes understood?: Yes

Causes reversible?: Unknown

Extreme fluctuations?: Unknown

#### Subpopulations

Number of subpopulations: 13

Trend: Decline (inferred)

Justification for trend: *P. nodosum* is a widespread and particularly abundant species in native and few patches of exotic forests. A decreasing in the number of subpopulations is inferred as a consequence of the spread of the invasive plant *Hedychium gardnerianum* that is changing the structure of the forest and the cover of bryophytes and ferns in the soil with impacts on the species.

Extreme fluctuations?: Unknown

Severe fragmentation?: No

#### Habitat

System: Terrestrial

Habitat specialist: Yes

Habitat (narrative): The species occurs in native forests (mainly dominated by *Juniperus brevifolia*, *Laurus azorica* and *Ilex perado spp. azorica*) and *Cryptomeria japonica* plantations in Flores, Faial, Pico, S. Jorge, Terceira, S. Miguel and Santa Maria islands (Azores), with an altitudinal range between 400 and 1200 m.

Trend in extent, area or quality?: Decline (inferred)

Justification for trend: In the past, the species has probably strongly declined due to changes in habitat size and quality (Triantis et al. 2010). Currently the quality of the habitat in part of its range is decreasing due to the invasions of alien plants (namely *Hedychium gardnerianum*) and *Cryptomeria japonica* plantations managemen.

##### Habitat

Habitat importance: Major Importance

Habitats: 1.4. Forest - Temperate14.3. Artificial/Terrestrial - Plantations16. Introduced vegetation

#### Habitat

Habitat importance: Major Importance

Habitats: 1.4. Forest - Temperate14.3. Artificial/Terrestrial - Plantations16. Introduced vegetation

#### Ecology

Size: 0.18 - 0.25 cm

Generation length (yr): 1

Dependency of single sp?: No

Ecology and traits (narrative): This is an univoltine species. Adults and larvae are herbivores and feed on plant tissues. This is a dead wood specialist (i.e. saprophagous).

#### Threats

Justification for threats: In the past, the species has probably strongly declined due to changes in habitat size and quality (Triantis et al. 2010, Terzopoulou et al. 2015). The most important ongoing threats to this species are *Cryptomeria japonica* wood & pulp plantations management an the spread of invasive plants changing the structure of the forest. Based on Ferreira et al. (2016) the habitat will further decline as a consequence of climate change (increasing number of droughts and habitat shifting & alteration).

##### Threats

Threat type: Ongoing

Threats: 2.2.1. Agriculture & aquaculture - Wood & pulp plantations - Small-holder plantations8.1.2. Invasive and other problematic species, genes & diseases - Invasive non-native/alien species/diseases - Named species

##### Threats

Threat type: Future

Threats: 11.1. Climate change & severe weather - Habitat shifting & alteration11.2. Climate change & severe weather - Droughts

#### Threats

Threat type: Ongoing

Threats: 2.2.1. Agriculture & aquaculture - Wood & pulp plantations - Small-holder plantations8.1.2. Invasive and other problematic species, genes & diseases - Invasive non-native/alien species/diseases - Named species

#### Threats

Threat type: Future

Threats: 11.1. Climate change & severe weather - Habitat shifting & alteration11.2. Climate change & severe weather - Droughts

#### Conservation

Justification for conservation actions: The species is not protected by regional law. Its habitat is in regionally protected areas (Natural Parks of Pico, S. Jorge, Terceira, Pico, S. Miguel and Santa Maria). The Terceira Natural Park administration is currently starting control measures of the invasive plants. Further spread of invasive plants needs to be stopped in most islands order to avoid any future declines of the species. Degraded habitats should be restored and a strategy needs to be developed to address the future threat by climate change. It is necessary a monitoring plan for the invertebrate community in the habitat in order to contribute to the conservation of this species. A habitat management plan is needed and anticipated to be developed during the coming years.

##### Conservation actions

Conservation action type: In Place

Conservation actions: 1. Land/water protection1.1. Land/water protection - Site/area protection2. Land/water management2.2. Land/water management - Invasive/problematic species control4. Education & awareness

##### Conservation actions

Conservation action type: Needed

Conservation actions: 2.1. Land/water management - Site/area management2.2. Land/water management - Invasive/problematic species control2.3. Land/water management - Habitat & natural process restoration4. Education & awareness5.4.3. Law & policy - Compliance and enforcement - Sub-national level

#### Conservation actions

Conservation action type: In Place

Conservation actions: 1. Land/water protection1.1. Land/water protection - Site/area protection2. Land/water management2.2. Land/water management - Invasive/problematic species control4. Education & awareness

#### Conservation actions

Conservation action type: Needed

Conservation actions: 2.1. Land/water management - Site/area management2.2. Land/water management - Invasive/problematic species control2.3. Land/water management - Habitat & natural process restoration4. Education & awareness5.4.3. Law & policy - Compliance and enforcement - Sub-national level

#### Other

Justification for use and trade: The species is not utilized.

##### Use and trade

Use type: International

##### Ecosystem services

Ecosystem service type: Important

Ecosystem services: 7. Nutrient Cycling8. Habitat Maintenance

##### Research needed

Research needed: 1.2. Research - Population size, distribution & trends1.3. Research - Life history & ecology3.1. Monitoring - Population trends3.4. Monitoring - Habitat trends

Justification for research needed: Further research is needed into its ecology and life history in order to find extant specimens in more native forest sites and obtain information on population size, distribution and trends. It is also necessary a monitoring plan for the invertebrate community in the habitat in order to contribute to perform a species potential recovery plan in some of the islands. An Area-based Management Plan is needed for some of the subpopulations, namely in Santa Maria and S. Miguel. Monitoring every ten years using the BALA protocol will inform about habitat quality (see e.g. Gaspar et al. 2011).

#### Use and trade

Use type: International

#### Ecosystem services

Ecosystem service type: Important

Ecosystem services: 7. Nutrient Cycling8. Habitat Maintenance

#### Research needed

Research needed: 1.2. Research - Population size, distribution & trends1.3. Research - Life history & ecology3.1. Monitoring - Population trends3.4. Monitoring - Habitat trends

Justification for research needed: Further research is needed into its ecology and life history in order to find extant specimens in more native forest sites and obtain information on population size, distribution and trends. It is also necessary a monitoring plan for the invertebrate community in the habitat in order to contribute to perform a species potential recovery plan in some of the islands. An Area-based Management Plan is needed for some of the subpopulations, namely in Santa Maria and S. Miguel. Monitoring every ten years using the BALA protocol will inform about habitat quality (see e.g. Gaspar et al. 2011).

#### Viability analysis

Justification for probability: 

### Sphaericus velhocabrali

#### Species information

Scientific name: Sphaericus velhocabrali

Species authority: Israelson, 1984

Common names: Cigarette beetle, Death wacth beetle, Drugstore beetle, Furniture beetle, Powerpost beetle (English)

Kingdom: Animalia

Phylum: Arthropoda

Class: Insecta

Order: Coleoptera

Family: Anobiidae

Taxonomic notes: *Sphaericus velhocabrali* was described from a individuals collected in Santa. Maria island, on the 9.VII.1982 (Israelson 1984a).

Region for assessment: Global

#### Editor & Reviewers

##### Reviewers

Reviewers: Anja Danielczak

##### Editor

Editor: Axel Hochkirch

#### Reviewers

Reviewers: Anja Danielczak

#### Editor

Editor: Axel Hochkirch

#### Geographic range

Biogeographic realm: Palearctic

Countries: Portugal

Map of records (Google Earth): Suppl. material 26

Basis of EOO and AOO: Observed

Basis (narrative): *Sphaericus velhocabrali* is a single-island endemic species from Santa Maria (Azores, Portugal) (Borges et al. 2010).

Min Elevation/Depth (m): 0

Max Elevation/Depth (m): 150

Range description: The extent of occurrence (EOO) is 8 km^2^ and the maximum area of occupancy (AOO) is 8 km^2^. The species cccurs only in a small coastal area at Ponta de São Lourenço.

#### New occurrences

#### Extent of occurrence

EOO (km2): 8

Trend: Decline (observed)

Justification for trend: This species occurs in habitats associated with agricultural activities of Santa Maria island. Possibly the EOO value is slightly overestimated since the species has a very small range. The species continues in decline due to human land-use change and agricultural activities at low elevations.

Causes ceased?: No

Causes understood?: Yes

Causes reversible?: Unknown

Extreme fluctuations?: Unknown

#### Area of occupancy

Trend: Decline (observed)

Justification for trend: This species occurs in habitats associated with agricultural activities of Santa Maria island. Possibly the AOO value is slightly overestimated. The species continues in decline due to human disturbance.

Causes ceased?: No

Causes understood?: Yes

Causes reversible?: Unknown

Extreme fluctuations?: Unknown

AOO (km2): 8

#### Locations

Number of locations: 1

Justification for number of locations: This species occurs in habitats associated with agricultural activities (vineyards) of Santa Maria island.

Trend: Unknown

Extreme fluctuations?: No

#### Population

Trend: Decline (inferred)

Justification for trend: The species is only known from a single subpopulation on Santa Maria island. A continuing decline in the number of mature individuals is inferred due to the ongoing threats, mainly due to human disturbance associated to agricultural activities.

Basis for decline: (c) a decline in area of occupancy, extent of occurrence and/or quality of habitat

Causes ceased?: No

Causes understood?: Yes

Causes reversible?: Unknown

Extreme fluctuations?: Unknown

#### Subpopulations

Number of subpopulations: 1

Trend: Unknown

Justification for trend: The species is only known from a single subpopulation on Santa Maria island.

Extreme fluctuations?: Unknown

Severe fragmentation?: No

#### Habitat

System: Terrestrial

Habitat specialist: Yes

Habitat (narrative): This species occurs in habitats associated to agricultural areas (vineyards) in Sant Maria island (Azores), with an altitudinal range between 0 and 150 m (Israelson 1984a).

Trend in extent, area or quality?: Decline (inferred)

Justification for trend: Due to human disturbance associated to agricultural activities.

##### Habitat

Habitat importance: Major Importance

Habitats: 14.3. Artificial/Terrestrial - Plantations16. Introduced vegetation

#### Habitat

Habitat importance: Major Importance

Habitats: 14.3. Artificial/Terrestrial - Plantations16. Introduced vegetation

#### Ecology

Size: 0.14 - 0.20 cm

Generation length (yr): 1

Dependency of single sp?: No

Ecology and traits (narrative): This is an univoltine species. *S. velhocabrali* is an herbivorous xylophagous species (i.e. whose diet consists primarily of wood).

#### Threats

Justification for threats: In the past, the species has probably strongly declined due to changes in habitat size and quality (Triantis et al. 2010). Based on Ferreira et al. (2016) the habitat will further decline as a consequence of climate change (increasing number of droughts and habitat shifting & alteration). In addition, environmental degradation is also occurring due to agriculture activities an invasive species, such as *Hedychium gardnerianum*, since this species is changing the habitat structure, namely decreasing the cover of bryophytes and ferns in the soil and promoting the spread of other plants. Main additional threat will be the change of vineyards to other land-use, or urban development due to tourism.

##### Threats

Threat type: Ongoing

Threats: 2.1. Agriculture & aquaculture - Annual & perennial non-timber crops8.1.2. Invasive and other problematic species, genes & diseases - Invasive non-native/alien species/diseases - Named species

##### Threats

Threat type: Future

Threats: 1.1. Residential & commercial development - Housing & urban areas11.1. Climate change & severe weather - Habitat shifting & alteration11.2. Climate change & severe weather - Droughts

#### Threats

Threat type: Ongoing

Threats: 2.1. Agriculture & aquaculture - Annual & perennial non-timber crops8.1.2. Invasive and other problematic species, genes & diseases - Invasive non-native/alien species/diseases - Named species

#### Threats

Threat type: Future

Threats: 1.1. Residential & commercial development - Housing & urban areas11.1. Climate change & severe weather - Habitat shifting & alteration11.2. Climate change & severe weather - Droughts

#### Conservation

Justification for conservation actions: The species is not protected by regional law, but some enforcement should be put in place in this direction. Thus, it is necessary a monitoring plan for the invertebrate community in the habitat in order to contribute to the conservation of this species. Since this species is one of the few endemic species of insects in the Azores that lives associated with vineyards, it is suggested that some awareness measures should be put in practice, due to its unique natural value of the species and cultural value of vineyards for the Azorean region. Therefore, current habitat should be maintained and a strategy needs to be developed to address the future threat by climate change.

##### Conservation actions

Conservation action type: Needed

Conservation actions: 1.1. Land/water protection - Site/area protection2.1. Land/water management - Site/area management2.2. Land/water management - Invasive/problematic species control2.3. Land/water management - Habitat & natural process restoration4.1. Education & awareness - Formal education4.3. Education & awareness - Awareness & communications5.4.3. Law & policy - Compliance and enforcement - Sub-national level

#### Conservation actions

Conservation action type: Needed

Conservation actions: 1.1. Land/water protection - Site/area protection2.1. Land/water management - Site/area management2.2. Land/water management - Invasive/problematic species control2.3. Land/water management - Habitat & natural process restoration4.1. Education & awareness - Formal education4.3. Education & awareness - Awareness & communications5.4.3. Law & policy - Compliance and enforcement - Sub-national level

#### Other

Justification for use and trade: The species is not utilized.

##### Use and trade

Use type: International

##### Ecosystem services

Ecosystem service type: Important

Ecosystem services: 8. Habitat Maintenance

##### Research needed

Research needed: 1.2. Research - Population size, distribution & trends1.3. Research - Life history & ecology2.2. Conservation Planning - Area-based Management Plan3.1. Monitoring - Population trends3.4. Monitoring - Habitat trends

Justification for research needed: Further research is needed into its ecology and life history in order to find extant specimens in additional coastal areas in Santa Maria and obtain information on population size, distribution and trends. It is also necessary a monitoring plan for the invertebrate community in the habitat in order to contribute to perform a species potential recovery plan.

#### Use and trade

Use type: International

#### Ecosystem services

Ecosystem service type: Important

Ecosystem services: 8. Habitat Maintenance

#### Research needed

Research needed: 1.2. Research - Population size, distribution & trends1.3. Research - Life history & ecology2.2. Conservation Planning - Area-based Management Plan3.1. Monitoring - Population trends3.4. Monitoring - Habitat trends

Justification for research needed: Further research is needed into its ecology and life history in order to find extant specimens in additional coastal areas in Santa Maria and obtain information on population size, distribution and trends. It is also necessary a monitoring plan for the invertebrate community in the habitat in order to contribute to perform a species potential recovery plan.

#### Viability analysis

Justification for probability: 

### Athous azoricus

#### Species information

Scientific name: Athous azoricus

Species authority: Platia & Gudenzi, 2002

Common names: Click beetle, Wireworm (English); Escaravelho-mola-dos-Açores (Portuguese)

Kingdom: Animalia

Phylum: Arthropoda

Class: Insecta

Order: Coleoptera

Family: Elateridae

Taxonomic notes: *Athous azoricus* was described from a single male collected in S. Miguel island. Female has the same coloration of the male; it differs latter by the more convex and quadrangular pronotum and shorter antennae not reaching for two articles the apices of the posterior angles of pronotum (Platia and Borges 2002).

**Figure 20.**
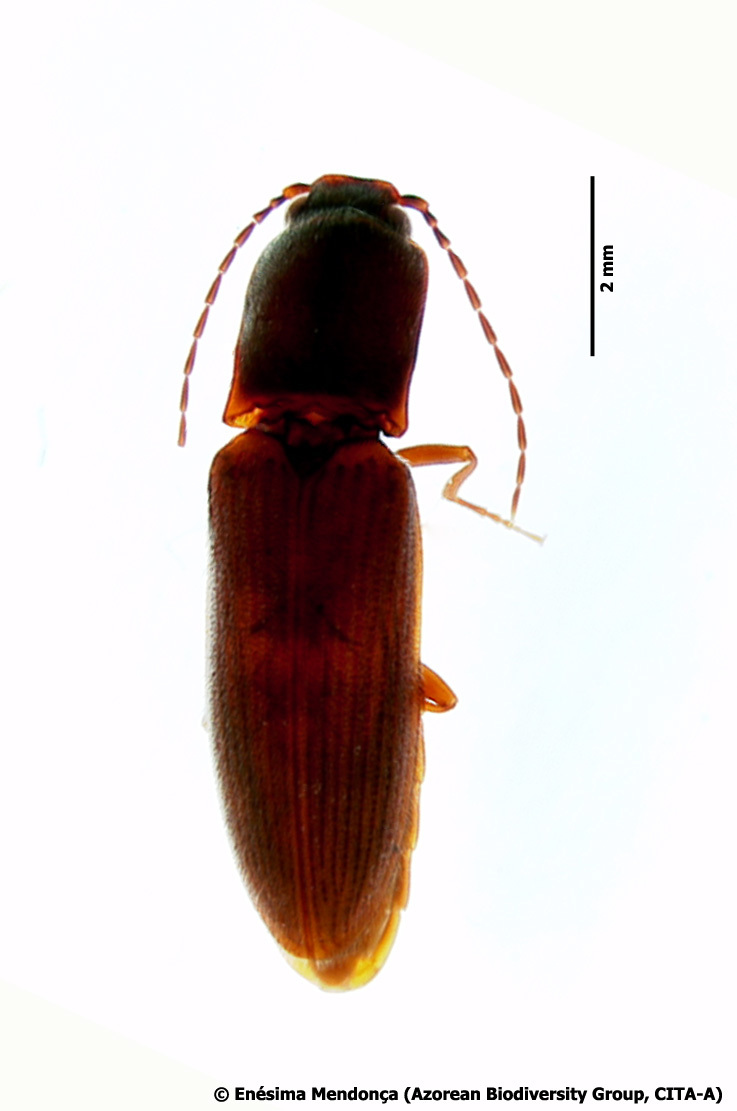
*Athous azoricus* from Terceira (Azores) (Credit: Enésima Mendonça).

Figure(s) or Photo(s): Fig. 20

Region for assessment: Global

#### Editor & Reviewers

##### Reviewers

Reviewers: Anja Danielczak

##### Editor

Editor: Axel Hochkirch

#### Reviewers

Reviewers: Anja Danielczak

#### Editor

Editor: Axel Hochkirch

#### Geographic range

Biogeographic realm: Palearctic

Countries: Portugal

Map of records (Google Earth): Suppl. material 27

Basis of EOO and AOO: Observed

Basis (narrative): The extent of occurrence (EOO) is ca 14,000 km^2^ and the maximum area of occupancy (AOO) is 44 km^2^.

Min Elevation/Depth (m): 50

Max Elevation/Depth (m): 300

Range description: *Athous azoricus* is an endemic species present in Flores, Graciosa, Terceira and S. Miguel islands (Azores, Portugal) (Borges et al. 2010).

#### New occurrences

#### Extent of occurrence

EOO (km2): 14,000

Trend: Decline (inferred)

Justification for trend: The species keeps a decline trend due to native forest destruction, landscape transformation associated to agricultural activities, introduced species and habitat fragmentation.

Causes ceased?: No

Causes understood?: Yes

Causes reversible?: Unknown

Extreme fluctuations?: Unknown

#### Area of occupancy

Trend: Decline (inferred)

Justification for trend: The species occurs in native forests patches of the Flores, Graciosa, Terceira, and S. Miguel islands. The species keeps a decline trend due to native forest destruction, landscape transformation associated to agricultural activities, introduced species and habitat fragmentation.

Causes ceased?: Yes

Causes understood?: Yes

Causes reversible?: Unknown

Extreme fluctuations?: Unknown

AOO (km2): 44

#### Locations

Number of locations: 5

Justification for number of locations: This species occurs in five native and exotic forest patches in the Flores, Graciosa, Terceira and S. Miguel islands.

Trend: Decline (inferred)

Justification for trend: Five locations known that were highly impacted by land use changes and invasive plants in the last ten years.

Extreme fluctuations?: Unknown

#### Population

Trend: Decline (inferred)

Justification for trend: This species is rare. There is an inferred continuing decline in the number of mature individuals since the five known subpopulations are under threat due to major land-use changes at lower elevations.

Basis for decline: (c) a decline in area of occupancy, extent of occurrence and/or quality of habitat

Causes ceased?: No

Causes understood?: Yes

Causes reversible?: Unknown

Extreme fluctuations?: Unknown

#### Subpopulations

Number of subpopulations: 5

Trend: Decline (inferred)

Justification for trend: This species is rare. There is an inferred continuing decline in the number of subpopulations thatare under threat due to major land-use changes at lower elevations.

Extreme fluctuations?: Unknown

Severe fragmentation?: Yes

Justification for fragmentation: Major land-use changes at low and middle elevations promoted the creation of small patches of native and exotic forest in all islands. The species occurs in five natural and exotic forest fragments that are isolated in a sea of pastures and *Cryptomeria japonica* plantations. In the last ten years a spread of invasive plants is changing the habitat structure, namely decreasing the cover of bryophytes and ferns in the soil and promoting the spread of other plants.

#### Habitat

System: Terrestrial

Habitat specialist: Yes

Habitat (narrative): The species occurs in native and exotic forests of the Flores, Graciosa, Terceira, and S. Miguel islands (Azores), with an altitudinal range between 50 and 300 m.

Trend in extent, area or quality?: Decline (observed)

Justification for trend: Impact of introduced invasive plant speciesm namely *Hedychium gardnerianum* and *Pittosporum undulatum* and human activities decreases the quality of habitat at lower and middle elevations.

##### Habitat

Habitat importance: Major Importance

Habitats: 1.4. Forest - Temperate14.3. Artificial/Terrestrial - Plantations

#### Habitat

Habitat importance: Major Importance

Habitats: 1.4. Forest - Temperate14.3. Artificial/Terrestrial - Plantations

#### Ecology

Size: 0.95 cm

Generation length (yr): 1

Dependency of single sp?: No

Ecology and traits (narrative): Adults and larvae are herbivores and feed of plant tissues. This is an univoltine species.

#### Threats

Justification for threats: In the past, the species has probably strongly declined due to changes in habitat size and quality (Terzopoulou et al. 2015, Triantis et al. 2010). Currently the main threat to this species is the impact of introduced species and the agricultural activities, including the impact of *Cryptomeria japonica* wood & pulp plantations management. Based on Ferreira et al. 2016 the habitat will further decline as a consequence of climate change (increasing number of droughts).

##### Threats

Threat type: Ongoing

Threats: 2.1. Agriculture & aquaculture - Annual & perennial non-timber crops2.2.1. Agriculture & aquaculture - Wood & pulp plantations - Small-holder plantations8.1.2. Invasive and other problematic species, genes & diseases - Invasive non-native/alien species/diseases - Named species

##### Threats

Threat type: Future

Threats: 11.1. Climate change & severe weather - Habitat shifting & alteration11.2. Climate change & severe weather - Droughts

#### Threats

Threat type: Ongoing

Threats: 2.1. Agriculture & aquaculture - Annual & perennial non-timber crops2.2.1. Agriculture & aquaculture - Wood & pulp plantations - Small-holder plantations8.1.2. Invasive and other problematic species, genes & diseases - Invasive non-native/alien species/diseases - Named species

#### Threats

Threat type: Future

Threats: 11.1. Climate change & severe weather - Habitat shifting & alteration11.2. Climate change & severe weather - Droughts

#### Conservation

Justification for conservation actions: The species is not protected by regional law. Degraded habitats should be restored and a strategy needs to be developed to address the future threat by climate change. It is necessary a monitoring plan for the invertebrate community in the habitat in order to contribute to the conservation of this species. A habitat management plan is needed and anticipated to be developed during the coming years. Formal education and awareness is needed to allow future investments in restored habitats invaded by invasive plants.

##### Conservation actions

Conservation action type: Needed

Conservation actions: 1.1. Land/water protection - Site/area protection2.1. Land/water management - Site/area management2.2. Land/water management - Invasive/problematic species control2.3. Land/water management - Habitat & natural process restoration4. Education & awareness5.4.3. Law & policy - Compliance and enforcement - Sub-national level

#### Conservation actions

Conservation action type: Needed

Conservation actions: 1.1. Land/water protection - Site/area protection2.1. Land/water management - Site/area management2.2. Land/water management - Invasive/problematic species control2.3. Land/water management - Habitat & natural process restoration4. Education & awareness5.4.3. Law & policy - Compliance and enforcement - Sub-national level

#### Other

##### Use and trade

Use type: International

##### Ecosystem services

Ecosystem service type: Less important

Ecosystem services: 7. Nutrient Cycling

##### Research needed

Research needed: 1.2. Research - Population size, distribution & trends1.3. Research - Life history & ecology2.2. Conservation Planning - Area-based Management Plan3.1. Monitoring - Population trends3.4. Monitoring - Habitat trends

Justification for research needed: Further research is needed into its ecology and life history in order to find extant specimens in additional native forest fragments in several islands and obtain information on population size, distribution and trends. It is also necessary a area-based management plan and a monitoring plan for the invertebrate community in the habitat in order to contribute to perform a species potential recovery plan. Monitoring every ten years using the BALA protocol will inform about habitat quality (see e.g. Gaspar et al. 2011).

#### Use and trade

Use type: International

#### Ecosystem services

Ecosystem service type: Less important

Ecosystem services: 7. Nutrient Cycling

#### Research needed

Research needed: 1.2. Research - Population size, distribution & trends1.3. Research - Life history & ecology2.2. Conservation Planning - Area-based Management Plan3.1. Monitoring - Population trends3.4. Monitoring - Habitat trends

Justification for research needed: Further research is needed into its ecology and life history in order to find extant specimens in additional native forest fragments in several islands and obtain information on population size, distribution and trends. It is also necessary a area-based management plan and a monitoring plan for the invertebrate community in the habitat in order to contribute to perform a species potential recovery plan. Monitoring every ten years using the BALA protocol will inform about habitat quality (see e.g. Gaspar et al. 2011).

#### Viability analysis

Justification for probability: 

### Athous pomboi

#### Species information

Scientific name: Athous pomboi

Species authority: Platia & Borges, 2002

Common names: Click beetle, Wireworm (English); Escaravelho-mola-de-Santa-Maria (Portuguese)

Kingdom: Animalia

Phylum: Arthropoda

Class: Insecta

Order: Coleoptera

Family: Elateridae

Taxonomic notes: *Athous pomboi* was described from two individuals collected in Santa Maria island, between 20.VIII.1997 and 27.VIII.1997. Both deposited in the University of Azores in Terceira at the "Dalberto Teixeira Pombo collection". This new species is distinguished from *A. azoricus* essentially by the darker colour, in the male by the greater convexity of pronotum, the shape of anterior frontal margin, and shorter antennae (Platia and Borges 2002).

**Figure 21.**
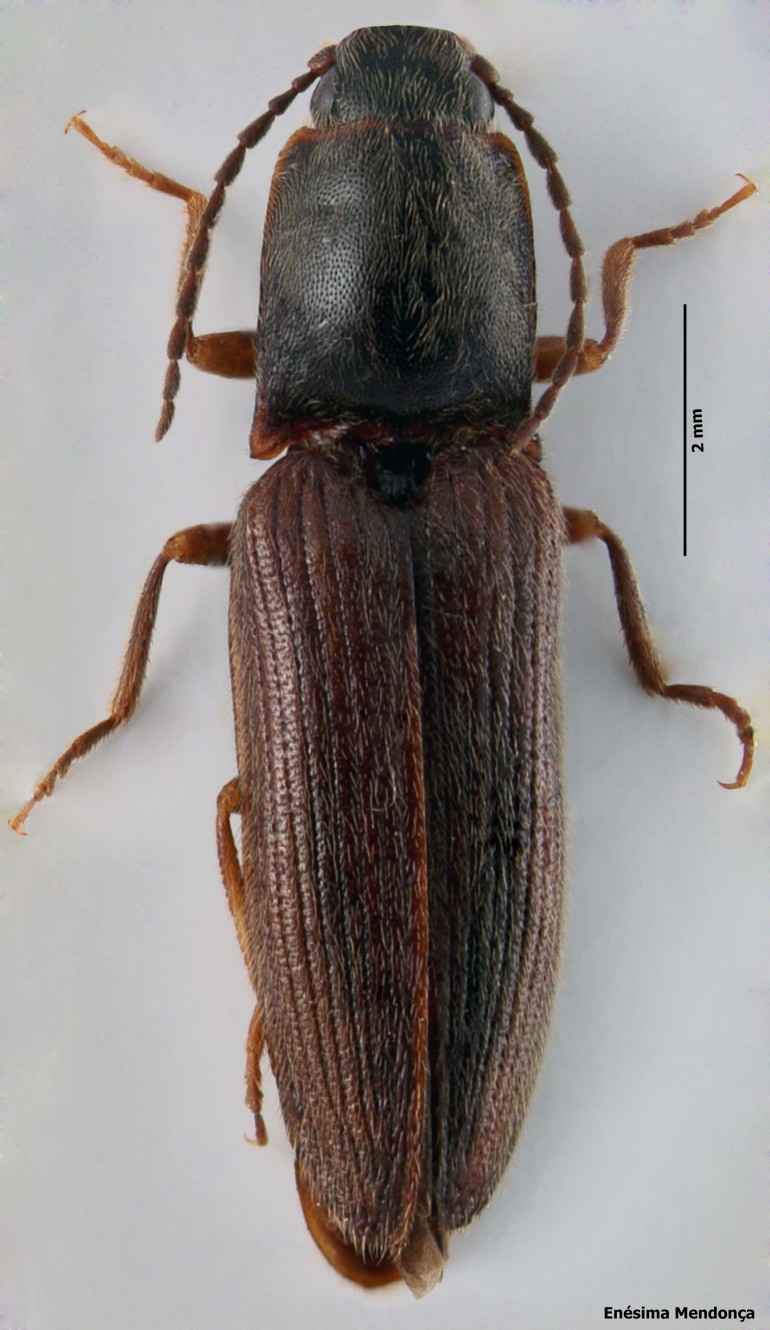
*Athous pomboi* from Pico Alto (Santa Maria, Azores) (Credit: Enésima Mendonça).

Figure(s) or Photo(s): Fig. 21

Region for assessment: Global

#### Editor & Reviewers

##### Reviewers

Reviewers: Anja Danielczak

##### Editor

Editor: Axel Hochkirch

#### Reviewers

Reviewers: Anja Danielczak

#### Editor

Editor: Axel Hochkirch

#### Geographic range

Biogeographic realm: Palearctic

Countries: Portugal

Map of records (Google Earth): Suppl. material 28

Basis of EOO and AOO: Observed

Basis (narrative): The extent of occurrence (EOO) is 40 km^2^ and the maximum area of occupancy (AOO) is 40 km^2^.

Min Elevation/Depth (m): 100

Max Elevation/Depth (m): 550

Range description: *Athous pomboi* is a single-island endemic species restricted to Santa Maria (Azores, Portugal) (Borges et al. 2010), known from Natural Forest Reserve of Pico Alto.

#### New occurrences

#### Extent of occurrence

EOO (km2): 40

Trend: Decline (inferred)

Justification for trend: This species occurs in several native and exotic forest patches of Santa Maria island. The area of its remaining native habitat is now only around 0.09 km². The extent of occurrence of this species continues in decline due to habitat degradation in the native forest (mostly due to invasive plants) and to habitat fragmentation.

Causes ceased?: No

Causes understood?: Yes

Causes reversible?: Unknown

Extreme fluctuations?: Unknown

#### Area of occupancy

Trend: Decline (inferred)

Justification for trend: The species occurs in native and exotic forests of the Sta. Maria island. The size of its remaining native habitat is now only 0.09 km². The area of occupancy of this species continues in decline due to habitat degradation in the native forest (mostly due to invasive plants) and to habitat fragmentation.

Causes ceased?: No

Causes understood?: Yes

Causes reversible?: Unknown

Extreme fluctuations?: Unknown

AOO (km2): 40

#### Locations

Number of locations: 4

Justification for number of locations: This species occurs in one native and three exotic forest patches in Sta. Maria island.

Trend: Decline (inferred)

Justification for trend: Four locations that were highly impacted by invasive plants in the last ten years. The native forest location has a very low Index of Biotic Integrity (Gaspar et al. 2011) with a area of 0.09 km² and invasive plants can drive this species to extinction very fast.

Extreme fluctuations?: Unknown

#### Population

Trend: Decline (inferred)

Justification for trend: The species is very rare in all known locations in Sta. Maria island. A continuing decline in the number of mature individuals is inferred due to small patches and the expansion of alien plants.

Basis for decline: (c) a decline in area of occupancy, extent of occurrence and/or quality of habitat

Causes ceased?: No

Causes understood?: Yes

Causes reversible?: Unknown

Extreme fluctuations?: Unknown

#### Subpopulations

Number of subpopulations: 2

Trend: Decline (inferred)

Justification for trend: The species is very rare in all known locations in Sta. Maria island. A continuing decline in the number of subpopulations is inferred due to small patches and the expansion of alien plants.

Extreme fluctuations?: Unknown

Severe fragmentation?: Yes

Justification for fragmentation: Major land-use changes at all elevations in S. Maria island promoted the creation of small patches of native and exotic forest. The species occurs in one natural forest fragment and small not sustainable small patches of exotic forest that are isolated in a sea of pastures and *Cryptomeria japonica* plantations. With exception of the native forest all the other sites are under severe threat.

#### Habitat

System: Terrestrial

Habitat specialist: Yes

Habitat (narrative): The species occurs in native forests, *Cryptomeria japonica* plantations and *Acacia* spp. exotic forests in S. Maria (Azores), with an altitudinal range between 100 and 550 m.

Trend in extent, area or quality?: Decline (observed)

Justification for trend: In the last ten years invasive plant species are spreading (e.g. *Hedychium gardnerianum*; *Pittosporum undulatum*) changing the structure of the forest and the cover of bryophytes and ferns in the soil which will impact the species habitat quality.

##### Habitat

Habitat importance: Major Importance

Habitats: 1.4. Forest - Temperate3.4. Shrubland - Temperate14.3. Artificial/Terrestrial - Plantations16. Introduced vegetation

#### Habitat

Habitat importance: Major Importance

Habitats: 1.4. Forest - Temperate3.4. Shrubland - Temperate14.3. Artificial/Terrestrial - Plantations16. Introduced vegetation

#### Ecology

Size: 0.94 cm

Generation length (yr): 1

Dependency of single sp?: No

Ecology and traits (narrative): Adults and larvae are herbivores and feed on plant tissues, being active during the night. Based on seasonal data from SLAM traps obtained in several islands between 2012 and 2016 (Borges et al. 2017b), the adults are active all year, being most abundant in summer. This is an univoltine species.

#### Threats

Justification for threats: In the past, the species has probably strongly declined due to changes in habitat size and quality (Triantis et al. 2010, Terzopoulou et al. 2015). One of the most important ongoing threats to this species is the spread of invasive plants (*Hedychium gardnerianum* and *Pittosporum undulatum*), agriculture activities, *Cryptomeria japonica* pulp plantations management and habitat degradation in the unique site of native forest. Based on Ferreira et al. 2016 the habitat will further decline as a consequence of climate change (increasing number of droughts and habitat shifting & alteration).

##### Threats

Threat type: Ongoing

Threats: 2.2.1. Agriculture & aquaculture - Wood & pulp plantations - Small-holder plantations2.3. Agriculture & aquaculture - Livestock farming & ranching8.2.2. Invasive and other problematic species, genes & diseases - Problematic native species/diseases - Named species

##### Threats

Threat type: Future

Threats: 11.1. Climate change & severe weather - Habitat shifting & alteration11.2. Climate change & severe weather - Droughts

#### Threats

Threat type: Ongoing

Threats: 2.2.1. Agriculture & aquaculture - Wood & pulp plantations - Small-holder plantations2.3. Agriculture & aquaculture - Livestock farming & ranching8.2.2. Invasive and other problematic species, genes & diseases - Problematic native species/diseases - Named species

#### Threats

Threat type: Future

Threats: 11.1. Climate change & severe weather - Habitat shifting & alteration11.2. Climate change & severe weather - Droughts

#### Conservation

Justification for conservation actions: The species is not protected by regional law. Its main native habitat is in a regionally protected area (Natural Park of Santa Maria). Further spread of invasive plants needs to be stopped in order to avoid any future declines of the species. Degraded habitats should be restored and a strategy needs to be developed to address the future threat by climate change. It is necessary a monitoring plan for the invertebrate community in the habitat in order to contribute to the conservation of this species. A habitat management plan is needed and anticipated to be developed during the coming years.

##### Conservation actions

Conservation action type: In Place

Conservation actions: 1. Land/water protection1.1. Land/water protection - Site/area protection2.2. Land/water management - Invasive/problematic species control

##### Conservation actions

Conservation action type: Needed

Conservation actions: 2.1. Land/water management - Site/area management2.2. Land/water management - Invasive/problematic species control2.3. Land/water management - Habitat & natural process restoration4. Education & awareness5.4.3. Law & policy - Compliance and enforcement - Sub-national level

#### Conservation actions

Conservation action type: In Place

Conservation actions: 1. Land/water protection1.1. Land/water protection - Site/area protection2.2. Land/water management - Invasive/problematic species control

#### Conservation actions

Conservation action type: Needed

Conservation actions: 2.1. Land/water management - Site/area management2.2. Land/water management - Invasive/problematic species control2.3. Land/water management - Habitat & natural process restoration4. Education & awareness5.4.3. Law & policy - Compliance and enforcement - Sub-national level

#### Other

##### Use and trade

Use type: International

##### Ecosystem services

Ecosystem service type: Less important

Ecosystem services: 7. Nutrient Cycling

##### Research needed

Research needed: 1.2. Research - Population size, distribution & trends1.3. Research - Life history & ecology2.2. Conservation Planning - Area-based Management Plan3.1. Monitoring - Population trends3.4. Monitoring - Habitat trends

Justification for research needed: Further research is needed into its ecology and life history in order to find extant specimens in additional fragments of exotic forest in S. Maria and obtain information on population size, distribution and trends. It is also necessary an area-based management plan and a monitoring plan for the invertebrate community in the habitat in order to contribute to perform a species potential recovery plan. Monitoring every ten years using the BALA protocol will inform about habitat quality (see e.g. Gaspar et al. 2011).

#### Use and trade

Use type: International

#### Ecosystem services

Ecosystem service type: Less important

Ecosystem services: 7. Nutrient Cycling

#### Research needed

Research needed: 1.2. Research - Population size, distribution & trends1.3. Research - Life history & ecology2.2. Conservation Planning - Area-based Management Plan3.1. Monitoring - Population trends3.4. Monitoring - Habitat trends

Justification for research needed: Further research is needed into its ecology and life history in order to find extant specimens in additional fragments of exotic forest in S. Maria and obtain information on population size, distribution and trends. It is also necessary an area-based management plan and a monitoring plan for the invertebrate community in the habitat in order to contribute to perform a species potential recovery plan. Monitoring every ten years using the BALA protocol will inform about habitat quality (see e.g. Gaspar et al. 2011).

#### Viability analysis

Justification for probability: 

### Heteroderes azoricus

#### Species information

Scientific name: Heteroderes azoricus

Species authority: (Tarnier, 1860)

Synonyms: *Oophorus azoricus* Tarnier, 1860

Common names: Click beetle (English); Escaravelho-mola (Portuguese)

Kingdom: Animalia

Phylum: Arthropoda

Class: Insecta

Order: Coleoptera

Family: Elateridae

Region for assessment: Global

#### Editor & Reviewers

##### Reviewers

Reviewers: Anja Danielczak

##### Editor

Editor: Axel Hochkirch

#### Reviewers

Reviewers: Anja Danielczak

#### Editor

Editor: Axel Hochkirch

#### Geographic range

Biogeographic realm: Palearctic

Countries: Portugal

Map of records (Google Earth): Suppl. material 29

Basis of EOO and AOO: Observed

Basis (narrative): The extent of occurrence (EOO) is ca 39,000 km^2^ and the maximum area of occupancy (AOO) is 200 km^2^.

Min Elevation/Depth (m): 0

Max Elevation/Depth (m): 500

Range description: *Heteroderes azoricus* is an endemic species occurring in all Azorean islands (Borges et al. 2010), known from the Natural Forest Reserve of Pico Alto in Santa Maria.

#### New occurrences

#### Extent of occurrence

EOO (km2): 39,000

Trend: Decline (inferred)

Justification for trend: The Extent of Occurrence includes large areas of unsuitable habitats. The species keeps a decline trend due to native forest destruction at lower elevations and habitat continuing destruction and fragmentation.

Causes ceased?: No

Causes understood?: Yes

Causes reversible?: Unknown

Extreme fluctuations?: Unknown

#### Area of occupancy

Trend: Decline (inferred)

Justification for trend: The species keeps a decline trend due to native forest destruction and habitat fragmentation at lower elevations.

Causes ceased?: No

Causes understood?: Yes

Causes reversible?: Unknown

Extreme fluctuations?: Unknown

AOO (km2): 200

#### Locations

Number of locations: 35

Justification for number of locations: This is a widespread species that occurs in 35 locations of native and exotic forests and in agricultural landuses of the Flores, Faial, Graciosa, Terceira, S. Miguel and Sta. Maria islands.

Trend: Decline (inferred)

Justification for trend: 35 locations, in which many of them in the last ten years were highly impacted by invasive species spread, exotic forest cut, intensive pasture management and urban development. Possibly some of these locations are not currently adequate for the species.

Extreme fluctuations?: Unknown

#### Population

Trend: Decline (inferred)

Justification for trend: *H. azoricus* is a widespread and particularly abundant species in several habitats. A decline in the population abundance is inferred as a consequence of the spread invasive plant species, exotic forest cut, intensive pasture management and urban development.

Causes ceased?: No

Causes understood?: Yes

Causes reversible?: Unknown

Extreme fluctuations?: Unknown

#### Subpopulations

Number of subpopulations: 9

Trend: Stable

Justification for trend: *H. azoricus* is a widespread and particularly abundant species in several habitats. We assume stability in the number of subpopulations, despite de fact that some subpopulations are under threat.

Extreme fluctuations?: Unknown

Severe fragmentation?: No

#### Habitat

System: Terrestrial

Habitat specialist: No

Habitat (narrative): The species occurs in several habitats, like native forests, exotic forests, lava formations, grasslands and in agricultural land-uses of the Flores, Faial, Graciosa, Terceira, S. Miguel and Sta. Maria islands (Azores). It is widespread by the low elevation habitats in the archipelago (altitudinal range between 0 and 500 m).

Trend in extent, area or quality?: Decline (observed)

Justification for trend: Many of the fragments in the last ten years were highly impacted by invasive species spread, exotic forest cut, intensive pasture management and urban development. Possibly some of the sites are not currently adequate for the species.

##### Habitat

Habitat importance: Major Importance

Habitats: 1.4. Forest - Temperate3.4. Shrubland - Temperate4. Grassland14.2. Artificial/Terrestrial - Pastureland14.3. Artificial/Terrestrial - Plantations16. Introduced vegetation

#### Habitat

Habitat importance: Major Importance

Habitats: 1.4. Forest - Temperate3.4. Shrubland - Temperate4. Grassland14.2. Artificial/Terrestrial - Pastureland14.3. Artificial/Terrestrial - Plantations16. Introduced vegetation

#### Ecology

Size: 0.85-10.5 cm

Generation length (yr): 1

Dependency of single sp?: No

Ecology and traits (narrative): Adults and larvae are herbivores and feed on plant tissues. It is common to find many individuals under the bark of exotic trees. Based on seasonal data from SLAM traps obtained in several islands between 2012 and 2016 (Borges et al. 2017b) most of the adults are active in summer. This is an univoltine species.

#### Threats

Justification for threats: In the past, the species has probably strongly declined due to changes in habitat size and quality (Triantis et al. 2010, Terzopoulou et al. 2015). Currently agriculture activities, *Cryptomeria japonica* plantations as well as invasive plants are promoting dramatic changes in the low elevation habitats. Based on Ferreira et al. 2016 the habitat will further decline as a consequence of climate changes (increasing number of droughts and habitat shifting & alteration).

##### Threats

Threat type: Ongoing

Threats: 2.1.2. Agriculture & aquaculture - Annual & perennial non-timber crops - Small-holder farming2.2.1. Agriculture & aquaculture - Wood & pulp plantations - Small-holder plantations8.1.2. Invasive and other problematic species, genes & diseases - Invasive non-native/alien species/diseases - Named species

##### Threats

Threat type: Future

Threats: 11.1. Climate change & severe weather - Habitat shifting & alteration11.2. Climate change & severe weather - Droughts

#### Threats

Threat type: Ongoing

Threats: 2.1.2. Agriculture & aquaculture - Annual & perennial non-timber crops - Small-holder farming2.2.1. Agriculture & aquaculture - Wood & pulp plantations - Small-holder plantations8.1.2. Invasive and other problematic species, genes & diseases - Invasive non-native/alien species/diseases - Named species

#### Threats

Threat type: Future

Threats: 11.1. Climate change & severe weather - Habitat shifting & alteration11.2. Climate change & severe weather - Droughts

#### Conservation

Justification for conservation actions: The species is not protected by regional law. Its main native habitat is in a regionally protected area (Natural Park of Santa Maria). In the remaining islands the species range is outside protected areas. Further spread of invasive plants needs to be stopped in order to avoid any future declines of the species. Degraded habitats should be restored and a strategy needs to be developed to address the future threat by climate change. It is necessary a monitoring plan for the invertebrate community in the habitat in order to contribute to the conservation of this species. A habitat management plan is needed and anticipated to be developed during the coming years.

##### Conservation actions

Conservation action type: In Place

Conservation actions: 1. Land/water protection1.1. Land/water protection - Site/area protection2.2. Land/water management - Invasive/problematic species control

##### Conservation actions

Conservation action type: Needed

Conservation actions: 2.1. Land/water management - Site/area management2.2. Land/water management - Invasive/problematic species control2.3. Land/water management - Habitat & natural process restoration4. Education & awareness5.4.3. Law & policy - Compliance and enforcement - Sub-national level

#### Conservation actions

Conservation action type: In Place

Conservation actions: 1. Land/water protection1.1. Land/water protection - Site/area protection2.2. Land/water management - Invasive/problematic species control

#### Conservation actions

Conservation action type: Needed

Conservation actions: 2.1. Land/water management - Site/area management2.2. Land/water management - Invasive/problematic species control2.3. Land/water management - Habitat & natural process restoration4. Education & awareness5.4.3. Law & policy - Compliance and enforcement - Sub-national level

#### Other

##### Use and trade

Use type: International

##### Ecosystem services

Ecosystem service type: Less important

Ecosystem services: 7. Nutrient Cycling

##### Research needed

Research needed: 1.2. Research - Population size, distribution & trends1.3. Research - Life history & ecology3.1. Monitoring - Population trends3.4. Monitoring - Habitat trends

Justification for research needed: Further research is needed into its ecology and life history in order to find extant specimens in historical sites and in additional low elevation sites and obtain information on population size, distribution and trends. It is also necessary a monitoring plan for the invertebrate community in the habitat in order to contribute to perform a species potential recovery plan. Monitoring every ten years using the BALA protocol will inform about habitat quality (see e.g. Gaspar et al. 2011).

#### Use and trade

Use type: International

#### Ecosystem services

Ecosystem service type: Less important

Ecosystem services: 7. Nutrient Cycling

#### Research needed

Research needed: 1.2. Research - Population size, distribution & trends1.3. Research - Life history & ecology3.1. Monitoring - Population trends3.4. Monitoring - Habitat trends

Justification for research needed: Further research is needed into its ecology and life history in order to find extant specimens in historical sites and in additional low elevation sites and obtain information on population size, distribution and trends. It is also necessary a monitoring plan for the invertebrate community in the habitat in order to contribute to perform a species potential recovery plan. Monitoring every ten years using the BALA protocol will inform about habitat quality (see e.g. Gaspar et al. 2011).

#### Viability analysis

Justification for probability: 

### Cryptolestes azoricus

#### Species information

Scientific name: Cryptolestes azoricus

Species authority: (Ratti, 1972)

Synonyms: *Leptophloeus azoricus* Ratti, 1972

Kingdom: Animalia

Phylum: Arthropoda

Class: Insecta

Order: Coleoptera

Family: Laemophloeidae

Region for assessment: Global

#### Editor & Reviewers

##### Reviewers

Reviewers: Anja Danielczak

##### Editor

Editor: Axel Hochkirch

#### Reviewers

Reviewers: Anja Danielczak

#### Editor

Editor: Axel Hochkirch

#### Geographic range

Biogeographic realm: Palearctic

Countries: Portugal

Map of records (Google Earth): Suppl. material 30

Basis of EOO and AOO: Observed

Basis (narrative): The area of its remaining native habitat is 0.09 km², but the AOO is 4 km². Its extent of occurrence (EOO) is therefore also 4 km².

Min Elevation/Depth (m): 400

Max Elevation/Depth (m): 550

Range description: *Cryptolestes azoricus* is a single-island endemic species from Santa Maria (Azores, Portugal) (Borges et al. 2010). This species is only known from the original Holotype for which there is no precise locality in the island, but possibly is located in the small remnant of native forest at Pico Alto Natural Forest reserve.

#### New occurrences

#### Extent of occurrence

EOO (km2): 4

Trend: Decline (inferred)

Justification for trend: This species possibly occurs in the only remaining native forest patch from Santa Maria island. There is an inferred continuing decline in EOO due to the spread of invasive plants and observed loss of habitat area in the last 100 years and additional loss of habitat quality in the last 10 years.

Causes ceased?: No

Causes understood?: Yes

Causes reversible?: Unknown

Extreme fluctuations?: Unknown

#### Area of occupancy

Trend: Decline (inferred)

Justification for trend: Based on one locality, the main native forest remaining in the island. There is an inferred continuing decline in AOO due to the spread of invasive plants and observed loss of habitat area in the last 100 years and additional loss of habitat quality in the last 10 years.

Causes ceased?: No

Causes understood?: Yes

Causes reversible?: Unknown

Extreme fluctuations?: Unknown

AOO (km2): 4

#### Locations

Number of locations: 1

Justification for number of locations: The main current native forest that is highly threatened by invasive plants, namely *Hedychium gardnerianum* and *Pittosporum undulatum* that are changing the structure of the forest and the cover of bryophytes and ferns in the soil which will impact the species habitat quality.

Trend: Decline (inferred)

Justification for trend: Only one location of native forest left that has a very low Index of Biotic Integrity (Gaspar et al. 2011) with a area of 0.09 km² and invasive plants can drive this species to extinction very fast The other possibe locations are now with exotic plantations of *Cryptomeria japonica* or pastures.

Extreme fluctuations?: Unknown

#### Population

Trend: Decline (inferred)

Justification for trend: The species is very rare and only known from a single possible sustainable subpopulation. A continuing decline in the number of mature individuals is inferred from recent past deforestation and the ongoing habitat degradation due to invasions of alien plants.

Basis for decline: (c) a decline in area of occupancy, extent of occurrence and/or quality of habitat

Causes ceased?: No

Causes understood?: Yes

Causes reversible?: Unknown

Extreme fluctuations?: Unknown

#### Subpopulations

Number of subpopulations: 1

Trend: Decline (inferred)

Justification for trend: The species is very rare and only known from a single possible sustainable subpopulation that may become extinct due to recent past deforestation and the ongoing habitat degradation due to invasions of alien plants.

Extreme fluctuations?: Unknown

Severe fragmentation?: No

#### Habitat

System: Terrestrial

Habitat specialist: Yes

Habitat (narrative): The species possibly occurs in the single native forests patch of Santa Maria, surrounded by plantations of exotic trees and pastures. This species has an altitudinal range between 400 and 500 m.

Trend in extent, area or quality?: Decline (observed)

Justification for trend: In the last ten years invasive plant species are spreading (e.g. *Hedychium gardnerianum*; *Pittosporum undulatum*) changing the structure of the forest and the cover of bryophytes and ferns in the soil which will impact the species habitat quality

##### Habitat

Habitat importance: Major Importance

Habitats: 1.4. Forest - Temperate

#### Habitat

Habitat importance: Major Importance

Habitats: 1.4. Forest - Temperate

#### Ecology

Generation length (yr): 1

Dependency of single sp?: No

Ecology and traits (narrative): This is a predator that lives under bark of native trees. This is an univoltine species.

#### Threats

Justification for threats: In the past, the species has probably strongly declined due to major deforestation (Triantis et al. 2010). The most important ongoing threats to this species are *Cryptomeria japonica* wood & pulp plantations management andthe spread of invasive plants (*Hedychium gardnerianum* and *Pittosporum undulatum*) that are changing the habitat structure in the main native forest, namely decreasing the cover of bryophytes and ferns in the soil and promoting the spread of other plants. Based on Ferreira et al. 2016 the habitat will decline as a consequence of climate change (increasing number of droughts and habitat shifting & alteration), which may drive this species to extinction, because it is depending on humid forests.

##### Threats

Threat type: Ongoing

Threats: 2.2.1. Agriculture & aquaculture - Wood & pulp plantations - Small-holder plantations8.1.2. Invasive and other problematic species, genes & diseases - Invasive non-native/alien species/diseases - Named species

##### Threats

Threat type: Future

Threats: 11.1. Climate change & severe weather - Habitat shifting & alteration11.2. Climate change & severe weather - Droughts

#### Threats

Threat type: Ongoing

Threats: 2.2.1. Agriculture & aquaculture - Wood & pulp plantations - Small-holder plantations8.1.2. Invasive and other problematic species, genes & diseases - Invasive non-native/alien species/diseases - Named species

#### Threats

Threat type: Future

Threats: 11.1. Climate change & severe weather - Habitat shifting & alteration11.2. Climate change & severe weather - Droughts

#### Conservation

Justification for conservation actions: The species is not protected by regional law. Its habitat is in a regionally protected area (Natural Park of Santa Maria). The Santa Maria Natural Park administration is currently starting control measures of the invasive plants. Further spread of invasive plants needs to be stopped in order to avoid any future declines of the species. Degraded habitats should be restored and a strategy needs to be developed to address the future threat by climate change. A general monitoring scheme for the invertebrate community in the habitat is in place, but the population of this particular species and its habitat needs to be monitored in more detail. A habitat management plan is needed and anticipated to be developed during the coming years.

##### Conservation actions

Conservation action type: In Place

Conservation actions: 1. Land/water protection1.1. Land/water protection - Site/area protection2.2. Land/water management - Invasive/problematic species control

##### Conservation actions

Conservation action type: Needed

Conservation actions: 2.1. Land/water management - Site/area management2.2. Land/water management - Invasive/problematic species control2.3. Land/water management - Habitat & natural process restoration4. Education & awareness5.4.3. Law & policy - Compliance and enforcement - Sub-national level

#### Conservation actions

Conservation action type: In Place

Conservation actions: 1. Land/water protection1.1. Land/water protection - Site/area protection2.2. Land/water management - Invasive/problematic species control

#### Conservation actions

Conservation action type: Needed

Conservation actions: 2.1. Land/water management - Site/area management2.2. Land/water management - Invasive/problematic species control2.3. Land/water management - Habitat & natural process restoration4. Education & awareness5.4.3. Law & policy - Compliance and enforcement - Sub-national level

#### Other

##### Use and trade

Use type: International

##### Ecosystem services

Ecosystem service type: Less important

Ecosystem services: 12. Biocontrol

##### Research needed

Research needed: 1.2. Research - Population size, distribution & trends1.3. Research - Life history & ecology2.2. Conservation Planning - Area-based Management Plan3.1. Monitoring - Population trends3.4. Monitoring - Habitat trends

Justification for research needed: Further research is needed into its ecology and life history in order to find extant specimens in the surrounding areas of Pico Alto (S, Maria) and obtain information on population size, distribution and trends. It is also necessary an area-based management plan and a monitoring plan for the invertebrate community in the habitat in order to contribute to perform a species potential recovery plan. Monitoring every ten years using the BALA protocol will inform about habitat quality (see e.g. Gaspar et al. 2011).

#### Use and trade

Use type: International

#### Ecosystem services

Ecosystem service type: Less important

Ecosystem services: 12. Biocontrol

#### Research needed

Research needed: 1.2. Research - Population size, distribution & trends1.3. Research - Life history & ecology2.2. Conservation Planning - Area-based Management Plan3.1. Monitoring - Population trends3.4. Monitoring - Habitat trends

Justification for research needed: Further research is needed into its ecology and life history in order to find extant specimens in the surrounding areas of Pico Alto (S, Maria) and obtain information on population size, distribution and trends. It is also necessary an area-based management plan and a monitoring plan for the invertebrate community in the habitat in order to contribute to perform a species potential recovery plan. Monitoring every ten years using the BALA protocol will inform about habitat quality (see e.g. Gaspar et al. 2011).

#### Viability analysis

Justification for probability: 

### Metophthalmus occidentalis

#### Species information

Scientific name: Metophthalmus occidentalis

Species authority: Israelson, 1984

Common names: Mould beetle, Minute brown, Plaster beetle, Scavenger beetle (English)

Kingdom: Animalia

Phylum: Arthropoda

Class: Insecta

Order: Coleoptera

Family: Latridiidae

Taxonomic notes: This species was described from two individuals, collected in Santa Maria island (Pico Alto), between 4.VIII.1983 and 6.VIII.1983. These specimens are deposited in G. Israelson and G. Gillerfors collections. The Azorean species differ of the Macaronesian forms by uniform body colour, broader prothorax, non-raised elytral interstriae, considerably smaller size, more convex elytra and/or in details of the upper-side sculpture (Israelson 1984b).

**Figure 22.**
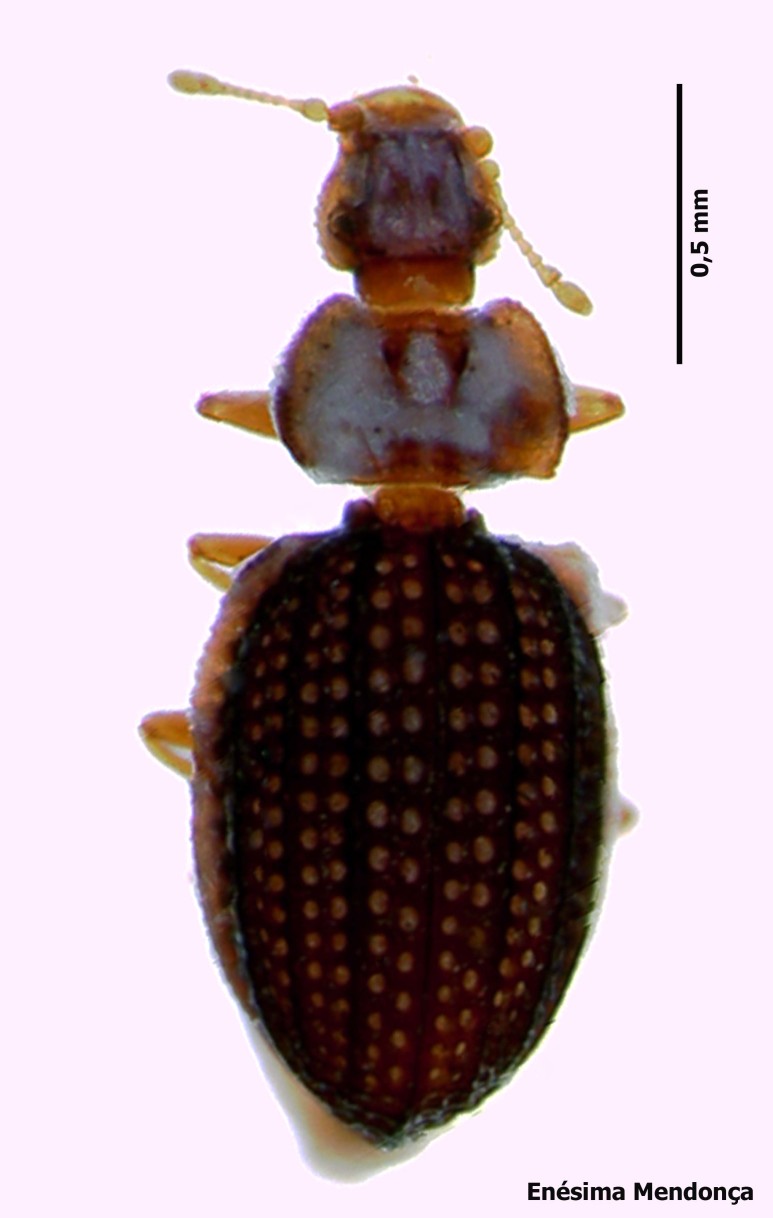
*Metophthalmus occidentalis* from Caldeira da Graciosa (Graciosa, Azores) (Credit: Enésima Mendonça).

Figure(s) or Photo(s): Fig. 22

Region for assessment: Global

#### Editor & Reviewers

##### Reviewers

Reviewers: Anja Danielczak

##### Editor

Editor: Axel Hochkirch

#### Reviewers

Reviewers: Anja Danielczak

#### Editor

Editor: Axel Hochkirch

#### Geographic range

Biogeographic realm: Palearctic

Countries: Portugal

Map of records (Google Earth): Suppl. material 31

Basis of EOO and AOO: Observed

Basis (narrative): The extent of occurrence (EOO) is ca 23,000 km^2^ and the maximum area of occupancy (AOO) is 48 km^2^.

Min Elevation/Depth (m): 100

Max Elevation/Depth (m): 550

Range description: *Metophthalmus occidentalis* is an endemic species present in Faial, Graciosa, S. Miguel and Santa Maria islands (Azores, Portugal) (Borges et al. 2010), known from Natural Forest Reserve of Pico Alto (Santa Maria) and occurring also in some exotic plantations in the other islands.

#### New occurrences

#### Extent of occurrence

EOO (km2): 23,000

Trend: Decline (inferred)

Justification for trend: The Extent of Occurrence includes large areas of unsuitable habitats. The species keeps a decline trend due to native forest destruction and habitat fragmentation.

Causes ceased?: No

Causes understood?: Yes

Causes reversible?: Unknown

Extreme fluctuations?: Unknown

#### Area of occupancy

Trend: Decline (inferred)

Justification for trend: The species occurs in several native and exotic forests of the Faial, Graciosa, S. Miguel and Santa Maria islands. The species continues in decline due to native forest destruction and habitat fragmentation.

Causes ceased?: No

Causes understood?: Yes

Causes reversible?: Unknown

Extreme fluctuations?: Unknown

AOO (km2): 48

#### Locations

Number of locations: 7

Justification for number of locations: The species occurs in seven exotic and native forest patches in the Faial, Graciosa, S. Miguel and Santa Maria islands.

Trend: Decline (inferred)

Justification for trend: Seven locations that were highly impacted by invasive plants in the last ten years, and native forest patch included in a Natural Reserve of Santa Maria island has a very low Index of Biotic Integrity (Gaspar et al. 2011).

Extreme fluctuations?: Unknown

#### Population

Trend: Decline (inferred)

Justification for trend: *M. occidentalis* is a widespread and particularly abundant species in native and exotic forests. A decline in the population abundance is inferred as a consequence of the spread invasive plant species and exotic forest cut.

Causes ceased?: No

Causes understood?: Yes

Causes reversible?: Unknown

Extreme fluctuations?: Unknown

#### Subpopulations

Number of subpopulations: 4

Trend: Stable

Justification for trend: *M. occidentalis* is a widespread and particularly abundant species in native and exotic forests. We assume stability in the number of subpopulations, despite de fact that some subpopulations are under threat.

Extreme fluctuations?: Unknown

Severe fragmentation?: No

#### Habitat

System: Terrestrial

Habitat specialist: No

Habitat (narrative): This species occurs in native forests, exotic forests (dominated by *Pittosporum undulatum*) and *Cryptomeria japonica* plantations in several Azorean islands (Faial, Graciosa, S. Miguel and Santa Maria islands), with an altitudinal range between 100 and 500 m.

Trend in extent, area or quality?: Decline (inferred)

Justification for trend: Many of the fragments in the last ten years were highly impacted by invasive species spread and exotic forest cut.Possibly some of the sites are not currently adequate for the species.

##### Habitat

Habitat importance: Major Importance

Habitats: 1.4. Forest - Temperate14.3. Artificial/Terrestrial - Plantations16. Introduced vegetation

#### Habitat

Habitat importance: Major Importance

Habitats: 1.4. Forest - Temperate14.3. Artificial/Terrestrial - Plantations16. Introduced vegetation

#### Ecology

Generation length (yr): 0

Dependency of single sp?: No

Ecology and traits (narrative): This species is a descomposer of organic matter living in the soil. Based on seasonal data from SLAM traps obtained in several islands between 2012 and 2016 (Borges et al. 2017b), the adults are active all year, being most abundant in spring and summer. This is bivoltine species.

#### Threats

Justification for threats: In the past, the species has probably strongly declined due to changes in habitat size and quality (Triantis et al. 2010). The main current threats are *Cryptomeria japonica* wood & pulp plantations management and the advance of the invasive plant *Hedychium gardnerianum* that is changing the habitat structure, namely decreasing the cover of bryophytes and ferns in the soil and promoting the spread of other plants. Based on Ferreira et al. 2016 the habitat will further decline as a consequence of climate change (increasing number of droughts and habitat shifting & alteration).

##### Threats

Threat type: Ongoing

Threats: 2.2.1. Agriculture & aquaculture - Wood & pulp plantations - Small-holder plantations8.1.2. Invasive and other problematic species, genes & diseases - Invasive non-native/alien species/diseases - Named species

##### Threats

Threat type: Future

Threats: 11.1. Climate change & severe weather - Habitat shifting & alteration11.2. Climate change & severe weather - Droughts

#### Threats

Threat type: Ongoing

Threats: 2.2.1. Agriculture & aquaculture - Wood & pulp plantations - Small-holder plantations8.1.2. Invasive and other problematic species, genes & diseases - Invasive non-native/alien species/diseases - Named species

#### Threats

Threat type: Future

Threats: 11.1. Climate change & severe weather - Habitat shifting & alteration11.2. Climate change & severe weather - Droughts

#### Conservation

Justification for conservation actions: The species is not protected by regional law. Its habitat is in regionally protected areas (Natural Parks of S. Miguel and Santa Maria). Degraded habitats should be restored and a strategy needs to be developed to address the future threat by climate change. It is necessary a monitoring plan for the invertebrate community in the habitat in order to contribute to the conservation of this species. A habitat management plan is needed and anticipated to be developed during the coming years.

##### Conservation actions

Conservation action type: In Place

Conservation actions: 1. Land/water protection1.1. Land/water protection - Site/area protection

##### Conservation actions

Conservation action type: Needed

Conservation actions: 2.1. Land/water management - Site/area management2.2. Land/water management - Invasive/problematic species control2.3. Land/water management - Habitat & natural process restoration4. Education & awareness5.4.3. Law & policy - Compliance and enforcement - Sub-national level

#### Conservation actions

Conservation action type: In Place

Conservation actions: 1. Land/water protection1.1. Land/water protection - Site/area protection

#### Conservation actions

Conservation action type: Needed

Conservation actions: 2.1. Land/water management - Site/area management2.2. Land/water management - Invasive/problematic species control2.3. Land/water management - Habitat & natural process restoration4. Education & awareness5.4.3. Law & policy - Compliance and enforcement - Sub-national level

#### Other

##### Use and trade

Use type: International

##### Ecosystem services

Ecosystem service type: Less important

Ecosystem services: 7. Nutrient Cycling

##### Research needed

Research needed: 1.2. Research - Population size, distribution & trends1.3. Research - Life history & ecology3.1. Monitoring - Population trends3.4. Monitoring - Habitat trends

Justification for research needed: Further research is needed into its ecology and life history in order to find extant specimens in additional low elevaton sites in several islands and obtain information on population size, distribution and trends. It is also necessary a monitoring plan for the invertebrate community in the habitat in order to contribute to perform a species potential recovery plan. Monitoring every ten years using the BALA protocol will inform about habitat quality (see e.g. Gaspar et al. 2011).

#### Use and trade

Use type: International

#### Ecosystem services

Ecosystem service type: Less important

Ecosystem services: 7. Nutrient Cycling

#### Research needed

Research needed: 1.2. Research - Population size, distribution & trends1.3. Research - Life history & ecology3.1. Monitoring - Population trends3.4. Monitoring - Habitat trends

Justification for research needed: Further research is needed into its ecology and life history in order to find extant specimens in additional low elevaton sites in several islands and obtain information on population size, distribution and trends. It is also necessary a monitoring plan for the invertebrate community in the habitat in order to contribute to perform a species potential recovery plan. Monitoring every ten years using the BALA protocol will inform about habitat quality (see e.g. Gaspar et al. 2011).

#### Viability analysis

Justification for probability: 

### Catops velhocabrali

#### Species information

Scientific name: Catops velhocabrali

Species authority: Blas & Borges, 1998

Common names: Azorean small scavenger beetle (English)

Kingdom: Animalia

Phylum: Arthropoda

Class: Insecta

Order: Coleoptera

Family: Leiodidae

Taxonomic notes: *Catops velhocabrali* was described from a single male collected in Santa Maria island, from 12 to 18.VI.1990 (holotype). It is deposited in the University of Azores in Terceira ("Dalberto Teixeira Pombo collection"). *Catops velhocabrali* differs from its probable nearest *C. thurepalmi* and *C. antoniomachado*i from the Canaries, by having a stronger and more convex general appearance and a less transverse pronotum. It also differs in the antennae, maxillary palpus and aedeagus (Blas and Borges 1999).

Region for assessment: Global

#### Editor & Reviewers

##### Reviewers

Reviewers: Anja Danielczak

##### Editor

Editor: Axel Hochkirch

#### Reviewers

Reviewers: Anja Danielczak

#### Editor

Editor: Axel Hochkirch

#### Geographic range

Biogeographic realm: Palearctic

Countries: Portugal

Map of records (Google Earth): Suppl. material 32

Basis of EOO and AOO: Observed

Basis (narrative): The extent of occurrence (EOO) is 12 km^2^ and the maximum area of occupancy (AOO) is 12 km^2^.

Min Elevation/Depth (m): 450

Max Elevation/Depth (m): 550

Range description: *Catops velhocabrali* is a single-island endemic species from Santa Maria (Azores, Portugal) (Borges et al. 2010), known from Natural Forest Reserve of Pico Alto.

#### New occurrences

#### Extent of occurrence

EOO (km2): 12

Trend: Decline (inferred)

Justification for trend: This species occurs in native and exotic forest patches and geological formations of Santa Maria island. The species continues in decline due to native forest destruction, habitat fragmentation and degradation of the geologic formations.

Causes ceased?: No

Causes understood?: Yes

Causes reversible?: Unknown

Extreme fluctuations?: Unknown

#### Area of occupancy

Trend: Decline (inferred)

Justification for trend: The species occurs in native and exotic forests and geological formations of the Santa Maria islands. The area of its remaining native habitat is now only 0.09 km². The species continues in decline due to native forest destruction, habitat fragmentation and degradation of the geologic formations.

Causes ceased?: No

Causes understood?: Yes

Causes reversible?: Unknown

Extreme fluctuations?: Unknown

AOO (km2): 12

#### Locations

Number of locations: 1

Justification for number of locations: This species occurs in native and exotic forest patches and geological formations at Pico Alto Santa Maria island.

Trend: Decline (inferred)

Justification for trend: One location included in a Natural Reserve of Santa Maria island that has a very low Index of Biotic Integrity (Gaspar et al. 2011) as a consequence of a dramatic spread of invasive plants (e.g. *Hedychium gardnerianum*; *Pittosporum undulatum*), that are changing the structure of the forest and the cover of bryophytes and ferns in the soil decreasing the quality of the habitat with impacts on the species.

Extreme fluctuations?: Unknown

#### Population

Trend: Decline (inferred)

Justification for trend: The species is very rare and only known from a single subpopulation in Santa Maria island in native forest patch included in a Natural Reserve (Pico Alto) that has a very low Index of Biotic Integrity (Gaspar et al. 2011). A continuing decline in the number of mature individuals is inferred due to human activities (associated with agriculture and cattle pollution), a small subpopulation, small patches and by the expansion of alien plants.

Basis for decline: (c) a decline in area of occupancy, extent of occurrence and/or quality of habitat

Causes ceased?: No

Causes understood?: Yes

Causes reversible?: Unknown

Extreme fluctuations?: Unknown

#### Subpopulations

Number of subpopulations: 1

Trend: Decline (inferred)

Justification for trend: The species is very rare and only known from a single subpopulation in Santa Maria island. A continuing decline in the number of subpopulations is inferred due to human activities (associated with agriculture and cattle pollution), small patches and by the expansion of alien plants.

Extreme fluctuations?: Unknown

Severe fragmentation?: No

#### Habitat

System: Terrestrial

Habitat specialist: Yes

Habitat (narrative): This species occurs in different habitats: native forests, *Cryptomeria japonica* plantations and MSS - Mesocavernous Shallow Stratum in Santa Maria island (Blas and Borges 1999). This species has an altitudinal range between 450 and 550 m.

Trend in extent, area or quality?: Decline (observed)

Justification for trend: Ongoing invasion of exotic plants that are changing the structure of the forest and the cover of bryophytes and ferns in the soil with impacts on the species.

##### Habitat

Habitat importance: Major Importance

Habitats: 1.4. Forest - Temperate7.1. Caves and Subterranean Habitats (non-aquatic) - Caves7.2. Caves and Subterranean Habitats (non-aquatic) - Other Subterranean Habitats14.3. Artificial/Terrestrial - Plantations16. Introduced vegetation

#### Habitat

Habitat importance: Major Importance

Habitats: 1.4. Forest - Temperate7.1. Caves and Subterranean Habitats (non-aquatic) - Caves7.2. Caves and Subterranean Habitats (non-aquatic) - Other Subterranean Habitats14.3. Artificial/Terrestrial - Plantations16. Introduced vegetation

#### Ecology

Size: 0.35 cm

Generation length (yr): 1

Dependency of single sp?: No

Ecology and traits (narrative): This is a decomposer of organic matter (saprophagous) with night activity. This is an univoltine species.

#### Threats

Justification for threats: In the past, the species has probably strongly declined due to changes in habitat size and quality (Terzopoulou et al. 2015, Triantis et al. 2010). Currently the most important ongoing threats to this species are *Cryptomeria japonica* wood & pulp plantations management and the spread of invasive plants namely *Pittosporum undulatum* and *Hedychium gardnerianum* since are changing the habitat structure, namely decreasing the cover of bryophytes and ferns in the soil and promoting the spread of other plants. Based on Ferreira et al. 2016 the habitat will further decline as a consequence of climate change (increasing number of droughts and habitat shifting & alteration).

##### Threats

Threat type: Ongoing

Threats: 2.2.1. Agriculture & aquaculture - Wood & pulp plantations - Small-holder plantations8.1.2. Invasive and other problematic species, genes & diseases - Invasive non-native/alien species/diseases - Named species

##### Threats

Threat type: Future

Threats: 11.1. Climate change & severe weather - Habitat shifting & alteration11.2. Climate change & severe weather - Droughts

#### Threats

Threat type: Ongoing

Threats: 2.2.1. Agriculture & aquaculture - Wood & pulp plantations - Small-holder plantations8.1.2. Invasive and other problematic species, genes & diseases - Invasive non-native/alien species/diseases - Named species

#### Threats

Threat type: Future

Threats: 11.1. Climate change & severe weather - Habitat shifting & alteration11.2. Climate change & severe weather - Droughts

#### Conservation

Justification for conservation actions: The species is not protected by regional law. Its habitat is in a regionally protected area (Natural Park of Santa Maria). The Santa Maria Natural Park administration is currently starting control measures of the invasive plants. Further spread of invasive plants needs to be stopped in order to avoid any future declines of the species. Degraded habitats should be restored and a strategy needs to be developed to address the future threat by climate change. It is necessary a monitoring plan for the invertebrate community in the MSS habitat in order to contribute to the conservation of this species. A habitat management plan is needed and anticipated to be developed during the coming years.

##### Conservation actions

Conservation action type: In Place

Conservation actions: 1. Land/water protection1.1. Land/water protection - Site/area protection2. Land/water management2.2. Land/water management - Invasive/problematic species control

##### Conservation actions

Conservation action type: Needed

Conservation actions: 2.1. Land/water management - Site/area management2.2. Land/water management - Invasive/problematic species control2.3. Land/water management - Habitat & natural process restoration4. Education & awareness5.4.3. Law & policy - Compliance and enforcement - Sub-national level

#### Conservation actions

Conservation action type: In Place

Conservation actions: 1. Land/water protection1.1. Land/water protection - Site/area protection2. Land/water management2.2. Land/water management - Invasive/problematic species control

#### Conservation actions

Conservation action type: Needed

Conservation actions: 2.1. Land/water management - Site/area management2.2. Land/water management - Invasive/problematic species control2.3. Land/water management - Habitat & natural process restoration4. Education & awareness5.4.3. Law & policy - Compliance and enforcement - Sub-national level

#### Other

##### Use and trade

Use type: International

##### Ecosystem services

Ecosystem service type: Less important

Ecosystem services: 7. Nutrient Cycling

##### Research needed

Research needed: 1.2. Research - Population size, distribution & trends1.3. Research - Life history & ecology2.2. Conservation Planning - Area-based Management Plan3.1. Monitoring - Population trends3.4. Monitoring - Habitat trends

Justification for research needed: Further research is needed into its ecology and life history in order to find extant specimens in new sites with MSS habitat and obtain information on population size, distribution and trends. It is also necessary a monitoring plan for the invertebrate community in the habitat in order to contribute to perform a species potential recovery plan. Monitoring every ten years using the BALA protocol will inform about habitat quality (see e.g. Gaspar et al. 2011).

#### Use and trade

Use type: International

#### Ecosystem services

Ecosystem service type: Less important

Ecosystem services: 7. Nutrient Cycling

#### Research needed

Research needed: 1.2. Research - Population size, distribution & trends1.3. Research - Life history & ecology2.2. Conservation Planning - Area-based Management Plan3.1. Monitoring - Population trends3.4. Monitoring - Habitat trends

Justification for research needed: Further research is needed into its ecology and life history in order to find extant specimens in new sites with MSS habitat and obtain information on population size, distribution and trends. It is also necessary a monitoring plan for the invertebrate community in the habitat in order to contribute to perform a species potential recovery plan. Monitoring every ten years using the BALA protocol will inform about habitat quality (see e.g. Gaspar et al. 2011).

#### Viability analysis

Justification for probability: 

### Aleochara freyi

#### Species information

Scientific name: Aleochara freyi

Species authority: Bernhauer 1940

Common names: Rove beetle (English)

Kingdom: Animalia

Phylum: Arthropoda

Class: Insecta

Order: Coleoptera

Family: Staphylinidae

Region for assessment: Global

#### Editor & Reviewers

##### Reviewers

Reviewers: Anja Danielczak

##### Editor

Editor: Axel Hochkirch

#### Reviewers

Reviewers: Anja Danielczak

#### Editor

Editor: Axel Hochkirch

#### Geographic range

Biogeographic realm: Palearctic

Countries: Portugal

Map of records (Google Earth): Suppl. material 33

Basis of EOO and AOO: Observed

Basis (narrative): The extent of occurrence (EOO) is 8 km^2^ and the maximum area of occupancy (AOO) is 8 km^2^.

Min Elevation/Depth (m): 1200

Max Elevation/Depth (m): 1800

Range description: *Aleochara freyi* is single-island endemic species from Pico (Azores, Portugal) (Borges et al. 2010), known from Pico mountain protected area.

#### New occurrences

#### Extent of occurrence

EOO (km2): 8

Trend: Decline (inferred)

Justification for trend: This species occurs in a fragment of native forest of Pico island (Montanha do Pico). Possibly the EOO value is slightly overestimated. The species continues in decline due to native forest destruction and habitat fragmentation, with creation of pastures.

Causes ceased?: No

Causes understood?: Yes

Causes reversible?: Unknown

Extreme fluctuations?: Unknown

#### Area of occupancy

Trend: Decline (inferred)

Justification for trend: This species occurs in a fragment of native forest of Pico island (Montanha do Pico). Possibly the AOO value is slightly overestimated. The species continues in decline due to native forest destruction and habitat fragmentation, with creation of pastures.

Causes ceased?: No

Causes understood?: Yes

Causes reversible?: Unknown

Extreme fluctuations?: Unknown

AOO (km2): 8

#### Locations

Number of locations: 1

Justification for number of locations: This species occurs in one single native forest patch in Pico island (Montanha do Pico).

Trend: Decline (inferred)

Justification for trend: Only one location left, and the site is under disturbance due to cattle grazing in high altitude semi-natural pastures.

Extreme fluctuations?: Unknown

#### Population

Trend: Decline (inferred)

Justification for trend: The species is only known from a single subpopulation in Pico island. A continuing decline in the number of mature individuals is inferred from historical records. This species can be on the edge of extinction due to major historical changes in its type locality.

Basis for decline: (c) a decline in area of occupancy, extent of occurrence and/or quality of habitat

Causes ceased?: No

Causes understood?: Yes

Causes reversible?: Unknown

Extreme fluctuations?: Unknown

#### Subpopulations

Number of subpopulations: 1

Trend: Decline (inferred)

Justification for trend: The species is only known from a single subpopulation in Pico island. A continuing decline in the number of subpopulations is inferred from historical records.

Extreme fluctuations?: Unknown

Severe fragmentation?: No

#### Habitat

System: Terrestrial

Habitat specialist: Yes

Habitat (narrative): This species occurs in one single native forest patch (dominated by *Juniperus brevifolia* and *Erica azorica*), located at high altitude, in Pico island (Montanha do Pico).

Trend in extent, area or quality?: Decline (observed)

Justification for trend: Destruction of habitat for creation of pastures a trend that still occurs.

##### Habitat

Habitat importance: Major Importance

Habitats: 1.4. Forest - Temperate3.4. Shrubland - Temperate

#### Habitat

Habitat importance: Major Importance

Habitats: 1.4. Forest - Temperate3.4. Shrubland - Temperate

#### Ecology

Size: 0.33-0.4 cm

Generation length (yr): 1

Dependency of single sp?: No

Ecology and traits (narrative): This is a nocturnal predator that lives under bark of native trees and in the soil. This is an univoltine species.

#### Threats

Justification for threats: In the past, the species has probably strongly declined due to changes in habitat size and quality, mostly creation of pastures (Triantis et al. 2010). One of the most important ongoing threat to this species is the high elevation dairy cattle and meat cattle semi-natural pastures and the spread of invasive plants namely *Hedychium gardnerianum* since are changing the habitat structure, namely decreasing the cover of bryophytes and ferns in the soil and promoting the spread of other plants. Based on Ferreira et al. 2016) the habitat will further decline as a consequence of climate change (increasing number of droughts and habitat shifting & alteration).

##### Threats

Threat type: Ongoing

Threats: 2.3.2. Agriculture & aquaculture - Livestock farming & ranching - Small-holder grazing, ranching or farming8.1.2. Invasive and other problematic species, genes & diseases - Invasive non-native/alien species/diseases - Named species

##### Threats

Threat type: Future

Threats: 11.1. Climate change & severe weather - Habitat shifting & alteration11.2. Climate change & severe weather - Droughts

#### Threats

Threat type: Ongoing

Threats: 2.3.2. Agriculture & aquaculture - Livestock farming & ranching - Small-holder grazing, ranching or farming8.1.2. Invasive and other problematic species, genes & diseases - Invasive non-native/alien species/diseases - Named species

#### Threats

Threat type: Future

Threats: 11.1. Climate change & severe weather - Habitat shifting & alteration11.2. Climate change & severe weather - Droughts

#### Conservation

Justification for conservation actions: The species is not protected by regional law. Its habitat is in a regionally protected area (Natural Park of Pico; Reserva Natural da Montanha do Pico). Degraded habitats should be restored and a strategy needs to be developed to address the future threat by climate change. It is necessary a monitoring plan for the invertebrate community in the habitat in order to contribute to the conservation of this species. A habitat management plan is needed and anticipated to be developed during the coming years.

##### Conservation actions

Conservation action type: In Place

Conservation actions: 1. Land/water protection1.1. Land/water protection - Site/area protection2.2. Land/water management - Invasive/problematic species control

##### Conservation actions

Conservation action type: Needed

Conservation actions: 2.1. Land/water management - Site/area management2.2. Land/water management - Invasive/problematic species control2.3. Land/water management - Habitat & natural process restoration4. Education & awareness5.4.3. Law & policy - Compliance and enforcement - Sub-national level

#### Conservation actions

Conservation action type: In Place

Conservation actions: 1. Land/water protection1.1. Land/water protection - Site/area protection2.2. Land/water management - Invasive/problematic species control

#### Conservation actions

Conservation action type: Needed

Conservation actions: 2.1. Land/water management - Site/area management2.2. Land/water management - Invasive/problematic species control2.3. Land/water management - Habitat & natural process restoration4. Education & awareness5.4.3. Law & policy - Compliance and enforcement - Sub-national level

#### Other

##### Use and trade

Use type: International

##### Ecosystem services

Ecosystem service type: Less important

Ecosystem services: 12. Biocontrol

##### Research needed

Research needed: 1.2. Research - Population size, distribution & trends1.3. Research - Life history & ecology2.2. Conservation Planning - Area-based Management Plan3.1. Monitoring - Population trends3.4. Monitoring - Habitat trends

Justification for research needed: Further research is needed into its ecology and life history in order to find extant specimens in the high elevation semi-natural pastures of Pico island and obtain information on population size, distribution and trends. It is also necessary a monitoring plan for the invertebrate community in the habitat in order to contribute to perform a species potential recovery plan. Monitoring every ten years using the BALA protocol will inform about habitat quality (see e.g. Gaspar et al. 2011).

#### Use and trade

Use type: International

#### Ecosystem services

Ecosystem service type: Less important

Ecosystem services: 12. Biocontrol

#### Research needed

Research needed: 1.2. Research - Population size, distribution & trends1.3. Research - Life history & ecology2.2. Conservation Planning - Area-based Management Plan3.1. Monitoring - Population trends3.4. Monitoring - Habitat trends

Justification for research needed: Further research is needed into its ecology and life history in order to find extant specimens in the high elevation semi-natural pastures of Pico island and obtain information on population size, distribution and trends. It is also necessary a monitoring plan for the invertebrate community in the habitat in order to contribute to perform a species potential recovery plan. Monitoring every ten years using the BALA protocol will inform about habitat quality (see e.g. Gaspar et al. 2011).

#### Viability analysis

Justification for probability: 

### Atheta azorica

#### Species information

Scientific name: Atheta azorica

Species authority: Bernhauer, 1936

Common names: Rove beetle (English)

Kingdom: Animalia

Phylum: Arthropoda

Class: Insecta

Order: Coleoptera

Family: Staphylinidae

Region for assessment: Global

#### Editor & Reviewers

##### Reviewers

Reviewers: Anja Danielczak

##### Editor

Editor: Axel Hochkirch

#### Reviewers

Reviewers: Anja Danielczak

#### Editor

Editor: Axel Hochkirch

#### Geographic range

Biogeographic realm: Palearctic

Countries: Portugal

Map of records (Google Earth): Suppl. material 34

Basis of EOO and AOO: Observed

Basis (narrative): The historical unknown locality is assumed as one 2 km x 2 km cell.

Range description: *Atheta azorica* was described for Azores but without indication of the island of occurrence. It was never found after its description and consequently there is no precise indication of its locality.

#### New occurrences

#### Extent of occurrence

EOO (km2): 4

Trend: Unknown

Justification for trend: The historical unknown locality is assumed as one 2 km x 2 km cell.

Causes ceased?: Unknown

Causes understood?: Unknown

Causes reversible?: Unknown

Extreme fluctuations?: Unknown

#### Area of occupancy

Trend: Unknown

Justification for trend: *Atheta azorica* was described for Azores but without indication of the island of occurrence. It was never found after its description and consequently there is no precise indication of its locality.

Causes ceased?: Unknown

Causes understood?: Unknown

Causes reversible?: Unknown

Extreme fluctuations?: Unknown

AOO (km2): 4

#### Locations

Number of locations: 1

Justification for number of locations: The historical unknown precise location.

Trend: Unknown

Extreme fluctuations?: Unknown

#### Population

Trend: Unknown

Justification for trend: There is no information available.

Causes ceased?: Unknown

Causes understood?: Unknown

Causes reversible?: Unknown

Extreme fluctuations?: Unknown

#### Subpopulations

Trend: Unknown

Justification for trend: There is no information available.

Extreme fluctuations?: Unknown

Severe fragmentation?: No

#### Habitat

System: Terrestrial

Habitat specialist: Yes

Habitat (narrative): This species is originally associated with native forest.

Trend in extent, area or quality?: Unknown

##### Habitat

Habitat importance: Major Importance

Habitats: 1.4. Forest - Temperate

#### Habitat

Habitat importance: Major Importance

Habitats: 1.4. Forest - Temperate

#### Ecology

Generation length (yr): 1

Dependency of single sp?: Unknown

Ecology and traits (narrative): This is a predator species originally associated with native forest, but with unknown current distribution and ecology.

#### Threats

Justification for threats: In the past, the species has probably strongly declined due to changes in habitat size and quality, mostly creation of pastures (Triantis et al. 2010). The most important ongoing threats to this species are the managment of pulp plantations of *Cryptomeria japonica* and the spread of invasive plants namely *Hedychium gardnerianum* since are changing the habitat structure, namely decreasing the cover of bryophytes and ferns in the soil and promoting the spread of other plants. Based on Ferreira et al. 2016 the habitat will further decline as a consequence of climate change (increasing number of droughts a nd habitat shifting & alteration).

##### Threats

Threat type: Ongoing

Threats: 2.2.1. Agriculture & aquaculture - Wood & pulp plantations - Small-holder plantations8.1.2. Invasive and other problematic species, genes & diseases - Invasive non-native/alien species/diseases - Named species

##### Threats

Threat type: Future

Threats: 11.1. Climate change & severe weather - Habitat shifting & alteration11.2. Climate change & severe weather - Droughts

#### Threats

Threat type: Ongoing

Threats: 2.2.1. Agriculture & aquaculture - Wood & pulp plantations - Small-holder plantations8.1.2. Invasive and other problematic species, genes & diseases - Invasive non-native/alien species/diseases - Named species

#### Threats

Threat type: Future

Threats: 11.1. Climate change & severe weather - Habitat shifting & alteration11.2. Climate change & severe weather - Droughts

#### Conservation

Justification for conservation actions: The species is not protected by regional law. No information is available on its location and consequently there are no conservation measures planned.

##### Conservation actions

Conservation action type: Needed

#### Conservation actions

Conservation action type: Needed

#### Other

##### Use and trade

Use type: International

##### Ecosystem services

Ecosystem service type: Less important

Ecosystem services: 12. Biocontrol

##### Research needed

Research needed: 1.2. Research - Population size, distribution & trends1.3. Research - Life history & ecology

Justification for research needed: Further research is needed into its ecology and life history in order to find extant specimens in at least one of the islands and obtain information on population size, distribution and trends.

#### Use and trade

Use type: International

#### Ecosystem services

Ecosystem service type: Less important

Ecosystem services: 12. Biocontrol

#### Research needed

Research needed: 1.2. Research - Population size, distribution & trends1.3. Research - Life history & ecology

Justification for research needed: Further research is needed into its ecology and life history in order to find extant specimens in at least one of the islands and obtain information on population size, distribution and trends.

#### Viability analysis

Justification for probability: 

### Atheta caprariensis

#### Species information

Scientific name: Atheta caprariensis

Species authority: Israelson, 1985

Common names: Rove beetle (English)

Kingdom: Animalia

Phylum: Arthropoda

Class: Insecta

Order: Coleoptera

Family: Staphylinidae

Taxonomic notes: *Atheta caprariensis* was described from individuals collected in S. Miguel island (Furnas), between 28.VII.1983 and 31.VII.1984. These individuals are deposited in G. Israelson and G. Gillerfors collections. This species is closely related with species from the subgenera *Notothecta* and *Atheta*-complex, but amply distinguished by its larger size among several other characters. The inner armature of the penis of the present species seems to be rather weak for a member of this subgenus but may have been reduced, still more so in the following species (Israelson 1985b).

Region for assessment: Global

#### Editor & Reviewers

##### Reviewers

Reviewers: Anja Danielczak

##### Editor

Editor: Axel Hochkirch

#### Reviewers

Reviewers: Anja Danielczak

#### Editor

Editor: Axel Hochkirch

#### Geographic range

Biogeographic realm: Palearctic

Countries: Portugal

Map of records (Google Earth): Suppl. material 35

Basis of EOO and AOO: Observed

Basis (narrative): The extent of occurrence (EOO) is 8 km^2^ and the maximum area of occupancy (AOO) is 8 km^2^.

Min Elevation/Depth (m): 500

Max Elevation/Depth (m): 600

Range description: *Atheta caprariensis* is a single-island endemic species from S. Miguel (Azores, Portugal) (Borges et al. 2010), known from Furnas region.

#### New occurrences

#### Extent of occurrence

EOO (km2): 8

Trend: Decline (inferred)

Justification for trend: This species occurs in modified habitats at S. Miguel island (Furnas). Possibly the EOO value is slightly overestimated. The species continues in decline due to native forest destruction and habitat fragmentation.

Causes ceased?: No

Causes understood?: Yes

Causes reversible?: Unknown

Extreme fluctuations?: Unknown

#### Area of occupancy

Trend: Decline (inferred)

Justification for trend: This species occurs in a fragment of exotic forest of S. Miguel island (Furnas). Possibly the AOO value is slightly overestimated due to urbanization and pasture intensification. The species continues in decline due to native forest destruction and habitat fragmentation. The current habitat is highly disturbed.

Causes ceased?: No

Causes understood?: Yes

Causes reversible?: Unknown

Extreme fluctuations?: Unknown

AOO (km2): 8

#### Locations

Number of locations: 1

Justification for number of locations: This species occurs in one single native forest patch in S. Miguel island (Furnas).

Trend: Decline (inferred)

Justification for trend: In the last 50 years major alterations were made in the territory with impacts in native habitats. Only one site left with additional major changes in the last 10 years with the creation of a public park around Furnas Lake.

Extreme fluctuations?: Unknown

#### Population

Trend: Decline (inferred)

Justification for trend: The species is only known from a single subpopulations in S. Miguel island. The abundance is unknown and possibly decreasing due to the impact of major urban, forestry and agriculture changes in the historical locality.

Causes ceased?: No

Causes understood?: Yes

Causes reversible?: Unknown

Extreme fluctuations?: Unknown

#### Subpopulations

Number of subpopulations: 1

Trend: Decline (inferred)

Justification for trend: The species is only known from a single subpopulations in S. Miguel island. A decline in the number of is unknown and possibly decreasing due to major urban, forestry and agriculture changes.

Extreme fluctuations?: Unknown

Severe fragmentation?: No

#### Habitat

System: Terrestrial

Habitat specialist: Yes

Habitat (narrative): This species occurs in one single exotic forest patch in S. Miguel island (Furnas) (Israelson 1985), with an altitudinal range between 500 and 600 m.

Trend in extent, area or quality?: Decline (observed)

Justification for trend: Destruction of habitat for creation of urban areas, industrial *Cryptomeria japonica* plantations and pastures.

##### Habitat

Habitat importance: Major Importance

Habitats: 1.4. Forest - Temperate14.3. Artificial/Terrestrial - Plantations

#### Habitat

Habitat importance: Major Importance

Habitats: 1.4. Forest - Temperate14.3. Artificial/Terrestrial - Plantations

#### Ecology

Size: 0.26-0.33

Generation length (yr): 1

Dependency of single sp?: No

Ecology and traits (narrative): It is a nocturnal predator that lives under bark of native and exotic trees and in the soil. This is an univoltine species.

#### Threats

Justification for threats: In the past, the species has probably strongly declined due to changes in habitat size and quality (Terzopoulou et al. 2015, Triantis et al. 2010). Currently the main threat is the major changes in habitats for urban use, industrial plantations of *Cryptomeria japonica* and pastures, but also the spread of invasive plants namely *Hedychium gardnerianum* since are changing the habitat structure, namely decreasing the cover of bryophytes and ferns in the soil and promoting the spread of other plants. Based on Ferreira et al. 2016 the habitat will further decline as a consequence of climate change (increasing number of droughts and habitat shifting & alteration).

##### Threats

Threat type: Ongoing

Threats: 2.2.1. Agriculture & aquaculture - Wood & pulp plantations - Small-holder plantations8.1.2. Invasive and other problematic species, genes & diseases - Invasive non-native/alien species/diseases - Named species12. Other options - Other threat

##### Threats

Threat type: Future

Threats: 11.1. Climate change & severe weather - Habitat shifting & alteration11.2. Climate change & severe weather - Droughts

#### Threats

Threat type: Ongoing

Threats: 2.2.1. Agriculture & aquaculture - Wood & pulp plantations - Small-holder plantations8.1.2. Invasive and other problematic species, genes & diseases - Invasive non-native/alien species/diseases - Named species12. Other options - Other threat

#### Threats

Threat type: Future

Threats: 11.1. Climate change & severe weather - Habitat shifting & alteration11.2. Climate change & severe weather - Droughts

#### Conservation

Justification for conservation actions: The species is not protected by regional law. Its habitat is in a degraded area that should be restored and a strategy needs to be developed to address the future threat by climate change. It is necessary a monitoring plan for the invertebrate community in the habitat in order to contribute to the conservation of this species. A habitat management plan is needed and anticipated to be developed during the coming years.

##### Conservation actions

Conservation action type: Needed

Conservation actions: 1.1. Land/water protection - Site/area protection2.1. Land/water management - Site/area management2.2. Land/water management - Invasive/problematic species control2.3. Land/water management - Habitat & natural process restoration4. Education & awareness5.4.3. Law & policy - Compliance and enforcement - Sub-national level

#### Conservation actions

Conservation action type: Needed

Conservation actions: 1.1. Land/water protection - Site/area protection2.1. Land/water management - Site/area management2.2. Land/water management - Invasive/problematic species control2.3. Land/water management - Habitat & natural process restoration4. Education & awareness5.4.3. Law & policy - Compliance and enforcement - Sub-national level

#### Other

##### Use and trade

Use type: International

##### Ecosystem services

Ecosystem service type: Less important

Ecosystem services: 12. Biocontrol

##### Research needed

Research needed: 1.2. Research - Population size, distribution & trends1.3. Research - Life history & ecology2.2. Conservation Planning - Area-based Management Plan3.1. Monitoring - Population trends3.4. Monitoring - Habitat trends

Justification for research needed: Further research is needed into its ecology and life history in order to find extant specimens in Furnas and surrounded areas and obtain information on population size, distribution and trends. It is also necessary a area-based management plan and a monitoring plan for the invertebrate community in the habitat in order to contribute to perform a species potential recovery plan. Monitoring every ten years using the BALA protocol will inform about habitat quality (see e.g. Gaspar et al. 2011).

#### Use and trade

Use type: International

#### Ecosystem services

Ecosystem service type: Less important

Ecosystem services: 12. Biocontrol

#### Research needed

Research needed: 1.2. Research - Population size, distribution & trends1.3. Research - Life history & ecology2.2. Conservation Planning - Area-based Management Plan3.1. Monitoring - Population trends3.4. Monitoring - Habitat trends

Justification for research needed: Further research is needed into its ecology and life history in order to find extant specimens in Furnas and surrounded areas and obtain information on population size, distribution and trends. It is also necessary a area-based management plan and a monitoring plan for the invertebrate community in the habitat in order to contribute to perform a species potential recovery plan. Monitoring every ten years using the BALA protocol will inform about habitat quality (see e.g. Gaspar et al. 2011).

#### Viability analysis

Justification for probability: 

### Atheta dryochares

#### Species information

Scientific name: Atheta dryochares

Species authority: Israelson, 1985

Common names: Rove beetle (English); Escaravelho-de-asa-curta (Portuguese)

Kingdom: Animalia

Phylum: Arthropoda

Class: Insecta

Order: Coleoptera

Family: Staphylinidae

Taxonomic notes: This species was described from individuals collected in S. Miguel island (Furnas; Ponta Delgada) between 11.VII.1982 and 31.VII.1984. These specimens are deposited in the G. Israelson collection. An Azorean species of similar size and colour as *A. dryochares* is *A.* (*Hummleriella*) *azorica* Bernhauer, described on a single female. According to the description the latter would differ from the former by its abnormally large head, very small eyes, only being a third as long as the temples, furthermore by the antennal structure and by the abdominal tergite VI being provided with a very weak basal impression (Israelson 1985b).

**Figure 23.**
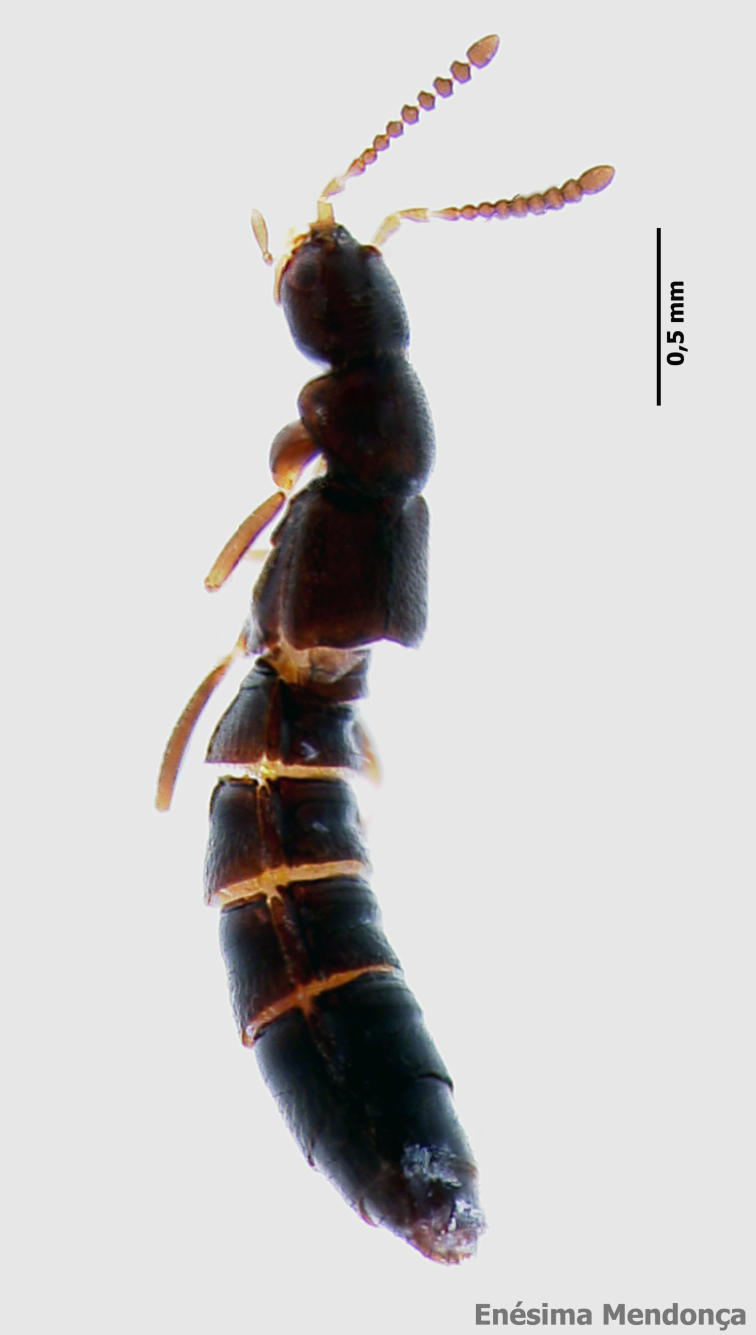
*Atheta dryochares* from Terra Brava (Terceira, Azores) (Credit: Enésima Mendonça).

Figure(s) or Photo(s): Fig. 23

Region for assessment: Global

#### Editor & Reviewers

##### Reviewers

Reviewers: Anja Danielczak

##### Editor

Editor: Axel Hochkirch

#### Reviewers

Reviewers: Anja Danielczak

#### Editor

Editor: Axel Hochkirch

#### Geographic range

Biogeographic realm: Palearctic

Countries: Portugal

Map of records (Google Earth): Suppl. material 36

Basis of EOO and AOO: Observed

Basis (narrative): The extent of occurrence (EOO) is ca 25,000 km^2^ and the maximum area of occupancy (AOO) is 104 km^2^.

Min Elevation/Depth (m): 400

Max Elevation/Depth (m): 1200

Range description: *Atheta dryochares* is an endemic species occurring in Faial, Pico, Graciosa, São Jorge, Terceira, S. Miguel and Santa Maria islands (Azores, Portugal) (Borges et al. 2010; Borges, unpublished data), known in Natural Forest Reserves of Caldeira do Faial (Faial), Caveiro and Mistério da Prainha (Pico), Topo (São Jorge), Biscoito da Ferraria, Pico Galhardo, Serra de Sta. Bárbara and Terra Brava (Terceira); Pico da Vara, Atalhada and Graminhais (S. Miguel); and Pico Alto (Santa Maria).

#### New occurrences

#### Extent of occurrence

EOO (km2): 25,000

Trend: Stable

Justification for trend: The Extent of Occurrence includes large areas of unsuitable habitats. However, the species is widely distributed occurring in many islands.

Causes ceased?: No

Causes understood?: Yes

Causes reversible?: Unknown

Extreme fluctuations?: Unknown

#### Area of occupancy

Trend: Stable

Justification for trend: This species occurs only in native forest patches of seven islands. The AOO with native forest is approximately 40 km^2^. The species occurs in a pristine habitat, the canopy of endemic trees from Azores.

Causes ceased?: No

Causes understood?: Yes

Causes reversible?: Unknown

Extreme fluctuations?: Unknown

AOO (km2): 104

#### Locations

Number of locations: 13

Justification for number of locations: This species occurs in thirteen native forest patches in seven islands.

Trend: Decline (inferred)

Justification for trend: Thirteen locations that were highly impacted by invasive plants in the last ten years, with the spread of *Pittosporum undulatum, Hedychium gardnerianum* (in all islands) and *Clethra arborea* (in S. Miguel) that are changing habitat structure.

Extreme fluctuations?: Unknown

#### Population

Trend: Stable

Justification for trend: The species is particularly abundant in the canopy of endemic trees, and subpopulations are known in seven islands (Faial, Pico, Graciosa, São Jorge, Terceira, S. Miguel and Sta. Maria). The species presents a stable population. The habitat is protected and we assume no impact for the population.

Causes ceased?: No

Causes understood?: Yes

Causes reversible?: Unknown

Extreme fluctuations?: Unknown

#### Subpopulations

Number of subpopulations: 7

Trend: Stable

Justification for trend: The species is particularly abundant in the canopy of endemic trees, and subpopulations are known in seven islands. The habitat is protected and we assume no impact for the subpopulations.

Extreme fluctuations?: Unknown

Severe fragmentation?: No

#### Habitat

System: Terrestrial

Habitat specialist: Yes

Habitat (narrative): This species occurs in native forests dominated by Ilex perado subsp. azorica, *Laurus azorica*, *Erica azorica*, *Juniperus brevifolia* and *Vaccinium cylindraceum*, in the islands of Faial, Pico, São Jorge, Graciosa, Terceira, S. Miguel and Sta Maria (Azores). This species has an altitudinal range between 400 and 1200 m.

Trend in extent, area or quality?: Stable

Justification for trend: The habitat (endemic trees - canopy) is currently protected and the tree canopies are still well preserved.

##### Habitat

Habitat importance: Major Importance

Habitats: 1.4. Forest - Temperate

#### Habitat

Habitat importance: Major Importance

Habitats: 1.4. Forest - Temperate

#### Ecology

Size: 0.17-0.24 cm

Generation length (yr): 1

Dependency of single sp?: No

Ecology and traits (narrative): This is a predator that lives under bark, and is associated with lichens and bryophytes of endemic trees, being active during the night. Based on seasonal data from SLAM traps obtained in several islands between 2012 and 2016 (Borges et al. 2017b), the adults are active all year, being most abundant in spring and summer. This is an univoltine species.

#### Threats

Justification for threats: In the past, the species has probably strongly declined due to changes in habitat size and quality (Triantis et al. 2010, Terzopoulou et al. 2015). Currently the most important ongoing threats to this species are *Cryptomeria japonica* wood & pulp plantations management and the spread of invasive plants namely *Pittosporum undulatum*, *Clethra arborea* and *Hedychium gardnerianum* since are changing the habitat structure, namely decreasing the cover of bryophytes and ferns in the soil and promoting the spread of other plants. Based on Ferreira et al. 2016 the habitat will further decline as a consequence of climate change (increasing number of droughts and habitat shifting & alteration).

##### Threats

Threat type: Ongoing

Threats: 2.2.1. Agriculture & aquaculture - Wood & pulp plantations - Small-holder plantations8.1.2. Invasive and other problematic species, genes & diseases - Invasive non-native/alien species/diseases - Named species

##### Threats

Threat type: Future

Threats: 11.1. Climate change & severe weather - Habitat shifting & alteration11.2. Climate change & severe weather - Droughts

#### Threats

Threat type: Ongoing

Threats: 2.2.1. Agriculture & aquaculture - Wood & pulp plantations - Small-holder plantations8.1.2. Invasive and other problematic species, genes & diseases - Invasive non-native/alien species/diseases - Named species

#### Threats

Threat type: Future

Threats: 11.1. Climate change & severe weather - Habitat shifting & alteration11.2. Climate change & severe weather - Droughts

#### Conservation

Justification for conservation actions: The species is not protected by regional law. Its habitat is located in regionally protected areas (Natural Parks of Faial, Pico, São Jorge, Terceira, S. Miguel and Sta. Maria). Degraded habitats should be restored and a strategy needs to be developed to address the future threat by climate change. It is necessary a monitoring plan for the invertebrate community in the habitat in order to contribute to the conservation of this species. A habitat management plan is needed and anticipated to be developed during the coming years. Formal education and awareness is needed to allow future investments in restore habitats invaded by invasive plants.

##### Conservation actions

Conservation action type: In Place

Conservation actions: 1. Land/water protection1.1. Land/water protection - Site/area protection2.2. Land/water management - Invasive/problematic species control

##### Conservation actions

Conservation action type: Needed

Conservation actions: 2.1. Land/water management - Site/area management2.2. Land/water management - Invasive/problematic species control2.3. Land/water management - Habitat & natural process restoration4. Education & awareness5.4.3. Law & policy - Compliance and enforcement - Sub-national level

#### Conservation actions

Conservation action type: In Place

Conservation actions: 1. Land/water protection1.1. Land/water protection - Site/area protection2.2. Land/water management - Invasive/problematic species control

#### Conservation actions

Conservation action type: Needed

Conservation actions: 2.1. Land/water management - Site/area management2.2. Land/water management - Invasive/problematic species control2.3. Land/water management - Habitat & natural process restoration4. Education & awareness5.4.3. Law & policy - Compliance and enforcement - Sub-national level

#### Other

##### Use and trade

Use type: International

##### Ecosystem services

Ecosystem service type: Less important

Ecosystem services: 12. Biocontrol

##### Research needed

Research needed: 1.2. Research - Population size, distribution & trends1.3. Research - Life history & ecology2.2. Conservation Planning - Area-based Management Plan3.1. Monitoring - Population trends3.4. Monitoring - Habitat trends

Justification for research needed: Further research is needed into its ecology and life history in order to find extant specimens in another islands and obtain information on population size, distribution and trends. It is also necessary a monitoring plan for the invertebrate community in the habitat in order to contribute to perform a species potential recovery plan in some islands where invasive plants are changing habitat structure. Monitoring every ten years using the BALA protocol will inform about habitat quality (see Gaspar et al. 2011).

#### Use and trade

Use type: International

#### Ecosystem services

Ecosystem service type: Less important

Ecosystem services: 12. Biocontrol

#### Research needed

Research needed: 1.2. Research - Population size, distribution & trends1.3. Research - Life history & ecology2.2. Conservation Planning - Area-based Management Plan3.1. Monitoring - Population trends3.4. Monitoring - Habitat trends

Justification for research needed: Further research is needed into its ecology and life history in order to find extant specimens in another islands and obtain information on population size, distribution and trends. It is also necessary a monitoring plan for the invertebrate community in the habitat in order to contribute to perform a species potential recovery plan in some islands where invasive plants are changing habitat structure. Monitoring every ten years using the BALA protocol will inform about habitat quality (see Gaspar et al. 2011).

#### Viability analysis

Justification for probability: 

### Atheta floresensis

#### Species information

Scientific name: Atheta floresensis

Species authority: Pace, 2004

Common names: Rove beetle (English)

Kingdom: Animalia

Phylum: Arthropoda

Class: Insecta

Order: Coleoptera

Family: Staphylinidae

Region for assessment: Global

#### Editor & Reviewers

##### Reviewers

Reviewers: Anja Danielczak

##### Editor

Editor: Axel Hochkirch

#### Reviewers

Reviewers: Anja Danielczak

#### Editor

Editor: Axel Hochkirch

#### Geographic range

Biogeographic realm: Palearctic

Countries: Portugal

Map of records (Google Earth): Suppl. material 37

Basis of EOO and AOO: Observed

Basis (narrative): The extent of occurrence (EOO) is 8 km^2^ and the maximum area of occupancy (AOO) is 8 km^2^.

Min Elevation/Depth (m): 200

Max Elevation/Depth (m): 500

Range description: *Atheta floresensis* is a single-island endemic species from Flores (Azores, Portugal) (Borges et al. 2010), known from a single locality.

#### New occurrences

#### Extent of occurrence

EOO (km2): 8

Trend: Decline (inferred)

Justification for trend: This species occurs in a small fragment of highly modified vegetation in Flores island. The species continues in decline due to native forest destruction, invasive plants and habitat modification

Causes ceased?: No

Causes understood?: Yes

Causes reversible?: Unknown

Extreme fluctuations?: Unknown

#### Area of occupancy

Trend: Decline (inferred)

Justification for trend: This species occurs in a small fragment of highly modified vegetation in Flores island. The species continues in decline due to native forest destruction, invasive plants and habitat modification.

Causes ceased?: No

Causes understood?: Yes

Causes reversible?: Unknown

Extreme fluctuations?: Unknown

AOO (km2): 8

#### Locations

Number of locations: 1

Justification for number of locations: The species occurs in a single human modified forest fragment in the Flores island.

Trend: Decline (inferred)

Justification for trend: Between 1940 and 1950 major land-use changes occurred in the island. In the last 10 years invasive plants namely *Pittosporum undulatum* and *Hedychium gardnerianum* are spreading and changing the structure of the habitat, namely decreasing the cover of bryophytes and ferns with impacts on the species.

Extreme fluctuations?: Unknown

#### Population

Trend: Decline (inferred)

Justification for trend: The species is rare and only known from a single subpopulation in Flores island. The area of occurrence is highly modified duet to human activities and invasive plant species. We assume some impact for the abundance of the population.

Basis for decline: (c) a decline in area of occupancy, extent of occurrence and/or quality of habitat

Causes ceased?: No

Causes understood?: Yes

Causes reversible?: Unknown

Extreme fluctuations?: Unknown

#### Subpopulations

Number of subpopulations: 1

Trend: Decline (inferred)

Justification for trend: The species is rare and only known from a single subpopulation in Flores island. The area of occurrence is highly modified due to human activities and invasive plant species. We assume a decline for the number of subpopulations.

Extreme fluctuations?: Unknown

Severe fragmentation?: No

#### Habitat

System: Terrestrial

Habitat specialist: Yes

Habitat (narrative): This species occurs in a small fragment of human modified forest in Flores island (Azores), dominated by *Pittosporum undulatum*. This species has an altitudinal range between 200 and 500 m.

Trend in extent, area or quality?: Decline (observed)

Justification for trend: Destruction of habitat for agriculture, and there is the spread of an invasive plant (*Pittosporum undulatum*).

##### Habitat

Habitat importance: Major Importance

Habitats: 1.4. Forest - Temperate

#### Habitat

Habitat importance: Major Importance

Habitats: 1.4. Forest - Temperate

#### Ecology

Size: 0.26-0.33 cm

Generation length (yr): 1

Dependency of single sp?: No

Ecology and traits (narrative): Adults and larvae are nocturnal predators and were found in wet debris and moss near the margin of a small river. This is an univoltine species.

#### Threats

Justification for threats: In the past, the species has probably strongly declined due to changes in habitat size and quality (Triantis et al. 2010, Terzopoulou et al. 2015). Currently the main threat is the major changes in habitats for urban use, industrial plantations of *Cryptomeria japonica* and pastures, but also the spread of invasive plants namely *Hedychium gardnerianum* and *Pittosporum undulatum* since are changing the habitat structure, namely decreasing the cover of bryophytes and ferns in the soil and promoting the spread of other plants. Based on Ferreira et al. 2016 the habitat will further decline as a consequence of climate change (increasing number of droughts and habitat shifting & alteration).

##### Threats

Threat type: Ongoing

Threats: 2.2.1. Agriculture & aquaculture - Wood & pulp plantations - Small-holder plantations8.1.2. Invasive and other problematic species, genes & diseases - Invasive non-native/alien species/diseases - Named species

##### Threats

Threat type: Future

Threats: 11.1. Climate change & severe weather - Habitat shifting & alteration11.2. Climate change & severe weather - Droughts

#### Threats

Threat type: Ongoing

Threats: 2.2.1. Agriculture & aquaculture - Wood & pulp plantations - Small-holder plantations8.1.2. Invasive and other problematic species, genes & diseases - Invasive non-native/alien species/diseases - Named species

#### Threats

Threat type: Future

Threats: 11.1. Climate change & severe weather - Habitat shifting & alteration11.2. Climate change & severe weather - Droughts

#### Conservation

Justification for conservation actions: The species is not protected by regional law. Its habitat is in a degraded area that should be restored and a strategy needs to be developed to address the future threat by climate change. It is necessary a monitoring plan for the invertebrate community in the habitat in order to contribute to the conservation of this species. A habitat management plan is needed and anticipated to be developed during the coming years.

##### Conservation actions

Conservation action type: Needed

Conservation actions: 2.1. Land/water management - Site/area management2.2. Land/water management - Invasive/problematic species control2.3. Land/water management - Habitat & natural process restoration4. Education & awareness5.4.3. Law & policy - Compliance and enforcement - Sub-national level

#### Conservation actions

Conservation action type: Needed

Conservation actions: 2.1. Land/water management - Site/area management2.2. Land/water management - Invasive/problematic species control2.3. Land/water management - Habitat & natural process restoration4. Education & awareness5.4.3. Law & policy - Compliance and enforcement - Sub-national level

#### Other

##### Use and trade

Use type: International

##### Ecosystem services

Ecosystem service type: Less important

Ecosystem services: 12. Biocontrol

##### Research needed

Research needed: 1.2. Research - Population size, distribution & trends1.3. Research - Life history & ecology2.2. Conservation Planning - Area-based Management Plan3.1. Monitoring - Population trends3.4. Monitoring - Habitat trends

Justification for research needed: Further research is needed into its ecology and life history in order to find extant specimens in other sites in Flores, particularly in native forest and obtain information on population size, distribution and trends. It is also necessary a area-based management plan and a monitoring plan for the invertebrate community in the habitat in order to contribute to perform a species potential recovery plan. Monitoring every ten years using the BALA protocol will inform about habitat quality (see e.g. Gaspar et al. 2011).

#### Use and trade

Use type: International

#### Ecosystem services

Ecosystem service type: Less important

Ecosystem services: 12. Biocontrol

#### Research needed

Research needed: 1.2. Research - Population size, distribution & trends1.3. Research - Life history & ecology2.2. Conservation Planning - Area-based Management Plan3.1. Monitoring - Population trends3.4. Monitoring - Habitat trends

Justification for research needed: Further research is needed into its ecology and life history in order to find extant specimens in other sites in Flores, particularly in native forest and obtain information on population size, distribution and trends. It is also necessary a area-based management plan and a monitoring plan for the invertebrate community in the habitat in order to contribute to perform a species potential recovery plan. Monitoring every ten years using the BALA protocol will inform about habitat quality (see e.g. Gaspar et al. 2011).

#### Viability analysis

Justification for probability: 

### Euconnus azoricus

#### Species information

Scientific name: Euconnus azoricus

Species authority: Franz, 1969

Common names: Ant-like stone beetle (English)

Kingdom: Animalia

Phylum: Arthropoda

Class: Insecta

Order: Coleoptera

Family: Staphylinidae

Region for assessment: Global

#### Editor & Reviewers

##### Reviewers

Reviewers: Anja Danielczak

##### Editor

Editor: Axel Hochkirch

#### Reviewers

Reviewers: Anja Danielczak

#### Editor

Editor: Axel Hochkirch

#### Geographic range

Biogeographic realm: Palearctic

Countries: Portugal

Map of records (Google Earth): Suppl. material 38

Basis of EOO and AOO: Observed

Basis (narrative): The extent of occurrence (EOO) is ca 7,000 km^2^ and the maximum area of occupancy (AOO) is 44 km^2^.

Min Elevation/Depth (m): 10

Max Elevation/Depth (m): 800

Range description: *Euconnus azoricus* is an endemic species from Terceira, Pico and São Miguel (Azores, Portugal) (Borges et al. 2010 and unpublished data), known from Monte Brasil (Terceira), Furnas (São Miguel) and Pico Redondo (Pico).

#### New occurrences

#### Extent of occurrence

EOO (km2): 7,000

Trend: Decline (inferred)

Justification for trend: This species occurs in a fragment of native and exotic forest of Terceira island (Monte Brasil), an area with exotic *Cryptomeria japonica* plantations in Furnas (S. Miguel) and in a small fragment with native forest and *Pinus*. sp. (Pico Redondo; Pico). Possibly the EOO value is slightly overestimated. The species continues in decline due to native forest destruction, invasive plants and habitat modification.

Causes ceased?: No

Causes understood?: Yes

Causes reversible?: Unknown

Extreme fluctuations?: Unknown

#### Area of occupancy

Trend: Decline (inferred)

Justification for trend: This species occurs in a fragment of native and exotic forest of Terceira island (Monte Brasil), an area with exotic *Cryptomeria japonica* plantations in Furnas (S. Miguel) and in a small fragment with native forest and *Pinus*. sp. (Pico Redondo; Pico). Possibly the AOO value is slightly overestimated. The species continues in decline due to native forest destruction, invasive plants and habitat modification.

Causes ceased?: No

Causes understood?: Yes

Causes reversible?: Unknown

Extreme fluctuations?: Unknown

AOO (km2): 44

#### Locations

Number of locations: 3

Justification for number of locations: This species occurs in three highly modified forest patches in Terceira island (Monte Brasil), Furnas (São Miguel) and Pico Island (Pico Redondo).

Trend: Decline (inferred)

Justification for trend: Only three locations left that were highly impacted by land use changes and invasive plants in the last ten years.

Extreme fluctuations?: Unknown

#### Population

Trend: Decline (inferred)

Justification for trend: The species is only known from three isolated subpopulations, one in Terceira island (Monte Brasil), one in Furnas (São Miguel) and a recent finding in Pico Redondo (Pico Island). A continuing decline in the number of mature individuals is inferred from historical and recent habitat modification. This species can be on the edge of extinction at Terceira island due to major recent changes in its type locality.

Causes ceased?: No

Causes understood?: Yes

Causes reversible?: Unknown

Extreme fluctuations?: Unknown

#### Subpopulations

Number of subpopulations: 3

Trend: Decline (inferred)

Justification for trend: The species is only known from three isolated subpopulations, one in Terceira island (Monte Brasil), one in Furnas (São Miguel) and a recent finding in Pico Redondo (Pico Island). A continuing decline in the number of subpopulations is inferred from habitat modification.

Extreme fluctuations?: Unknown

Severe fragmentation?: Yes

Justification for fragmentation: Major land-use changes at low and middle elevations promoted the creation of small patches of native and exotic forest that are isolated in a sea of pastures and *Cryptomeria japonica* plantations and that were highly impacted by invasive plants in the last ten years.

#### Habitat

System: Terrestrial

Habitat specialist: Yes

Habitat (narrative): This species occurs in a forest patch with native and exotic vegetation in Terceira island (Monte Brasil), in the highly modified area of Furnas (São Miguel) dominated by *Cryptomeria japonica* plantations and in a fragment of native forest dominated by *Juniperus brevifolia* mixed with planted *Pinus* sp. in Pico Redondo at Pico island. Altitudinal range is between 10 and 800 m.

Trend in extent, area or quality?: Decline (observed)

Justification for trend: In Terceira, destruction of habitat for creation of urban areas, agriculture, and there is the spread of an invasive plant (*Pittosporum undulatum*). In Pico, plantation of *Pinus* sp. mixed with native vegetation. In São Miguel the *Cryptomeria japonica* plantations are being heavily invaded by *Hedychium gardnerianum*.

##### Habitat

Habitat importance: Major Importance

Habitats: 1.4. Forest - Temperate14.3. Artificial/Terrestrial - Plantations

#### Habitat

Habitat importance: Major Importance

Habitats: 1.4. Forest - Temperate14.3. Artificial/Terrestrial - Plantations

#### Ecology

Size: 0.19 cm

Generation length (yr): 1

Dependency of single sp?: No

Ecology and traits (narrative): This is a nocturnal predator that lives under bark of native trees and in the soil. This is an univoltine species.

#### Threats

Justification for threats: In the past, the species has probably strongly declined due to changes in habitat size and quality (Triantis et al. 2010). In the last 50 years the invasive plant *Pittosporum undulatum* spread in the area of Monte Brasil with the major decrease of native trees and shrubs and current dominance of *Pittosporum undulatum*. In Pico island the plantation of *Pinus* sp. mixed within native vegetation may become a problem for the adequate persistence of native plants. In Furnas, spread of *Hedychium gardnerianum* is destroying the habitat, since is changing the habitat structure, namely decreasing the cover of bryophytes and ferns in the soil and promoting the spread of other plants. Based on Ferreira et al. 2016 the habitat will further decline as a consequence of climate change (increasing number of droughts and habitat shifting & alteration).

##### Threats

Threat type: Ongoing

Threats: 2.2.1. Agriculture & aquaculture - Wood & pulp plantations - Small-holder plantations8.1.2. Invasive and other problematic species, genes & diseases - Invasive non-native/alien species/diseases - Named species

##### Threats

Threat type: Future

Threats: 11.1. Climate change & severe weather - Habitat shifting & alteration11.2. Climate change & severe weather - Droughts

#### Threats

Threat type: Ongoing

Threats: 2.2.1. Agriculture & aquaculture - Wood & pulp plantations - Small-holder plantations8.1.2. Invasive and other problematic species, genes & diseases - Invasive non-native/alien species/diseases - Named species

#### Threats

Threat type: Future

Threats: 11.1. Climate change & severe weather - Habitat shifting & alteration11.2. Climate change & severe weather - Droughts

#### Conservation

Justification for conservation actions: The species is not protected by regional law. Its habitat is in regionally protected areas (Natural Parks of Pico, Terceira, S. Miguel). Degraded habitats should be restored and a strategy needs to be developed to address the current threat by invasive plants (*Pittosporum undulatum* and *Hedychium gardnerianum*). It is necessary a monitoring plan for the invertebrate community in the habitat in order to contribute to the conservation of this species. A habitat management plan is needed and anticipated to be developed during the coming years.

##### Conservation actions

Conservation action type: In Place

Conservation actions: 1. Land/water protection1.1. Land/water protection - Site/area protection2.2. Land/water management - Invasive/problematic species control

##### Conservation actions

Conservation action type: Needed

Conservation actions: 2.1. Land/water management - Site/area management2.2. Land/water management - Invasive/problematic species control2.3. Land/water management - Habitat & natural process restoration4. Education & awareness5.4.3. Law & policy - Compliance and enforcement - Sub-national level

#### Conservation actions

Conservation action type: In Place

Conservation actions: 1. Land/water protection1.1. Land/water protection - Site/area protection2.2. Land/water management - Invasive/problematic species control

#### Conservation actions

Conservation action type: Needed

Conservation actions: 2.1. Land/water management - Site/area management2.2. Land/water management - Invasive/problematic species control2.3. Land/water management - Habitat & natural process restoration4. Education & awareness5.4.3. Law & policy - Compliance and enforcement - Sub-national level

#### Other

##### Use and trade

Use type: International

##### Ecosystem services

Ecosystem service type: Less important

Ecosystem services: 12. Biocontrol

##### Research needed

Research needed: 1.2. Research - Population size, distribution & trends1.3. Research - Life history & ecology2.2. Conservation Planning - Area-based Management Plan3.1. Monitoring - Population trends3.4. Monitoring - Habitat trends

Justification for research needed: Further research is needed into its ecology and life history in order to find extant specimens in new localities in known islands and also in another islands and obtain information on population size, distribution and trends. It is also necessary a area-based management plan and a monitoring plan for the invertebrate community in the habitat in order to contribute to perform a species potential recovery plan. Monitoring every ten years using the BALA protocol will inform about habitat quality (see e.g. Gaspar et al. 2011).

#### Use and trade

Use type: International

#### Ecosystem services

Ecosystem service type: Less important

Ecosystem services: 12. Biocontrol

#### Research needed

Research needed: 1.2. Research - Population size, distribution & trends1.3. Research - Life history & ecology2.2. Conservation Planning - Area-based Management Plan3.1. Monitoring - Population trends3.4. Monitoring - Habitat trends

Justification for research needed: Further research is needed into its ecology and life history in order to find extant specimens in new localities in known islands and also in another islands and obtain information on population size, distribution and trends. It is also necessary a area-based management plan and a monitoring plan for the invertebrate community in the habitat in order to contribute to perform a species potential recovery plan. Monitoring every ten years using the BALA protocol will inform about habitat quality (see e.g. Gaspar et al. 2011).

#### Viability analysis

Justification for probability: 

### Geostiba melanocephala

#### Species information

Scientific name: Geostiba melanocephala

Species authority: (Crotch, 1867)

Synonyms: *Sipalia melanocephala* Crotch; *Xenomma capillaricornis* Grav.

Common names: Rove beetle (English)

Kingdom: Animalia

Phylum: Arthropoda

Class: Insecta

Order: Coleoptera

Family: Staphylinidae

Region for assessment: Global

#### Editor & Reviewers

##### Reviewers

Reviewers: Anja Danielczak

##### Editor

Editor: Axel Hochkirch

#### Reviewers

Reviewers: Anja Danielczak

#### Editor

Editor: Axel Hochkirch

#### Geographic range

Biogeographic realm: Palearctic

Countries: Portugal

Map of records (Google Earth): Suppl. material 39

Basis of EOO and AOO: Observed

Basis (narrative): Based on a unique cell of the historical locality.

Range description: *Geostiba melanocephala* is a single-island endemic species from São Miguel (Azores, Portugal) (Borges et al. 2010). This species is very rare and possibly it is near extinction.

#### New occurrences

#### Extent of occurrence

EOO (km2): 0-4

Trend: Stable

Justification for trend: Based on the area of a unique cell of the historical locality. The species is considered extinct in the historical locality possibly due to habitat destruction. Not sampled recently despite some intensive field work (Borges et al. 2005, Borges et al. 2016).

Causes ceased?: No

Causes understood?: Yes

Causes reversible?: Unknown

Extreme fluctuations?: Unknown

#### Area of occupancy

Trend: Stable

Justification for trend: Based on the area of a unique cell of the historical locality. The species is considered extinct in the historical locality possibly due to habitat destruction. Not sampled recently despite some intensive field work (Borges et al. 2005, Borges et al. 2016).

Causes ceased?: No

Causes understood?: Yes

Causes reversible?: Unknown

Extreme fluctuations?: Unknown

AOO (km2): 0-4

#### Locations

Number of locations: 0-1

Justification for number of locations: The original historical location

Trend: Unknown

Justification for trend: Possibly extinct.

Extreme fluctuations?: Unknown

#### Population

Trend: Stable

Justification for trend: The species is only known from a single subpopulation. Possibly extinct.

Causes ceased?: No

Causes understood?: Yes

Causes reversible?: Unknown

Extreme fluctuations?: Unknown

#### Subpopulations

Number of subpopulations: 0-1

Trend: Stable

Justification for trend: The species is only known from a single subpopulation. Possibly extinct.

Extreme fluctuations?: Unknown

Severe fragmentation?: No

#### Habitat

System: Terrestrial

Habitat specialist: Unknown

Habitat (narrative): The species occurred in the native forest of São Miguel Island (Azores), but it is considered possibly extinct. This is a nocturnal predator. The current altitudinal range is unknown.

Trend in extent, area or quality?: Decline (observed)

Justification for trend: Since the historical record, the native habitat in the island of São Miguel was greatly reduced to accomodate pastures and *Cryptomeria japonica* plantations (Triantis et al. 2010).

##### Habitat

Habitat importance: Major Importance

Habitats: 1.4. Forest - Temperate

#### Habitat

Habitat importance: Major Importance

Habitats: 1.4. Forest - Temperate

#### Ecology

Generation length (yr): 1

Dependency of single sp?: No

Ecology and traits (narrative): This is a nocturnal predator. The current altitudinal range is unknown. This is an univoltine species.

#### Threats

Justification for threats: In the past, the species has probably strongly declined due to changes in habitat size. In the last 50 years additional major changes occurred with pasture intensification and the spread of the invasive plant *Hedychium gardnerianum* that is changing the habitat structure, namely decreasing the cover of bryophytes and ferns in the soil and promoting the spread of other plants. Based on Ferreira et al. 2016 the habitat will further decline as a consequence of climate change (increasing number of droughts and habitat shifting & alteration).

##### Threats

Threat type: Ongoing

Threats: 2.2.1. Agriculture & aquaculture - Wood & pulp plantations - Small-holder plantations8.1.2. Invasive and other problematic species, genes & diseases - Invasive non-native/alien species/diseases - Named species12. Other options - Other threat

##### Threats

Threat type: Future

Threats: 11.1. Climate change & severe weather - Habitat shifting & alteration11.2. Climate change & severe weather - Droughts

#### Threats

Threat type: Ongoing

Threats: 2.2.1. Agriculture & aquaculture - Wood & pulp plantations - Small-holder plantations8.1.2. Invasive and other problematic species, genes & diseases - Invasive non-native/alien species/diseases - Named species12. Other options - Other threat

#### Threats

Threat type: Future

Threats: 11.1. Climate change & severe weather - Habitat shifting & alteration11.2. Climate change & severe weather - Droughts

#### Conservation

Justification for conservation actions: The species is not protected by regional law. Its habitat is possibly in a regionally protected area (Natural Park of São Miguel Island). Degraded habitats should be restored and of critical importance is the continued expansion of invasive plant species. A strategy needs to be developed to address the future threat by climate change. It is necessary a monitoring plan for the invertebrate community in the habitat in order to contribute to the conservation of this species. A habitat management plan is needed and anticipated to be developed during the coming years. Formal education and awareness is needed to allow future investments in restored habitats invaded by invasive plants

##### Conservation actions

Conservation action type: In Place

Conservation actions: 1. Land/water protection1.1. Land/water protection - Site/area protection

##### Conservation actions

Conservation action type: Needed

Conservation actions: 2.1. Land/water management - Site/area management2.2. Land/water management - Invasive/problematic species control2.3. Land/water management - Habitat & natural process restoration4. Education & awareness5.4.3. Law & policy - Compliance and enforcement - Sub-national level

#### Conservation actions

Conservation action type: In Place

Conservation actions: 1. Land/water protection1.1. Land/water protection - Site/area protection

#### Conservation actions

Conservation action type: Needed

Conservation actions: 2.1. Land/water management - Site/area management2.2. Land/water management - Invasive/problematic species control2.3. Land/water management - Habitat & natural process restoration4. Education & awareness5.4.3. Law & policy - Compliance and enforcement - Sub-national level

#### Other

##### Use and trade

Use type: International

##### Ecosystem services

Ecosystem service type: Less important

Ecosystem services: 12. Biocontrol

##### Research needed

Research needed: 1.2. Research - Population size, distribution & trends1.3. Research - Life history & ecology2.2. Conservation Planning - Area-based Management Plan3.1. Monitoring - Population trends3.4. Monitoring - Habitat trends

Justification for research needed: Further research is needed into its ecology and life history in order to find extant specimens in the native forests of S. Miguel island and obtain information on population size, distribution and trends. It is also necessary a monitoring plan for the invertebrate community in the habitat in order to contribute to perform a species potential recovery plan. Monitoring every ten years using the BALA protocol will inform about habitat quality (see e.g. Gaspar et al. 2011).

#### Use and trade

Use type: International

#### Ecosystem services

Ecosystem service type: Less important

Ecosystem services: 12. Biocontrol

#### Research needed

Research needed: 1.2. Research - Population size, distribution & trends1.3. Research - Life history & ecology2.2. Conservation Planning - Area-based Management Plan3.1. Monitoring - Population trends3.4. Monitoring - Habitat trends

Justification for research needed: Further research is needed into its ecology and life history in order to find extant specimens in the native forests of S. Miguel island and obtain information on population size, distribution and trends. It is also necessary a monitoring plan for the invertebrate community in the habitat in order to contribute to perform a species potential recovery plan. Monitoring every ten years using the BALA protocol will inform about habitat quality (see e.g. Gaspar et al. 2011).

#### Viability analysis

Justification for probability: 

### Medon varamontis

#### Species information

Scientific name: Medon varamontis

Species authority: Assing, 2013

Common names: Rove beetle (English)

Kingdom: Animalia

Phylum: Arthropoda

Class: Insecta

Order: Coleoptera

Family: Staphylinidae

Taxonomic notes: This species was described from five specimens collected in 2013 in Pico da Vara in S. Miguel island. *M. varamontis* belongs to the *M. ferrugineus* group, based on the male sexual characters (Assing 2013).

Region for assessment: Global

#### Editor & Reviewers

##### Reviewers

Reviewers: Anja Danielczak

##### Editor

Editor: Axel Hochkirch

#### Reviewers

Reviewers: Anja Danielczak

#### Editor

Editor: Axel Hochkirch

#### Geographic range

Biogeographic realm: Palearctic

Countries: Portugal

Map of records (Google Earth): Suppl. material 40

Basis of EOO and AOO: Observed

Basis (narrative): The extent of occurrence (EOO) is 4 km^2^ and the maximum area of occupancy (AOO) is 4 km^2^.

Min Elevation/Depth (m): 1000

Max Elevation/Depth (m): 1200

Range description: *Medon varamontis* is a single-island endemic species from S. Miguel (Azores, Portugal) (Borges et al. 2010), known from Natural Forest Reserve of Pico da Vara.

#### New occurrences

#### Extent of occurrence

EOO (km2): 4

Trend: Decline (inferred)

Justification for trend: This species occurs in a fragment of native forest of S. Miguel island (Tronqueira in NFF of Pico da Vara). Possibly the EOO value is slightly overestimated. The EOO is in decline due to native forest destruction, habitat fragmentation and the spread of invasive plants that are changing the habitat of the species.

Causes ceased?: No

Causes understood?: Yes

Causes reversible?: Unknown

Extreme fluctuations?: Unknown

#### Area of occupancy

Trend: Decline (inferred)

Justification for trend: This species occurs in a fragment of native forest of S. Miguel island (Tronqueira in NFF of Pico da Vara). Possibly the AOO value is slightly overestimated. The AOO is in decline due to native forest destruction, habitat fragmentation and the spread of invasive plants that are changing the habitat of the species.

Causes ceased?: No

Causes understood?: Yes

Causes reversible?: Unknown

Extreme fluctuations?: Unknown

AOO (km2): 4

#### Locations

Number of locations: 1

Justification for number of locations: This species occurs in one single native forest patch in S. Miguel island (Tronqueira).

Trend: Decline (inferred)

Justification for trend: Only one site left that is now well protected and had a relatively high value of biotic integrity (Gaspar et al. 2011). However, in the last ten years invasive plant species spreading (e.g. *Hedychium gardnerianum*; *Clethra arborea*) are changing the structure of the forest and the cover of bryophytes and ferns in the soil decreasing the quality of the habitat with impacts on the species.

Extreme fluctuations?: Unknown

#### Population

Trend: Decline (inferred)

Justification for trend: The species is particularly abundant and only known from a single subpopulation in a high elevation area in S. Miguel island. There is an inferred declining in the population due to invasive plant species spreading (e.g. *Hedychium gardnerianum*; *Clethra arborea*), that are changing the structure of the forest and the cover of bryophytes and ferns in the soil decreasing the quality of the habitat with impacts on the species.

Causes ceased?: No

Causes understood?: Yes

Causes reversible?: Unknown

Extreme fluctuations?: Unknown

#### Subpopulations

Number of subpopulations: 1

Trend: Decline (inferred)

Justification for trend: The species is particularly abundant and only known from a single subpopulation in a high elevation area in S. Miguel island. There is an inferred declining , since this subpopulation may be threatened due to invasive plant species spreading (e.g. *Hedychium gardnerianum*; *Clethra arborea*), that are changing the structure of the forest and the cover of bryophytes and ferns in the soil decreasing the quality of the habitat with impacts on the species.

Extreme fluctuations?: Unknown

Severe fragmentation?: No

#### Habitat

System: Terrestrial

Habitat specialist: Yes

Habitat (narrative): This species occurs in one single native natural grassland and bog patch (dominated by *Sphagnum* spp.) located at high altitude with also the scattered presence of the Azorean cedar (*Juniperus brevifolia*), in S. Miguel island (Pico da Vara). This species has an altitudinal range between 1000 and 1200 m.

Trend in extent, area or quality?: Decline (observed)

Justification for trend: Destruction of habitat for creation of plantations of *Cryptomeria japonica*a amd the spread of invasive plants (e.g. *Hedychium gardnerianum*; *Clethra arborea*).

##### Habitat

Habitat importance: Major Importance

Habitats: 4. Grassland5.4. Wetlands (inland) - Bogs, Marshes, Swamps, Fens, Peatlands

#### Habitat

Habitat importance: Major Importance

Habitats: 4. Grassland5.4. Wetlands (inland) - Bogs, Marshes, Swamps, Fens, Peatlands

#### Ecology

Size: 0.45-0.48

Generation length (yr): 1

Dependency of single sp?: No

Ecology and traits (narrative): This is a nocturnal predator that lives in the soil associated with grass roots and litter. This is an univoltine species.

#### Threats

Justification for threats: In the past, the species has probably strongly declined due to changes in habitat size and quality (Triantis et al. 2010), mostly the creation of plantations of *Cryptomeria japonica* and pastures. The management of *Cryptomeria japonica* is still a problem. One of the most important ongoing threats to this species is the spread of invasive plants, namely *Hedychium gardnerianum and Clethra arborea* that are changing the habitat structure, namely decreasing the cover of bryophytes and ferns in the soil and promoting the spread of other plants. Based on Ferreira et al. 2016 the habitat will further decline as a consequence of climate change (increasing number of droughts and habitat shifting & alteration).

##### Threats

Threat type: Ongoing

Threats: 2.2.1. Agriculture & aquaculture - Wood & pulp plantations - Small-holder plantations8.1.2. Invasive and other problematic species, genes & diseases - Invasive non-native/alien species/diseases - Named species

##### Threats

Threat type: Future

Threats: 11.1. Climate change & severe weather - Habitat shifting & alteration11.2. Climate change & severe weather - Droughts

#### Threats

Threat type: Ongoing

Threats: 2.2.1. Agriculture & aquaculture - Wood & pulp plantations - Small-holder plantations8.1.2. Invasive and other problematic species, genes & diseases - Invasive non-native/alien species/diseases - Named species

#### Threats

Threat type: Future

Threats: 11.1. Climate change & severe weather - Habitat shifting & alteration11.2. Climate change & severe weather - Droughts

#### Conservation

Justification for conservation actions: The species is not protected by regional law. Its habitat is in a regionally protected area (Natural Park of S. Miguel). Further spread of invasive plants needs to be stopped in order to avoid any future declines of the species. Degraded habitats should be restored and a strategy needs to be developed to address the future threat by climate change. It is necessary a monitoring plan for the invertebrate community in the habitat in order to contribute to the conservation of this species. A habitat management plan is needed and anticipated to be developed during the coming years. Formal education and awareness is needed to allow future investments in restored habitats invaded by invasive plants.

##### Conservation actions

Conservation action type: In Place

Conservation actions: 1. Land/water protection1.1. Land/water protection - Site/area protection2.2. Land/water management - Invasive/problematic species control

##### Conservation actions

Conservation action type: Needed

Conservation actions: 2.1. Land/water management - Site/area management2.2. Land/water management - Invasive/problematic species control2.3. Land/water management - Habitat & natural process restoration4. Education & awareness5.4.3. Law & policy - Compliance and enforcement - Sub-national level

#### Conservation actions

Conservation action type: In Place

Conservation actions: 1. Land/water protection1.1. Land/water protection - Site/area protection2.2. Land/water management - Invasive/problematic species control

#### Conservation actions

Conservation action type: Needed

Conservation actions: 2.1. Land/water management - Site/area management2.2. Land/water management - Invasive/problematic species control2.3. Land/water management - Habitat & natural process restoration4. Education & awareness5.4.3. Law & policy - Compliance and enforcement - Sub-national level

#### Other

##### Use and trade

Use type: International

##### Ecosystem services

Ecosystem service type: Less important

Ecosystem services: 12. Biocontrol

##### Research needed

Research needed: 1.2. Research - Population size, distribution & trends1.3. Research - Life history & ecology2.2. Conservation Planning - Area-based Management Plan3.1. Monitoring - Population trends3.4. Monitoring - Habitat trends

Justification for research needed: Further research is needed into its ecology and life history in order to find more extant specimens in additional areas around Pico da Vara (S. Miguel) and obtain information on population size, distribution and trends. It is also necessary a monitoring plan for the invertebrate community in the habitat in order to contribute to perform a species potential recovery plan. Monitoring every ten years using the BALA protocol will inform about habitat quality (see e.g. Gaspar et al. 2011).

#### Use and trade

Use type: International

#### Ecosystem services

Ecosystem service type: Less important

Ecosystem services: 12. Biocontrol

#### Research needed

Research needed: 1.2. Research - Population size, distribution & trends1.3. Research - Life history & ecology2.2. Conservation Planning - Area-based Management Plan3.1. Monitoring - Population trends3.4. Monitoring - Habitat trends

Justification for research needed: Further research is needed into its ecology and life history in order to find more extant specimens in additional areas around Pico da Vara (S. Miguel) and obtain information on population size, distribution and trends. It is also necessary a monitoring plan for the invertebrate community in the habitat in order to contribute to perform a species potential recovery plan. Monitoring every ten years using the BALA protocol will inform about habitat quality (see e.g. Gaspar et al. 2011).

#### Viability analysis

Justification for probability: 

### Phloeostiba azorica

#### Species information

Scientific name: Phloeostiba azorica

Species authority: (Fauvel, 1900)

Synonyms: *Homalium clavicorne* Wollaston; *Phloeonomus azoricus* Fauvel

Common names: Rove beetle (English)

Kingdom: Animalia

Phylum: Arthropoda

Class: Insecta

Order: Coleoptera

Family: Staphylinidae

**Figure 24.**
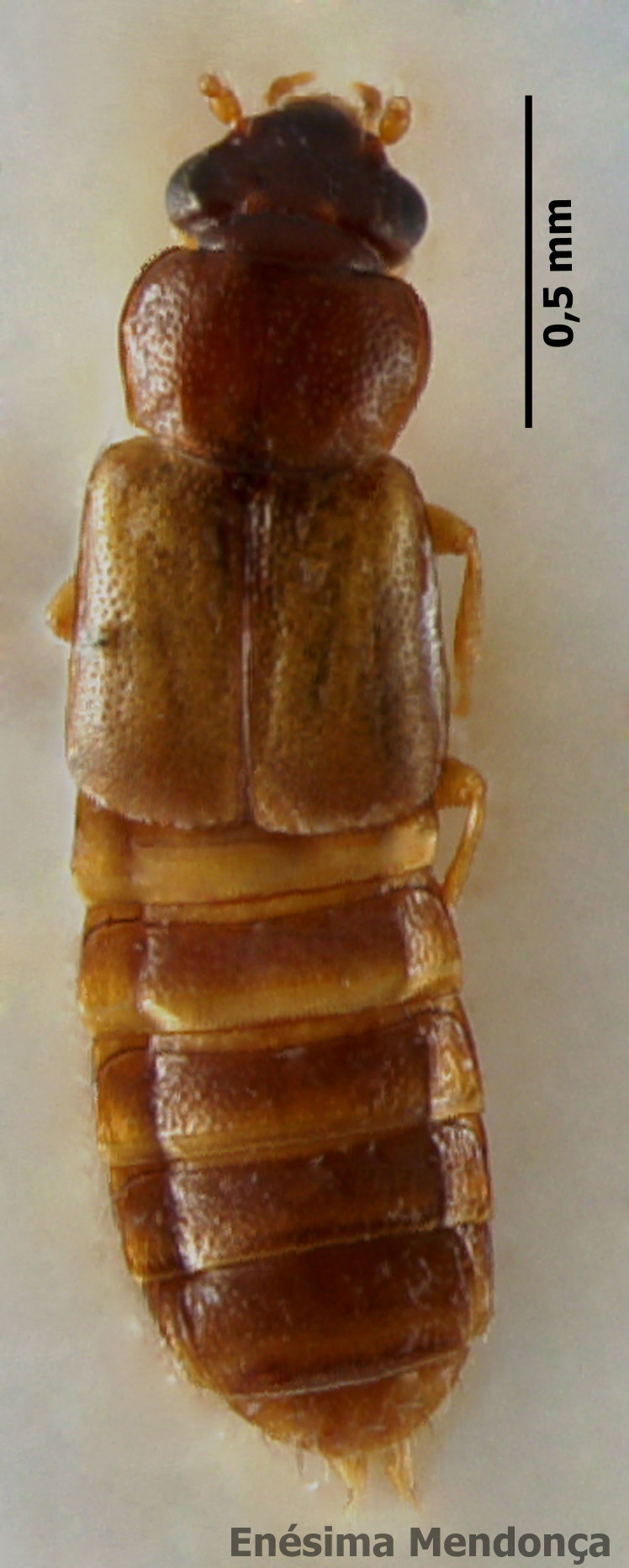
*Phloeostiba azorica* from Terra Chã (Terceira, Azores) (Credit: Enésima Mendonça).

Figure(s) or Photo(s): Fig. 24

Region for assessment: Global

#### Editor & Reviewers

##### Reviewers

Reviewers: Anja Danielczak

##### Editor

Editor: Axel Hochkirch

#### Reviewers

Reviewers: Anja Danielczak

#### Editor

Editor: Axel Hochkirch

#### Geographic range

Biogeographic realm: Palearctic

Countries: Portugal

Map of records (Google Earth): Suppl. material 41

Basis of EOO and AOO: Observed

Basis (narrative): The extent of occurrence (EOO) is ca 8,800 km^2^ and the maximum area of occupancy (AOO) is 36 km^2^.

Min Elevation/Depth (m): 300

Max Elevation/Depth (m): 1200

Range description: *Phloeostiba azorica* is an endemic species occurring in Flores, Pico, São Jorge, Graciosa, Terceira and S. Miguel islands (Azores, Portugal) (Borges et al. 2010), known in Natural Forest Reserves of Mistério da Prainha and Lagoa Caiado (Pico), Pico Pinheiro (S. Jorge); Biscoito da Ferraria, Caldeira de Guilherme Moniz and Serra de Sta. Bárbara (Terceira); and Atalhada and Graminhais (S. Miguel).

#### New occurrences

#### Extent of occurrence

EOO (km2): 8,800

Trend: Decline (inferred)

Justification for trend: The Extent of Occurrence includes large areas of unsuitable habitats. The EOO continues in decline due to native forest destruction, habitat fragmentation and the spread of invasive plants.

Causes ceased?: No

Causes understood?: Yes

Causes reversible?: Unknown

Extreme fluctuations?: Unknown

#### Area of occupancy

Trend: Decline (inferred)

Justification for trend: This species occurs in native forest patches of Pico, Terceira and S. Miguel islands. The AOO with native forest is approximately 30 km^2^. The AOO continues in decline due to native forest destructin, habitat fragmentation and the spread of invasive plants.

Causes ceased?: No

Causes understood?: Yes

Causes reversible?: Unknown

Extreme fluctuations?: Unknown

AOO (km2): 36

#### Locations

Number of locations: 8

Justification for number of locations: This species occurs in eight native forest patches in the islands of Pico (Mistério da Prainha and Lagoa do Caiado), São Jorge (Pico Pinheiro), Terceira (Biscoito da Ferraria, Caldeira de Guilherme Moniz and Serra de Sta. Bárbara) and S. Miguel (Atalhada and Graminhais) islands.

Trend: Decline (inferred)

Justification for trend: Eight locations that were highly impacted by invasive plants in the last ten years, namely Pico Pinheiro (S. Jorge), Biscoito da Ferraria and Caldeira de Guilherme Moniz (Terceira) and Atalhada (S. Miguel).

Extreme fluctuations?: Unknown

#### Population

Trend: Decline (inferred)

Justification for trend: The species is particularly abundant and subpopulations are known in Flores, Terceira, Pico, São Jorge, Gracioasa and S. Miguel islands. There is an inferred declining in the population due to invasive plant species spreading (e.g. *Hedychium gardnerianum*, *Pittosporum undulatum*), that are changing the structure of the forest decreasing the quality of the habitat with impacts on the species.

Causes ceased?: No

Causes understood?: Yes

Causes reversible?: Unknown

Extreme fluctuations?: Unknown

#### Subpopulations

Number of subpopulations: 6

Trend: Decline (inferred)

Justification for trend: The species is particularly abundant and subpopulations are known in Flores, Terceira, Pico, São Jorge, Gracioasa and S. Miguel. There is an inferred declining in the number of subpopulations due to invasive plant species spreading (e.g. *Hedychium gardnerianum*, *Pittosporum undulatum*), that are changing the structure of the forest decreasing the quality of the habitat with impacts on the species, At least Guilherme Moniz (Terceira) and Atalhada (S. Miguel) have very low values of biotic integrity (Gaspar et al. 2011).

Extreme fluctuations?: Unknown

Severe fragmentation?: Yes

Justification for fragmentation: Major land-use changes at middle elevations promoted the creation of small patches of native forest. The species occurs in seven natural forest fragments that are isolated in a sea of pastures and *Cryptomeria japonica* plantations and that were highly impacted by invasive plants in the last ten years.

#### Habitat

System: Terrestrial

Habitat specialist: Yes

Habitat (narrative): This species occurs in native forest patches in the islands of Flores, Terceira, Pico, São Jorge, Graciosa and S. Miguel (Azores), with an altitudinal range between 300 and 1200 m.

Trend in extent, area or quality?: Decline (inferred)

Justification for trend: Destruction of habitat for creation of plantations of *Cryptomeria japonica* and the spread of invasive plants (e.g. *Hedychium gardnerianum; Pittosporum undulatum*).

##### Habitat

Habitat importance: Major Importance

Habitats: 1.4. Forest - Temperate

#### Habitat

Habitat importance: Major Importance

Habitats: 1.4. Forest - Temperate

#### Ecology

Size: 0.23-0.37 cm

Generation length (yr): 1

Dependency of single sp?: No

Ecology and traits (narrative): This is a nocturnal predator that lives under bark of native trees and in the soil. Based on seasonal data from SLAM traps obtained in several islands between 2012 and 2016 (Borges et al. 2017b), the adults are active in summer. This is an univoltine species.

#### Threats

Justification for threats: In the past, the species has probably strongly declined due to changes in habitat size and quality (Triantis et al. 2010, Terzopoulou et al. 2015). One of the most important ongoing threats to this species is the managment of pulp plantations of *Cryptomeria japonica* and the spread of invasive plants, namely *Hedychium gardnerianum* and *Pittosporum undulatum* that are changing the habitat structure, namely decreasing the cover of bryophytes and ferns in the soil and promoting the spread of other plants. Based on Ferreira et al. 2016 the habitat will further decline as a consequence of climate change (increasing number of droughts and habitat shifting & alteration).

##### Threats

Threat type: Ongoing

Threats: 2.2.1. Agriculture & aquaculture - Wood & pulp plantations - Small-holder plantations8.1.2. Invasive and other problematic species, genes & diseases - Invasive non-native/alien species/diseases - Named species

##### Threats

Threat type: Future

Threats: 11.1. Climate change & severe weather - Habitat shifting & alteration11.2. Climate change & severe weather - Droughts

#### Threats

Threat type: Ongoing

Threats: 2.2.1. Agriculture & aquaculture - Wood & pulp plantations - Small-holder plantations8.1.2. Invasive and other problematic species, genes & diseases - Invasive non-native/alien species/diseases - Named species

#### Threats

Threat type: Future

Threats: 11.1. Climate change & severe weather - Habitat shifting & alteration11.2. Climate change & severe weather - Droughts

#### Conservation

Justification for conservation actions: The species is not protected by regional law. Its habitat is in regionally protected areas (Natural Parks of Pico, São Jorge, Terceira and S. Miguel). Degraded habitats should be restored and a strategy needs to be developed to address the future threat by climate change. It is necessary a monitoring plan for the invertebrate community in the habitat in order to contribute to the conservation of this species. A habitat management plan is needed and anticipated to be developed during the coming years.

##### Conservation actions

Conservation action type: In Place

Conservation actions: 1. Land/water protection1.1. Land/water protection - Site/area protection2.2. Land/water management - Invasive/problematic species control

##### Conservation actions

Conservation action type: Needed

Conservation actions: 2.1. Land/water management - Site/area management2.2. Land/water management - Invasive/problematic species control2.3. Land/water management - Habitat & natural process restoration4. Education & awareness5.4.3. Law & policy - Compliance and enforcement - Sub-national level

#### Conservation actions

Conservation action type: In Place

Conservation actions: 1. Land/water protection1.1. Land/water protection - Site/area protection2.2. Land/water management - Invasive/problematic species control

#### Conservation actions

Conservation action type: Needed

Conservation actions: 2.1. Land/water management - Site/area management2.2. Land/water management - Invasive/problematic species control2.3. Land/water management - Habitat & natural process restoration4. Education & awareness5.4.3. Law & policy - Compliance and enforcement - Sub-national level

#### Other

##### Use and trade

Use type: International

##### Ecosystem services

Ecosystem service type: Very important

##### Research needed

Research needed: 1.2. Research - Population size, distribution & trends1.3. Research - Life history & ecology2.2. Conservation Planning - Area-based Management Plan3.1. Monitoring - Population trends3.4. Monitoring - Habitat trends

Justification for research needed: Further research is needed into its ecology and life history in order to find extant specimens in additional areas of native forest and in additional islands and obtain information on population size, distribution and trends. It is also necessary a monitoring plan for the invertebrate community in the habitat in order to contribute to perform a species potential recovery plan. Monitoring every ten years using the BALA protocol will inform about habitat quality (see e.g. Gaspar et al. 2011).

#### Use and trade

Use type: International

#### Ecosystem services

Ecosystem service type: Very important

#### Research needed

Research needed: 1.2. Research - Population size, distribution & trends1.3. Research - Life history & ecology2.2. Conservation Planning - Area-based Management Plan3.1. Monitoring - Population trends3.4. Monitoring - Habitat trends

Justification for research needed: Further research is needed into its ecology and life history in order to find extant specimens in additional areas of native forest and in additional islands and obtain information on population size, distribution and trends. It is also necessary a monitoring plan for the invertebrate community in the habitat in order to contribute to perform a species potential recovery plan. Monitoring every ten years using the BALA protocol will inform about habitat quality (see e.g. Gaspar et al. 2011).

#### Viability analysis

Justification for probability: 

### Phytosus schatzmayri

#### Species information

Scientific name: Phytosus schatzmayri

Species authority: Bernhauer, 1941

Common names: Rove beetle (English)

Kingdom: Animalia

Phylum: Arthropoda

Class: Insecta

Order: Coleoptera

Family: Staphylinidae

Region for assessment: Global

#### Editor & Reviewers

##### Reviewers

Reviewers: Anja Danielczak

##### Editor

Editor: Axel Hochkirch

#### Reviewers

Reviewers: Anja Danielczak

#### Editor

Editor: Axel Hochkirch

#### Geographic range

Biogeographic realm: Palearctic

Countries: Portugal

Map of records (Google Earth): Suppl. material 42

Basis of EOO and AOO: Observed

Basis (narrative): It has a very small extent of occurrence (EOO = 0-12 km²) and area of occupancy (AOO = 0-12 km²). Based on the area of thee unique cells of the historical locality.

Min Elevation/Depth (m): 0

Max Elevation/Depth (m): 200

Range description: *Phytosus schatzmayri* is a single-island endemic species from S. Miguel (Azores, Portugal) (Borges et al. 2010). This species is considered very rare and possibly near extinction (Terzopoulou et al. 2015).

#### New occurrences

#### Extent of occurrence

EOO (km2): 0-12

Trend: Stable

Justification for trend: The species is considered possibly extinct in the historical locality possibly due to habitat destruction. Not sampled recently despite some intensive field work (Borges et al. 2016).

Causes ceased?: No

Causes understood?: Yes

Causes reversible?: Unknown

Extreme fluctuations?: Unknown

#### Area of occupancy

Trend: Stable

Justification for trend: Based on the area of thee unique cells of the historical locality. The species is considered extinct in the historical locality possibly due to habitat destruction. Not sampled recently despite some intensive field work (Borges et al. 2016).

Causes ceased?: No

Causes understood?: Yes

Causes reversible?: Unknown

Extreme fluctuations?: Unknown

AOO (km2): 0-12

#### Locations

Number of locations: 0-1

Justification for number of locations: The historical location (Ponta Delgada, S.Miguel island).

Trend: Unknown

Justification for trend: Possibly extinct.

Extreme fluctuations?: Unknown

#### Population

Trend: Stable

Justification for trend: The species is only known from a single subpopulation. According to Terzopoulou et al. 2015 this species is possibly extinct.

Causes ceased?: No

Causes understood?: Yes

Causes reversible?: Unknown

Extreme fluctuations?: Unknown

#### Subpopulations

Number of subpopulations: 0-1

Trend: Stable

Justification for trend: The species is only known from a single subpopulation. According to Terzopoulou et al. 2015 this species is possibly extinct.

Extreme fluctuations?: Unknown

Severe fragmentation?: No

#### Habitat

System: Terrestrial

Habitat specialist: Yes

Habitat (narrative): The species occurred in the native forest of São Miguel Island (Azores), with an altitudinal range between 0 and 200 m. The species is possibly extinct.

Trend in extent, area or quality?: Decline (observed)

Justification for trend: Since the historical record, the native habitat in the island of São Miguel was greatly reduced to accomodate pastures and *Cryptomeria plantations* (Triantis et al. 2010) and the historical locality was possibly destroyed as a consequence of urbanization.

##### Habitat

Habitat importance: Major Importance

Habitats: 1.4. Forest - Temperate

#### Habitat

Habitat importance: Major Importance

Habitats: 1.4. Forest - Temperate

#### Ecology

Size: 0.2-0.28

Generation length (yr): 1

Dependency of single sp?: No

Ecology and traits (narrative): This is a nocturnal predator species usually associated with plant debris in the soil. This is an univoltine species.

#### Threats

Justification for threats: In the past, the species has probably strongly declined due to changes in habitat size. Currently the historical locality was highly modified due to urbanization. Based on Ferreira et al. 2016 the habitat will further decline as a consequence of climate change (increasing number of droughts and habitat shifting & alteration).

##### Threats

Threat type: Ongoing

Threats: 1.1. Residential & commercial development - Housing & urban areas

##### Threats

Threat type: Future

Threats: 11.1. Climate change & severe weather - Habitat shifting & alteration11.2. Climate change & severe weather - Droughts

#### Threats

Threat type: Ongoing

Threats: 1.1. Residential & commercial development - Housing & urban areas

#### Threats

Threat type: Future

Threats: 11.1. Climate change & severe weather - Habitat shifting & alteration11.2. Climate change & severe weather - Droughts

#### Conservation

Justification for conservation actions: The species is not protected by regional law. A strategy needs to be developed to address the future threat by climate change. It is necessary a monitoring plan for the invertebrate community in private gardens in Ponta Delgada. Formal education and awareness is needed to allow future investments in restored habitats invaded by invasive plants.

##### Conservation actions

Conservation action type: Needed

Conservation actions: 2.1. Land/water management - Site/area management2.2. Land/water management - Invasive/problematic species control2.3. Land/water management - Habitat & natural process restoration4. Education & awareness5.4.3. Law & policy - Compliance and enforcement - Sub-national level

#### Conservation actions

Conservation action type: Needed

Conservation actions: 2.1. Land/water management - Site/area management2.2. Land/water management - Invasive/problematic species control2.3. Land/water management - Habitat & natural process restoration4. Education & awareness5.4.3. Law & policy - Compliance and enforcement - Sub-national level

#### Other

##### Use and trade

Use type: International

##### Ecosystem services

Ecosystem service type: Less important

Ecosystem services: 12. Biocontrol

##### Research needed

Research needed: 1.2. Research - Population size, distribution & trends1.3. Research - Life history & ecology3.1. Monitoring - Population trends3.4. Monitoring - Habitat trends

Justification for research needed: Further research is needed into its ecology and life history in order to find extant specimens, possibly in public and private gardens, and obtain information on population size, distribution and trends. It is also necessary a monitoring plan for the invertebrate community in the habitat in order to contribute to perform a species potential recovery plan.

#### Use and trade

Use type: International

#### Ecosystem services

Ecosystem service type: Less important

Ecosystem services: 12. Biocontrol

#### Research needed

Research needed: 1.2. Research - Population size, distribution & trends1.3. Research - Life history & ecology3.1. Monitoring - Population trends3.4. Monitoring - Habitat trends

Justification for research needed: Further research is needed into its ecology and life history in order to find extant specimens, possibly in public and private gardens, and obtain information on population size, distribution and trends. It is also necessary a monitoring plan for the invertebrate community in the habitat in order to contribute to perform a species potential recovery plan.

#### Viability analysis

Justification for probability: 

### Nesotes azoricus

#### Species information

Scientific name: Nesotes azoricus

Species authority: (Crotch, 1867)

Synonyms: *Helops azoricus* Crotch, 1867

Common names: Darkling beetle, False Wireworm, Mealworms (English)

Kingdom: Animalia

Phylum: Arthropoda

Class: Insecta

Order: Coleoptera

Family: Tenebrionidae

Region for assessment: Global

#### Editor & Reviewers

##### Reviewers

Reviewers: Anja Danielczak

##### Editor

Editor: Axel Hochkirch

#### Reviewers

Reviewers: Anja Danielczak

#### Editor

Editor: Axel Hochkirch

#### Geographic range

Biogeographic realm: Palearctic

Countries: Portugal

Map of records (Google Earth): Suppl. material 43

Basis of EOO and AOO: Observed

Basis (narrative): The extent of occurrence (EOO) is 0-8 km^2^ and the maximum area of occupancy (AOO) is 0-8 km^2^.

Min Elevation/Depth (m): 500

Max Elevation/Depth (m): 600

Range description: *Nesotes azoricus* is a single-island endemic species from S. Miguel (Azores, Portugal) (Borges et al. 2010), known from Furnas (S. Miguel). The species is considered possibly extinct (Terzopoulou et al. 2015).

#### New occurrences

#### Extent of occurrence

EOO (km2): 0-8

Trend: Stable

Justification for trend: This species occurs in modified habitats at S. Miguel island (Furnas). Possibly the EOO value is slightly overestimated. The species is considered possibly extinct (Terzopoulou et al. 2015).

Causes ceased?: No

Causes understood?: Yes

Causes reversible?: Unknown

Extreme fluctuations?: Unknown

#### Area of occupancy

Trend: Stable

Justification for trend: This species occurs in a fragment of exotic forest of S. Miguel island (Furnas). Possibly the AOO value is slightly overestimated due to urbanization and pasture intensification. The species is considered possibly extinct (Terzopoulou et al. 2015).

Causes ceased?: No

Causes understood?: Yes

Causes reversible?: Unknown

Extreme fluctuations?: Unknown

AOO (km2): 0-8

#### Locations

Number of locations: 0-1

Justification for number of locations: This species occurs in one single native forest patch in S. Miguel island (Furnas), but is possibly extinct (Terzopoulou et al. 2015)

Trend: Stable

Justification for trend: In the last 50 years major alterations were made in the territory with impacts in native habitats. Only one site left, but the current habitat is highly disturbed and according to Terzopoulou et al. 2015 this species is almost extinct.

Extreme fluctuations?: Unknown

#### Population

Trend: Stable

Justification for trend: The species is only known from a single subpopulation in S. Miguel island. The abundance is unknown and possibly decreasing due to major urban and agriculture changes. According to Terzopoulou et al. 2015 this species is almost extinct.

Causes ceased?: No

Causes understood?: Yes

Causes reversible?: Unknown

Extreme fluctuations?: Unknown

#### Subpopulations

Number of subpopulations: 0-1

Trend: Stable

Justification for trend: The species is only known from a single subpopulation in S. Miguel island. According to Terzopoulou et al. 2015 this species is almost extinct.

Extreme fluctuations?: Unknown

Severe fragmentation?: No

#### Habitat

System: Terrestrial

Habitat specialist: Yes

Habitat (narrative): The species occurs in a single native forest patch in the S. Miguel island (Furnas), with an altitudinal range between 500 and 600 m.

Trend in extent, area or quality?: Decline (observed)

Justification for trend: Destruction of habitat for creation of urban areas, industrial plantations and pastures.

##### Habitat

Habitat importance: Major Importance

Habitats: 1.4. Forest - Temperate

#### Habitat

Habitat importance: Major Importance

Habitats: 1.4. Forest - Temperate

#### Ecology

Generation length (yr): 1

Dependency of single sp?: No

Ecology and traits (narrative): It is a detritivore species that feed of decomposition organic matter and lives in the soil. This is an univoltine species.

#### Threats

Justification for threats: In the past, the species has probably strongly declined due to changes in habitat size and quality (Triantis et al. 2010, Terzopoulou et al. 2015). Main recent past and ongoig threats are destruction of habitat for creation of urban areas, industrial plantations of *Cryptomeria japonica* and pastures and the spread of invasive plants (*Hedychium gardnerianum*) that are changing the habitat structure, namely decreasing the cover of bryophytes and ferns in the soil and promoting the spread of other plants. Based on Ferreira et al. 2016 the habitat will further decline as a consequence of climate change (increasing number of droughts and habitat shifting & alteration).

##### Threats

Threat type: Ongoing

Threats: 1.1. Residential & commercial development - Housing & urban areas2.2.1. Agriculture & aquaculture - Wood & pulp plantations - Small-holder plantations8.1.2. Invasive and other problematic species, genes & diseases - Invasive non-native/alien species/diseases - Named species

##### Threats

Threat type: Future

Threats: 11.1. Climate change & severe weather - Habitat shifting & alteration11.2. Climate change & severe weather - Droughts

#### Threats

Threat type: Ongoing

Threats: 1.1. Residential & commercial development - Housing & urban areas2.2.1. Agriculture & aquaculture - Wood & pulp plantations - Small-holder plantations8.1.2. Invasive and other problematic species, genes & diseases - Invasive non-native/alien species/diseases - Named species

#### Threats

Threat type: Future

Threats: 11.1. Climate change & severe weather - Habitat shifting & alteration11.2. Climate change & severe weather - Droughts

#### Conservation

Justification for conservation actions: The species is not protected by regional law. Its habitat is in a regionally protected area (Natural Park of S. Miguel; Área de Paisagem Protegida das Furnas). Degraded habitats should be restored and a strategy needs to be developed to address the future threat by climate change. It is necessary a monitoring plan for the invertebrate community in the habitat in order to contribute to the conservation of this species.

##### Conservation actions

Conservation action type: In Place

Conservation actions: 1. Land/water protection1.1. Land/water protection - Site/area protection

##### Conservation actions

Conservation action type: Needed

Conservation actions: 2.1. Land/water management - Site/area management2.2. Land/water management - Invasive/problematic species control2.3. Land/water management - Habitat & natural process restoration4. Education & awareness5.4.3. Law & policy - Compliance and enforcement - Sub-national level

#### Conservation actions

Conservation action type: In Place

Conservation actions: 1. Land/water protection1.1. Land/water protection - Site/area protection

#### Conservation actions

Conservation action type: Needed

Conservation actions: 2.1. Land/water management - Site/area management2.2. Land/water management - Invasive/problematic species control2.3. Land/water management - Habitat & natural process restoration4. Education & awareness5.4.3. Law & policy - Compliance and enforcement - Sub-national level

#### Other

##### Use and trade

Use type: International

##### Ecosystem services

Ecosystem service type: Less important

Ecosystem services: 7. Nutrient Cycling

##### Research needed

Research needed: 1.2. Research - Population size, distribution & trends1.3. Research - Life history & ecology2.2. Conservation Planning - Area-based Management Plan3.1. Monitoring - Population trends3.4. Monitoring - Habitat trends

Justification for research needed: Further research is needed into its ecology and life history in order to find extant specimens at Furnas but also in native forests in Pico da Vara, and obtain information on population size, distribution and trends. It is also necessary an area-based management plan and a monitoring plan for the invertebrate community in the habitat in order to contribute to perform a species potential recovery plan. Monitoring every ten years using the BALA protocol will inform about habitat quality (see e.g. Gaspar et al. 2011).

#### Use and trade

Use type: International

#### Ecosystem services

Ecosystem service type: Less important

Ecosystem services: 7. Nutrient Cycling

#### Research needed

Research needed: 1.2. Research - Population size, distribution & trends1.3. Research - Life history & ecology2.2. Conservation Planning - Area-based Management Plan3.1. Monitoring - Population trends3.4. Monitoring - Habitat trends

Justification for research needed: Further research is needed into its ecology and life history in order to find extant specimens at Furnas but also in native forests in Pico da Vara, and obtain information on population size, distribution and trends. It is also necessary an area-based management plan and a monitoring plan for the invertebrate community in the habitat in order to contribute to perform a species potential recovery plan. Monitoring every ten years using the BALA protocol will inform about habitat quality (see e.g. Gaspar et al. 2011).

#### Viability analysis

Justification for probability: 

### Tarphius acuminatus

#### Species information

Scientific name: Tarphius acuminatus

Species authority: Gillerfors, 1987

Common names: Ironclad beetle (English); Escaravelho-cascudo-da-mata (Portuguese)

Kingdom: Animalia

Phylum: Arthropoda

Class: Insecta

Order: Coleoptera

Family: Zopheridae

Taxonomic notes: This species is characterized by (Borges et al. 2017): anterior margin of clypeus markedly arcuate inwards; elytral nodules fair developed with a pattern formula 3/2, 3, 1/2, 0; external row of the lateral margin of pronotum with more or less 15 setae; pronotal and elytral setae long and acuminate (needle shaped); aedeagus as in Fig. 8 (in Gillerfors 1986b).

**Figure 25.**
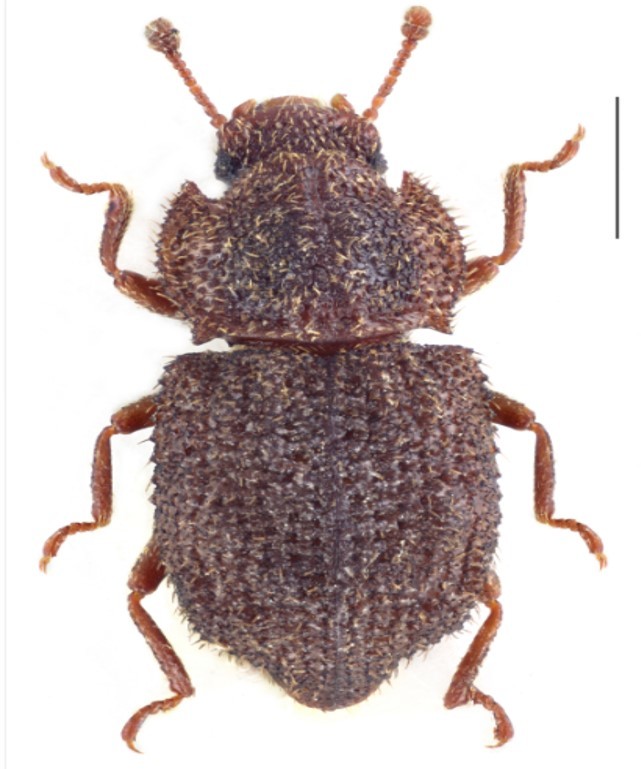
*Tarphius acuminatus* from Cabecinhos (Pico, Azores) (Credit: Erno-Endre Gergely). Scale 0.5 mm.

Figure(s) or Photo(s): Fig. 25

Region for assessment: Global

#### Editor & Reviewers

##### Reviewers

Reviewers: Anja Danielczak

##### Editor

Editor: Axel Hochkirch

#### Reviewers

Reviewers: Anja Danielczak

#### Editor

Editor: Axel Hochkirch

#### Geographic range

Biogeographic realm: Palearctic

Countries: Portugal

Map of records (Google Earth): Suppl. material 44

Basis of EOO and AOO: Observed

Basis (narrative): The extent of occurrence (EOO) is 8 km^2^ and the maximum area of occupancy (AOO) is 8 km^2^.

Min Elevation/Depth (m): 600

Max Elevation/Depth (m): 800

Range description: *Tarphius acuminatus* is a single-island endemic species restricted to Pico island (Azores, Portugal) (Borges et al. 2010,Borges et al. 2017), known from Natural Forest Reserve of Lagoa do Caiado.

#### New occurrences

#### Extent of occurrence

EOO (km2): 8

Trend: Decline (inferred)

Justification for trend: It is a very rare species that occurs in the native forest of Pico island, with a reduced extent of occurrence. The species continues in decline due to habitat loss and the expansion of invasive plant species (Borges et al. 2017).

Causes ceased?: No

Causes understood?: Yes

Causes reversible?: Unknown

Extreme fluctuations?: Unknown

#### Area of occupancy

Trend: Decline (inferred)

Justification for trend: The species occurs in two small patches of native forest, one of it included in a Natural Forest Reserve of Pico. The AOO is overestimated. The species continues in decline due to reduced area of occupancy, habitat loss and the expansion of invasive plant species (Borges et al. 2017).

Causes ceased?: No

Causes understood?: Yes

Causes reversible?: Unknown

Extreme fluctuations?: Unknown

AOO (km2): 8

#### Locations

Number of locations: 2

Justification for number of locations: The species occurs in two patches of native forest in Pico Island.

Trend: Decline (inferred)

Justification for trend: In the last 50 years major alterations were made in the territory with impacts in native habitats. Two locations known that were highly impacted by invasive plants in the last ten years.

Extreme fluctuations?: Unknown

#### Population

Trend: Decline (inferred)

Justification for trend: The species is very rare and only occurs in two small patches of native forest in Pico island. A continuing decline in the number of mature individuals is inferred from monitoring schemes and from the ongoing habitat degradation due to invasions of alien plants (*Hedychium gardnerianum*) (Borges et al. 2017).

Basis for decline: (c) a decline in area of occupancy, extent of occurrence and/or quality of habitat

Causes ceased?: No

Causes understood?: Yes

Causes reversible?: Unknown

Extreme fluctuations?: Unknown

#### Subpopulations

Number of subpopulations: 2

Trend: Decline (inferred)

Justification for trend: The species is very rare and only from two subpopulations that occur in two small patches of native forest in Pico island. A continuing decline in the number of subpopulations is inferred from monitoring schemes and from the ongoing habitat degradation due to invasions of alien plants (*Hedychium gardnerianum*) (Borges et al. 2017).

Extreme fluctuations?: Unknown

Severe fragmentation?: Yes

Justification for fragmentation: Major land-use changes at middle and high elevations promoted the creation of small patches of native forest. The species occurs in two natural forest fragments that are isolated in a sea of semi-natural pastures and that were highly impacted by invasive plants in the last ten years. At least one of the subpopulations is under threat due to the expansion of invasive plants.

#### Habitat

System: Terrestrial

Habitat specialist: Yes

Habitat (narrative): The species is very rare, and it only occurs in two small patches of native forest, in Pico island (Borges et al. 2017). It has an altitudinal range between 600 and 800 m.

Trend in extent, area or quality?: Decline (observed)

Justification for trend: In the past, the species has probably strongly declined due to changes in habitat size and quality (Triantis et al. 2010). Currently the rapid advance of invasive plant species are decreasing the quality of the habitat (Borges et al. 2017).

##### Habitat

Habitat importance: Major Importance

Habitats: 1.4. Forest - Temperate

#### Habitat

Habitat importance: Major Importance

Habitats: 1.4. Forest - Temperate

#### Ecology

Size: 0.33 cm

Generation length (yr): 1

Dependency of single sp?: No

Ecology and traits (narrative): This is a nocturnal fungivorous species that lives in the soil and in dead trunks of endemic trees. This is an univoltine species.

#### Threats

Justification for threats: In the past, the species has probably strongly declined due to changes in habitat size and quality (Triantis et al. 2010). Currently, the rapid advance and expansion of invasive plants species is the major threat (Borges et al. 2017), particularly *Hedychium gardnerianum* that is changing the habitat structure, namely decreasing the cover of bryophytes and ferns in the soil and promoting the spread of other plants. Based on Ferreira et al. 2016 the habitat will further decline as a consequence of climate change (increasing number of droughts and habitat shifting and alteration).

##### Threats

Threat type: Ongoing

Threats: 8.1.2. Invasive and other problematic species, genes & diseases - Invasive non-native/alien species/diseases - Named species

##### Threats

Threat type: Future

Threats: 11.1. Climate change & severe weather - Habitat shifting & alteration11.2. Climate change & severe weather - Droughts

#### Threats

Threat type: Ongoing

Threats: 8.1.2. Invasive and other problematic species, genes & diseases - Invasive non-native/alien species/diseases - Named species

#### Threats

Threat type: Future

Threats: 11.1. Climate change & severe weather - Habitat shifting & alteration11.2. Climate change & severe weather - Droughts

#### Conservation

Justification for conservation actions: The species is not protected by regional law. Its habitat is in a regionally protected area (Natural Reserve of Lagoa do Caiado, in Pico island). Degraded habitats should be restored with the removal of invasive species. A strategy needs also to be developed to address the future threat by climate change. It is necessary a monitoring plan for the invertebrate community in the habitat in order to contribute to the conservation of this species. A habitat management plan is needed and anticipated to be developed during the coming years. Since this species is an icone of the relict native Azorean forests, it is suggested that some awareness measures should be put in practice.

##### Conservation actions

Conservation action type: In Place

Conservation actions: 1. Land/water protection1.1. Land/water protection - Site/area protection2.2. Land/water management - Invasive/problematic species control

##### Conservation actions

Conservation action type: Needed

Conservation actions: 2.1. Land/water management - Site/area management2.2. Land/water management - Invasive/problematic species control2.3. Land/water management - Habitat & natural process restoration4. Education & awareness5.4.3. Law & policy - Compliance and enforcement - Sub-national level

#### Conservation actions

Conservation action type: In Place

Conservation actions: 1. Land/water protection1.1. Land/water protection - Site/area protection2.2. Land/water management - Invasive/problematic species control

#### Conservation actions

Conservation action type: Needed

Conservation actions: 2.1. Land/water management - Site/area management2.2. Land/water management - Invasive/problematic species control2.3. Land/water management - Habitat & natural process restoration4. Education & awareness5.4.3. Law & policy - Compliance and enforcement - Sub-national level

#### Other

##### Use and trade

Use type: International

##### Ecosystem services

Ecosystem service type: Less important

Ecosystem services: 7. Nutrient Cycling

##### Research needed

Research needed: 1.2. Research - Population size, distribution & trends1.3. Research - Life history & ecology2.1. Conservation Planning - Species Action/Recovery Plan2.2. Conservation Planning - Area-based Management Plan3.1. Monitoring - Population trends3.4. Monitoring - Habitat trends

Justification for research needed: Further research is needed into its ecology and life history in order to find extant specimens in more patches of native vegetation at Mistério da Prainha and Caveiro and obtain information on population size, distribution and trends. It is also necessary an area-based management plan and a monitoring plan for the invertebrate community in the habitat in order to contribute to perform a species potential recovery plan. Monitoring every ten years using the BALA protocol will inform about habitat quality (see e.g. Gaspar et al. 2011).

#### Use and trade

Use type: International

#### Ecosystem services

Ecosystem service type: Less important

Ecosystem services: 7. Nutrient Cycling

#### Research needed

Research needed: 1.2. Research - Population size, distribution & trends1.3. Research - Life history & ecology2.1. Conservation Planning - Species Action/Recovery Plan2.2. Conservation Planning - Area-based Management Plan3.1. Monitoring - Population trends3.4. Monitoring - Habitat trends

Justification for research needed: Further research is needed into its ecology and life history in order to find extant specimens in more patches of native vegetation at Mistério da Prainha and Caveiro and obtain information on population size, distribution and trends. It is also necessary an area-based management plan and a monitoring plan for the invertebrate community in the habitat in order to contribute to perform a species potential recovery plan. Monitoring every ten years using the BALA protocol will inform about habitat quality (see e.g. Gaspar et al. 2011).

#### Viability analysis

Justification for probability: 

### Tarphius azoricus

#### Species information

Scientific name: Tarphius azoricus

Species authority: Gillerfors, 1986

Common names: Ironclad beetle (English); Escaravelho-cascudo-da-mata (Portuguese)

Kingdom: Animalia

Phylum: Arthropoda

Class: Insecta

Order: Coleoptera

Family: Zopheridae

Taxonomic notes: This species belongs to the “azoricus+wollastoni+depressus” complex and is characterized by (Borges et al. 2017): anterior margin of clypeus straight or slightly arcuate inwards; external row of the lateral margin of pronotum with 30-34 setae; pronotal and elytral setae short and obtuse (leaf shaped); elytral nodules well developed with a pattern formula 3, 3, 2, 1; aedeagus as in Fig. 7 (in Gillerfors 1986b).

**Figure 26.**
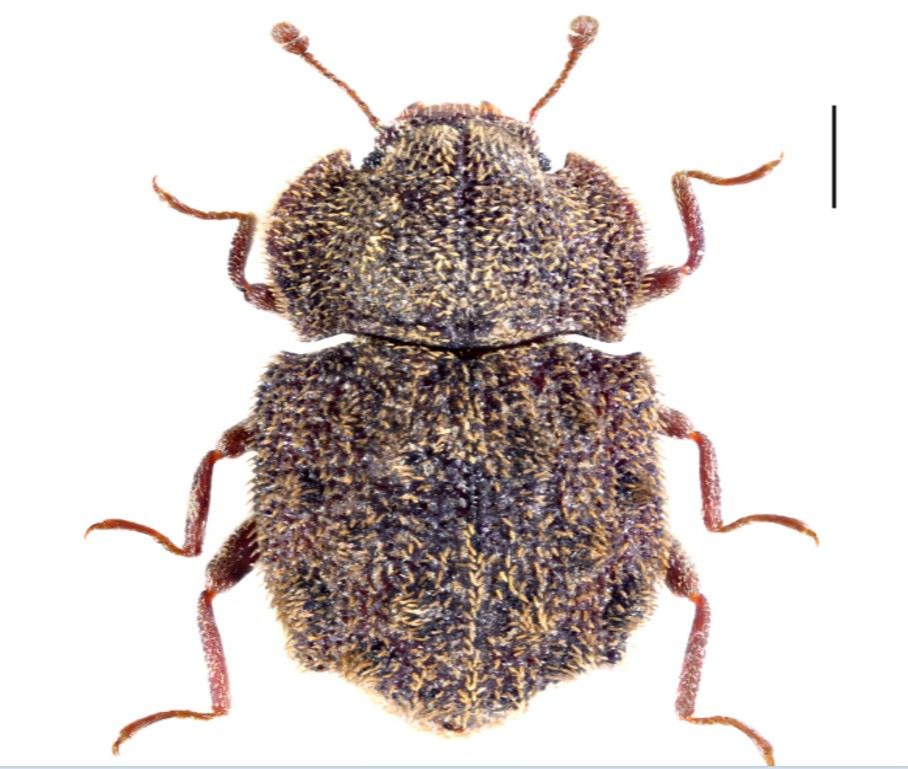
*Tarphius azoricus* from Flores (Azores) (Credit: Erno-Endre Gergely). Scale 0.5 mm.

Figure(s) or Photo(s): Fig. 26

Region for assessment: Global

#### Editor & Reviewers

##### Reviewers

Reviewers: Anja Danielczak

##### Editor

Editor: Axel Hochkirch

#### Reviewers

Reviewers: Anja Danielczak

#### Editor

Editor: Axel Hochkirch

#### Geographic range

Biogeographic realm: Palearctic

Countries: Portugal

Map of records (Google Earth): Suppl. material 45

Basis of EOO and AOO: Observed

Basis (narrative): The extent of occurrence (EOO) is *ca.* 6,300 km^2^ and the maximum area of occupancy (AOO) is 72 km^2^.

Min Elevation/Depth (m): 500

Max Elevation/Depth (m): 1000

Range description: *Tarphius azoricus* is an endemic species from S. Miguel and Flores islands (Azores, Portugal) (Borges et al. 2010, Borges et al. 2017), known from Natural Forest Reserves of Atalhada and Pico da Vara (S. Miguel).

#### New occurrences

#### Extent of occurrence

EOO (km2): 6,300

Trend: Decline (inferred)

Justification for trend: The EOO continues in decline due to *Cryptomeria japonica* pulp plantations management, habitat loss and the expansion of invasive plant species (Borges et al. 2017).

Causes ceased?: No

Causes understood?: Yes

Causes reversible?: Unknown

Extreme fluctuations?: Unknown

#### Area of occupancy

Trend: Decline (inferred)

Justification for trend: The species occurs in native and exotic forests of S. Miguel and Flores islands. Possibly the AOO value is overestimated. The species continues in decline due to *Cryptomeria japonica* pulp plantations management, habitat loss and the expansion of invasive plant species.

Causes ceased?: No

Causes understood?: Yes

Causes reversible?: Unknown

Extreme fluctuations?: Unknown

AOO (km2): 72

#### Locations

Number of locations: 5

Justification for number of locations: The species occurs in a five patches of native and exotic forests of S. Miguel and Flores island.

Trend: Decline (inferred)

Justification for trend: In the last 50 years major alterations were made in the territory with impacts in native habitats. Five locations known that were highly impacted by invasive plants in the last ten years.

Extreme fluctuations?: Unknown

#### Population

Trend: Decline (inferred)

Justification for trend: The species is abundant in native and exotic forests of S. Miguel but very rare in Flores island. A continuing decline in the number of mature individuals is inferred from monitoring schemes and from the ongoing habitat degradation due to invasions of alien plants (namely *Hedychium gardnerianum*) and the *Cryptomeria japonica*a management (Borges et al. 2017).

Basis for decline: (c) a decline in area of occupancy, extent of occurrence and/or quality of habitat

Causes ceased?: No

Causes understood?: Yes

Causes reversible?: Unknown

Extreme fluctuations?: Unknown

#### Subpopulations

Number of subpopulations: 5

Trend: Decline (inferred)

Justification for trend: The species is abundant in native and exotic forests of S. Miguel but subpopulations in Flores island are very rare. A continuing decline in the number of subpopulations is inferred from monitoring schemes and from the ongoing habitat degradation due to invasions of alien plants (namely *Hedychium gardnerianum*) and the *Cryptomeria japonica*a plantations management (Borges et al. 2017).

Extreme fluctuations?: Unknown

Severe fragmentation?: Yes

Justification for fragmentation: Major land-use changes at middle and high elevations promoted the creation of small patches of native forest. The species occurs in three natural forest fragments in S. Miguel and two exotic forest patches in Flores that are isolated in a sea of pastures and *Cryptomeria japonica* plantations and that were highly impacted by invasive plants in the last ten years.

#### Habitat

System: Terrestrial

Habitat specialist: Yes

Habitat (narrative): The species occurs under bark of several trees (subcortical), both endemic and exotic. It also occurs in exotic forests dominated by *Cryptomeria japonica* (Borges et al. 2017).

Trend in extent, area or quality?: Decline (observed)

Justification for trend: In the past, the species has probably strongly declined due to changes in habitat size and quality (Triantis et al. 2010). Currently the rapid advance of invasive plant species are decreasing the quality of the habitat (Borges et al. 2017).

##### Habitat

Habitat importance: Major Importance

Habitats: 1.4. Forest - Temperate14.3. Artificial/Terrestrial - Plantations16. Introduced vegetation

#### Habitat

Habitat importance: Major Importance

Habitats: 1.4. Forest - Temperate14.3. Artificial/Terrestrial - Plantations16. Introduced vegetation

#### Ecology

Size: 0.33 cm

Generation length (yr): 1

Dependency of single sp?: No

Ecology and traits (narrative): This species has an altitudinal range between 500 and 1000 m. It is a nocturnal fungivorous species. This is an univoltine species.

#### Threats

Justification for threats: In the past, the species has probably strongly declined due to changes in habitat size and quality (Triantis et al. 2010). Currently, the rapid advance and expansion of invasive plants species is the major threat (Borges et al. 2017), particularly *Hedychium gardnerianum* that is changing the habitat structure, namely decreasing the cover of bryophytes and ferns in the soil and promoting the spread of other plants. The management of *Cryptomeria japonica* plantations could be also a problem for the subpopulations living in this habitat. Based on Ferreira et al. 2016 the habitat will further decline as a consequence of climate change (increasing number of droughts and habitat shifting and alteration).

##### Threats

Threat type: Ongoing

Threats: 2.2.1. Agriculture & aquaculture - Wood & pulp plantations - Small-holder plantations8.1.2. Invasive and other problematic species, genes & diseases - Invasive non-native/alien species/diseases - Named species

##### Threats

Threat type: Future

Threats: 11.1. Climate change & severe weather - Habitat shifting & alteration11.2. Climate change & severe weather - Droughts

#### Threats

Threat type: Ongoing

Threats: 2.2.1. Agriculture & aquaculture - Wood & pulp plantations - Small-holder plantations8.1.2. Invasive and other problematic species, genes & diseases - Invasive non-native/alien species/diseases - Named species

#### Threats

Threat type: Future

Threats: 11.1. Climate change & severe weather - Habitat shifting & alteration11.2. Climate change & severe weather - Droughts

#### Conservation

Justification for conservation actions: The species is not protected by regional law. Its habitat is in regionally protected areas (Natural Forest Reserves of Atalhada and Pico da Vara in S. Miguel). Degraded habitats should be restored with the removal of invasive species. A strategy needs also to be developed to address the future threat by climate change. It is necessary a monitoring plan for the invertebrate community in the habitat in order to contribute to the conservation of this species. A habitat management plan is needed and anticipated to be developed during the coming years. Since this species is an icone of the relict native Azorean forests, it is suggested that some awareness measures should be put in practice.

##### Conservation actions

Conservation action type: In Place

Conservation actions: 1. Land/water protection1.1. Land/water protection - Site/area protection2.2. Land/water management - Invasive/problematic species control

##### Conservation actions

Conservation action type: Needed

Conservation actions: 2.1. Land/water management - Site/area management2.2. Land/water management - Invasive/problematic species control2.3. Land/water management - Habitat & natural process restoration4. Education & awareness5.4.3. Law & policy - Compliance and enforcement - Sub-national level

#### Conservation actions

Conservation action type: In Place

Conservation actions: 1. Land/water protection1.1. Land/water protection - Site/area protection2.2. Land/water management - Invasive/problematic species control

#### Conservation actions

Conservation action type: Needed

Conservation actions: 2.1. Land/water management - Site/area management2.2. Land/water management - Invasive/problematic species control2.3. Land/water management - Habitat & natural process restoration4. Education & awareness5.4.3. Law & policy - Compliance and enforcement - Sub-national level

#### Other

##### Use and trade

Use type: International

##### Ecosystem services

Ecosystem service type: Less important

Ecosystem services: 7. Nutrient Cycling

##### Research needed

Research needed: 1.2. Research - Population size, distribution & trends1.3. Research - Life history & ecology2.2. Conservation Planning - Area-based Management Plan3.1. Monitoring - Population trends3.4. Monitoring - Habitat trends

Justification for research needed: Further research is needed into its ecology and life history in order to find extant specimens in more patches of native vegetation particularly in Flores and obtain information on population size, distribution and trends. It is also necessary an area-based management plan and a monitoring plan for the invertebrate community in the habitat in order to contribute to perform a species potential recovery plan. Monitoring every ten years using the BALA protocol will inform about habitat quality (see e.g. Gaspar et al. 2011).

#### Use and trade

Use type: International

#### Ecosystem services

Ecosystem service type: Less important

Ecosystem services: 7. Nutrient Cycling

#### Research needed

Research needed: 1.2. Research - Population size, distribution & trends1.3. Research - Life history & ecology2.2. Conservation Planning - Area-based Management Plan3.1. Monitoring - Population trends3.4. Monitoring - Habitat trends

Justification for research needed: Further research is needed into its ecology and life history in order to find extant specimens in more patches of native vegetation particularly in Flores and obtain information on population size, distribution and trends. It is also necessary an area-based management plan and a monitoring plan for the invertebrate community in the habitat in order to contribute to perform a species potential recovery plan. Monitoring every ten years using the BALA protocol will inform about habitat quality (see e.g. Gaspar et al. 2011).

#### Viability analysis

Justification for probability: 

### Tarphius depressus

#### Species information

Scientific name: Tarphius depressus

Species authority: Gillerfors, 1985

Common names: Ironclad beetle (English); Escaravelho-cascudo-da-mata (Portuguese)

Kingdom: Animalia

Phylum: Arthropoda

Class: Insecta

Order: Coleoptera

Family: Zopheridae

Taxonomic notes: This species belongs to the “*azoricus+wollastoni+depressus*” complex and is characterized by (Borges et al. 2017): lateral margins of pronotum straight and sub-parallel in the posterior half; elytral nodules well developed with a pattern formula 3, 3, 2, 1.

**Figure 27.**
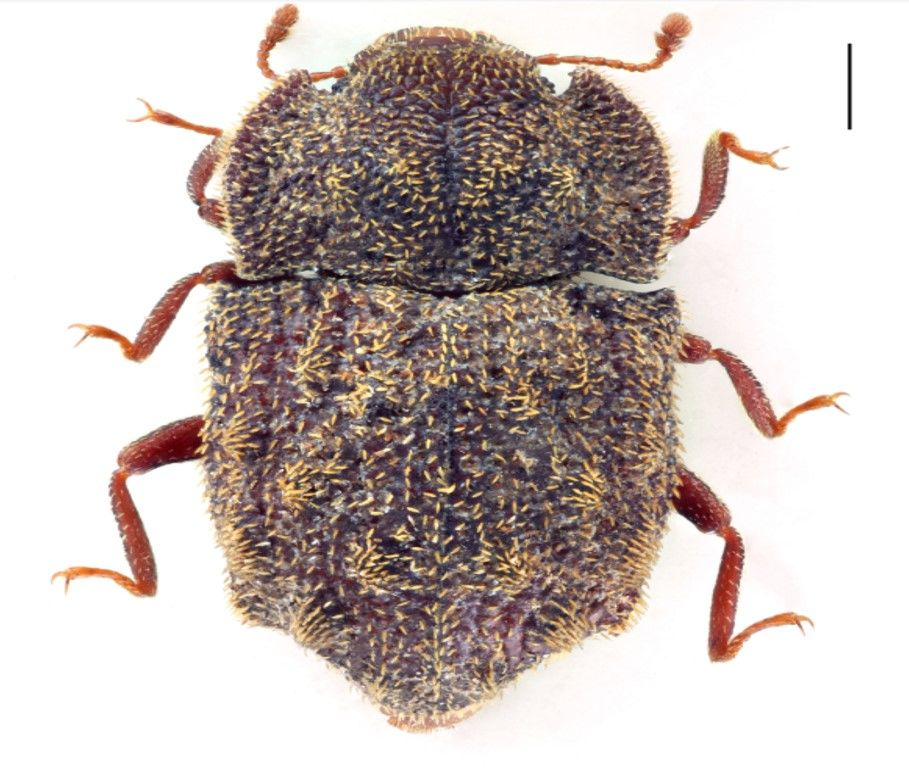
*Tarphius depressus* from Pico Alto (Santa Maria, Azores) (Credit: Erno-Endre Gergely). Scale 0.5 mm.

Figure(s) or Photo(s): Fig. 27

Region for assessment: Global

#### Editor & Reviewers

##### Reviewers

Reviewers: Anja Danielczak

##### Editor

Editor: Axel Hochkirch

#### Reviewers

Reviewers: Anja Danielczak

#### Editor

Editor: Axel Hochkirch

#### Geographic range

Biogeographic realm: Palearctic

Countries: Portugal

Map of records (Google Earth): Suppl. material 46

Basis of EOO and AOO: Observed

Basis (narrative): The extent of occurrence (EOO) is 24 km^2^ and the maximum area of occupancy (AOO) is 24 km^2^.

Min Elevation/Depth (m): 200

Max Elevation/Depth (m): 500

Range description: *Tarphius depressus* is a single-island endemic species restricted to Santa Maria island (Azores, Portugal) (Borges et al. 2010, Borges et al. 2017), known from Natural Forest Reserve of Pico Alto (Santa Maria).

#### New occurrences

#### Extent of occurrence

EOO (km2): 24

Trend: Decline (inferred)

Justification for trend: The species occurs in native and exotic forests of Santa Maria island. The EOO is sligthly overestimated, given that includes habitats not occupied by this species. The species continues in decline due to *Cryptomeria japonica* pulp plantations management, habitat loss and the expansion of invasive plant species (Borges et al. 2017).

Causes ceased?: No

Causes understood?: Yes

Causes reversible?: Unknown

Extreme fluctuations?: Unknown

#### Area of occupancy

Trend: Decline (inferred)

Justification for trend: The species occurs in native and exotic forests of Santa Maria island. Possibly the AOO value is overestimated. The species continues in decline due to *Cryptomeria japonica* pulp plantations management, habitat loss and the expansion of invasive plant species

Causes ceased?: No

Causes understood?: Yes

Causes reversible?: Unknown

Extreme fluctuations?: Unknown

AOO (km2): 24

#### Locations

Number of locations: 6

Justification for number of locations: The species occurs in six patches of native and exotic forests of Santa Maria island.

Trend: Decline (inferred)

Justification for trend: In the last 50 years major alterations were made in the territory with impacts in native habitats. Six locations that were highly impacted by invasive plants in the last ten years and some of them may disappear due to *Cryptomeria japonica* cut.

Extreme fluctuations?: Unknown

#### Population

Trend: Decline (inferred)

Justification for trend: The species is relatively abundant in native and exotic forests of Sta. Maria island. A continuing decline in the number of mature individuals is inferred from monitoring schemes and from the ongoing habitat degradation due to invasions of alien plants (namely *Pittosporum undulatum* and *Hedychium gardnerianum*) and the *Cryptomeria japonica* pulp plantations management (Borges et al. 2017).

Basis for decline: (c) a decline in area of occupancy, extent of occurrence and/or quality of habitat

Causes ceased?: No

Causes understood?: Yes

Causes reversible?: Unknown

Extreme fluctuations?: Unknown

#### Subpopulations

Number of subpopulations: 2

Trend: Decline (inferred)

Justification for trend: The species is abundant in native and exotic forests of Sta. Maria island, having two subpopulations. A continuing decline in the number of subpopulations is inferred from monitoring schemes and from the ongoing habitat degradation due to invasions of alien plants (namely *Pittosporum undulatum* and *Hedychium gardnerianum*) and the *Cryptomeria japonica* pulp plantations management (Borges et al. 2017).

Extreme fluctuations?: Unknown

Severe fragmentation?: Yes

Justification for fragmentation: Major land-use changes at all elevations in S. Maria island promoted the creation of small patches of native and exotic forest. The species occurs in one natural and several small exotic forest fragments that are isolated in a sea of pastures and that were highly impacted by invasive plants in the last ten years.

#### Habitat

System: Terrestrial

Habitat specialist: Yes

Habitat (narrative): The species is relatively abundant. It occurs under the bark of several trees (subcortical), both endemic and exotic. It also occurs in exotic forests dominated by *Cryptomeria japonica* (Borges et al. 2017). This species has an altitudinal range between 200 and 500 m.

Trend in extent, area or quality?: Decline (observed)

Justification for trend: In the past, the species has probably strongly declined due to changes in habitat size and quality (Triantis et al. 2010). Currently the rapid advance of invasive plant species are decreasing the quality of the habitat (Borges et al. 2017).

##### Habitat

Habitat importance: Major Importance

Habitats: 1.4. Forest - Temperate14.3. Artificial/Terrestrial - Plantations

#### Habitat

Habitat importance: Major Importance

Habitats: 1.4. Forest - Temperate14.3. Artificial/Terrestrial - Plantations

#### Ecology

Size: 0.32 cm

Generation length (yr): 1

Dependency of single sp?: No

Ecology and traits (narrative): This is a nocturnal fungivorous species. This is an univoltine species.

#### Threats

Justification for threats: In the past, the species has probably strongly declined due to changes in habitat size and quality (Triantis et al. 2010). Currently, the rapid advance and expansion of invasive plants species is the major threat (Borges et al. 2017), particularly *Hedychium gardnerianum* that is changing the habitat structure, namely decreasing the cover of bryophytes and ferns in the soil and promoting the spread of other plants. The management of *Cryptomeria* plantations could be also a problem for the subpopulations living in this habitat. Based on Ferreira et al. 2016 the habitat will further decline as a consequence of climate change (increasing number of droughts and habitat shifting and alteration).

##### Threats

Threat type: Ongoing

Threats: 2.2.1. Agriculture & aquaculture - Wood & pulp plantations - Small-holder plantations8.1.2. Invasive and other problematic species, genes & diseases - Invasive non-native/alien species/diseases - Named species

##### Threats

Threat type: Future

Threats: 11.1. Climate change & severe weather - Habitat shifting & alteration11.2. Climate change & severe weather - Droughts

#### Threats

Threat type: Ongoing

Threats: 2.2.1. Agriculture & aquaculture - Wood & pulp plantations - Small-holder plantations8.1.2. Invasive and other problematic species, genes & diseases - Invasive non-native/alien species/diseases - Named species

#### Threats

Threat type: Future

Threats: 11.1. Climate change & severe weather - Habitat shifting & alteration11.2. Climate change & severe weather - Droughts

#### Conservation

Justification for conservation actions: The species is not protected by regional law. Its habitat is in a regionally protected area (Natural Forest Reserve of Pico Alto in Santa Maria island). Degraded habitats should be restored with the removal of invasive species. A strategy needs also to be developed to address the future threat by climate change. It is necessary a monitoring plan for the invertebrate community in the habitat in order to contribute to the conservation of this species. A habitat management plan is needed and anticipated to be developed during the coming years. Since this species is an icone of the relict native Azorean forests, it is suggested that some awareness measures should be put in practice.

##### Conservation actions

Conservation action type: In Place

Conservation actions: 1. Land/water protection1.1. Land/water protection - Site/area protection2.2. Land/water management - Invasive/problematic species control

##### Conservation actions

Conservation action type: Needed

Conservation actions: 2.1. Land/water management - Site/area management2.2. Land/water management - Invasive/problematic species control2.3. Land/water management - Habitat & natural process restoration4. Education & awareness5.4.3. Law & policy - Compliance and enforcement - Sub-national level

#### Conservation actions

Conservation action type: In Place

Conservation actions: 1. Land/water protection1.1. Land/water protection - Site/area protection2.2. Land/water management - Invasive/problematic species control

#### Conservation actions

Conservation action type: Needed

Conservation actions: 2.1. Land/water management - Site/area management2.2. Land/water management - Invasive/problematic species control2.3. Land/water management - Habitat & natural process restoration4. Education & awareness5.4.3. Law & policy - Compliance and enforcement - Sub-national level

#### Other

##### Use and trade

Use type: International

##### Ecosystem services

Ecosystem service type: Less important

Ecosystem services: 7. Nutrient Cycling

##### Research needed

Research needed: 1.2. Research - Population size, distribution & trends1.3. Research - Life history & ecology2.2. Conservation Planning - Area-based Management Plan3.1. Monitoring - Population trends3.4. Monitoring - Habitat trends

Justification for research needed: Further research is needed into its ecology and life history in order to find extant specimens in more patches of exotic vegetation around Pico Alto and obtain information on population size, distribution and trends. It is also necessary an area-based management plan and a monitoring plan for the invertebrate community in the habitat in order to contribute to perform a species potential recovery plan. Monitoring every ten years using the BALA protocol will inform about habitat quality (see e.g. Gaspar et al. 2011).

#### Use and trade

Use type: International

#### Ecosystem services

Ecosystem service type: Less important

Ecosystem services: 7. Nutrient Cycling

#### Research needed

Research needed: 1.2. Research - Population size, distribution & trends1.3. Research - Life history & ecology2.2. Conservation Planning - Area-based Management Plan3.1. Monitoring - Population trends3.4. Monitoring - Habitat trends

Justification for research needed: Further research is needed into its ecology and life history in order to find extant specimens in more patches of exotic vegetation around Pico Alto and obtain information on population size, distribution and trends. It is also necessary an area-based management plan and a monitoring plan for the invertebrate community in the habitat in order to contribute to perform a species potential recovery plan. Monitoring every ten years using the BALA protocol will inform about habitat quality (see e.g. Gaspar et al. 2011).

#### Viability analysis

Justification for probability: 

### Tarphius floresensis

#### Species information

Scientific name: Tarphius floresensis

Species authority: Borges & Serrano, 2017

Common names: Ironclad beetle (English); Escaravelho-cascudo-da-mata (Portuguese)

Kingdom: Animalia

Phylum: Arthropoda

Class: Insecta

Order: Coleoptera

Family: Zopheridae

Taxonomic notes: The species belongs to the “*tornvalli*” complex and is characterized by (Borges et al. 2017): pronotal and elytral setae recumbent and slightly acuminate; external row of the lateral margin of pronotum with 27-28 setae.

**Figure 28.**
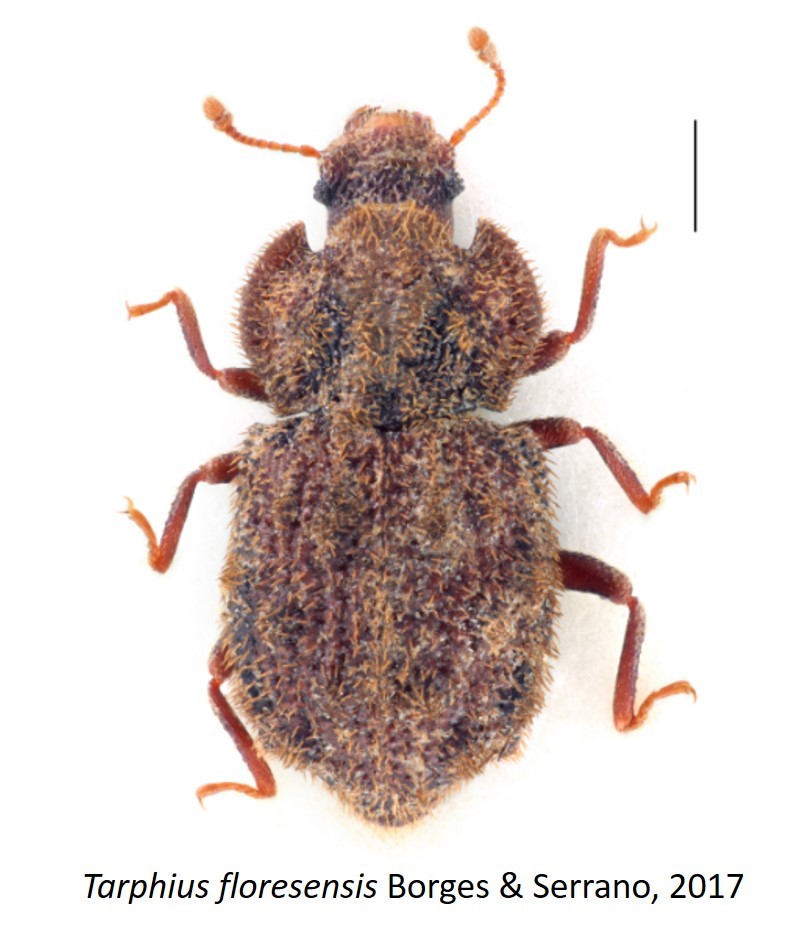
*Tarphius floresensis* from Pico Alto (Flores, Azores) (Credit: Erno-Endre Gergely). Scale 0.5 mm.

Figure(s) or Photo(s): Fig. 28

Region for assessment: Global

#### Editor & Reviewers

##### Reviewers

Reviewers: Anja Danielczak

##### Editor

Editor: Axel Hochkirch

#### Reviewers

Reviewers: Anja Danielczak

#### Editor

Editor: Axel Hochkirch

#### Geographic range

Biogeographic realm: Palearctic

Countries: Portugal

Map of records (Google Earth): Suppl. material 47

Basis of EOO and AOO: Observed

Basis (narrative): The extent of occurrence (EOO) is *ca.* 90 km^2^ and the maximum area of occupancy (AOO) is 72 km^2^.

Min Elevation/Depth (m): 300

Max Elevation/Depth (m): 1000

Range description: *Tarphius floresensis* is a single-island endemic species restricted to Flores island (Azores, Portugal) Borges et al. 2010, Borges et al. 2017), known from Natural Forest Reserves of Morro alto e Pico da Sé and Caldeiras Funda e Rasa.

#### New occurrences

#### Extent of occurrence

EOO (km2): 90

Trend: Decline (inferred)

Justification for trend: This species occurs in native forests included in the two Natural Forest Reserves of Flores island. It also occurs in exotic forest patches (mainly dominated by *Cryptomeria japonica* and *Acacia* spp.). The Extent of Occurrence is sligthly overestimated, given that includes habitats not occupied by this species. The species continues in decline due to native forest destruction, *Cryptomeria japonica* pulp plantations management and due to habitat degradation by the rapid advance of invasive plant species.

Causes ceased?: No

Causes understood?: Yes

Causes reversible?: Unknown

Extreme fluctuations?: Unknown

#### Area of occupancy

Trend: Decline (inferred)

Justification for trend: The species occurs in native forests included in the two Natural Forest Reserves of Flores island. Possibly the AOO value is slighly overestimated, being the AOO with native forest only around 20 km². The species continues in decline due to native forest destruction, *Cryptomeria japonica* pulp plantations management and due to habitat degradation by the rapid advance of invasive plant species.

Causes ceased?: No

Causes understood?: Yes

Causes reversible?: Unknown

Extreme fluctuations?: Unknown

AOO (km2): 72

#### Locations

Number of locations: 10

Justification for number of locations: The species occurs in several native forest patches included in Natural Reserves and in exotic forest patches, in the Flores island.

Trend: Decline (inferred)

Justification for trend: In the last 50 years major alterations were made in the territory with impacts in native habitats. Ten locations that were highly impacted by invasive plants in the last ten years and some of them may disappear soon due to *Cryptomeria japonica* removal.

Extreme fluctuations?: Unknown

#### Population

Trend: Decline (observed)

Justification for trend: The species is abundant, particularly in the well preserved patches of native forests of Flores island. A continuing decline in the number of mature individuals is inferred from monitoring schemes and from the ongoing habitat degradation due to invasions of alien plants (*Hedychium gardnerianum* and *Hydrangea macrophylla*) (Borges et al. 2017).

Basis for decline: (c) a decline in area of occupancy, extent of occurrence and/or quality of habitat

Causes ceased?: No

Causes understood?: Yes

Causes reversible?: Unknown

Extreme fluctuations?: Unknown

#### Subpopulations

Number of subpopulations: 5

Trend: Decline (observed)

Justification for trend: The species is abundant in all five subpopulations, particularly in the two well preserved patches of native forests of Flores island. A continuing decline in the number of subpopulations is inferred from monitoring schemes and from the ongoing habitat degradation due to invasions of alien plants (*Hedychium gardnerianum* and *Hydrangea macrophylla*) and the removal of *Cryptomeria japonica* plantations (Borges et al. 2017).

Extreme fluctuations?: Unknown

Severe fragmentation?: Yes

Justification for fragmentation: Major land-use changes at all elevations in Flores island promoted the creation of small patches of native forest. The species occurs in two natural forest fragments and three *Cryptomeria japonica* plantations that are isolated in a sea of pastures and that were highly impacted by invasive plants in the last ten years.

#### Habitat

System: Terrestrial

Habitat specialist: Yes

Habitat (narrative): The species is particularly abundant, namely this species lives in the soil and occuring in some of the larger an well preserved patches of native forests of Flores island. It also occurs under the bark of endemic and exotic trees (Borges et al. 2017). This species has an altitudinal range between 300 and 1000 m.

Trend in extent, area or quality?: Decline (observed)

Justification for trend: In the past, the species has probably strongly declined due to changes in habitat size and quality (Triantis et al. 2010). Currently the rapid advance of invasive plant species are decreasing the quality of the habitat (Borges et al. 2017).

##### Habitat

Habitat importance: Major Importance

Habitats: 1.4. Forest - Temperate14.3. Artificial/Terrestrial - Plantations16. Introduced vegetation

#### Habitat

Habitat importance: Major Importance

Habitats: 1.4. Forest - Temperate14.3. Artificial/Terrestrial - Plantations16. Introduced vegetation

#### Ecology

Size: 0.27 cm

Generation length (yr): 1

Dependency of single sp?: No

Ecology and traits (narrative): This is a nocturnal fungivorous species. Univoltine species.

#### Threats

Justification for threats: In the past, the species has probably strongly declined due to changes in habitat size and quality (Triantis et al. 2010). Currently, the rapid advance and expansion of invasive plants species is the major threat (Borges et al. 2017), particularly *Hedychium gardnerianum* and *Hydrangea macrophylla* since are changing the habitat structure, namely decreasing the cover of bryophytes and ferns in the soil and promoting the spread of other plants. The management of *Cryptomeria japonica* plantations could be also a problem for the subpopulations living in this habitat. Based on Ferreira et al. 2016 the habitat will further decline as a consequence of climate change (increasing number of droughts and habitat shifting and alteration).

##### Threats

Threat type: Ongoing

Threats: 2.2.1. Agriculture & aquaculture - Wood & pulp plantations - Small-holder plantations8.1.2. Invasive and other problematic species, genes & diseases - Invasive non-native/alien species/diseases - Named species

##### Threats

Threat type: Future

Threats: 11.1. Climate change & severe weather - Habitat shifting & alteration11.2. Climate change & severe weather - Droughts

#### Threats

Threat type: Ongoing

Threats: 2.2.1. Agriculture & aquaculture - Wood & pulp plantations - Small-holder plantations8.1.2. Invasive and other problematic species, genes & diseases - Invasive non-native/alien species/diseases - Named species

#### Threats

Threat type: Future

Threats: 11.1. Climate change & severe weather - Habitat shifting & alteration11.2. Climate change & severe weather - Droughts

#### Conservation

Justification for conservation actions: The species is not protected by regional law. Its habitat is in a regionally protected area (Natural Forest Reserves of Morro alto e Pico da Sé and Caldeiras Funda e Rasa, in Flores island). Degraded habitats should be restored with the removal of invasive species. A strategy needs also to be developed to address the future threat by climate change. It is necessary a monitoring plan for the invertebrate community in the habitat in order to contribute to the conservation of this species. A habitat management plan is needed and anticipated to be developed during the coming years. Since this species is an icone of the relict native Azorean forests, it is suggested that some awareness measures should be put in practice.

##### Conservation actions

Conservation action type: In Place

Conservation actions: 1. Land/water protection1.1. Land/water protection - Site/area protection2.2. Land/water management - Invasive/problematic species control

##### Conservation actions

Conservation action type: Needed

Conservation actions: 2.1. Land/water management - Site/area management2.2. Land/water management - Invasive/problematic species control2.3. Land/water management - Habitat & natural process restoration4. Education & awareness5.4.3. Law & policy - Compliance and enforcement - Sub-national level

#### Conservation actions

Conservation action type: In Place

Conservation actions: 1. Land/water protection1.1. Land/water protection - Site/area protection2.2. Land/water management - Invasive/problematic species control

#### Conservation actions

Conservation action type: Needed

Conservation actions: 2.1. Land/water management - Site/area management2.2. Land/water management - Invasive/problematic species control2.3. Land/water management - Habitat & natural process restoration4. Education & awareness5.4.3. Law & policy - Compliance and enforcement - Sub-national level

#### Other

##### Use and trade

Use type: International

##### Ecosystem services

Ecosystem service type: Less important

Ecosystem services: 7. Nutrient Cycling

##### Research needed

Research needed: 1.2. Research - Population size, distribution & trends1.3. Research - Life history & ecology2.2. Conservation Planning - Area-based Management Plan3.1. Monitoring - Population trends3.4. Monitoring - Habitat trends

Justification for research needed: Further research is needed into its ecology and life history in order to find extant specimens in more patches of native vegetationbut also at lower elevation modified exotic forests and obtain information on population size, distribution and trends. It is also necessary an area-based management plan and a monitoring plan for the invertebrate community in the habitat in order to contribute to perform a species potential recovery plan. Monitoring every ten years using the BALA protocol will inform about habitat quality (see e.g. Gaspar et al. 2011).

#### Use and trade

Use type: International

#### Ecosystem services

Ecosystem service type: Less important

Ecosystem services: 7. Nutrient Cycling

#### Research needed

Research needed: 1.2. Research - Population size, distribution & trends1.3. Research - Life history & ecology2.2. Conservation Planning - Area-based Management Plan3.1. Monitoring - Population trends3.4. Monitoring - Habitat trends

Justification for research needed: Further research is needed into its ecology and life history in order to find extant specimens in more patches of native vegetationbut also at lower elevation modified exotic forests and obtain information on population size, distribution and trends. It is also necessary an area-based management plan and a monitoring plan for the invertebrate community in the habitat in order to contribute to perform a species potential recovery plan. Monitoring every ten years using the BALA protocol will inform about habitat quality (see e.g. Gaspar et al. 2011).

#### Viability analysis

Justification for probability: 

### Tarphius furtadoi

#### Species information

Scientific name: Tarphius furtadoi

Species authority: Borges & Serrano, 2017

Common names: Ironclad beetle (English); Escaravelho-cascudo-da-mata (Portuguese)

Kingdom: Animalia

Phylum: Arthropoda

Class: Insecta

Order: Coleoptera

Family: Zopheridae

Taxonomic notes: The species belongs to the “*tornvalli*” complex and is characterized by (Borges et al. 2017): setae acuminate with lateral sides slightly rounded and maximum width at middle; external row of the lateral margin of pronotum with 30 or more setae.

**Figure 29.**
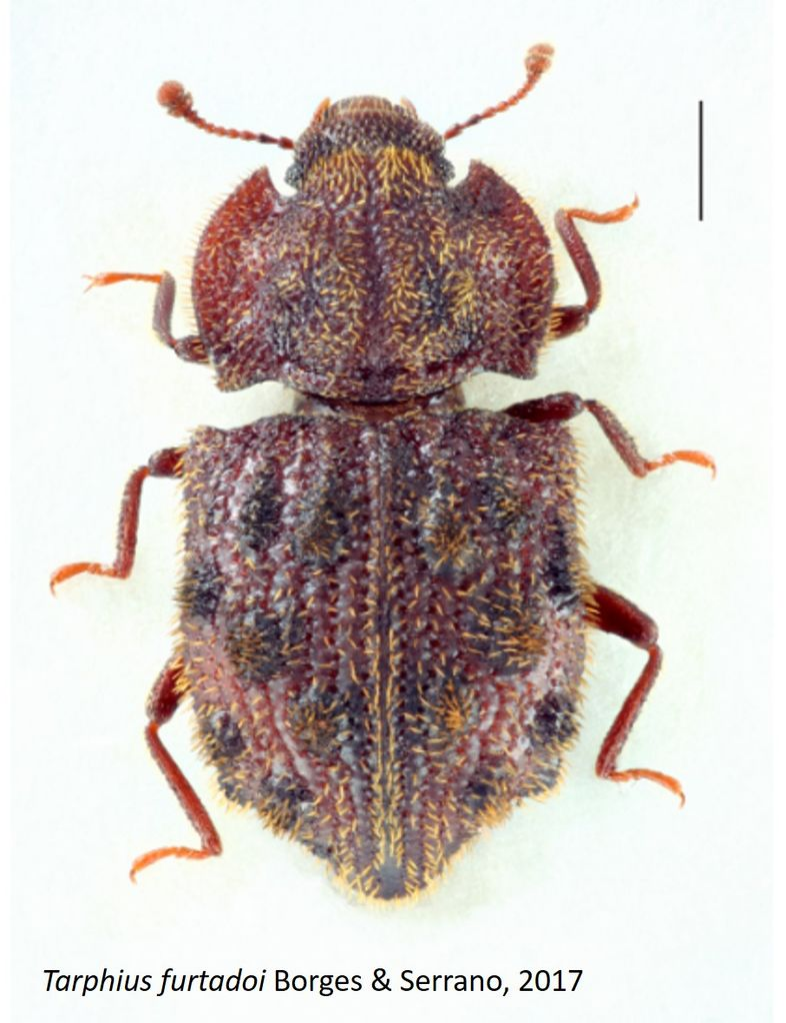
*Tarphius furtadoi* from São Jorge (Azores) (Credit: Erno-Endre Gergely). Scale 0.5 mm.

Figure(s) or Photo(s): Fig. 29

Region for assessment: Global

#### Editor & Reviewers

##### Reviewers

Reviewers: Anja Danielczak

##### Editor

Editor: Axel Hochkirch

#### Reviewers

Reviewers: Anja Danielczak

#### Editor

Editor: Axel Hochkirch

#### Geographic range

Biogeographic realm: Palearctic

Countries: Portugal

Map of records (Google Earth): Suppl. material 48

Basis of EOO and AOO: Observed

Basis (narrative): The extent of occurrence (EOO) is *ca.* 890 km^2^ and the maximum area of occupancy (AOO) is 48 km^2^.

Min Elevation/Depth (m): 250

Max Elevation/Depth (m): 1000

Range description: *Tarphius furtadoi* is an endemic species occurring in S. Jorge, Pico and Faial islands (Azores, Portugal) (Borges et al. 2010, Borges et al. 2017), known from Natural Forest Reserves of Caveiro, Lagoa do Caiado and Mistério da Prainha (Pico island); Pico Pinheiro and Topo (S. Jorge island) and Cabeço do Fogo and Caldeira do Faial (Faial island).

#### New occurrences

#### Extent of occurrence

EOO (km2): 890

Trend: Decline (inferred)

Justification for trend: The EOO includes large areas of unsuitable habitats. The species continues in decline due to native forest destruction, *Cryptomeria japonica* and *Acacia* spp. plantations management and due to habitat degradation by the rapid advance of invasive plant species.

Causes ceased?: No

Causes understood?: Yes

Causes reversible?: Unknown

Extreme fluctuations?: Unknown

#### Area of occupancy

Trend: Decline (inferred)

Justification for trend: The species occurs in native forests included in Natural Forest Reserves of S. Jorge, Pico and Faial islands. Possibly the AOO value is overestimated. The species continues in decline due to native forest destruction, *Cryptomeria japonica* and *Acacia* spp. plantations management and due to habitat degradation by the rapid advance of invasive plant species.

Causes ceased?: No

Causes understood?: Yes

Causes reversible?: Unknown

Extreme fluctuations?: Unknown

AOO (km2): 48

#### Locations

Number of locations: 8

Justification for number of locations: The species occurs in several native forest patches included in Natural Forest Reserves and in exotic forest patches, in the S. Jorge, Pico and Faial islands.

Trend: Decline (inferred)

Justification for trend: In the last 50 years major alterations were made in the territory with impacts in native habitats. Eigth locations that were highly impacted by invasive plants in the last ten years and some may disappear in near future due to removal of exotic forest patches for pasture implementation.

Extreme fluctuations?: Unknown

#### Population

Trend: Decline (inferred)

Justification for trend: The species is abundant, particularly in the well preserved patches of native forests of S. Jorge, Pico and Faial islands. A continuing decline in the number of mature individuals is inferred from monitoring schemes and from the ongoing habitat degradation due to invasions of alien plants (Borges et al. 2017).

Basis for decline: (c) a decline in area of occupancy, extent of occurrence and/or quality of habitat

Causes ceased?: No

Causes understood?: Yes

Causes reversible?: Unknown

Extreme fluctuations?: Unknown

#### Subpopulations

Number of subpopulations: 8

Trend: Decline (inferred)

Justification for trend: The species is abundant, particularly in the subpopulations occurring in the well preserved patches of native forests of S. Jorge, Pico and Faial islands. A continuing decline in the number of subpopulations is inferred from monitoring schemes and from the ongoing habitat degradation due to invasions of alien plants (Borges et al. 2017).

Extreme fluctuations?: Unknown

Severe fragmentation?: Yes

Justification for fragmentation: Major land-use changes at middle and high elevations promoted the creation of small patches of native forest. The species occurs in six natural forest fragments and two exotic patches of exotic forestthat are isolated in a sea of pastures and *Cryptomeria japonica* plantations and that were highly impacted by invasive plants in the last ten years.

#### Habitat

System: Terrestrial

Habitat specialist: Yes

Habitat (narrative): The species is particularly abundant, namely this species lives in the soil and occuring in some of the larger an well preserved patches of native forests of S. Jorge, Pico and Faial islands. It also occurs under the bark of dead wood of endemic and exotic trees (Borges et al. 2017). This species has an altitudinal range between 250 and 1000 m.

Trend in extent, area or quality?: Decline (observed)

Justification for trend: In the past, the species has probably strongly declined due to changes in habitat size and quality (Triantis et al. 2010). Currently the rapid advance of invasive plant species are decreasing the quality of the habitat (Borges et al. 2017).

##### Habitat

Habitat importance: Major Importance

Habitats: 1.4. Forest - Temperate14.3. Artificial/Terrestrial - Plantations16. Introduced vegetation

#### Habitat

Habitat importance: Major Importance

Habitats: 1.4. Forest - Temperate14.3. Artificial/Terrestrial - Plantations16. Introduced vegetation

#### Ecology

Size: 0.29 cm

Generation length (yr): 1

Dependency of single sp?: No

Ecology and traits (narrative): This is a nocturnal fungivorous species. This is an univoltine species.

#### Threats

Justification for threats: In the past, the species has probably strongly declined due to changes in habitat size and quality (Triantis et al. 2010). Currently, the rapid advance and expansion of invasive plants species is the major threat (Borges et al. 2017), particularly *Hedychium gardnerianum* and *Pittosporum undulatum* since are changing the habitat structure, namely decreasing the cover of bryophytes and ferns in the soil and promoting the spread of other plants. The management of *Cryptomeria japonica* plantations could be also a problem for the subpopulations living in this habitat. Based on Ferreira et al. 2016 the habitat will further decline as a consequence of climate change (increasing number of droughts and habitat shifting and alteration). The removal of some patches with exotic vegetation for pasture implementation may also be a threat for some subpopulations occurring in this marginal habitat.

##### Threats

Threat type: Ongoing

Threats: 2.2.1. Agriculture & aquaculture - Wood & pulp plantations - Small-holder plantations8.1.2. Invasive and other problematic species, genes & diseases - Invasive non-native/alien species/diseases - Named species

##### Threats

Threat type: Future

Threats: 2.3. Agriculture & aquaculture - Livestock farming & ranching11.1. Climate change & severe weather - Habitat shifting & alteration11.2. Climate change & severe weather - Droughts

#### Threats

Threat type: Ongoing

Threats: 2.2.1. Agriculture & aquaculture - Wood & pulp plantations - Small-holder plantations8.1.2. Invasive and other problematic species, genes & diseases - Invasive non-native/alien species/diseases - Named species

#### Threats

Threat type: Future

Threats: 2.3. Agriculture & aquaculture - Livestock farming & ranching11.1. Climate change & severe weather - Habitat shifting & alteration11.2. Climate change & severe weather - Droughts

#### Conservation

Justification for conservation actions: The species is not protected by regional law. Its habitat is in regionally protected areas (Natural Forest Reserves of Mistério da Prainha, Caveiro and Caiado in Pico island; Pico Pinheiro and Topo in S. Jorge island and Cabeço do Fogo and Caldeira do Faial in Faial island). Degraded habitats should be restored with the removal of invasive species. A strategy needs also to be developed to address the future threat by climate change. It is necessary a monitoring plan for the invertebrate community in the habitat in order to contribute to the conservation of this species. A habitat management plan is needed and anticipated to be developed during the coming years. Since this species is an icone of the relict native Azorean forests, it is suggested that some awareness measures should be put in practice.

##### Conservation actions

Conservation action type: In Place

Conservation actions: 1. Land/water protection1.1. Land/water protection - Site/area protection2.2. Land/water management - Invasive/problematic species control

##### Conservation actions

Conservation action type: Needed

Conservation actions: 2.1. Land/water management - Site/area management2.2. Land/water management - Invasive/problematic species control2.3. Land/water management - Habitat & natural process restoration4. Education & awareness5.4.3. Law & policy - Compliance and enforcement - Sub-national level

#### Conservation actions

Conservation action type: In Place

Conservation actions: 1. Land/water protection1.1. Land/water protection - Site/area protection2.2. Land/water management - Invasive/problematic species control

#### Conservation actions

Conservation action type: Needed

Conservation actions: 2.1. Land/water management - Site/area management2.2. Land/water management - Invasive/problematic species control2.3. Land/water management - Habitat & natural process restoration4. Education & awareness5.4.3. Law & policy - Compliance and enforcement - Sub-national level

#### Other

##### Use and trade

Use type: International

##### Ecosystem services

Ecosystem service type: Less important

Ecosystem services: 7. Nutrient Cycling

##### Research needed

Research needed: 1.2. Research - Population size, distribution & trends1.3. Research - Life history & ecology2.2. Conservation Planning - Area-based Management Plan3.1. Monitoring - Population trends3.4. Monitoring - Habitat trends

Justification for research needed: Further research is needed into its ecology and life history in order to find extant specimens in additional native forest fragments and obtain information on population size, distribution and trends. It is also necessary an area-based management plan and a monitoring plan for the invertebrate community in the habitat in order to contribute to perform a species potential recovery plan. Monitoring every ten years using the BALA protocol will inform about habitat quality (see e.g. Gaspar et al. 2011).

#### Use and trade

Use type: International

#### Ecosystem services

Ecosystem service type: Less important

Ecosystem services: 7. Nutrient Cycling

#### Research needed

Research needed: 1.2. Research - Population size, distribution & trends1.3. Research - Life history & ecology2.2. Conservation Planning - Area-based Management Plan3.1. Monitoring - Population trends3.4. Monitoring - Habitat trends

Justification for research needed: Further research is needed into its ecology and life history in order to find extant specimens in additional native forest fragments and obtain information on population size, distribution and trends. It is also necessary an area-based management plan and a monitoring plan for the invertebrate community in the habitat in order to contribute to perform a species potential recovery plan. Monitoring every ten years using the BALA protocol will inform about habitat quality (see e.g. Gaspar et al. 2011).

#### Viability analysis

Justification for probability: 

### Tarphius gabrielae

#### Species information

Scientific name: Tarphius gabrielae

Species authority: Borges & Serrano, 2017

Common names: Ironclad beetle (English); Escaravelho-cascudo-da-mata (Portuguese)

Kingdom: Animalia

Phylum: Arthropoda

Class: Insecta

Order: Coleoptera

Family: Zopheridae

Taxonomic notes: This species belongs to the “*azoricus+wollastoni+depressus*” complex and is characterized by (Borges et al. 2017): lateral margins of pronotum slightly arcuate between the anterior and the hind angles; pronotal setae obtuse, rigid and semi-erect; elytral nodules well developed with a pattern formula 3, 3, 2, 1.

**Figure 30.**
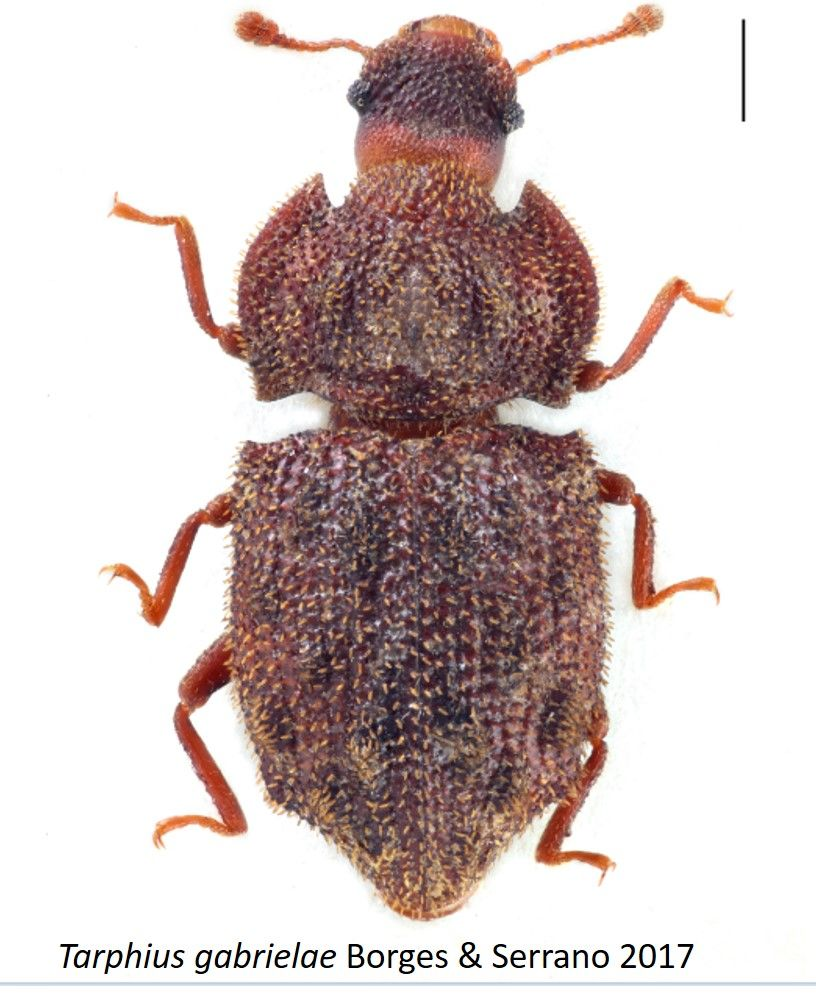
*Tarphius gabrielae* from Lagoa do Caiado (Pico, Azores) (Credit: Erno-Endre Gergely). Scale 0.5 mm.

Figure(s) or Photo(s): Fig. 30

Region for assessment: Global

#### Editor & Reviewers

##### Reviewers

Reviewers: Anja Danielczak

##### Editor

Editor: Axel Hochkirch

#### Reviewers

Reviewers: Anja Danielczak

#### Editor

Editor: Axel Hochkirch

#### Geographic range

Biogeographic realm: Palearctic

Countries: Portugal

Map of records (Google Earth): Suppl. material 49

Basis of EOO and AOO: Observed

Basis (narrative): The extent of occurrence (EOO) is 8 km^2^ and the maximum area of occupancy (AOO) is 8 km^2^.

Min Elevation/Depth (m): 700

Max Elevation/Depth (m): 850

Range description: *Tarphius gabrielae* is a single-island endemic species restricted to Pico island (Azores, Portugal) (Borges et al. 2017), known from Natural Forest Reserve of Lagoa do Caiado.

#### New occurrences

#### Extent of occurrence

EOO (km2): 8

Trend: Decline (inferred)

Justification for trend: This is a very rare species that occurs in the native forest of Pico island, with a reduced extent of occurrence. The species continues in decline due to habitat loss and the expansion of invasive plant species (Borges et al. 2017).

Causes ceased?: No

Causes understood?: Yes

Causes reversible?: Unknown

Extreme fluctuations?: Unknown

#### Area of occupancy

Trend: Decline (inferred)

Justification for trend: The species occurs in a small patch of native forest, included in a Natural Forest Reserve of Lagoa do Caiado Pico. The AOO is overestimated, being the AOO with native forest around only 1 km². The species continues in decline due to reduced area of occupancy, habitat loss and the expansion of invasive plant species (Borges et al. 2017).

Causes ceased?: No

Causes understood?: Yes

Causes reversible?: Unknown

Extreme fluctuations?: Unknown

AOO (km2): 8

#### Locations

Number of locations: 1

Justification for number of locations: A single fragment of native forest currently with less than 1 km^2^ and with a recent spread of invasive plants, namely *Hedychium gardnerianum*.

Trend: Decline (inferred)

Justification for trend: One location with a size of 1 km² and invasive plants can drive this species to extinction very fast.

Extreme fluctuations?: Unknown

#### Population

Trend: Decline (inferred)

Justification for trend: The species is very rare and only occurs in a small patch of native forest in Pico island. A continuing decline in the number of mature individuals is inferred from monitoring schemes and from the ongoing habitat degradation due to invasions of alien plants (*Hedychium gardnerianum*) (Borges et al. 2017), that are changing the structure of the forest and the cover of bryophytes and ferns in the soil decreasing the quality of the habitat with impacts on the species.

Causes ceased?: No

Causes understood?: Yes

Causes reversible?: Unknown

Extreme fluctuations?: Unknown

#### Subpopulations

Number of subpopulations: 1

Trend: Decline (inferred)

Justification for trend: The species is very rare with a single subpopulation that occurs in a small patch of native forest in Pico island. A continuing decline in the number of subpopulations is inferred from monitoring schemes and from the ongoing habitat degradation due to invasions of alien plants (*Hedychium gardnerianum*) (Borges et al. 2017).

Extreme fluctuations?: Unknown

Severe fragmentation?: No

#### Habitat

System: Terrestrial

Habitat specialist: Yes

Habitat (narrative): The species is very rare, and only occurs in a small patch of native forest dominated by *Juniperus brevifolia* and Ilex perado subsp. azorica in Pico island (Borges et al. 2017). It has an altitudinal range between 700 and 850 m.

Trend in extent, area or quality?: Decline (observed)

Justification for trend: In the past, the species has probably strongly declined due to changes in habitat size and quality (Triantis et al. 2010). Currently the rapid advance of invasive plant species (*Hedychium gardnerianum*) are changing the structure of the forest and the cover of bryophytes and ferns in the soil decreasing the quality of the habitat with impacts on the species (Borges et al. 2017).

##### Habitat

Habitat importance: Major Importance

Habitats: 1.4. Forest - Temperate

#### Habitat

Habitat importance: Major Importance

Habitats: 1.4. Forest - Temperate

#### Ecology

Size: 0.33 cm

Generation length (yr): 1

Dependency of single sp?: Yes

Dependent on species: *Euphorbia stygiana*

Dependent on IUCN Status: Critically Endangered (CR)

Ecology and traits (narrative): This is a nocturnal fungivorous species that lives in dead trunks of endemic trees and also in dead twigs of *Euphorbia stygiana*. This is an univoltine species.

#### Threats

Justification for threats: In the past, the species has probably strongly declined due to changes in habitat size and quality (Triantis et al. 2010). Currently, the rapid advance and expansion of invasive plants species is the major threat (Borges et al. 2017), particularly *Hedychium gardnerianum* since this species is changing the habitat structure, namely decreasing the cover of bryophytes and ferns in the soil and promoting the spread of other plants. Based on Ferreira et al. 2016 the habitat will further decline as a consequence of climate change (increasing number of droughts and habitat shifting and alteration).

##### Threats

Threat type: Ongoing

Threats: 2.2.1. Agriculture & aquaculture - Wood & pulp plantations - Small-holder plantations8.1.2. Invasive and other problematic species, genes & diseases - Invasive non-native/alien species/diseases - Named species

##### Threats

Threat type: Future

Threats: 11.1. Climate change & severe weather - Habitat shifting & alteration11.2. Climate change & severe weather - Droughts

#### Threats

Threat type: Ongoing

Threats: 2.2.1. Agriculture & aquaculture - Wood & pulp plantations - Small-holder plantations8.1.2. Invasive and other problematic species, genes & diseases - Invasive non-native/alien species/diseases - Named species

#### Threats

Threat type: Future

Threats: 11.1. Climate change & severe weather - Habitat shifting & alteration11.2. Climate change & severe weather - Droughts

#### Conservation

Justification for conservation actions: The species is not protected by regional law. Its habitat is in a regionally protected area (Natural Forest Reserve of Lagoa do Caiado in Pico island). Degraded habitats should be restored with the removal of invasive species. A strategy needs also to be developed to address the future threat by climate change. It is necessary a monitoring plan for the invertebrate community in the habitat in order to contribute to the conservation of this species. A habitat management plan is needed and anticipated to be developed during the coming years. Since this species is an icone of the relict native Azorean forests, it is suggested that some awareness measures should be put in practice.

##### Conservation actions

Conservation action type: In Place

Conservation actions: 1. Land/water protection1.1. Land/water protection - Site/area protection2. Land/water management2.2. Land/water management - Invasive/problematic species control

##### Conservation actions

Conservation action type: Needed

Conservation actions: 2.1. Land/water management - Site/area management2.2. Land/water management - Invasive/problematic species control2.3. Land/water management - Habitat & natural process restoration4. Education & awareness5.4.3. Law & policy - Compliance and enforcement - Sub-national level

#### Conservation actions

Conservation action type: In Place

Conservation actions: 1. Land/water protection1.1. Land/water protection - Site/area protection2. Land/water management2.2. Land/water management - Invasive/problematic species control

#### Conservation actions

Conservation action type: Needed

Conservation actions: 2.1. Land/water management - Site/area management2.2. Land/water management - Invasive/problematic species control2.3. Land/water management - Habitat & natural process restoration4. Education & awareness5.4.3. Law & policy - Compliance and enforcement - Sub-national level

#### Other

##### Use and trade

Use type: International

##### Ecosystem services

Ecosystem service type: Less important

Ecosystem services: 7. Nutrient Cycling

##### Research needed

Research needed: 1.2. Research - Population size, distribution & trends1.3. Research - Life history & ecology2.2. Conservation Planning - Area-based Management Plan3.1. Monitoring - Population trends3.4. Monitoring - Habitat trends

Justification for research needed: Further research is needed into its ecology and life history in order to find extant specimens in additional areas of native forest in Pico (e.g. Mistério da Prainha and Caveiro) and obtain information on population size, distribution and trends. It is also necessary an area-based management plan and a monitoring plan for the invertebrate community in the habitat in order to contribute to perform a species potential recovery plan. Monitoring every ten years using the BALA protocol will inform about habitat quality (see e.g. Gaspar et al. 2011).

#### Use and trade

Use type: International

#### Ecosystem services

Ecosystem service type: Less important

Ecosystem services: 7. Nutrient Cycling

#### Research needed

Research needed: 1.2. Research - Population size, distribution & trends1.3. Research - Life history & ecology2.2. Conservation Planning - Area-based Management Plan3.1. Monitoring - Population trends3.4. Monitoring - Habitat trends

Justification for research needed: Further research is needed into its ecology and life history in order to find extant specimens in additional areas of native forest in Pico (e.g. Mistério da Prainha and Caveiro) and obtain information on population size, distribution and trends. It is also necessary an area-based management plan and a monitoring plan for the invertebrate community in the habitat in order to contribute to perform a species potential recovery plan. Monitoring every ten years using the BALA protocol will inform about habitat quality (see e.g. Gaspar et al. 2011).

#### Viability analysis

Justification for probability: 

### Tarphius pomboi

#### Species information

Scientific name: Tarphius pomboi

Species authority: Borges, 1991

Common names: Ironclad beetle (English); Escaravelho-cascudo-da-mata (Portuguese)

Kingdom: Animalia

Phylum: Arthropoda

Class: Insecta

Order: Coleoptera

Family: Zopheridae

Taxonomic notes: This species is characterized by (Borges et al. 2017): anterior margin of clypeus arcuate inwards; humeral angle of elytron very protrude upwards resembling a nodule or a gibbositie; elytral nodules fair developed with a pattern formula 1, 3, 3, 2, 0.

**Figure 31.**
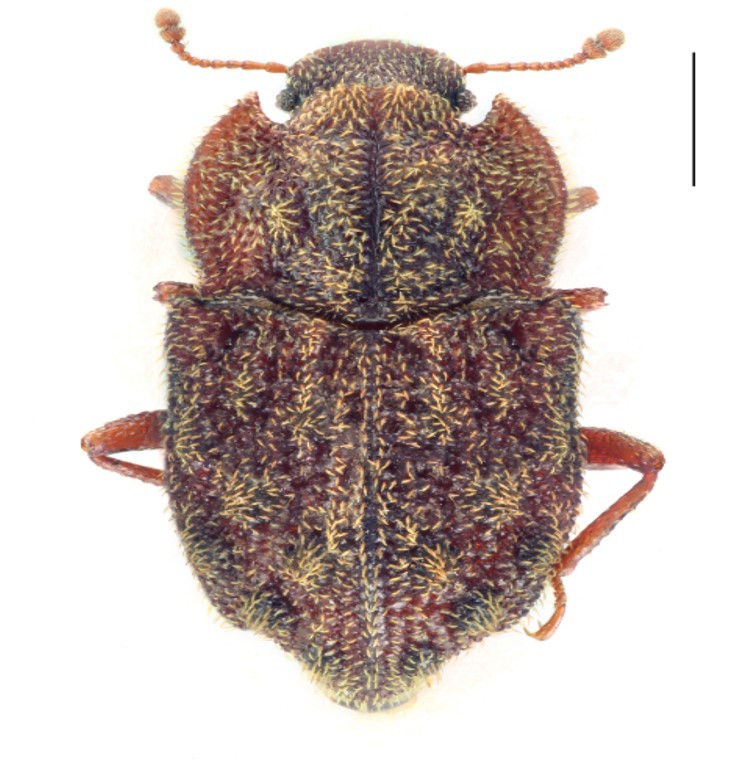
*Tarphius pomboi* from Pico Alto (Santa Maria, Azores) (Credit: Erno-Endre Gergely). Scale 0.5 mm.

Figure(s) or Photo(s): Fig. 31

Region for assessment: Global

#### Editor & Reviewers

##### Reviewers

Reviewers: Anja Danielczak

##### Editor

Editor: Axel Hochkirch

#### Reviewers

Reviewers: Anja Danielczak

#### Editor

Editor: Axel Hochkirch

#### Geographic range

Biogeographic realm: Palearctic

Countries: Portugal

Map of records (Google Earth): Suppl. material 50

Basis of EOO and AOO: Observed

Basis (narrative): The extent of occurrence (EOO) is 20 km^2^ and the maximum area of occupancy (AOO) is 20 km^2^.

Min Elevation/Depth (m): 200

Max Elevation/Depth (m): 500

Range description: *Tarphius pomboi*is a single-island endemic species restricted to Santa Maria island (Azores, Portugal) (Borges et al. 2010, Borges et al. 2017), known from Natural Forest Reserve of Pico Alto (Santa Maria).

#### New occurrences

#### Extent of occurrence

EOO (km2): 20

Trend: Decline (inferred)

Justification for trend: This species occurs in a small fragment of highly modified native vegetation in Santa Maria Island and also in some exotic patches of vegetation. The species continues in decline due to *Cryptomeria japonica* plantations management, habitat loss and the expansion of invasive plant species (Borges et al. 2017).

Causes ceased?: No

Causes understood?: Yes

Causes reversible?: Unknown

Extreme fluctuations?: Unknown

#### Area of occupancy

Trend: Decline (inferred)

Justification for trend: The species occurs in native forests included in Natural Forest Reserves of Santa Maria Island. Possibly the AOO value is overestimated, being the AOO with native forest only around 0.09 km². The species continues in decline due to reduced area of occupancy, *Cryptomeria japonica* plantations management, habitat loss and the expansion of invasive plant species (Borges et al. 2017).

Causes ceased?: No

Causes understood?: Yes

Causes reversible?: Unknown

Extreme fluctuations?: Unknown

AOO (km2): 20

#### Locations

Number of locations: 4

Justification for number of locations: This species occurs in a small fragment of highly modified native vegetation in Santa Maria Island and also in some exotic patches of vegetation. The species is very rare and the Natural Forest Reserve (Pico Alto) has a very low Index of Biotic Integrity (Gaspar et al. 2011).

Trend: Decline (inferred)

Justification for trend: In the last 50 years major alterations were made in the territory with impacts in native habitats. Four locations that were highly impacted by invasive plants in the last ten years. Some of the exotic patches may disappear in near future for the implementation of pastures.

Extreme fluctuations?: Unknown

#### Population

Trend: Decline (inferred)

Justification for trend: This is a rare species that occurs in native forests in Santa Maria island (Borges et al. 2017). A continuing decline in the number of mature individuals is inferred from monitoring schemes and from the ongoing habitat degradation due to invasions of alien plants (namely *Pittosporum undulatum* and *Hedychium gardnerianum*) and the *Cryptomeria japonica* management (Borges et al. 2017). Some of the exotic patches may disappear in near future for the implementation of pastures with further impacts on the population abundance.

Basis for decline: (c) a decline in area of occupancy, extent of occurrence and/or quality of habitat

Causes ceased?: No

Causes understood?: Yes

Causes reversible?: Unknown

Extreme fluctuations?: Unknown

#### Subpopulations

Number of subpopulations: 2

Trend: Decline (inferred)

Justification for trend: This is a rare species that occurs in native forests in Santa Maria island (Borges et al. 2017). A continuing decline in the number of subpopulations is inferred from monitoring schemes and from the ongoing habitat degradation due to invasions of alien plants (namely *Pittosporum undulatum* and *Hedychium gardnerianum*) and the *Cryptomeria japonica* pulp plantation management (Borges et al. 2017). The remaining patch on native vegetation located in a Natural Forest Reserve (Pico Alto) has a very low Index of Biotic Integrity (Gaspar et al. 2011).

Extreme fluctuations?: Unknown

Severe fragmentation?: Yes

Justification for fragmentation: Major land-use changes at all elevations in S. Maria island promoted the creation of small patches of native and exotic forest. The species occurs in one natural forest fragment and small patches of exotic forest that are isolated in a sea of pastures and *Cryptomeria japonica* plantations and that were highly impacted by invasive plants in the last ten years.

#### Habitat

System: Terrestrial

Habitat specialist: Yes

Habitat (narrative): This is a rare species that occurs in the native forests of Santa Maria island dominated by the endemic plants *Morella faya*, *Picconia azorica* and *Erica azorica* but also the invasive *Pittosporum undulatum* (Borges et al. 2017). It has an altitudinal range between 200 and 500 m.

Trend in extent, area or quality?: Decline (observed)

Justification for trend: In the past, the species has probably strongly declined due to changes in habitat size and quality (Triantis et al. 2010). Currently the rapid advance of invasive plant species are decreasing the quality of the habitat (Borges et al. 2017).

##### Habitat

Habitat importance: Major Importance

Habitats: 1.4. Forest - Temperate

#### Habitat

Habitat importance: Major Importance

Habitats: 1.4. Forest - Temperate

#### Ecology

Size: 0.25 cm

Generation length (yr): 1

Dependency of single sp?: No

Ecology and traits (narrative): This is a nocturnal fungivorous species that lives in the soil. This is an univoltine species.

#### Threats

Justification for threats: In the past, the species has probably strongly declined due to changes in habitat size and quality (Triantis et al. 2010). Currently, the rapid advance and expansion of invasive plants species is the major threat (Borges et al. 2010), particularly *Hedychium gardnerianum* since this species is changing the habitat structure, namely decreasing the cover of bryophytes and ferns in the soil and promoting the spread of other plants. *Cryptomeria japonica* wood & pulp plantations management can also be a problem. Based on Ferreira et al. 2016 the habitat will further decline as a consequence of climate change (increasing number of droughts and habitat shifting and alteration). An additional future threat will be the transformation of some of the exotic patches in pastures with further impacts on the population abundance.

##### Threats

Threat type: Ongoing

Threats: 2.2.1. Agriculture & aquaculture - Wood & pulp plantations - Small-holder plantations8.1.2. Invasive and other problematic species, genes & diseases - Invasive non-native/alien species/diseases - Named species

##### Threats

Threat type: Future

Threats: 2.3. Agriculture & aquaculture - Livestock farming & ranching11.1. Climate change & severe weather - Habitat shifting & alteration11.2. Climate change & severe weather - Droughts

#### Threats

Threat type: Ongoing

Threats: 2.2.1. Agriculture & aquaculture - Wood & pulp plantations - Small-holder plantations8.1.2. Invasive and other problematic species, genes & diseases - Invasive non-native/alien species/diseases - Named species

#### Threats

Threat type: Future

Threats: 2.3. Agriculture & aquaculture - Livestock farming & ranching11.1. Climate change & severe weather - Habitat shifting & alteration11.2. Climate change & severe weather - Droughts

#### Conservation

Justification for conservation actions: The species is protected by regional law (RAA 2012). Its habitat is in a regionally protected area (Natural Forest Reserve of Pico Alto in Sta Maria island). Degraded habitats should be restored with the removal of invasive species. A strategy needs also to be developed to address the future threat by climate change. It is necessary a monitoring plan for the invertebrate community in the habitat in order to contribute to the conservation of this species. A habitat management plan is needed and anticipated to be developed during the coming years. Since this species is an icone of the relict native Azorean forests, it is suggested that some awareness measures should be put in practice.

##### Conservation actions

Conservation action type: In Place

Conservation actions: 1. Land/water protection1.1. Land/water protection - Site/area protection5. Law & policy5.1. Law & policy - Legislation5.2. Law & policy - Policies and regulations

##### Conservation actions

Conservation action type: Needed

Conservation actions: 2.1. Land/water management - Site/area management2.2. Land/water management - Invasive/problematic species control2.3. Land/water management - Habitat & natural process restoration4. Education & awareness5.4.3. Law & policy - Compliance and enforcement - Sub-national level

#### Conservation actions

Conservation action type: In Place

Conservation actions: 1. Land/water protection1.1. Land/water protection - Site/area protection5. Law & policy5.1. Law & policy - Legislation5.2. Law & policy - Policies and regulations

#### Conservation actions

Conservation action type: Needed

Conservation actions: 2.1. Land/water management - Site/area management2.2. Land/water management - Invasive/problematic species control2.3. Land/water management - Habitat & natural process restoration4. Education & awareness5.4.3. Law & policy - Compliance and enforcement - Sub-national level

#### Other

##### Use and trade

Use type: International

##### Ecosystem services

Ecosystem service type: Less important

Ecosystem services: 7. Nutrient Cycling

##### Research needed

Research needed: 1.2. Research - Population size, distribution & trends1.3. Research - Life history & ecology2.2. Conservation Planning - Area-based Management Plan3.1. Monitoring - Population trends3.4. Monitoring - Habitat trends

Justification for research needed: Further research is needed into its ecology and life history in order to find additional extant specimens in the Pico Alto surrounded areas of exotic forest and obtain information on population size, distribution and trends. It is also necessary an area-based management pland and a monitoring plan for the invertebrate community in the habitat in order to contribute to perform a species potential recovery plan. Monitoring every ten years using the BALA protocol will inform about habitat quality (see e.g. Gaspar et al. 2011).

#### Use and trade

Use type: International

#### Ecosystem services

Ecosystem service type: Less important

Ecosystem services: 7. Nutrient Cycling

#### Research needed

Research needed: 1.2. Research - Population size, distribution & trends1.3. Research - Life history & ecology2.2. Conservation Planning - Area-based Management Plan3.1. Monitoring - Population trends3.4. Monitoring - Habitat trends

Justification for research needed: Further research is needed into its ecology and life history in order to find additional extant specimens in the Pico Alto surrounded areas of exotic forest and obtain information on population size, distribution and trends. It is also necessary an area-based management pland and a monitoring plan for the invertebrate community in the habitat in order to contribute to perform a species potential recovery plan. Monitoring every ten years using the BALA protocol will inform about habitat quality (see e.g. Gaspar et al. 2011).

#### Viability analysis

Justification for probability: 

### Tarphius relictus

#### Species information

Scientific name: Tarphius relictus

Species authority: Borges & Serrano, 2017

Common names: Ironclad beetle (English); Escaravelho-cascudo-da-mata (Portuguese)

Kingdom: Animalia

Phylum: Arthropoda

Class: Insecta

Order: Coleoptera

Family: Zopheridae

Taxonomic notes: The species belongs to the “*tornvalli*” complex and is characterized by (Borges et al. 2017): lateral margins of pronotum slightly arcuate and sub-parallel in the posterior half; setae less needle shaped; elytral nodules well developed with a pattern formula 2, 3, 2, 1.

**Figure 32.**
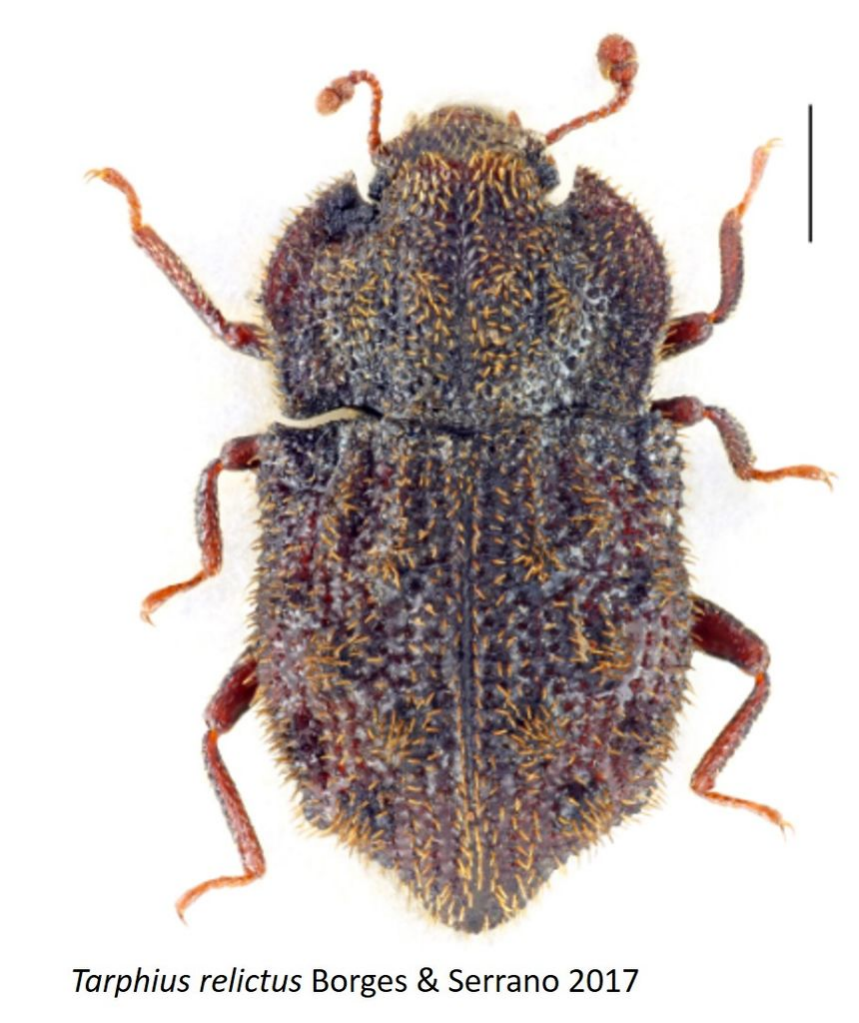
*Tarphius relictus* from Biscoito das Fontinhas (Terceira, Azores) (Credit: Erno-Endre Gergely). Scale 0.5 mm.

Figure(s) or Photo(s): Fig. 32

Region for assessment: Global

#### Editor & Reviewers

##### Reviewers

Reviewers: Anja Danielczak

##### Editor

Editor: Axel Hochkirch

#### Reviewers

Reviewers: Anja Danielczak

#### Editor

Editor: Axel Hochkirch

#### Geographic range

Biogeographic realm: Palearctic

Countries: Portugal

Map of records (Google Earth): Suppl. material 51

Basis of EOO and AOO: Observed

Basis (narrative): The extent of occurrence (EOO) is 8 km^2^ and the maximum area of occupancy (AOO) is 8 km^2^.

Min Elevation/Depth (m): 200

Max Elevation/Depth (m): 300

Range description: *Tarphius relictus* is a single-island endemic species restricted to Terceira island (Azores, Portugal) (Borges et al. 2017), known in only one locality (Biscito das Fontinhas), being a very rare species.

#### New occurrences

#### Extent of occurrence

EOO (km2): 8

Trend: Decline (inferred)

Justification for trend: This species occurs in a very small patch of exotic forest (dominated by *Acacia* spp.), located in Fontinhas (Terceira island). This is a very rare species with a reduced extent of occurrence. The species continues in decline due to habitat loss and invasive plant species, being the most endangered *Tarphius* species in the Azores (Borges et al. 2017).

Causes ceased?: No

Causes understood?: Yes

Causes reversible?: Unknown

Extreme fluctuations?: Unknown

#### Area of occupancy

Trend: Decline (inferred)

Justification for trend: The species occurs in a small, disturbed site covered by exotic trees in Terceira island. The AOO is clearly overestimated, being the AOO with exotic forest around only 0.09 km². The species continues in decline due to reduced area of occupancy and the existence of invasive plant species, being the most endangered *Tarphius* species in the Azores (Borges et al. 2017).

Causes ceased?: No

Causes understood?: Yes

Causes reversible?: Unknown

Extreme fluctuations?: Unknown

AOO (km2): 8

#### Locations

Number of locations: 1

Justification for number of locations: The species occurs in one single patch of exotic forest in Terceira island very disturbed.

Trend: Decline (inferred)

Justification for trend: One location with a area of 0.09 km² and invasive plants can drive this species to extinction very fast.

Extreme fluctuations?: Unknown

#### Population

Trend: Decline (inferred)

Justification for trend: The species is very rare and only occurs in a small patch of exotic forest (*Acacia* spp.) in Terceira island. This is the most endangered *Tarphius* species in the Azores, due to restricted area of distribution and the existence of invasive plant species (Borges et al. 2017).

Basis for decline: (c) a decline in area of occupancy, extent of occurrence and/or quality of habitat

Causes ceased?: No

Causes understood?: Yes

Causes reversible?: Unknown

Extreme fluctuations?: Unknown

#### Subpopulations

Number of subpopulations: 1

Trend: Decline (inferred)

Justification for trend: The species is known from a single subpopulation and is very rare only occuring in a small patch of exotic forest (*Acacia* spp.) in Terceira island. This is the most endangered *Tarphius* species in the Azores. The subpopulation may disappear if dramatich changes occur, namely the spread of invasive plants changing the structure of the current habitat

Extreme fluctuations?: Unknown

Severe fragmentation?: No

#### Habitat

System: Terrestrial

Habitat specialist: Yes

Habitat (narrative): The species is very rare, and only occurs in a small, disturbed site covered by exotic trees (dominated by *Acacia* spp.) at low altitude, in Terceira island (Borges et al. 2017). It has an altitudinal range between 200 and 300 m.

Trend in extent, area or quality?: Decline (observed)

Justification for trend: In the past, the species has probably strongly declined due to changes in habitat size and quality (Triantis et al. 2010). Currently the rapid advance of invasive plant species are decreasing the quality of the habitat (Borges et al. 2017).

##### Habitat

Habitat importance: Major Importance

Habitats: 16. Introduced vegetation

#### Habitat

Habitat importance: Major Importance

Habitats: 16. Introduced vegetation

#### Ecology

Size: 0.31 cm

Generation length (yr): 1

Dependency of single sp?: No

Ecology and traits (narrative): This is a nocturnal fungivorous species that lives in the soil and in dead wood. This is an univoltine species.

#### Threats

Justification for threats: In the past, the species has probably strongly declined due to changes in habitat size and quality (Triantis et al. 2010). Currently, the rapid advance and expansion of invasive plants species is the major threat (Borges et al. 2017), particularly *Hedychium gardnerianum* and *Pittosporum undulatum* since are changing the habitat structure, namely decreasing the cover of bryophytes and ferns in the soil and promoting the spread of other plants. The management of the *Acacia* spp. patches could be also a problem for the unique surviving subpopulation. Based on Ferreira et al. 2016 the habitat will further decline as a consequence of climate change (increasing number of droughts and habitat shifting and alteration).

##### Threats

Threat type: Ongoing

Threats: 2.1.2. Agriculture & aquaculture - Annual & perennial non-timber crops - Small-holder farming8.1.2. Invasive and other problematic species, genes & diseases - Invasive non-native/alien species/diseases - Named species

##### Threats

Threat type: Future

Threats: 11.1. Climate change & severe weather - Habitat shifting & alteration11.2. Climate change & severe weather - Droughts

#### Threats

Threat type: Ongoing

Threats: 2.1.2. Agriculture & aquaculture - Annual & perennial non-timber crops - Small-holder farming8.1.2. Invasive and other problematic species, genes & diseases - Invasive non-native/alien species/diseases - Named species

#### Threats

Threat type: Future

Threats: 11.1. Climate change & severe weather - Habitat shifting & alteration11.2. Climate change & severe weather - Droughts

#### Conservation

**Figure 33.**
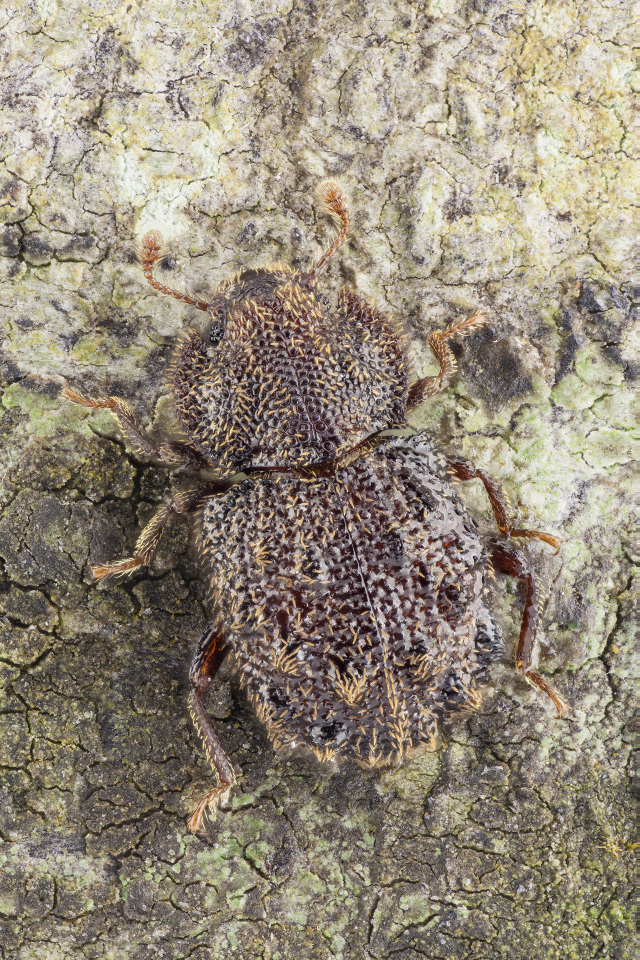
Extreme macro image of *Tarphius relictus* from Biscoito das Fontinhas (Terceira, Azores) (Credit: Javier Torrent).

Justification for conservation actions: The species is not protected by regional law. Its habitat is now included in the Natural Park of Terceira (IUCN Type V level of protection). Degraded habitats should be restored and a strategy needs to be developed to address the future threat by climate change. It is necessary a monitoring plan for the invertebrate community in the habitat in order to contribute to the conservation of this species. We suggest as a possible additional measure of conservation the translocation of individuals for the pristine patches of forest in the high altitude sites of Terceira Island (i.e. ex.situ conservation). Since this species is an icone of the relict native Azorean forests, it is suggested that some awareness measures should be put in practice using for instance images from extreme macro (e.g. Fig. 33)

##### Conservation actions

Conservation action type: In Place

Conservation actions: 1. Land/water protection1.1. Land/water protection - Site/area protection

##### Conservation actions

Conservation action type: Needed

Conservation actions: 2.1. Land/water management - Site/area management2.2. Land/water management - Invasive/problematic species control2.3. Land/water management - Habitat & natural process restoration4. Education & awareness5.4.3. Law & policy - Compliance and enforcement - Sub-national level

#### Conservation actions

Conservation action type: In Place

Conservation actions: 1. Land/water protection1.1. Land/water protection - Site/area protection

#### Conservation actions

Conservation action type: Needed

Conservation actions: 2.1. Land/water management - Site/area management2.2. Land/water management - Invasive/problematic species control2.3. Land/water management - Habitat & natural process restoration4. Education & awareness5.4.3. Law & policy - Compliance and enforcement - Sub-national level

#### Other

##### Use and trade

Use type: International

##### Ecosystem services

Ecosystem service type: Less important

Ecosystem services: 7. Nutrient Cycling

##### Research needed

Research needed: 1.2. Research - Population size, distribution & trends1.3. Research - Life history & ecology2.2. Conservation Planning - Area-based Management Plan3.1. Monitoring - Population trends3.4. Monitoring - Habitat trends

Justification for research needed: Further research is needed into its ecology and life history in order to find additional extant specimens in some meddle elevation forest patches in Terceira island and obtain information on population size, distribution and trends. It is also necessary an area-based management plan and a monitoring plan for the invertebrate community in the habitat in order to contribute to perform a species potential recovery plan.

#### Use and trade

Use type: International

#### Ecosystem services

Ecosystem service type: Less important

Ecosystem services: 7. Nutrient Cycling

#### Research needed

Research needed: 1.2. Research - Population size, distribution & trends1.3. Research - Life history & ecology2.2. Conservation Planning - Area-based Management Plan3.1. Monitoring - Population trends3.4. Monitoring - Habitat trends

Justification for research needed: Further research is needed into its ecology and life history in order to find additional extant specimens in some meddle elevation forest patches in Terceira island and obtain information on population size, distribution and trends. It is also necessary an area-based management plan and a monitoring plan for the invertebrate community in the habitat in order to contribute to perform a species potential recovery plan.

#### Viability analysis

Justification for probability: 

### Tarphius rufonodulosus

#### Species information

Scientific name: Tarphius rufonodulosus

Species authority: Israelson, 1984

Common names: Ironclad beetle (English); Escaravelho-cascudo-da-mata (Portuguese)

Kingdom: Animalia

Phylum: Arthropoda

Class: Insecta

Order: Coleoptera

Family: Zopheridae

Taxonomic notes: This species is characterized by (Borges et al. 2017): body surface sparingly covered with setae; external row of lateral margin of pronotum with about 30 short and leaf shaped setae; elytrae with pale pattern enclosing nodules; elytral nodules developed with a pattern formula 1, 2/3, 2, 2, 1.

**Figure 34.**
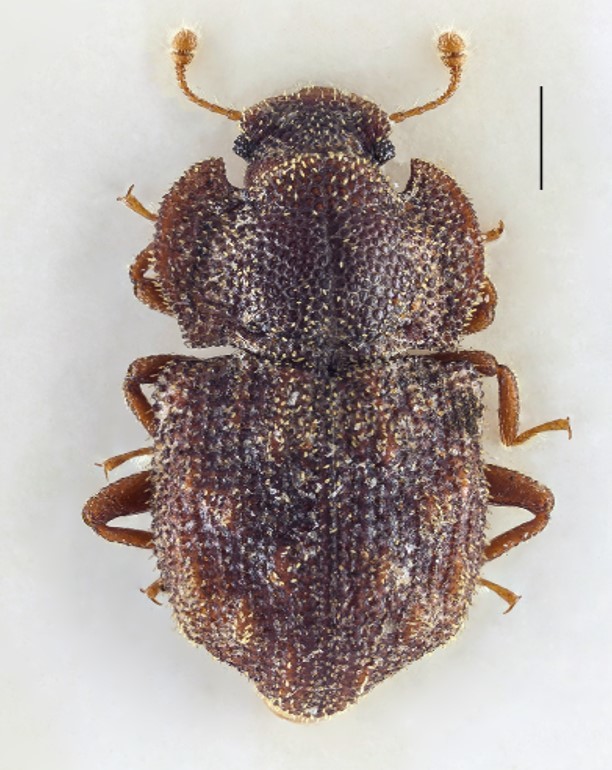
*Tarphius rufonodulosus* from Pico Alto (Santa Maria, Azores) (Credit: Erno-Endre Gergely). Scale 0.5 mm.

Figure(s) or Photo(s): Fig. 34

Region for assessment: Global

#### Editor & Reviewers

##### Reviewers

Reviewers: Anja Danielczak

##### Editor

Editor: Axel Hochkirch

#### Reviewers

Reviewers: Anja Danielczak

#### Editor

Editor: Axel Hochkirch

#### Geographic range

Biogeographic realm: Palearctic

Countries: Portugal

Map of records (Google Earth): Suppl. material 52

Basis of EOO and AOO: Observed

Basis (narrative): The extent of occurrence (EOO) is 28 km^2^ and the maximum area of occupancy (AOO) is 28 km^2^.

Min Elevation/Depth (m): 200

Max Elevation/Depth (m): 500

Range description: *Tarphius rufonodulosus* is a single-island endemic species restricted to Santa Maria island (Azores, Portugal) (Borges et al. 2010, Borges et al. 2017), known from Natural Forest Reserve of Pico Alto (Santa Maria).

#### New occurrences

#### Extent of occurrence

EOO (km2): 28

Trend: Decline (inferred)

Justification for trend: This species is particularly widespread, occurring in a small native patch of forest and in several exotic forests. The species continues in decline due to native forest destruction, *Cryptomeria japonica* pulp plantations management and due to habitat degradation by the rapid advance of invasive plant species that are changing the habitat (Borges et al. 2017).

Causes ceased?: No

Causes understood?: Yes

Causes reversible?: Unknown

Extreme fluctuations?: Unknown

#### Area of occupancy

Trend: Decline (inferred)

Justification for trend: The species occurs in native and exotic forests, included in a Natural Forest Reserve of S. Maria (Borges et al. 2017). The AOO is sligthy overestimated, being the AOO with native forest around 0.09 km². The species continues in decline due to native forest destruction, *Cryptomeria japonica* pulp plantations management and due to habitat degradation by the rapid advance of invasive plant speciesthat are changing the habitat (Borges et al. 2017).

Causes ceased?: No

Causes understood?: Yes

Causes reversible?: Unknown

Extreme fluctuations?: Unknown

AOO (km2): 28

#### Locations

Number of locations: 4

Justification for number of locations: The species occurs in several native forest patches included in a Natural Forest Reserve and in exotic forest patches, in the Santa Maria island.

Trend: Decline (inferred)

Justification for trend: In the last 50 years major alterations were made in the territory with impacts in native habitats. Four locations that were highly impacted by invasive plants in the last ten years and some of them may disappear as a consequence of transformation of patches of exotic forest into pastureland.

Extreme fluctuations?: Unknown

#### Population

Trend: Decline (inferred)

Justification for trend: The species is abundant, particularly in the canopy of several endemic trees in a patch of native forest of Santa Maria Island. A continuing decline in the number of mature individuals is inferred from monitoring schemes and from the ongoing habitat degradation due to invasions of alien plants (namely *Pittosporum undulatum*) and the *Cryptomeria japonica* pulp plantations management (Borges et al. 2017). Some of the patches with exotic plants may disappear in near future for the implementation of pastures with further impacts on the population abundance.

Causes ceased?: No

Causes understood?: Yes

Causes reversible?: Unknown

Extreme fluctuations?: Unknown

#### Subpopulations

Number of subpopulations: 4

Trend: Decline (inferred)

Justification for trend: The species is abundant, particularly in the canopy of several endemic trees in a patch of native forest of S. Maria Island. A continuing decline in the number of subpopulations is inferred from monitoring schemes and from the ongoing habitat degradation due to invasions of alien plants (namely *Pittosporum undulatum*) and the *Cryptomeria japonica* pulp plantations management (Borges et al. 2017). Some of the subpopulations occurring in exotic forest may disappear in near future for the implementation of pastures.

Extreme fluctuations?: Unknown

Severe fragmentation?: Yes

Justification for fragmentation: Major land-use changes at all elevations in S. Maria island promoted the creation of small patches of native and exotic forest. The species occurs in one natural forest fragment and small patches of exotic forest that are isolated in a sea of pastures and *Cryptomeria japonica* plantations that were highly impacted by invasive plants in the last ten years.

#### Habitat

System: Terrestrial

Habitat specialist: Yes

Habitat (narrative): The species is particularly abundant in the canopies of native trees (e.g. *Picconia azorica*) and under-bark of dead trees both in native and exotic forests (dominated by *Acacia* spp. and *Cryptomeria japonica*). This species has an altitudinal range between 200 and 500 m.

Trend in extent, area or quality?: Decline (observed)

Justification for trend: In the past, the species has probably strongly declined due to changes in habitat size and quality (Triantis et al. 2010). Currently the rapid advance of invasive plant species are decreasing the quality of the habitat (Borges et al. 2017).

##### Habitat

Habitat importance: Major Importance

Habitats: 1.4. Forest - Temperate14.3. Artificial/Terrestrial - Plantations16. Introduced vegetation

#### Habitat

Habitat importance: Major Importance

Habitats: 1.4. Forest - Temperate14.3. Artificial/Terrestrial - Plantations16. Introduced vegetation

#### Ecology

Size: 0.23 cm

Generation length (yr): 1

Dependency of single sp?: No

Ecology and traits (narrative): This is a nocturnal fungivorous species. This is an univoltine species.

#### Threats

Justification for threats: In the past, the species has probably strongly declined due to changes in habitat size and quality (Triantis et al. 2010). Currently, the rapid advance and expansion of invasive plants species is the major threat (Borges et al. 2017), particularly *Pittosporum undulatum* and *Hedychium gardnerianum* that ar changing the habitat structure, namely decreasing the cover of bryophytes and ferns in the soil and promoting the spread of other plants. The management of *Cryptomeria plantations* could be also a problem for the subpopulations living in this habitat. Based on Ferreira et al. 2016 the habitat will further decline as a consequence of climate change (increasing number of droughts and habitat shifting and alteration). An additional future threat will be the transformation of some of the exotic patches in pastures with further impacts on the population abundance.

##### Threats

Threat type: Ongoing

Threats: 2.2.1. Agriculture & aquaculture - Wood & pulp plantations - Small-holder plantations8.1.2. Invasive and other problematic species, genes & diseases - Invasive non-native/alien species/diseases - Named species

##### Threats

Threat type: Future

Threats: 2.3. Agriculture & aquaculture - Livestock farming & ranching11.1. Climate change & severe weather - Habitat shifting & alteration11.2. Climate change & severe weather - Droughts

#### Threats

Threat type: Ongoing

Threats: 2.2.1. Agriculture & aquaculture - Wood & pulp plantations - Small-holder plantations8.1.2. Invasive and other problematic species, genes & diseases - Invasive non-native/alien species/diseases - Named species

#### Threats

Threat type: Future

Threats: 2.3. Agriculture & aquaculture - Livestock farming & ranching11.1. Climate change & severe weather - Habitat shifting & alteration11.2. Climate change & severe weather - Droughts

#### Conservation

Justification for conservation actions: The species is not protected by regional law. Its habitat is in a regionally protected area (Natural Forest Reserve of Pico Alto in Sta Maria island). Degraded habitats should be restored with the removal of invasive species. A strategy needs also to be developed to address the future threat by climate change. It is necessary a monitoring plan for the invertebrate community in the habitat in order to contribute to the conservation of this species. A habitat management plan is needed and anticipated to be developed during the coming years. Since this species is an icone of the relict native Azorean forests, it is suggested that some awareness measures should be put in practice.

##### Conservation actions

Conservation action type: In Place

Conservation actions: 1. Land/water protection1.1. Land/water protection - Site/area protection

##### Conservation actions

Conservation action type: Needed

Conservation actions: 2.1. Land/water management - Site/area management2.2. Land/water management - Invasive/problematic species control2.3. Land/water management - Habitat & natural process restoration4. Education & awareness5.4.3. Law & policy - Compliance and enforcement - Sub-national level

#### Conservation actions

Conservation action type: In Place

Conservation actions: 1. Land/water protection1.1. Land/water protection - Site/area protection

#### Conservation actions

Conservation action type: Needed

Conservation actions: 2.1. Land/water management - Site/area management2.2. Land/water management - Invasive/problematic species control2.3. Land/water management - Habitat & natural process restoration4. Education & awareness5.4.3. Law & policy - Compliance and enforcement - Sub-national level

#### Other

##### Use and trade

Use type: International

##### Ecosystem services

Ecosystem service type: Less important

Ecosystem services: 7. Nutrient Cycling

##### Research needed

Research needed: 1.2. Research - Population size, distribution & trends1.3. Research - Life history & ecology3.1. Monitoring - Population trends3.4. Monitoring - Habitat trends

Justification for research needed: Further research is needed into its ecology and life history in order to find additional extant specimens in more forest areas in S. Maria and obtain information on population size, distribution and trends. It is also necessary a monitoring plan for the invertebrate community in the habitat in order to contribute to perform a species potential recovery plan. Monitoring every ten years using the BALA protocol will inform about habitat quality (see e.g. Gaspar et al. 2011).

#### Use and trade

Use type: International

#### Ecosystem services

Ecosystem service type: Less important

Ecosystem services: 7. Nutrient Cycling

#### Research needed

Research needed: 1.2. Research - Population size, distribution & trends1.3. Research - Life history & ecology3.1. Monitoring - Population trends3.4. Monitoring - Habitat trends

Justification for research needed: Further research is needed into its ecology and life history in order to find additional extant specimens in more forest areas in S. Maria and obtain information on population size, distribution and trends. It is also necessary a monitoring plan for the invertebrate community in the habitat in order to contribute to perform a species potential recovery plan. Monitoring every ten years using the BALA protocol will inform about habitat quality (see e.g. Gaspar et al. 2011).

#### Viability analysis

Justification for probability: 

### Tarphius serranoi

#### Species information

Scientific name: Tarphius serranoi

Species authority: Borges, 1991

Common names: Ironclad beetle (English); Escaravelho-cascudo-da-mata (Portuguese)

Kingdom: Animalia

Phylum: Arthropoda

Class: Insecta

Order: Coleoptera

Family: Zopheridae

Taxonomic notes: This species is characterized by (Borges et al. 2017): body surface covered with more dense setae; external row of lateral margin of pronotum with 15-16 almost leaf shaped setae; elytral nodules evanescent with a pattern formula 3/2, 3, 2, 0/1.

**Figure 35.**
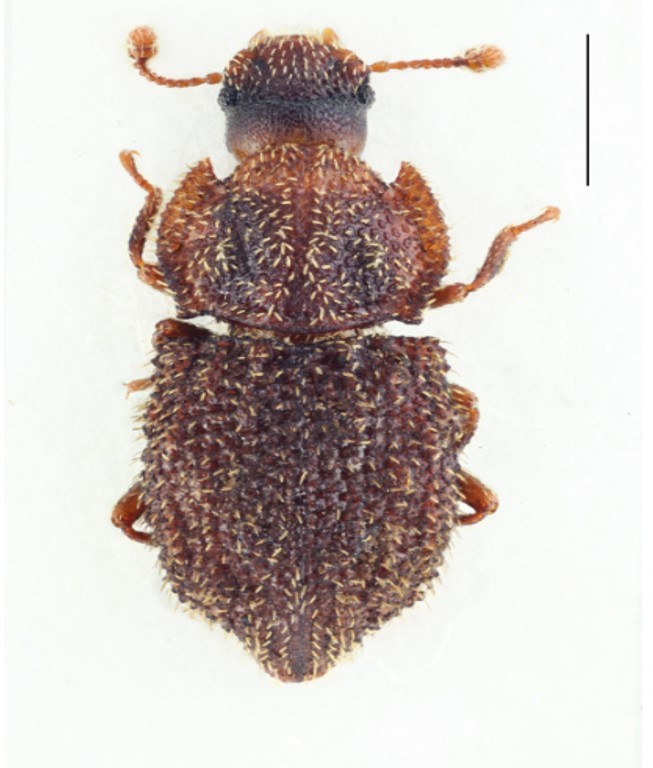
*Tarphius serranoi* from Pico Alto (Santa Maria, Azores) (Credit: Erno-Endre Gergely). Scale 0.5 mm.

Figure(s) or Photo(s): Fig. 35

Region for assessment: Global

#### Editor & Reviewers

##### Reviewers

Reviewers: Anja Danielczak

##### Editor

Editor: Axel Hochkirch

#### Reviewers

Reviewers: Anja Danielczak

#### Editor

Editor: Axel Hochkirch

#### Geographic range

Biogeographic realm: Palearctic

Countries: Portugal

Map of records (Google Earth): Suppl. material 53

Basis of EOO and AOO: Observed

Basis (narrative): The extent of occurrence (EOO) is 8 km^2^ and the maximum area of occupancy (AOO) is 8 km^2^.

Min Elevation/Depth (m): 400

Max Elevation/Depth (m): 50

Range description: *Tarphius serranoi* is a single-island endemic species restricted to Santa Maria island (Azores, Portugal) (Borges et al. 2010, Borges et al. 2017), known from Natural Forest Reserve of Pico Alto (S. Maria).

#### New occurrences

#### Extent of occurrence

EOO (km2): 8

Trend: Decline (inferred)

Justification for trend: This species occurs in a small patch of native forest (dominated by *Erica azorica* and *Picconia azorica*) included in a Natural forest Reserve of Santa Maria island. This is a very rare species with a reduced extent of occurrence. The species continues in decline due to habitat loss and the expansion of invasive plant species (Borges et al. 2017).

Causes ceased?: No

Causes understood?: Yes

Causes reversible?: Unknown

Extreme fluctuations?: Unknown

#### Area of occupancy

Trend: Decline (inferred)

Justification for trend: The species occurs in a small patch of native forest, included in a Natural Forest Reserve of Santa Maria. The AOO is overestimated, since the area of its remaining native habitat is now only around 0.09 km². The species continues in decline due to reduced area of occupancy, habitat loss and the expansion of invasive plant species (Borges et al. 2017).

Causes ceased?: No

Causes understood?: Yes

Causes reversible?: Unknown

Extreme fluctuations?: Unknown

AOO (km2): 8

#### Locations

Number of locations: 1

Justification for number of locations: The species occurs in a single native forest patch included in the Natural Forest Reserve of Pico Alto, that has a very low Index of Biotic Integrity (Gaspar et al. 2011).

Trend: Decline (inferred)

Justification for trend: One location that has a very low Index of Biotic Integrity (Gaspar et al. 2011) with a size of 0.09 km² and invasive plants can drive this species to extinction very fast.

Extreme fluctuations?: Unknown

#### Population

Trend: Decline (inferred)

Justification for trend: The species is very rare and only occurs in a small patch of native forest in Santa Maria island. A continuing decline in the number of mature individuals is inferred from monitoring protocols and from the ongoing habitat degradation due to invasions of alien plants (namely *Pittosporum undulatum* and *Hedychium gardnerianum*) and the *Cryptomeria japonica* management (Borges et al. 2017).

Basis for decline: (c) a decline in area of occupancy, extent of occurrence and/or quality of habitat

Causes ceased?: No

Causes understood?: Yes

Causes reversible?: Unknown

Extreme fluctuations?: Unknown

#### Subpopulations

Number of subpopulations: 1

Trend: Decline (inferred)

Justification for trend: The species is very rare and only occurs in a small patch of native forest in Santa Maria island. A continuing decline in the habitat is inferred from monitoring schemes and from the ongoing habitat degradation due to invasions of alien plants (namely *Pittosporum undulatum* and *Hedychium gardnerianum*) and the *Cryptomeria japonica* management (Borges et al. 2017). The remaining patch on native vegetation located in a Natural Forest Reserve (Pico Alto) has a very low Index of Biotic Integrity (Gaspar et al. 2011). As a consequence the subpopulation may become extinct in short to medium period.

Extreme fluctuations?: Unknown

Severe fragmentation?: No

#### Habitat

System: Terrestrial

Habitat specialist: Yes

Habitat (narrative): The species is very rare, and only occurs in a small patch of native forest, dominated by the native plantas *Erica azorica, Morella faya* and *Picconia azorica* and the invasive *Pittosporum undulatum* in Santa Maria island (Borges et al. 2017). It has an altitudinal range between 400 and 500 m.

Trend in extent, area or quality?: Decline (observed)

Justification for trend: In the past, the species has probably strongly declined due to changes in habitat size and quality (Triantis et al. 2010). Currently the rapid advance of invasive plant species are decreasing the quality of the habitat (Borges et al. 2017).

##### Habitat

Habitat importance: Major Importance

Habitats: 1.4. Forest - Temperate

#### Habitat

Habitat importance: Major Importance

Habitats: 1.4. Forest - Temperate

#### Ecology

Size: 0.23 cm

Generation length (yr): 1

Dependency of single sp?: No

Ecology and traits (narrative): This is a nocturnal fungivorous species that lives associated with lichens in tree canopies, but also in the soil. This is an univoltine species.

#### Threats

Justification for threats: In the past, the species has probably strongly declined due to changes in habitat size and quality (Triantis et al. 2010). Currently, the rapid advance and expansion of invasive plants species is the major threat (Borges et al. 2017), particularly *Pittosporum undulatum* and *Hedychium gardnerianum* that are changing the habitat structure, namely decreasing the cover of bryophytes and ferns in the soil and promoting the spread of other plants. Based on Ferreira et al. 2016 the habitat will further decline as a consequence of climate change (increasing number of droughts and habitat shifting and alteration).

##### Threats

Threat type: Ongoing

Threats: 2.2.1. Agriculture & aquaculture - Wood & pulp plantations - Small-holder plantations8.1.2. Invasive and other problematic species, genes & diseases - Invasive non-native/alien species/diseases - Named species

##### Threats

Threat type: Future

Threats: 11.1. Climate change & severe weather - Habitat shifting & alteration11.2. Climate change & severe weather - Droughts

#### Threats

Threat type: Ongoing

Threats: 2.2.1. Agriculture & aquaculture - Wood & pulp plantations - Small-holder plantations8.1.2. Invasive and other problematic species, genes & diseases - Invasive non-native/alien species/diseases - Named species

#### Threats

Threat type: Future

Threats: 11.1. Climate change & severe weather - Habitat shifting & alteration11.2. Climate change & severe weather - Droughts

#### Conservation

Justification for conservation actions: The species is protected by regional law (RAA 2012). Its habitat is in a regionally protected area (Natural Forest Reserve of Pico Alto in Sta Maria island). Degraded habitats should be restored with the removal of invasive species. A strategy needs also to be developed to address the future threat by climate change. It is necessary a monitoring plan for the invertebrate community in the habitat in order to contribute to the conservation of this species. A habitat management plan is needed and anticipated to be developed during the coming years. Since this species is an icone of the relict native Azorean forests, it is suggested that some awareness measures should be put in practice.

##### Conservation actions

Conservation action type: In Place

Conservation actions: 1. Land/water protection

##### Conservation actions

Conservation action type: Needed

Conservation actions: 2.1. Land/water management - Site/area management2.2. Land/water management - Invasive/problematic species control2.3. Land/water management - Habitat & natural process restoration4. Education & awareness5.4.3. Law & policy - Compliance and enforcement - Sub-national level

#### Conservation actions

Conservation action type: In Place

Conservation actions: 1. Land/water protection

#### Conservation actions

Conservation action type: Needed

Conservation actions: 2.1. Land/water management - Site/area management2.2. Land/water management - Invasive/problematic species control2.3. Land/water management - Habitat & natural process restoration4. Education & awareness5.4.3. Law & policy - Compliance and enforcement - Sub-national level

#### Other

##### Use and trade

Use type: International

##### Ecosystem services

Ecosystem service type: Less important

Ecosystem services: 7. Nutrient Cycling

##### Research needed

Research needed: 1.2. Research - Population size, distribution & trends1.3. Research - Life history & ecology2.2. Conservation Planning - Area-based Management Plan3.1. Monitoring - Population trends3.4. Monitoring - Habitat trends

Justification for research needed: Further research is needed into its ecology and life history in order to find additional extant specimens in areas od exotic plantations around Pico Alto (S. Maria) and obtain information on population size, distribution and trends. It is also necessary an area-based management plan and a monitoring plan for the invertebrate community in the habitat in order to contribute to perform a species potential recovery plan. Monitoring every ten years using the BALA protocol will inform about habitat quality (see e.g. Gaspar et al. 2011).

#### Use and trade

Use type: International

#### Ecosystem services

Ecosystem service type: Less important

Ecosystem services: 7. Nutrient Cycling

#### Research needed

Research needed: 1.2. Research - Population size, distribution & trends1.3. Research - Life history & ecology2.2. Conservation Planning - Area-based Management Plan3.1. Monitoring - Population trends3.4. Monitoring - Habitat trends

Justification for research needed: Further research is needed into its ecology and life history in order to find additional extant specimens in areas od exotic plantations around Pico Alto (S. Maria) and obtain information on population size, distribution and trends. It is also necessary an area-based management plan and a monitoring plan for the invertebrate community in the habitat in order to contribute to perform a species potential recovery plan. Monitoring every ten years using the BALA protocol will inform about habitat quality (see e.g. Gaspar et al. 2011).

#### Viability analysis

Justification for probability: 

### Tarphius tornvalli

#### Species information

Scientific name: Tarphius tornvalli

Species authority: Gillerfors, 1986

Common names: Ironclad beetle (English); Escaravelho-cascudo-da-mata (Portuguese)

Kingdom: Animalia

Phylum: Arthropoda

Class: Insecta

Order: Coleoptera

Family: Zopheridae

Taxonomic notes: The species belongs to the “*tornvalli*” complex and is characterized by (Borges et al. 2017): lateral margins of pronotum arcuate since the anterior angles to the posterior ones, slightly sinuate before the hind angles; setae strongly needle-like; elytral nodules well developed with a pattern formula 2, 3, 2, 1.

**Figure 36.**
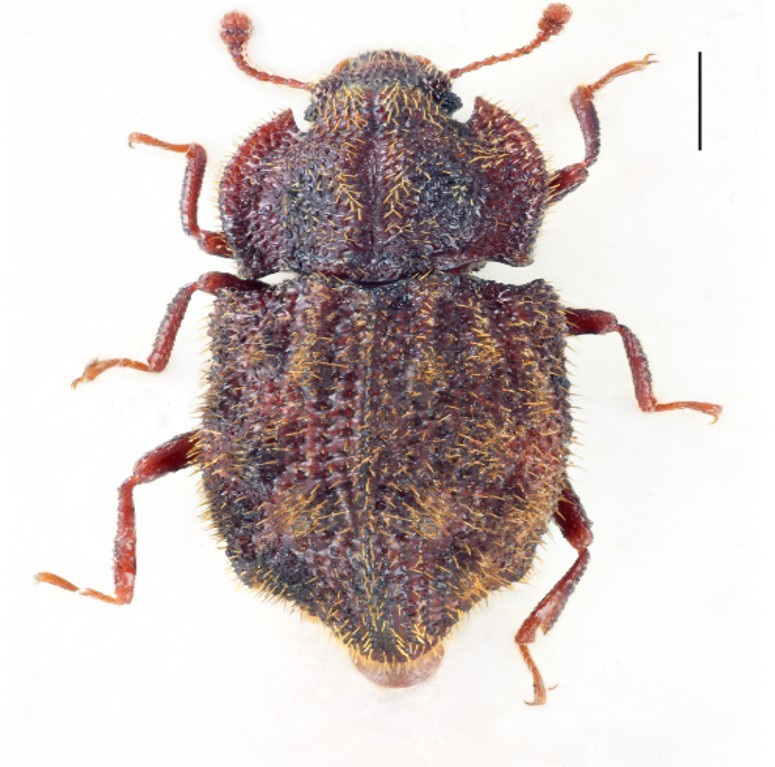
*Tarphius tornvalli* from Tronqueira (S. Miguel, Azores) (Credit: Erno-Endre Gergely). Scale 0.5 mm.

Figure(s) or Photo(s): Fig. 36

Region for assessment: Global

#### Editor & Reviewers

##### Reviewers

Reviewers: Anja Danielczak

##### Editor

Editor: Axel Hochkirch

#### Reviewers

Reviewers: Anja Danielczak

#### Editor

Editor: Axel Hochkirch

#### Geographic range

Biogeographic realm: Palearctic

Countries: Portugal

Map of records (Google Earth): Suppl. material 54

Basis of EOO and AOO: Observed

Basis (narrative): The extent of occurrence (EOO) is *ca.* 380 km^2^ and the maximum area of occupancy (AOO) is 52 km^2^.

Min Elevation/Depth (m): 500

Max Elevation/Depth (m): 1000

Range description: *Tarphius tornvalli* is a single-island endemic species restricted to São Miguel island (Azores, Portugal) (Borges et al. 2017), known from Natural Forest Reserves of Pico da Vara, Graminhais and Atalhada.

#### New occurrences

#### Extent of occurrence

EOO (km2): 380

Trend: Decline (inferred)

Justification for trend: This species occurs in native forests included in several Natural Forest Reserves of S. Miguel island. It also occurs in exotic forests (mainly dominated by *Cryptomeria japonica* plantations). The Extent of Occurrence includes habitats not occupied by this species. The species continues in decline due to native forest destruction, *Cryptomeria japonica* plantations management and due to habitat degradation by the rapid advance of invasive plant species.

Causes ceased?: No

Causes understood?: Yes

Causes reversible?: Unknown

Extreme fluctuations?: Unknown

#### Area of occupancy

Trend: Decline (inferred)

Justification for trend: The species occurs in native forests included in Natural Forest Reserves of S. Miguel island. Possibly the AOO value is overestimated. The species continues in decline due to native forest destruction, *Cryptomeria japonica* plantations management and due to habitat degradation by the rapid advance of invasive plant species.

Causes ceased?: No

Causes understood?: Yes

Causes reversible?: Unknown

Extreme fluctuations?: Unknown

AOO (km2): 52

#### Locations

Number of locations: 7

Justification for number of locations: The species occurs in several native forest patches included in Natural Forest Reserves and in *Cryptomeria* plantations, in the S. Miguel island.

Trend: Decline (inferred)

Justification for trend: In the last 50 years major alterations were made in the territory with impacts in native habitats. Seven locations that were highly impacted by invasive plants in the last ten years. These invasive plants are changing the structure of the forest and the cover of bryophytes and ferns in the soil decreasing the quality of the habitat with impacts on the species.

Extreme fluctuations?: Unknown

#### Population

Trend: Decline (inferred)

Justification for trend: The species is abundant, particularly in the well preserved patches of native forests of S. Miguel island. A continuing decline in the number of mature individuals is inferred from monitoring schemes and from the ongoing habitat degradation due to invasions of alien plants (*Hedychium gardnerianum*) (Borges et al. 2017).

Basis for decline: (c) a decline in area of occupancy, extent of occurrence and/or quality of habitat

Causes ceased?: No

Causes understood?: Yes

Causes reversible?: Unknown

Extreme fluctuations?: Unknown

#### Subpopulations

Number of subpopulations: 7

Trend: Decline (inferred)

Justification for trend: The species is abundant, particularly in the well preserved patches of native forests of S. Miguel island. A continuing decline in the number of subpopulations is inferred from monitoring schemes and from the ongoing habitat degradation due to invasions of alien plants (*Hedychium gardnerianum*) (Borges et al. 2017).

Extreme fluctuations?: Unknown

Severe fragmentation?: Yes

Justification for fragmentation: Major land-use changes at all elevations in S. Miguel island promoted the creation of small patches of native and exotic forest. The species occurs in three natural forest fragment and small patches of exotic forest that are isolated in a sea of pastures and *Cryptomeria japonica* plantations that were highly impacted by invasive plants in the last ten years.

#### Habitat

System: Terrestrial

Habitat specialist: Yes

Habitat (narrative): The species is particularly abundant, namely this species lives in the soil litter and occuring in some of the larger and well preserved patches of native forests of S. Miguel island. It also occurs under the bark of dead endemic and exotic trees (Borges et al. 2017). This species has an altitudinal range between 500 and 1000 m.

Trend in extent, area or quality?: Decline (observed)

Justification for trend: In the past, the species has probably strongly declined due to changes in habitat size and quality (Triantis et al. 2010). Currently the rapid advance of invasive plant species are decreasing the quality of the habitat (Borges et al. 2017).

##### Habitat

Habitat importance: Major Importance

Habitats: 1.4. Forest - Temperate14.3. Artificial/Terrestrial - Plantations16. Introduced vegetation

#### Habitat

Habitat importance: Major Importance

Habitats: 1.4. Forest - Temperate14.3. Artificial/Terrestrial - Plantations16. Introduced vegetation

#### Ecology

Size: 0.27 cm

Generation length (yr): 1

Dependency of single sp?: No

Ecology and traits (narrative): This is a nocturnal fungivorous species. This is an univoltine species.

#### Threats

Justification for threats: In the past, the species has probably strongly declined due to changes in habitat size and quality (Triantis et al. 2010). Currently, the rapid advance and expansion of invasive plants species is the major threat (Borges et al. 2017), particularly *Hedychium gardnerianum* and *Pittosporum undulatum* that are changing the habitat structure, namely decreasing the cover of bryophytes and ferns in the soil and promoting the spread of other plants. The management of *Cryptomeria japonica* plantations can be also a problem for the subpopulations living in this habitat. Based on Ferreira et al. 2016 the habitat will further decline as a consequence of climate change (increasing number of droughts and habitat shifting and alteration).

##### Threats

Threat type: Ongoing

Threats: 2.2.1. Agriculture & aquaculture - Wood & pulp plantations - Small-holder plantations8.1.2. Invasive and other problematic species, genes & diseases - Invasive non-native/alien species/diseases - Named species

##### Threats

Threat type: Future

Threats: 11.1. Climate change & severe weather - Habitat shifting & alteration11.2. Climate change & severe weather - Droughts

#### Threats

Threat type: Ongoing

Threats: 2.2.1. Agriculture & aquaculture - Wood & pulp plantations - Small-holder plantations8.1.2. Invasive and other problematic species, genes & diseases - Invasive non-native/alien species/diseases - Named species

#### Threats

Threat type: Future

Threats: 11.1. Climate change & severe weather - Habitat shifting & alteration11.2. Climate change & severe weather - Droughts

#### Conservation

Justification for conservation actions: The species is not protected by regional law. Its habitat is in regionally protected areas (Natural Forest Reserves of Pico da Vara, Graminhais and Atalhada, in S. Miguel island). Degraded habitats should be restored with the removal of invasive species. A strategy needs also to be developed to address the future threat by climate change. It is necessary a monitoring plan for the invertebrate community in the habitat in order to contribute to the conservation of this species. A habitat management plan is needed and anticipated to be developed during the coming years. Since this species is an icone of the relict native Azorean forests, it is suggested that some awareness measures should be put in practice.

##### Conservation actions

Conservation action type: In Place

Conservation actions: 1. Land/water protection1.1. Land/water protection - Site/area protection

##### Conservation actions

Conservation action type: Needed

Conservation actions: 2.1. Land/water management - Site/area management2.2. Land/water management - Invasive/problematic species control2.3. Land/water management - Habitat & natural process restoration4. Education & awareness5.4.3. Law & policy - Compliance and enforcement - Sub-national level

#### Conservation actions

Conservation action type: In Place

Conservation actions: 1. Land/water protection1.1. Land/water protection - Site/area protection

#### Conservation actions

Conservation action type: Needed

Conservation actions: 2.1. Land/water management - Site/area management2.2. Land/water management - Invasive/problematic species control2.3. Land/water management - Habitat & natural process restoration4. Education & awareness5.4.3. Law & policy - Compliance and enforcement - Sub-national level

#### Other

##### Use and trade

Use type: International

##### Ecosystem services

Ecosystem service type: Less important

Ecosystem services: 7. Nutrient Cycling

##### Research needed

Research needed: 1.2. Research - Population size, distribution & trends1.3. Research - Life history & ecology3.1. Monitoring - Population trends3.4. Monitoring - Habitat trends

Justification for research needed: Further research is needed into its ecology and life history in order to find extant specimens in additional areas in S. Miiguel namely in Lagoa do Fogo and Lagoa das Sete Cidades and obtain information on population size, distribution and trends. It is also necessary a monitoring plan for the invertebrate community in the habitat in order to contribute to perform a species potential recovery plan. Monitoring every ten years using the BALA protocol will inform about habitat quality (see e.g. Gaspar et al. 2011).

#### Use and trade

Use type: International

#### Ecosystem services

Ecosystem service type: Less important

Ecosystem services: 7. Nutrient Cycling

#### Research needed

Research needed: 1.2. Research - Population size, distribution & trends1.3. Research - Life history & ecology3.1. Monitoring - Population trends3.4. Monitoring - Habitat trends

Justification for research needed: Further research is needed into its ecology and life history in order to find extant specimens in additional areas in S. Miiguel namely in Lagoa do Fogo and Lagoa das Sete Cidades and obtain information on population size, distribution and trends. It is also necessary a monitoring plan for the invertebrate community in the habitat in order to contribute to perform a species potential recovery plan. Monitoring every ten years using the BALA protocol will inform about habitat quality (see e.g. Gaspar et al. 2011).

#### Viability analysis

Justification for probability: 

### Tarphius wollastoni

#### Species information

Scientific name: Tarphius wollastoni

Species authority: Crotch, 1867

Common names: Ironclad beetle (English); Escaravelho-cascudo-da-mata (Portuguese)

Kingdom: Animalia

Phylum: Arthropoda

Class: Insecta

Order: Coleoptera

Family: Zopheridae

Taxonomic notes: This was the first species of *Tarphius* described for the Azores. This species belongs to the “*azoricus+wollastoni+depressus*” complex and is characterized by (Borges et al. 2017): lateral margins of pronotum arcuate between the anterior and the hind angles; pronotal setae obtuse, rigid and decumbent elytral nodules well developed with a pattern formula 3, 3, 2, 1.

Region for assessment: Global

#### Editor & Reviewers

##### Reviewers

Reviewers: Anja Danielczak

##### Editor

Editor: Axel Hochkirch

#### Reviewers

Reviewers: Anja Danielczak

#### Editor

Editor: Axel Hochkirch

#### Geographic range

Biogeographic realm: Palearctic

Countries: Portugal

Map of records (Google Earth): Suppl. material 55

Basis of EOO and AOO: Observed

Basis (narrative): The extent of occurrence (EOO) is 8 km^2^ and the maximum area of occupancy (AOO) is 8 km^2^.

Min Elevation/Depth (m): 800

Max Elevation/Depth (m): 1000

Range description: *Tarphius wollastoni* is a single-island endemic species restricted to Faial island (Azores, Portugal) (Borges et al. 2017), known from Natural Forest Reserve of Caldeira do Faial.

#### New occurrences

#### Extent of occurrence

EOO (km2): 8

Trend: Decline (inferred)

Justification for trend: This species is very rare, occurring in the native forest of Faial island, with a reduced extent of occurrence. The species continues in decline due to habitat loss and the expansion of invasive plant species, namely *Rubus ulmifolius* and *Hedychium gardnerianum* (Borges et al. 2017).

Causes ceased?: No

Causes understood?: Yes

Causes reversible?: Unknown

Extreme fluctuations?: Unknown

#### Area of occupancy

Trend: Decline (inferred)

Justification for trend: The species occurs in a small patch of native forest, included in a Natural Forest Reserve of Faial. The AOO is overestimated, being the AOO with native forest only around 2 km². The species continues in decline due to reduced area of occupancy, habitat loss and the expansion of invasive plant species, namely *Rubus ulmifolius* and *Hedychium gardnerianum* (Borges et al. 2017).

Causes ceased?: No

Causes understood?: Yes

Causes reversible?: Unknown

Extreme fluctuations?: Unknown

AOO (km2): 8

#### Locations

Number of locations: 1

Justification for number of locations: A single fragment of native forest currently with less than 2 km^2^.

Trend: Decline (inferred)

Justification for trend: The expansion of invasive plants inside Caldeira do Faial is changing the habitat structure decreasing the cover of bryophytes and ferns in the soil with impacts on the species.

Extreme fluctuations?: Unknown

#### Population

Trend: Decline (inferred)

Justification for trend: The species is very rare and only occurs in a small patch of native forest in Faial island. A continuing decline in the number of mature individuals is inferred from monitoring schemes and from the ongoing habitat degradation due to invasions of alien plants (namely *Rubus ulmifolius* and *Hedychium gardnerianum*) (Borges et al. 2017).

Basis for decline: (c) a decline in area of occupancy, extent of occurrence and/or quality of habitat

Causes ceased?: No

Causes understood?: Yes

Causes reversible?: Unknown

Extreme fluctuations?: Unknown

#### Subpopulations

Number of subpopulations: 1

Trend: Decline (inferred)

Justification for trend: The species is very rare and only occurs in a small patch of native forest in Faial island. The expansion of invasive plants may lead to the extinction of the single subpopulation.

Extreme fluctuations?: Unknown

Severe fragmentation?: No

#### Habitat

System: Terrestrial

Habitat specialist: Yes

Habitat (narrative): The species is very rare, and only occurs in a small patch of native forest in Faial island, Caldeira do Faial (Borges et al. 2017). The species can also occur associated with *Cryptomeria japonica* in a small patch near Caldeira do Faial. It has an altitudinal range between 800 and 1000 m.

Trend in extent, area or quality?: Decline (observed)

Justification for trend: In the past, the species has probably strongly declined due to changes in habitat size and quality (Triantis et al. 2010). Currently the rapid advance of invasive plant species are decreasing the quality of the habitat (Borges et al. 2017).

##### Habitat

Habitat importance: Major Importance

Habitats: 1.4. Forest - Temperate

#### Habitat

Habitat importance: Major Importance

Habitats: 1.4. Forest - Temperate

#### Ecology

Size: 0.31 cm

Generation length (yr): 1

Dependency of single sp?: No

Ecology and traits (narrative): This is a nocturnal fungivorous species that lives in the soil and under bark of endemic trees. This is an univoltine species.

#### Threats

Justification for threats: In the past, the species has probably strongly declined due to changes in habitat size and quality (Triantis et al. 2010). Currently, the rapid advance and expansion of invasive plants species is the major threat (Borges et al. 2017), particularly *Hedychium gardnerianum* and *Rubus ulmifolius* that are changing the habitat structure, namely decreasing the cover of bryophytes and ferns in the soil and promoting the spread of other plants. Based on Ferreira et al. 2016 the habitat will further decline as a consequence of climate change (increasing number of droughts and habitat shifting and alteration).

##### Threats

Threat type: Ongoing

Threats: 2.2.1. Agriculture & aquaculture - Wood & pulp plantations - Small-holder plantations8.1.2. Invasive and other problematic species, genes & diseases - Invasive non-native/alien species/diseases - Named species

##### Threats

Threat type: Future

Threats: 11.1. Climate change & severe weather - Habitat shifting & alteration11.2. Climate change & severe weather - Droughts

#### Threats

Threat type: Ongoing

Threats: 2.2.1. Agriculture & aquaculture - Wood & pulp plantations - Small-holder plantations8.1.2. Invasive and other problematic species, genes & diseases - Invasive non-native/alien species/diseases - Named species

#### Threats

Threat type: Future

Threats: 11.1. Climate change & severe weather - Habitat shifting & alteration11.2. Climate change & severe weather - Droughts

#### Conservation

Justification for conservation actions: The species is not protected by regional law. Its habitat is in a regionally protected area (Natural Reserve of Caldeira do Faial in Faial island). Degraded habitats should be restored with the removal of invasive species. A strategy needs also to be developed to address the future threat by climate change. It is necessary a monitoring plan for the invertebrate community in the habitat in order to contribute to the conservation of this species. A habitat management plan is needed and anticipated to be developed during the coming years. Since this species is an icone of the relict native Azorean forests, it is suggested that some awareness measures should be put in practice.

##### Conservation actions

Conservation action type: In Place

Conservation actions: 1. Land/water protection1.1. Land/water protection - Site/area protection

##### Conservation actions

Conservation action type: Needed

Conservation actions: 2.1. Land/water management - Site/area management2.2. Land/water management - Invasive/problematic species control2.3. Land/water management - Habitat & natural process restoration4. Education & awareness5.4.3. Law & policy - Compliance and enforcement - Sub-national level

#### Conservation actions

Conservation action type: In Place

Conservation actions: 1. Land/water protection1.1. Land/water protection - Site/area protection

#### Conservation actions

Conservation action type: Needed

Conservation actions: 2.1. Land/water management - Site/area management2.2. Land/water management - Invasive/problematic species control2.3. Land/water management - Habitat & natural process restoration4. Education & awareness5.4.3. Law & policy - Compliance and enforcement - Sub-national level

#### Other

##### Use and trade

Use type: International

##### Ecosystem services

Ecosystem service type: Less important

Ecosystem services: 7. Nutrient Cycling

##### Research needed

Research needed: 1.2. Research - Population size, distribution & trends1.3. Research - Life history & ecology2.2. Conservation Planning - Area-based Management Plan3.1. Monitoring - Population trends3.4. Monitoring - Habitat trends

Justification for research needed: Further research is needed into its ecology and life history in order to find extant specimens in additional areas with native forest around Caldeira do Faial (namely some small patches in water streams) and obtain information on population size, distribution and trends. It is also necessary an area-based management plan and a monitoring plan for the invertebrate community in the habitat in order to contribute to perform a species potential recovery plan. Monitoring every ten years using the BALA protocol will inform about habitat quality (see e.g. Gaspar et al. 2011).

#### Use and trade

Use type: International

#### Ecosystem services

Ecosystem service type: Less important

Ecosystem services: 7. Nutrient Cycling

#### Research needed

Research needed: 1.2. Research - Population size, distribution & trends1.3. Research - Life history & ecology2.2. Conservation Planning - Area-based Management Plan3.1. Monitoring - Population trends3.4. Monitoring - Habitat trends

Justification for research needed: Further research is needed into its ecology and life history in order to find extant specimens in additional areas with native forest around Caldeira do Faial (namely some small patches in water streams) and obtain information on population size, distribution and trends. It is also necessary an area-based management plan and a monitoring plan for the invertebrate community in the habitat in order to contribute to perform a species potential recovery plan. Monitoring every ten years using the BALA protocol will inform about habitat quality (see e.g. Gaspar et al. 2011).

#### Viability analysis

Justification for probability: 

## Discussion

Most species assessed are single-island endemics with a very restricted distribution (66% occur in only one island) and having very small extent of occurrence (EOO) and area of occupancy (AOO). The grid cell used by IUCN will always overestimate the AOO in small island territories (Martín 2009, Cardoso et al. 2011). The real area of native forest patches in which most of the Azorean beetles still persist is very small, i.e, in 14 out of the 20 fragments the area is less than the 4 km^2^ corresponding to the IUCN grid cells. Therefore, the minimum AOO value as calculated by IUCN criteria in GeoCAT is already an overestimate of the real area of occupancy for most species and we did not use species distribution modelling as proposed by Cardoso et al. 2011. In fact, all the species restricted to the unique patch of native forest from S. Maria island (Pico Alto) occupy an area of only 0.09 km^2^, which with the addition of high levels of habitat destruction by invasive plants makes this small forest patch a road to extinction (Triantis et al. 2010, Terzopoulou et al. 2015).

Most of the species are now restricted to the Azorean network of protected areas (RAA 2012), but also common to some of the species is the fragmentation of their subpopulations, a continuing decline in EOO, AOO, habitat quality, number of locations and subpopulations caused by the ongoing threats from pasture intensification, forestry (*Cryptomeria japonica* pulp plantations management), invasive species (particularly *Pittosporum undulatum* and *Hedychium gardnerianum*) and future climatic changes (Ferreira et al. 2016). Therefore, we suggest as future measures of conservation: (1) a long-term monitoring plan for the species; (2) control of invasive species; (3) species-specific conservation action plans for the most highly threatened species.

The Azorean 18 Natural Forest Reserves were monitored on 2000 and 2010 (see Borges et al. 2016) and are expected to be monitored again in 2020. Monitoring every ten years using the BALA protocol will inform about habitat quality (see e.g. Gaspar et al. 2011) and allow a better understand on the future trends in EOO, AOO, habitat quality, number of locations and subpopulations for the Azorean endemic beetles.

Discussion

## Supplementary Material

Supplementary material 1 Species raw dat Data type: Occurrences
Brief description: Raw data on the species island distribution, AOO, EOO, altitudinal range and number of localities.
File: oo_140085.xlsx Borges, P. A. V. & Lamelas-Lopez, L.

Supplementary material 2 *Bembidion derelictus* map Data type: Map Google Earth
Brief description: Distribution of *Bembidion derelictus*
File: oo_137790.kmz Anja Danielczak

Supplementary material 3 *Bradycellus chavesi* map Data type: Map Google Earth
Brief description: Distribution of *Bradycellus chavesi*
File: oo_137791.kmz Anja Danielczak

Supplementary material 4 *Calathus carvalhoi* map Data type: Map Google Earth
Brief description: Distribution of *Calathus carvalhoi*
File: oo_137789.kmz Anja Danielczak

Supplementary material 5 *Calathus extensicollis* map Data type: Map Google Earth
Brief description: Distribution of *Calathus extensicollis*
File: oo_137823.kmz Anja Danielczak

Supplementary material 6 *Calathus lundbladi* map Data type: Map Google Earth
Brief description: Distribution of *Calathus lundbladi*
File: oo_137825.kmz Anja Danielczak

Supplementary material 7 *Calathus vicenteorum* map Data type: Map Google Earth
Brief description: Distribution of *Calathus vicenteorum*
File: oo_137826.kmz Anja Danielczak

Supplementary material 8 *Cedrorum azoricus azoricus* and *Cedrorum azoricus caveirensis* map Data type: Map Google Earth
Brief description: Distribution of *Cedrorum azoricus azoricus* and *Cedrorum azoricus caveirensis*
File: oo_137829.kmz Anja Danielczak

Supplementary material 9 *Olisthopus inclavatus* map Data type: Map Google Earth
Brief description: Distribution of *Olisthopus inclavatus*
File: oo_137861.kmz Anja Danielczak

Supplementary material 10 *Pseudanchomenus aptinoides* map Data type: Map Google Earth
Brief description: Distribution of *Pseudanchomenus aptinoides*
File: oo_137860.kmz Anja Danielczak

Supplementary material 11 *Trechus terrabravensis* map Data type: Map Google Earth
Brief description: Distribution of *Trechus terrabravensis*
File: oo_137865.kmz Anja Danielczak

Supplementary material 12 *Trechus torretassoi* map Data type: Map Google Earth
Brief description: Distribution of *Trechus torretassoi*
File: oo_137866.kmz Anja Danielczak

Supplementary material 13 *Mniophilosoma obscurum* map Data type: Map Google Earth
Brief description: Distribution of *Mniophilosoma obscurum*
File: oo_137868.kmz Anja Danielczak

Supplementary material 14 *Atlantocis gillerforsi* map Data type: Map Google Earth
Brief description: Distribution of *Atlantocis gillerforsi*
File: oo_137870.kmz Anja Danielczak

Supplementary material 15 *Calacalles azoricus* map Data type: Map Google Earth
Brief description: Distribution of *Calacalles azoricus*
File: oo_137875.kmz Anja Danielczak

Supplementary material 16 *Calacalles droueti* map Data type: Map Google Earth
Brief description: Distribution of *Calacalles droueti*
File: oo_137876.kmz Anja Danielczak

Supplementary material 17 *Calacalles subcarinatus* map Data type: Map Google Earth
Brief description: Distribution of *Calacalles subcarinatus*
File: oo_137877.kmz Anja Danielczak

Supplementary material 18 *Caulotrupis parvus* map Data type: Map Google Earth
Brief description: Distribution of *Caulotrupis parvus*
File: oo_137896.kmz Anja Danielczak

Supplementary material 19 *Donus multifidus* map Data type: Map Google Earth
Brief description: Distribution of *Donus multifidus*
File: oo_137897.kmz Anja Danielczak

Supplementary material 20 *Drouetius azoricus* map Data type: Map Google Earth
Brief description: Distribution of *Drouetius azoricus*
File: oo_137903.kmz Anja Danielczak

Supplementary material 21 *Drouetius borgesi* map Data type: Map Google Earth
Brief description: Distribution of *Drouetius borgesi*
File: oo_137904.kmz Anja Danielczak

Supplementary material 22 *Drouetius oceanicus* map Data type: Map Google Earth
Brief description: Distribution of *Drouetius oceanicus*
File: oo_137905.kmz Anja Danielczak

Supplementary material 23 *Neocnemis occidentalis* map Data type: Map Google Earth
Brief description: Distribution of *Neocnemis occidentalis*
File: oo_137948.kmz Anja Danielczak

Supplementary material 24 *Phloeosinus gillerforsi* map Data type: Map Google Earth
Brief description: Distribution of *Phloeosinus gillerforsi*
File: oo_137949.kmz Anja Danielczak

Supplementary material 25 *Pseudechinosoma nodosum* map Data type: Map Google Earth
Brief description: Distribution of *Pseudechinosoma nodosum*
File: oo_137951.kmz Anja Danielczak

Supplementary material 26 *Sphaericus velhocabrali* map Data type: Map Google Earth
Brief description: Distribution of *Sphaericus velhocabrali*
File: oo_137952.kmz Anja Danielczak

Supplementary material 27 *Athous azoricus* map Data type: Map Google Earth
Brief description: Distribution of *Athous azoricus*
File: oo_137953.kmz Anja Danielczak

Supplementary material 28 *Athous pomboi* map Data type: Map Google Earth
Brief description: Distribution of *Athous pomboi*
File: oo_137954.kmz Anja Danielczak

Supplementary material 29 *Heteroderes azoricus* map Data type: Map Google Earth
Brief description: Distribution of *Heteroderes azoricus*
File: oo_137955.kmz Anja Danielczak

Supplementary material 30 *Cryptolestes azoricus* map Data type: Map Google Earth
Brief description: Distribution of *Cryptolestes azoricus*
File: oo_137956.kmz Anja Danielczak

Supplementary material 31 *Metophthalmus occidentalis* map Data type: Map Google Earth
Brief description: Distribution of *Metophthalmus occidentalis*
File: oo_137957.kmz Anja Danielczak

Supplementary material 32 *Catops velhocabrali* map Data type: Map Google Earth
Brief description: Distribution of *Catops velhocabrali*
File: oo_137959.kmz Anja Danielczak

Supplementary material 33 *Aleochara freyi* map Data type: Map Google Earth
Brief description: Distribution of *Aleochara freyi*
File: oo_137960.kmz Anja Danielczak

Supplementary material 34 *Atheta azorica* map Data type: Map Google Earth
Brief description: Distribution of *Atheta azorica*
File: oo_137961.kmz Anja Danielczak

Supplementary material 35 *Atheta caprariensis* map Data type: Map Google Earth
Brief description: Distribution of *Atheta caprariensis*
File: oo_137962.kmz Anja Danielczak

Supplementary material 36 *Atheta dryochares* map Data type: Map Google Earth
Brief description: Distribution of *Atheta dryochares*
File: oo_137963.kmz Anja Danielczak

Supplementary material 37 *Atheta floresensis* map Data type: Map Google Earth
Brief description: Distribution of *Atheta floresensis*
File: oo_148541.kmz Anja Danielczak

Supplementary material 38 *Euconnus azoricus* map Data type: Map Google Earth
Brief description: Distribution of *Euconnus azoricus*
File: oo_137966.kmz Anja Danielczak

Supplementary material 39 *Geostiba melanocephala* map Data type: Map Google Earth
Brief description: Distribution of *Geostiba melanocephala*
File: oo_137967.kmz Anja Danielczak

Supplementary material 40 *Medon varamontis* map Data type: Map Google Earth
Brief description: Distribution of *Medon varamontis*
File: oo_137968.kmz Anja Danielczak

Supplementary material 41 *Phloeostiba azorica* map Data type: Map Google Earth
Brief description: Distribution of *Phloeostiba azorica*
File: oo_137969.kmz Anja Danielczak

Supplementary material 42 *Phytosus schatzmayri* map Data type: Map Google Earth
Brief description: Distribution of *Phytosus schatzmayri*
File: oo_137970.kmz Anja Danielczak

Supplementary material 43 *Nesotes azoricus* map Data type: Map Google Earth
Brief description: Distribution of *Nesotes azoricus*
File: oo_137971.kmz Anja Danielczak

Supplementary material 44 *Tarphius acuminatus* map Data type: Map Google Earth
Brief description: Distribution of *Tarphius acuminatus*
File: oo_137974.kmz Anja Danielczak

Supplementary material 45 *Tarphius azoricus* map Data type: Map Google Earth
Brief description: Distribution of *Tarphius azoricus*
File: oo_137975.kmz Anja Danielczak

Supplementary material 46 *Tarphius depressus* map Data type: Map Google Earth
Brief description: Distribution of *Tarphius depressus*
File: oo_137976.kmz Anja Danielczak

Supplementary material 47 *Tarphius floresensis* map Data type: Map Google Earth
Brief description: Distribution of *Tarphius floresensis*
File: oo_137977.kmz Anja Danielczak

Supplementary material 48 *Tarphius furtadoi* map Data type: Map Google Earth
Brief description: Distribution of*Tarphius furtadoi*
File: oo_137995.kmz Anja Danielczak

Supplementary material 49 *Tarphius gabrielae* map Data type: Map Google Earth
Brief description: Distribution of *Tarphius gabrielae*
File: oo_137996.kmz Anja Danielczak

Supplementary material 50 *Tarphius pomboi* map Data type: Map Google Earth
Brief description: Distribution of *Tarphius pomboi*
File: oo_137997.kmz Anja Danielczak

Supplementary material 51 *Tarphius relictus* map Data type: Map Google Earth
Brief description: Distribution of *Tarphius relictus*
File: oo_137998.kmz Anja Danielczak

Supplementary material 52 *Tarphius rufonodulosus* map Data type: Map Google Earth
Brief description: Distribution of *Tarphius rufonodulosus*
File: oo_137999.kmz Anja Danielczak

Supplementary material 53 *Tarphius serranoi* map Data type: Map Google Earth
Brief description: Distribution of *Tarphius serranoi*
File: oo_138000.kmz Anja Danielczak

Supplementary material 54 *Tarphius tornvalli* map Data type: Map Google Earth
Brief description: Distribution of *Tarphius tornvalli*
File: oo_138002.kmz Anja Danielczak

Supplementary material 55 *Tarphius wollastoni* map Data type: Map Google Earth
Brief description: Distribution of *Tarphius wollastoni*
File: oo_138003.kmz Anja Danielczak

## Hosting institution

Universidade dos Açores, Center for Ecology, Evolution and Environmental Changes (cE3c) and Azorean Biodiversity Group.

## Hosting institution

Universidade dos Açores, Center for Ecology, Evolution and Environmental Changes (cE3c) and Azorean Biodiversity Group.
